# Motor neuron survival is associated with reduced neuroinflammation and increased autophagy after brachial plexus avulsion injury in aldose reductase-deficient mice

**DOI:** 10.1186/s12974-022-02632-6

**Published:** 2022-11-09

**Authors:** Ke Zhong, Yu Huang, Prince last Mudenda Zilundu, Yaqiong Wang, Yingying Zhou, Guangyin Yu, Rao Fu, Sookja Kim Chung, Yamei Tang, Xiao Cheng, Lihua Zhou

**Affiliations:** 1 Department of Pharmacy, Sun-Yat-Sen Memorial Hospital, Sun Yat-sen University, 107 Yanjiang West Road, Guangzhou, 510120 Guangdong China; 2 Department of Anatomy, School of Medicine (Shenzhen), Sun Yat-sen University, Shenzhen, 518000 Guangdong China; 3 Department of Anatomy, Zhongshan School of Medicine, Sun Yat-sen University, Guangzhou, Guangdong China; 4 Department of Electron Microscope, Zhongshan School of Medicine, Sun Yat-Sen University, Guangzhou, Guangdong China; 5 Department of Anatomy, Neuroscience Laboratory for Cognitive and Developmental Disorders, Medical College of Jinan University, Guangzhou, Guangdong China; 6 Faculty of Medicine, Macau University of Science and Technology, Macau, China; 7 Department of Neurology, Sun Yat-sen Memorial Hospital, Sun Yat‑sen University, Guangzhou, China; 8 Key Laboratory of Malignant Tumor Gene Regulation and Target Therapy of Guangdong Higher Education Institutes, Sun Yat-sen University, Guangzhou, China; 9 Guangdong Province Key Laboratory of Brain Function and Disease, Zhongshan School of Medicine, Sun Yat-sen University, Guangzhou, China; 10 Guangdong Provincial Chinese Emergency Key Laboratory, Guangzhou, Guangdong China; 11 State Key Laboratory of Dampness, Syndrome of Traditional Chinese Medicine, Guangzhou, Guangdong China; 12 Department of Neurology, Guangdong Provincial Hospital of Traditional Chinese Medicine, 111 Dade Road, Guangzhou, Guangdong China

**Keywords:** Aldose reductase, Brachial plexus root avulsion, Motoneurons death, Autophagy, Neuroinflammation

## Abstract

Brachial plexus root avulsion (BPRA) is frequently caused by high-energy trauma including traffic accident and birth trauma, which will induces massive motoneurons (MNs) death as well as loss of motor and sensory function in the upper limb. The death of MNs is attributed to energy deficiency, neuroinflammation and oxidative stress at the injured ventral horn of spinal cord triggered by BPRA injury. It has been reported which aldose reductase (AR), an endogenous enzyme that catalyzes fructose synthesis, positively correlates with the poor prognosis following cerebral ischemic injury, diabetic retinopathy and diabetic peripheral neuropathy. However, the role of AR in BPRA remains unknown. Herein, we used a mouse model and found that in the spinal cord of BPRA mice, the upregulation of AR correlated significantly with (1) an inactivated SIRT1–AMPK–mTOR pathway and disrupted autophagy; (2) increased byproducts accumulation of lipid peroxidation metabolism and neuroinflammation; and (3) increased MNs death. Furthermore, our results demonstrated the role of AR in BPRA injury whereby the absence of AR (AR knockout mice, AR^−/−^) prevented the hyper-neuroinflammation and disrupted autophagy as well as motor neuron death caused by BPRA injury. Finally, we further demonstrate that AR inhibitor epalrestat is neuroprotective against BPRA injury by increasing autophagy level, alleviating neuroinflammation and rescuing MNs death in mice. Collectively, our data demonstrate that the AR upregulation in the spinal cord is an important factor contributing to autophagy disruption, neuroinflammation and MNs death following brachial plexus roots avulsion in mice. Our study also provides a promising therapy drug to assist re-implantation surgery for the treatment of BPRA.

**Supplementary Information:**

The online version contains supplementary material available at 10.1186/s12974-022-02632-6.

## Background

Brachial plexus roots avulsion (BPRA), mainly resulting from road traffic accidents or difficult childbirths, causes upper limb motor and sensory impairments which in turn seriously affects patient quality of life [1]. Motor neurons (MNs) tend to progressively degenerate and then die following brachial plexus root avulsion, thus leading to paralysis of the denervated muscles of the upper limbs [2]. Presently, numerous therapies including nerve root surgical reimplantation [3], local application of neurotrophic factors [4] and cell transplantation strategies [5] have been adopted. However, the neurological functional recovery remains unsatisfactory. Early and effective neuroprotective strategies are crucial to prolong the surgery therapeutic window and, thus, are essential to promote functional recovery following BPRA. Therefore, exploring novel medical approaches towards the treatment of BPRA is vital.

The pathophysiological sequelae of primary avulsion injury in the spinal cord include ion homeostatic disequilibrium, heightened glutamate excitotoxicity, energy deficiency, disruption of normal mitochondrial function, and integrity of micro vessels, leading to organelle damage, toxic protein aggregation and progressive MNs loss [1]. Autophagy, a lysosome-dependent catabolic process, is responsible for degrading cytoplasm-based proteins, their aggregates and damaged organelles [6]. Previous studies revealed an aberrant expression of autophagic associated proteins in postmortem central nervous system samples obtained from animal models of amyotrophic lateral sclerosis and patients. These studies provided preliminary evidence that autophagy played a significant function in driving MNs death [7].

Besides, another important hallmark of BPRA is neuroinflammation in the injured spinal cord, which is one of the indispensable processes related to the aftermath of nervous tissue damage as well as regeneration [8]. In addition, neuroinflammation aggravated degeneration and death of MNs after different types of spinal cord injuries, including BPRA [9]. Inflammation following BPRA involves glial activation (activated astrocytes and microglia) which triggers excess release of many growth factors as well as mediators of oxidative stress and inflammation [10].

AR belongs to the aldo–keto reductase (AKR) superfamily. The NADPH-dependent AR is the sole enzyme that catalyzes the metabolism of excess glucose into sorbitol via the polyol glucose metabolism pathway [11]. Moreover, the NAD^+^-dependent enzyme sorbitol dehydrogenase oxidizes sorbitol into fructose. Therefore, AR will deplete cellular NADPH and elevate cytosolic NADH/NAD^+^ ratios, which will reduce the activity of NAD^+^-dependent deacetylase Sirtuin-1 (SIRT1) [12]. Furthermore, other studies have reported that AR was also involved in the reductions of lipid peroxidation-derived, higher affinity-aldehydes, especially the transformation of 4-hydroxy-2-nonenal (4-HNE) to glutathionyl-1,4-dihydroxynonanol (GS-DHN) [13]. Subsequently, the GS-DHN appears to be a mediator in inflammatory signaling [14].

However, there are few studies evaluating the role and the plausible molecular mechanisms of AR in the context of BPRA and MNs survival. In this study, we demonstrated elevated levels of AR concomitant with neuroinflammation, disrupted autophagy and MNs death in BPRA mice model. Additionally, we established a causal role of AR in MNs death using the AR knockout mice subjected to BPRA. Finally, we provided a proof of concept that pharmacological inhibition of AR is a potential therapeutic strategy to attenuate MNs death in BPRA.

## Materials and methods

### Animals

AR gene knockout mice (AR^−/−^) in our stock were generated as described previously [15]. Age-matched normal C57BL/6 mice aged between 8 and 10 weeks and weighing 20–25 g were used as controls (wild-type mice, AR^+/+^). The wild-type mice were purchased from the Laboratory Animal Center of Sun Yat-sen University, Guangzhou city in China. The experimental animal use permit number is SYXK 2017-0081. The AR inhibitor epalrestat (EPAL, 40 mg/kg bodyweight) was administrated by daily (afternoon) oral gavage for 14 days to mice after receiving BPRA injury [16]. All the mice were housed under a 12-h light/dark cycle with unlimited access to standard mice chow and water available. The Chinese National Health and Medical Research Council animal ethics and ARRIVE guidelines were followed in executing surgical and animal care procedures. The Animal Care and Utilization Committee of Sun Yat-sen University approved all the experimental procedures.

### Animal surgery

The BPRA surgery was carried out aseptically as described in previous publications [17, 18]. Briefly, the avulsion groups mice were anesthetized with intraperitoneal injections of ketamine/xylazine (80/20 mg/kg) and maintained with 1% isoflurane. While in supine position, the right-side brachial plexus divisions, trunks and roots were carefully exposed, and the C5–T1 spinal nerve roots identified and then separated under a dissecting microscope (Cheng He Microsurgical Instruments Factory, Ning Bo, China). Micro-hemostatic forceps were used to grasp and pull out the dorsal and ventral rootlets of the region of interest. The spinal nerve region was cut immediately before the dorsal ganglion so that the proximal part bearing nerve rootlets was examined under a microscope to confirm whether or not the avulsion model succeeded. The muscles, fascia and skin were sutured in successively to close the surgical wound. The mice were then transferred into a pre-heated recovery chamber until fully awake and finally placed back to their home cages. In the sham control group, the laminectomy was performed to expose the right-side brachial plexus but avulsion was not carried out.

### Tissue preparation

At several time points (1, 3, 7 and 14 days post-injury, dpi) following BPRA, the mice were anaesthetized using an intraperitoneal injection of 1% sodium pentobarbital (40 mg/kg, Sigma–Aldrich). An incision was made on the dorsal part of the next to expose the muscles and separate them. A bilateral laminectomy was then performed under a dissection microscope to expose the C5 to T1 spinal segments. The segments were rapidly dissected off and immediately frozen in liquid nitrogen for the ease of division into ipsilateral and contralateral halves. The cut spinal cord segments were then stored in liquid nitrogen until needed for PCR, WB and ELISA. For histology studies, the roots avulsed mice were killed as mentioned above at days 1, 3, 7 and 14 dpi (*n* = 5/group). Their blood was washed out by transcardial perfusion with 0.9% normal saline and then fixed with pre-cooled 4% paraformaldehyde in 0.1 M phosphate buffer (pH 7.4). Bilateral laminectomy was carried out under a dissection microscope to expose the C7–C8 spinal segments. The segments of interest were carefully dissected out and post-fixed in 4% PFA for 3–4 h followed by overnight immersion in 30% (v/v) sucrose in PB solution in a fridge set at 4 °C. 30-μm transverse sections of the C7–C8 segments were cut on a freeze microtome and every third section was collected into 0.01 M PBS for use in staining described below.

### Neutral red staining

MNs loss was determined by neutral red staining as previously reported [17]. 1% neutral red (N4638, Sigma–Aldrich, USA) in 0.1 M acetic acid (pH 4.8) was used to stain sections for 2 h and then followed by a series of dehydration in graded concentrations of ethanol. The images were captured Nikon light microscope using the bright field feature. The counting of motor neurons was conducted by 2 laboratory personnel blinded to the injury or treatment conditions of the mice. The MNs loss was determined by its survival rate (ratio of ipsilateral/contralateral MNs number). Only large soma neurons bearing a visible nucleolus and located in lamina IX of Rexed were enumerated.

### Immunofluorescence

A Nikon Eclipse 90i, fluorescence microscope (Nikon, Japan) was used for immunofluorescence evaluations. The spinal cord slices were rinsed thrice in PBS and then incubated in 0.3% Triton X-100/0.1% bovine serum albumin for half an hour at room temperature. The slices where then incubated with the following primary antibodies; mouse anti-NeuN (Abcam UK), rabbit anti-GFAP (Cell Signaling Technology Inc. USA), rabbit anti-IBA-1 (Wako Bioproducts, USA), rabbit anti-Cleaved Caspase-3 (Cell Signaling Technology Inc. USA), rabbit anti-Bcl-2 (Cell Signaling Technology Inc. USA), rabbit anti-AMPKα (Cell Signaling Technology Inc. USA), rabbit anti-Beclin1 (Cell Signaling Technology Inc. USA), rabbit anti-GAP43 (Cell Signaling Technology Inc. USA), rabbit anti-mTOR (Cell Signaling Technology Inc. USA), rabbit anti-p-mTOR (Cell Signaling Technology Inc. USA), rabbit anti-p-CREB (Cell Signaling Technology Inc. USA), rabbit anti-LC3B (Cell Signaling Technology Inc. USA), rabbit anti-SIRT1 (Abcam UK), rabbit anti-P62 (Abcam UK), rabbit anti-4-HNE (Abcam UK), rabbit anti-p-AMPKα (Abcam UK), mouse anti-Arginase1 (Santa Cruz Biotechnology Inc USA), goat anti-CD16/32 (R&D Systems Inc USA) and goat anti-ChAT (1:500, Millipore USA) These slices were incubated with primary antibodies at 4 °C overnight. The slices were then washed thrice with PBS, and then incubated in darkness with the following secondary antibodies for 1–2 h at room temperature; goat anti-mouse with Alexa Fluor-488 (Invitrogen USA), goat anti-rabbit with Alexa Fluor-555 (Invitrogen USA), donkey anti-goat with Alexa Fluor-488 (Invitrogen USA), and donkey anti-rabbit with Alexa Fluor-555 (Invitrogen USA). After the secondary antibody incubation, the slices were rinsed thrice in PBS and then incubated in Hoechst 33,342 (H3570, Life Technologies, USA). Finally, the slices were air-dried, cover-slipped and observed under a fluorescence microscope. Omitting of either a primary or secondary antibody was used as a as a negative control. We calculated the mean number of immunoreactive cells from the total 10-immunofluorescence sections serially obtained from each mice (Every third sections from each mice used for staining and counting). The immunoreactive cells were quantified under the 20 × magnification in six circular areas of 100 μm^2^ which is located in lamina IX of Rexed of spinal cord ipsilateral ventral horn. Two independent persons blinded to the sidedness of the groups performed cell counting, pooling of means and data analysis following previously published protocols [18].

### Transmission electron microscopy

Transmission electron microscopy (TEM) was used to evaluate mitochondrial and autophagosome morphology in the spinal cord motor neurons following brachial plexus roots avulsion (days 1, 3, 7, and 14 post-injury; *n* = 5 mice). The deeply anesthetized mice were transcardially perfused using 0.9% normal saline until the exudate was clear of blood stain and then fixed using a mixture of 2% PFA and 2.5% glutaraldehyde (Sigma–Aldrich, G6257, USA) in 0.1 M PBS. A 1 mm^3^ piece of ventral horn grey matter tissue located in lamina IX of Rexed per mouse was cut off and post-fixed in 2% glutaraldehyde for another 2 h at 4 °C. The glutaraldehyde-fixed tissue was then washed thrice in 0.1 M cacodylate buffer and further post-fixed in 1% osmium tetroxide for another 2 h. After three rinses in distilled water, the pieces were then dehydrated in a graded series of ethanol concentrations. A half-acetone and half-resin mixture was used to infiltrate the tiny tissue pieces overnight at 4 °C. The tissues were embedded in resin and then cured under the following settings: (1) 37 °C overnight, (2) 45 °C for 12 h, and (3) 60 °C for 24 h. Afterward, a vibratome was used to obtain 70-nm thin sections which were then stained with 3% uranyl acetate for 20 min followed by 0.5% lead citrate for 5 min. Ultrastructural changes in the motor neurons were evaluated under the transmission electron microscope (Philips CM 10, Eindhoven, Netherlands) operated at 100 kV.

### Enzyme-linked immunosorbent assay (ELISA)

Spinal cord tissue cytokines were assayed using ELISA kits for IL-1β (Mouse IL-1 beta/IL-1F2 Quantikine ELISA Kit, MLB00C), IL-6 (Mouse IL-6 Quantikine ELISA Kit, M6000B), ICAM1 (Mouse ICAM-1/CD54 Quantikine ELISA Kit, MIC100) and IL-10 Mouse IL-10 Quantikine ELISA Kit, M1000B), which were all from R&D Systems. The assays were performed according to the manufacturer’s instructions.

### Real-time quantitative reverse transcription PCR (qRT-PCR)

TRIzol Reagent (Invitrogen) was used for Total RNA extraction following recommended procedures laid down by the kit manufacturer. RNA concentration and purity (260/280 ratio) were measured using UV spectroscopy. The total RNA was reverse-transcribed using the Fast Quant RT Kit (with gDNase) (TIANGEN, China) following the manufacturer’s recommended steps. Then, real-time PCR was performed using SYBR Green assays (TIANGEN, China) according to the manufacturer’s instructions. All primers used in this study are listed in Table 1. The PCR settings on the CFX96 touch detection system (Bio–Rad, Hercules, CA, USA) were set as follows: a denaturation step-95 °C for 15 min followed by 40 × PCR cycles at 95 °C for 10 s and 60 °C for 30 s. For quantitative results, the expression of mRNA was represented as the fold change using the 2^−ΔΔct^ method and was normalized to the control gene GAPDH. Messenger RNA expression differences between the avulsion groups and sham operated group were compared using Student’s *t*-test in SPSS (IBM SPSS Inc Version 22.0 USA). A *p*-value less than 0.05 was deemed statistically significant.

**Table 1:** Gene primers used for real-time PCR analyses

Sequence name	Primer sequence
AR	F:5′- ACGGCTATGGAACAACTA-3′
	R:5′- TGTGGCAGTATTCAATCAG-3′
Arg1	F:5′- GAACACGGCAGTGGCTTTAAC-3′
	R:5′- TGCTTAGCTCTGTCTGCTTTGC-3′
IL10	F:5′- GCAGCTCTAGGAGCATGTGG-3′
	R:5′-TGGGACCTCCTTCCCCACAA-3′
iNOS	F:5′- CCCTTCAATGGTTGGTACATGG-3′
	R:5′- ACATTGATCTCCGTGACAGCC-3′
IL-1b	F:5′- CTTCAGGCAGGCAGTATCAC-3′
	R:5′- CAGCAGGTTATCATCATCATCC-3′
GAPDH	F:5′-CCCTGAGCTGAACGGGAAGCTCAC-3′
	R:5′- CTTGCTGTAGCCAAATTCGTTGCT-3′

### Western blotting

An electric homogenizer was used to process frozen ventral horn tissue samples in a whole-cell lysis buffer (Key GEN Biotech) mixed with a protease inhibitor and 1 mM PMSF (Sigma–Aldrich). The homogenates were left on ice for 1 h and then centrifuged at 12,000 rpm for 30 min at 4 °C. The resultant supernatants were pipetted out into new tubes. A small amount from each tube was used for protein concentrations using the BCA (Thermo Scientific) method. Equal amounts of protein from each sample were then run through the 10% SDS–PAGE and electro-transferred onto respective polyvinylidene fluoride (PVDF) membranes (Millipore, 0.45 μm) using Transblot Turbo (Bio–Rad, USA) at 300 mA for 1 h. The membranes bearing electro-transferred proteins were incubated with 5% nonfat milk/Tris buffer containing Tween-20 buffer (TBST; 10 mM Tris–HCl, pH 8.0; 150 mM NaCl; and 0.1% Tween 20) for 2 h at room temperature. The strips corresponding to target proteins’ molecular weights were then incubated in the primary antibodies, listed in the immunofluorescence method section, at 4 °C overnight. The membranes were then washed thrice with TBST (10 min each) and then probed with the corresponding secondary antibodies conjugated with horseradish peroxidase (Abcam, UK) at room temperature for 2 h. The membranes were then washed thrice for 10 min each using TBST remove unbound secondary antibodies and then visualized using enhanced chemiluminescence (Thermo Scientific Inc., USA). The densities of specific bands were measured using ImageJ software’s densitometry feature and normalized against a loading control (GAPDH).

### Statistical analysis

All data are expressed as the mean ± standard error of the mean (SEM). The data were analyzed to one-way or two-way ANOVA, as appropriate, and then followed by a Bonferroni or Tukey post hoc test for statistically significant results. A *p* < 0.05 was considered to be statistically significant.

## Results

### AR is upregulated in BPRA mice

**Fig. 1:**
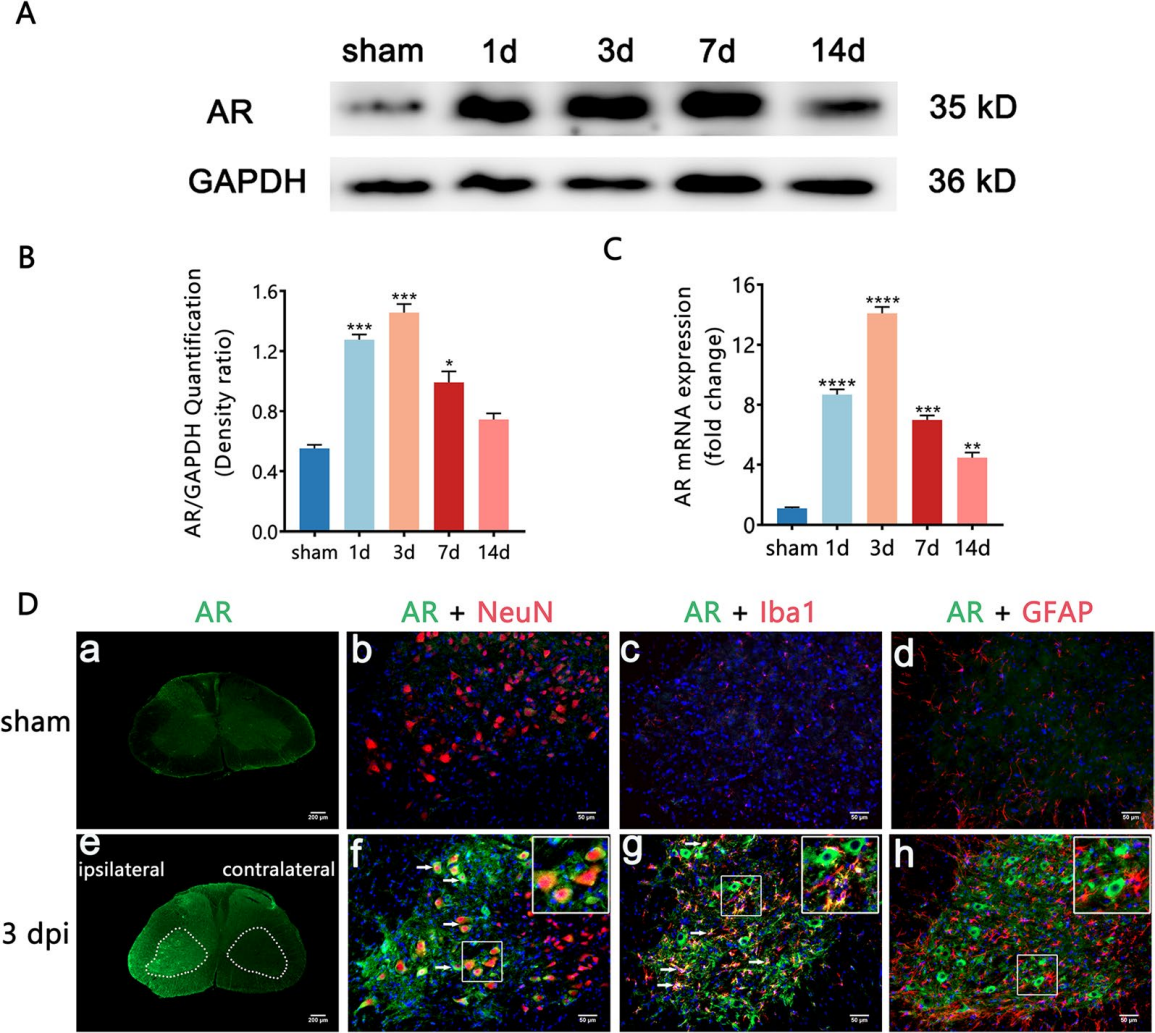
BPRA mouse spinal cord showed increased AR expression. **A**, **B** Spinal cord AR protein expression normalized against a loading control (GAPDH) and **C** mRNA expression following BPRA injury. **D** Representative images showing elevated spinal AR levels indicated by immunofluorescence staining of neurons (NeuN), microglia (Iba1) and astrocytes (GFAP). The dashed lines in the ventral horn mark areas where MNs are situated. The white arrows indicate colocalization of AR (green color) with NeuN-, IBA1- or GFAP (red color)-positive cells. All data are presented as the mean ± SEM, *n* = 5 mice/group. *p < 0.01, **p < 0.01, ***p < 0.001, ****p < 0.0001 vs. sham, scale bar = 200 µm (**a**, **e**), scale bar = 50 µm (**b**–**d**, **f**–**h**)

Generally, AR is hardly expressed in the normal tissue. However, there are upregulations of AR expression following stroke, non-alcoholic fatty liver disease, diabetic retinopathy as well as diabetic peripheral neuropathy [16, 19–21]. In our study, the expression of AR in the spinal cord of BPRA mice was compared with sham mice. AR was upregulated approximately 13-fold at the mRNA level and threefold at the protein level at 3 dpi (day post-injury) compared with the sham group (Fig. 1A–C). Immunofluorescence staining of spinal cord tissues also showed that the BPRA mice exhibit stronger AR expression than the sham mice at 3 dpi, and AR localization was predominantly cytoplasmic in neurons and microglia on the ipsilateral ventral horn of the spinal cord (Fig. 1D).

### Genetic ablation of AR in mice prevented BPRA-mediated MNs death

**Fig. 2:**
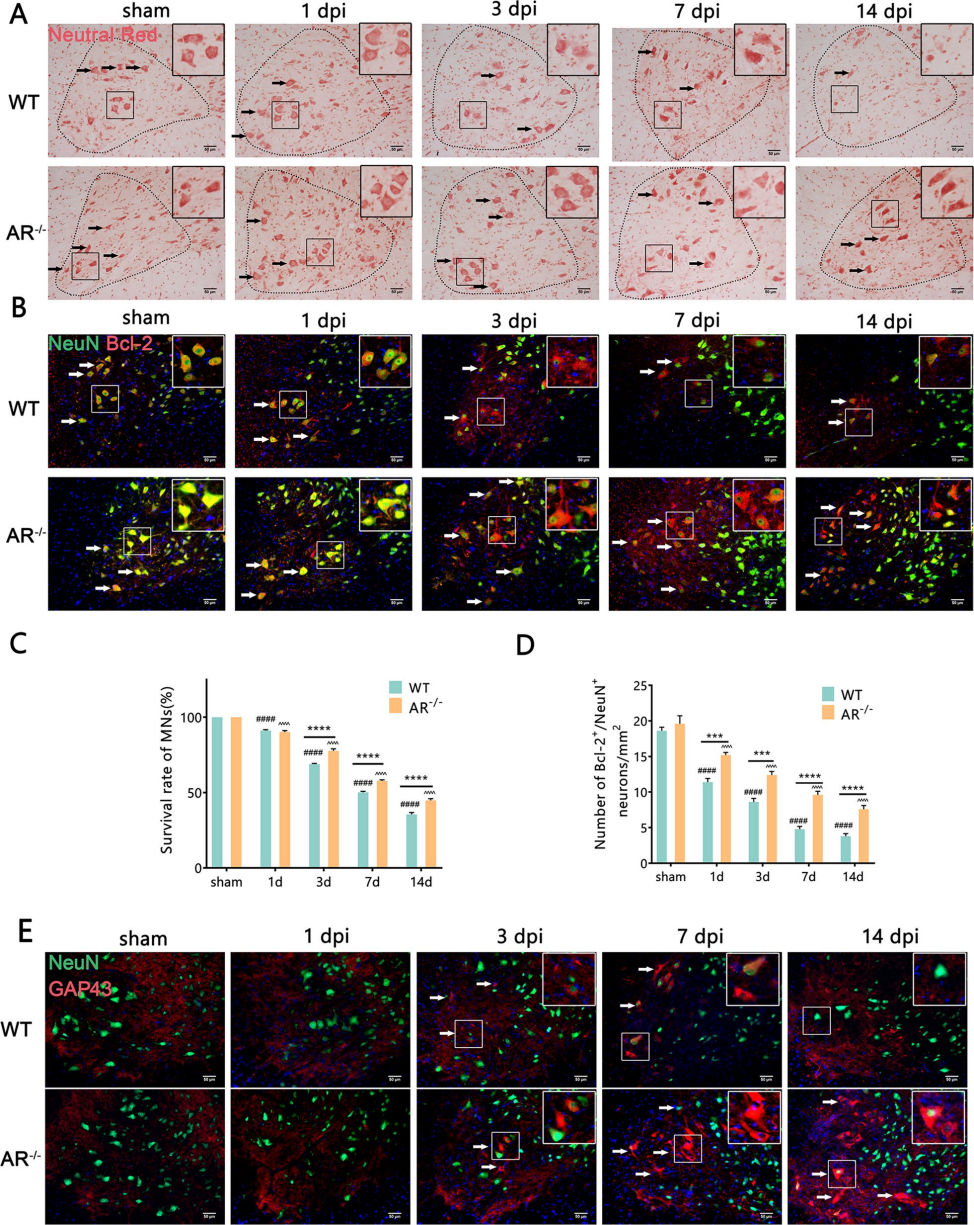

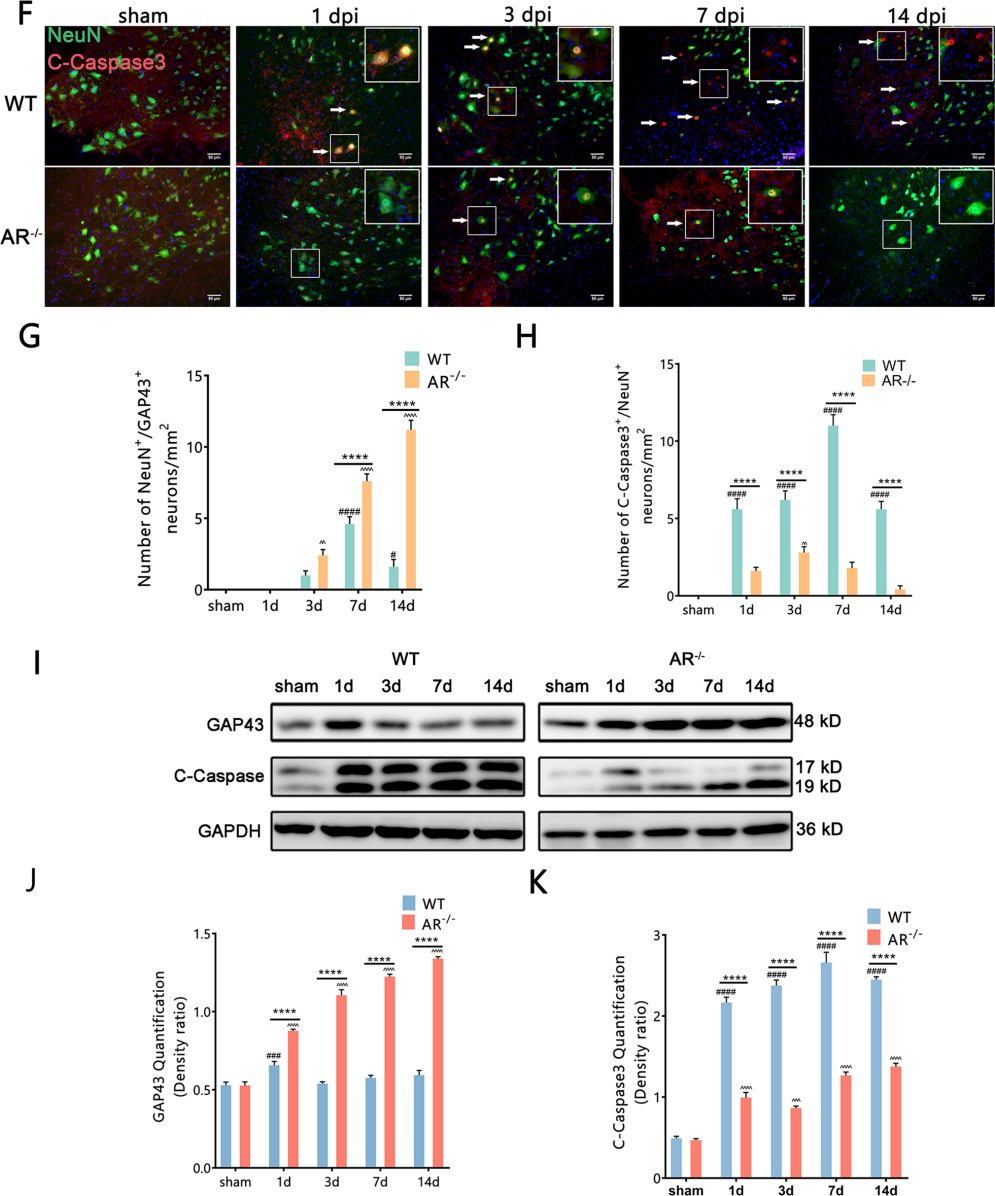
Downregulation of apoptosis-related protein and upregulation of MNs survival rate in AR^−/−^ mice after BPRA injury. **A** Spinal cords were counterstained with neutral red at 1–14 dpi to evaluate the MN survival rate. **B** Representative images showing Bcl-2 and NeuN staining at 1–14 dpi. **C**, **D** Summary of number changes in the ipsilateral spinal MN survival rate and Bcl-2 staining with quantification of NeuN-positive neurons between AR^−/−^ and WT mice. **E** Representative images of MNs regeneration indicated by GAP43 staining with quantification of NeuN-positive neurons. **F** Representative images of MNs apoptosis in mice as measured by C-Caspase3 double staining with quantification of NeuN-positive neurons. **G**, **H** Summary of number changes in ipsilateral spinal C-Caspase3, GAP43 and NeuN colocalization-positive neurons between AR^−/−^ and WT mice. **I**–**K** Representative images of spinal cord C-Caspase3 and GAP43 protein expression in AR^−/−^ and WT mice normalized against a loading control (GAPDH). All data were analyzed by two-way ANOVA and are presented as the mean ± SEM, *n* = 5 mice/group, ^####^*p* < 0.0001, WT vs. sham group, ^^^^^^*p* < 0.0001, AR^−/−^ vs. sham group, ****p < 0.0001, WT vs. sham group. Scale bar = 50 μm (**A**, **B**, **E**, **F**)

To investigate the effects of AR upregulation in BPRA, we sought to find out whether the deficiency of AR (examined using AR knockout mice, AR^−/−^) alleviated MNs death in the model of BPRA mice. Initially, we examined the effect of AR deficiency on MNs survival by staining neutral red, and the survival rate of injured MNs was assessed as the ratio of ipsilateral ventral horn neutral red-positive MNs to those on the contralateral ventral horn [22]. As shown in Fig. 2A and C, the survival ratio of MNs in AR^−/−^ group was higher than WT group at 3, 7 and 14 dpi on the ipsilateral ventral horn of the spinal cord. Furthermore, AR^−/−^ mice exhibited an increase in NeuN and anti-apoptosis gene Bcl-2 colocalization-positive neurons on the ipsilateral ventral horn of the spinal cord at 1–14 dpi (Fig. 2B, D). In addition, compared with the WT group, the AR^−/−^ group showed more regeneration-related protein GAP43/NeuN colocalization-positive neurons at 7–14 dpi and higher GAP43 protein expression at 1–14 dpi on the ipsilateral ventral horn of the spinal cord (Fig. 2E, G, I, J). BPRA resulted in an increase in the apoptosis-related protein C-Caspase3/NeuN colocalization-positive neurons and C-Caspase3 protein expression level, which was attenuated in AR^−/−^ mice at 1–14 dpi on the ipsilateral side of the spinal cord (Fig. 2F, H, I, K). Collectively, the results provided experimental evidence that genetic ablation of AR in mice prevented BPRA-mediated MNs death.

### BPRA-mediated MNs death was attenuated by enhancing autophagy level in AR^−/−^ mice

**Fig. 3:**
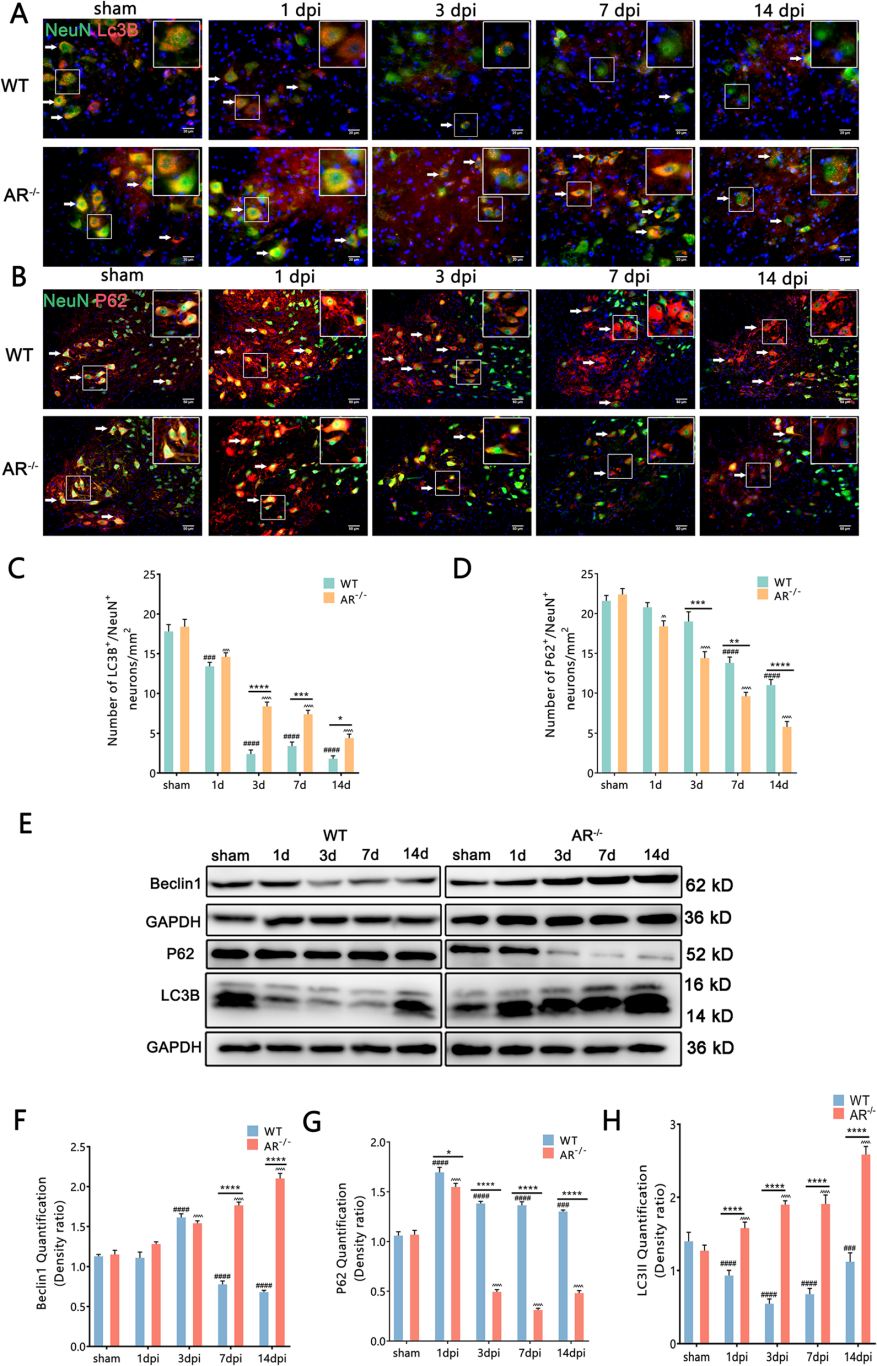
Protection against BPRA-mediated motor injury in AR^−/−^ mice occurs by enhancing autophagy. **A**, **B** Representative images of autophagy hallmarks in mice as measured by LC3B and P62 double staining with NeuN. **C**, **D** Number changes in ipsilateral spinal LC3B/NeuN colocalization-positive neurons and P62/NeuN colocalization-positive neurons between AR^−/−^ and WT mice. **E**–**H** Representative images of spinal cord Beclin1, P62 and LC3B protein expression normalized against a loading control (GAPDH) between AR^−/−^ and WT mice. Data were analyzed by two-way ANOVA and are presented as the mean ± SEM, *n* = 5 mice/group, ^####^*p* < 0.0001, WT vs. sham group, ^^^^^^*p* < 0.0001, AR^−/−^ vs. sham group, ****p < 0.0001, WT vs. sham group. Scale bar = 50 μm (**B**) and 20 μm (**A**)

The dysregulation of autophagy is involved in motor neuron diseases, including spinal cord injury and amyotrophic lateral sclerosis. To evaluate the autophagy level after BPRA, we assessed the expression of the autophagy protein, LC3B in the injured spinal cord by immunofluorescence and western blotting. The conversion of LC3B-I to LC3B-II by adding phosphatidylethanolamine is important to autophagosome formation and this is considered as a marker of autophagosome formation and accumulation. Compared with BPRA WT mice, AR^−/−^ mice had greatly increased LC3B/NeuN colocalization-positive neurons at 3–14 dpi on the ipsilateral ventral horn of the spinal cord and higher LC3B-II protein expression at 1–14 dpi on the ipsilateral side of the spinal cord (Fig. 3A, C, E, H), indicating the more accumulation of autophagosomes after injury in AR^−/−^ mice.

To elucidate the autophagy mechanisms following BPRA, we assessed the levels of proteins that would regulate and form autophagosomes. Increased accumulation of LC3-II is likely due to either increased formation or decreased degradation of autophagosomes. Ubiquitinated cargo is delivered to autophagosomes by the adapter protein P62 (SQSTM1). During this delivery, the adapter protein (p62) is also degraded by autophagy alongside its cargo. Therefore, the accumulation of p62 suggests that autophagic degradation has been disrupted. Compared with BPRA WT mice, AR^−/−^ mice had greatly decreased P62/NeuN colocalization-positive neurons at 3–14 dpi on the ipsilateral ventral horn of the spinal cord and lower P62 protein expression at 1–14 dpi on the ipsilateral side of the spinal cord (Fig. 3B, D, E, G). Moreover, western blot analysis showed more expression of the autophagy regulatory protein Beclin1 in AR^−/−^ mice on the ipsilateral side of the spinal cord at 7 and 14 dpi than in WT mice, suggesting that the genetic ablation of AR increased initiation of autophagy (Fig. 3E, F). Overall, these results suggested BPRA-mediated MNs death was suppressed in AR^−/−^ mice, which may be associated with enhanced autophagy level.

### Autophagic vacuoles and mitochondria number were improved in a BPRA model of AR^−/−^ mice

**Fig. 4:**
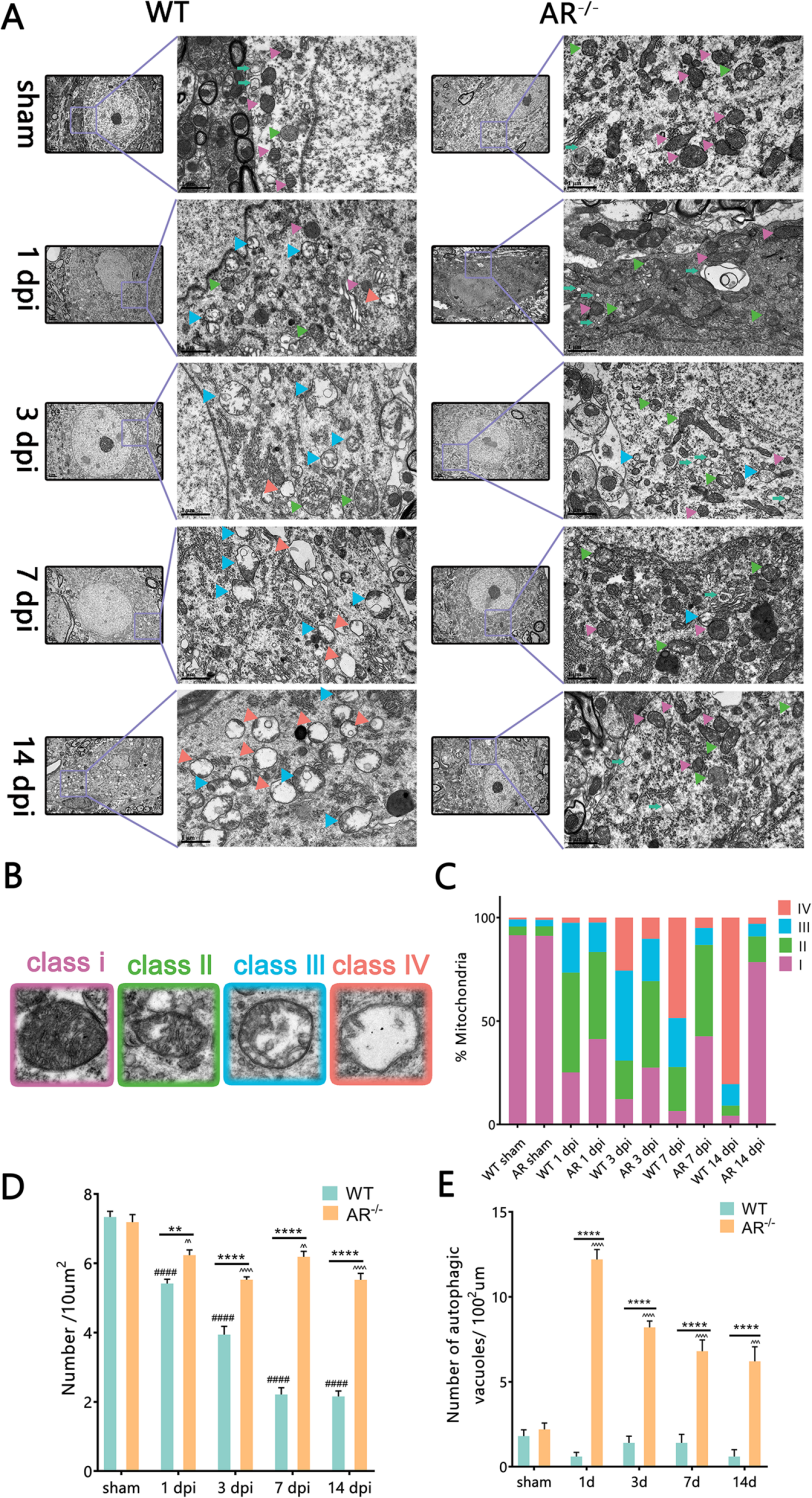
MNs display altered mitochondrial structure between AR^−/−^ and WT mice at 1–14 d after BPRA injury. **A** Electron microscopy images of the ultrastructure of MNs in AR^−/−^ and WT mice at 1–14 d after BPRA injury. Colored triangle indicate mitochondria, green arrows indicate autophagic vacuoles. Representative images of mitochondrial classes I (purple triangle), II (green triangle), III (blue triangle), and IV (orange triangle) (**B**) and their quantitative distributions (**C**) in WT and AR^−/−^ mice. Quantitative graphs of the means ± SEM of mitochondria number/10 µm^2^ (**D**) and autophagic vacuoles number (**E**). Quantification was performed in at least 20 different fields (> 150 mitochondria) per mouse, *n* = 5 mice/group. ^####^*p* < 0.0001, WT vs. sham group, ^^^^^^*p* < 0.0001, AR^−/−^ vs. sham group, ****p < 0.0001, WT vs. sham group

We further used electron microscopy (EM) to evaluate autophagic vacuoles number of MNs in both WT and AR^−/−^ mice following BPRA. The structures of typical autophagic vacuoles are shown under high-magnification (13,500 ×) EM (green arrow in Fig. 4A). Compared with WT mice, the number of autophagic vacuoles was remarkably increased at 1–14 dpi in AR^−/−^ mice (Fig. 4E). These results provided definite evidence that autophagy initiation was induced in AR^−/−^ mice following BPRA injury.

Mitochondria accommodate most cell energetic demands by generating ATP. Moderate autophagy is responsible for degrading damaged mitochondria caused by BPRA injury. We analyzed mitochondrial structures and number in the BPRA model at 1–14 dpi in WT and AR^−/−^ mice. We first examined mitochondrial morphology and size by transmission electron microscopy and observed that there were larger mitochondria with altered crista organization in MNs from WT mice when comparing with those in AR^−/−^ mice following BPRA (colored arrowheads in Fig. 4A). We further analyzed mitochondrial alterations in depth and classified mitochondrial morphology into four categories namely [23]: class I: fairly dark mitochondria, with a uniform matrix filled with densely packed and regularly distributed cristae; class II: mitochondria with disrupted cristae and a loss of matrix density; class III: empty mitochondria with disorganized cristae or cristae on the periphery; and class IV: swollen mitochondria with disrupted membranes) (Fig. 4B). Quantifications of mitochondrial subclasses were then performed, revealing that the WT mice at 14 dpi exhibited 4.2% mitochondrial class I, 4.9% class II, 10.4% class III and 80.5% class IV (Fig. 4C). However, the AR^−/−^ mice at 14 dpi displayed a drastic increase in “healthy” mitochondria class I (78.5%) and class II (12.4%), class III (6.1%), class IV (3%) (Fig. 4C). We also observed that AR^−/−^ mice also displayed a significant increase in the mitochondrial number at 1–14 dpi (Fig. 4D) compared with the WT mice following BPRA injury. Taken together, these findings demonstrated that genetic ablation of AR could eliminate damaged mitochondria and enhance autophagy level in BPRA mice mode.

### Genetic ablation of AR in mice prevented the BPRA-mediated decrease in SIRT1–AMPK–mTOR signaling

**Fig. 5:**
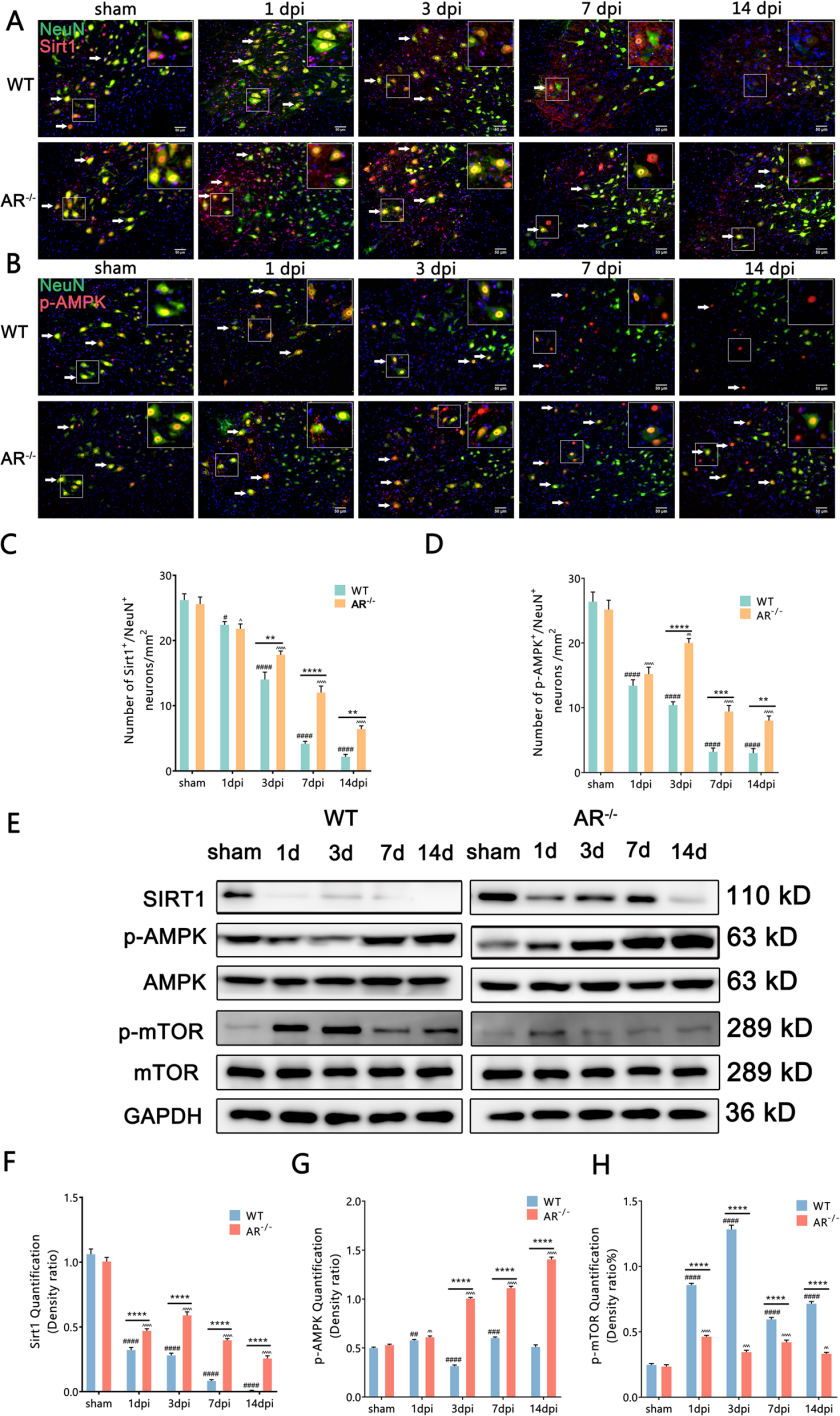
AR^−/−^ mice show enhanced autophagy level by activating SIRT1–AMPK–mTOR signaling. **A**, **B** Representative images of SIRT1 and p-AMPK IF staining with NeuN-positive neurons. **C**, **D** Number changes in ipsilateral spinal SIRT1, p-AMPK and NeuN colocalization-positive neurons between AR^−/−^ and WT mice. **E**–**H** Representative images of spinal cord SIRT1, p-AMPK and p-mTOR protein expression normalized against a loading control (GAPDH) in AR^−/−^ and WT mice. Data were analyzed by two-way ANOVA and are presented as the mean ± SEM, *n* = 5 mice/group. ^####^*p* < 0.0001, WT vs. sham group, ^^^^^^*p* < 0.0001, AR^−/−^ vs. sham group, ****p < 0.0001, WT vs. sham group. Scale bar = 50 μm (**A**, **B**)

Elevated AR activity is known to deplete cellular NADPH and cause high cytosolic NADH/NAD^+^ ratio. This results to loss of NAD^+^-dependent deacetylase SIRT1 activity [12]. Compared with WT mice, AR^−/−^ mice had greatly increased SIRT1/NeuN colocalization-positive neurons at 3–14 dpi on the ipsilateral ventral horn of the spinal cord and higher SIRT1 protein expression at 1–14 dpi on the ipsilateral side of the spinal cord (Fig. 5A, C, E, F). Our results indicate that BPRA decreased the expression of SIRT1 and that AR^−/−^ mice significantly restored the BPRA-induced decrease in SIRT1 expression.

A rising number of studies suggest that aberrant mTOR signaling impacts many pathways, such as glucose metabolism, energy production, mitochondrial function, cell growth and autophagy. AMPK and mTOR have a reciprocal relationship mediated by SIRT1, and the substrates of mTORC1 suppress autophagy [24]. We further measured the effect of AR ablation on BPRA-induced expression of AMPK and mTOR in motor neurons. Compared with WT mice, AR^−/−^ mice had greatly increased p-AMPK/NeuN colocalization-positive neurons on the ipsilateral ventral horn of the spinal cord and higher p-AMPK protein expression at 3–14 dpi on the ipsilateral side of the spinal cord (Fig. 5B, D, E, G). Similarly, the BPRA-induced mTOR phosphorylation increase was also prevented in AR^−/−^ mice at 1–14 dpi on the ipsilateral side of the spinal cord (Fig. 5H). It may infer that genetic ablation of AR potentially enhance SIRT1–AMPK–mTOR signaling and autophagy level following BPRA injury.

### Protection against BPRA injury via attenuation of neuroinflammation and promoting microglia to switch from a pro-inflammatory to anti-inflammatory phenotype in AR^−/−^ mice

**Fig. 6:**
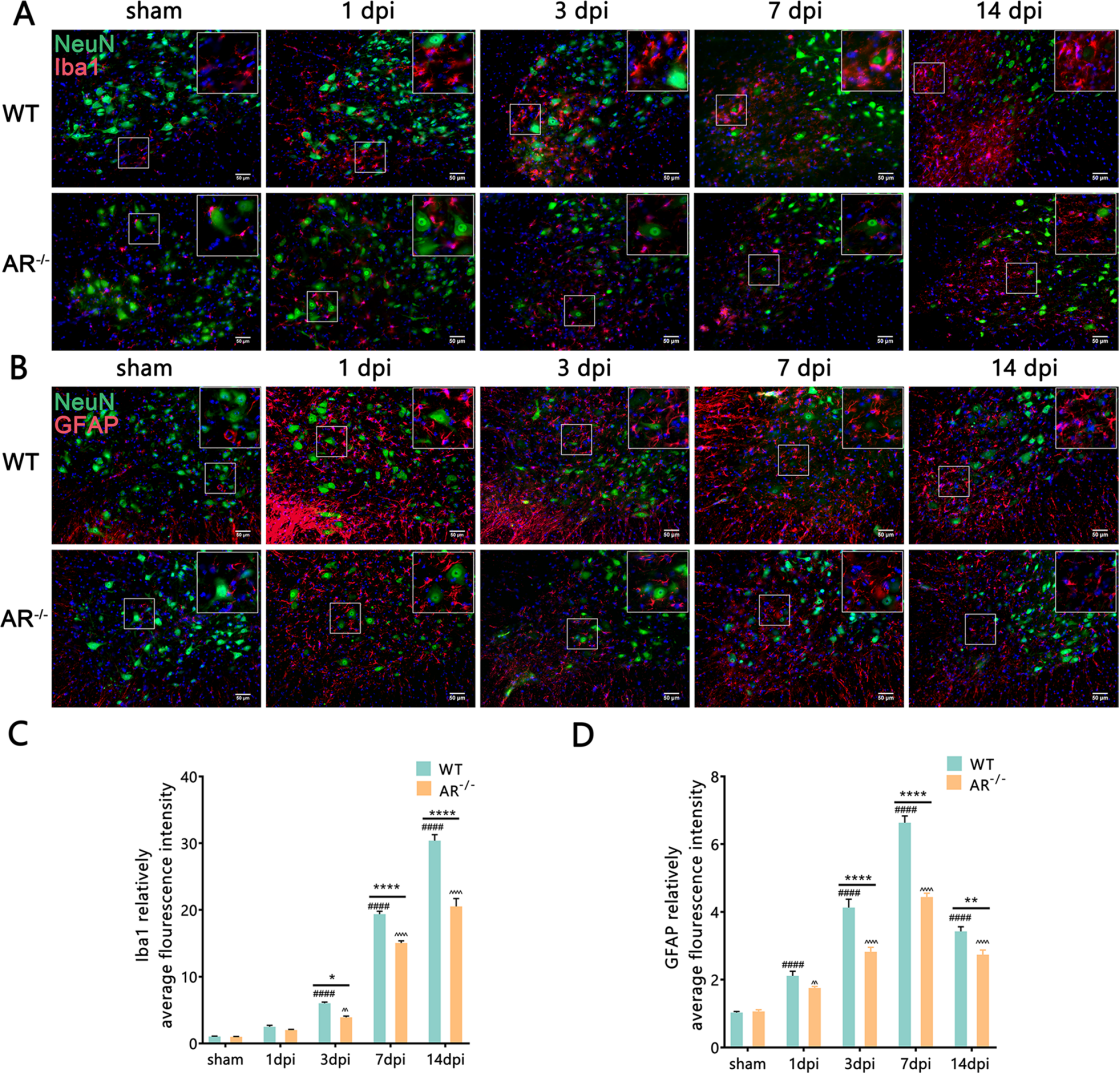
Attenuation of BPRA injury-induced neuroinflammation in the injured spinal cord in AR^−/−^ mice. **A**, **B** Representative images of Iba1 and GFAP IF staining with NeuN. **C**, **D** Summary of changes in ipsilateral spinal IBA1 and GFAP average fluorescence intensity between AR^−/−^ and WT mice. Data were analyzed by two-way ANOVA and are presented as the mean ± SEM, *n* = 5 mice/group, ^####^*p* < 0.0001, WT vs. sham group, ^^^^^^*p* < 0.0001, AR^−/−^ vs. sham group, ****p < 0.0001, WT vs. sham group. Scale bar = 50 μm (**A**, **B**)

**Fig. 7:**
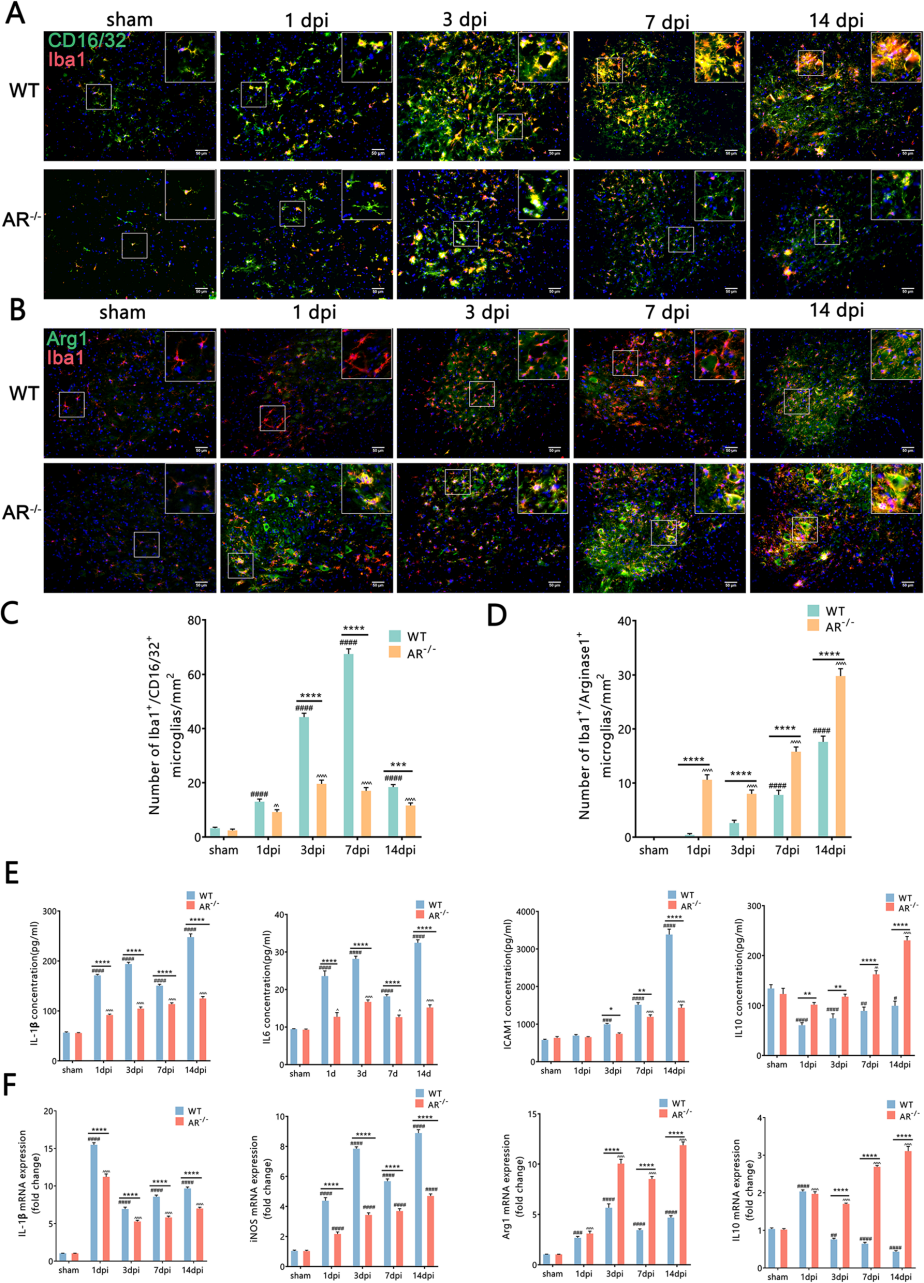
Protection against BPRA injury by switching microglia from a pro-inflammatory to anti-inflammatory phenotype in AR-/- mice. **A**, **B** Representative images of CD16/32 and Arginase 1 IF staining with quantification of Iba1-positive microglia. **C**, **D** Summary of number changes in ipsilateral spinal CD16/32^+^ and Arginase1^+^ cells colocalization-positive to microglia between AR^−/−^ and WT mice. **E** Summary of changes in ipsilateral spinal IL-1β, IL-6, ICAM1, and IL-10 levels between AR^−/−^ and WT mice. **F** Summary of changes in ipsilateral spinal IL-1β, IL-6, Arginase 1, and IL-10 mRNA levels between AR^−/−^ and WT mice. Data were analyzed by two-way ANOVA and are presented as the mean ± SEM, *n* = 5 mice/group, ^####^*p* < 0.0001, WT vs. sham group, ^^^^^^*p* < 0.0001, AR^−/−^ vs. sham group, ****p < 0.0001, WT vs. sham group. Scale bar = 50 μm (**A**, **B**)

Following BPRA injury, the immunoreactive expression of Iba1 and GFAP, as markers of microglia and astrocytes, respectively, was used to assess neuroinflammation. Compared with the WT mice, the AR^−/−^ mice had dramatically decreased Iba1 and GFAP average fluorescence intensity at 3–14 dpi on the ipsilateral ventral horn of the spinal cord (Fig. 6A–D). This evidence indicates that genetic ablation of AR attenuated ventral horn neuroinflammation in the injured spinal segment. Activated microglia can either become pro-inflammatory or anti-inflammatory phenotypes [25]. Compared with the WT group, the AR^−/−^ group showed a dramatically decreased proportions of pro-inflammatory (CD16/32^+^/Iba-1^+^) microglia at 3–14 dpi (Fig. 7A, C) and an increased proportions of anti-inflammatory (Arginase1^+^/Iba-1^+^) microglia at 1–14 dpi on the ipsilateral ventral horn of the spinal cord (Fig. 7B, D). We further determined whether the AR-deficient mice also showed aberrant expression of cytokines that are functionally more important. Compared with WT mice, AR^−/−^ mice had significantly reduced pro-inflammatory cytokines IL-1β and IL-6 levels at 1–14 dpi and inflammatory responses protein ICAM1 levels at 3–14 dpi, while increasing anti-inflammatory cytokine IL-10 levels at 1–14 dpi (Fig. 7E) on the ipsilateral side of the injured spinal cord. Moreover, compared with WT mice, AR^−/−^ mice had significantly reduced pro-inflammatory cytokines IL-1β mRNA levels at 1–14 dpi and iNOS mRNA levels at 1–14 dpi, while increasing anti-inflammatory cytokines IL-10 mRNA levels at 3–14 dpi and Arg-1 mRNA levels at 3–14 dpi (Fig. 7F) on the ipsilateral side of the injured spinal cord. These results further indicate that genetic ablation of AR promotes microglia to switch from a pro-inflammatory to anti-inflammatory phenotype following BPRA injury.

### Genetic ablation of AR acts via 4-HNE–p-CREB signaling to exert its anti-inflammatory effects

**Fig. 8:**
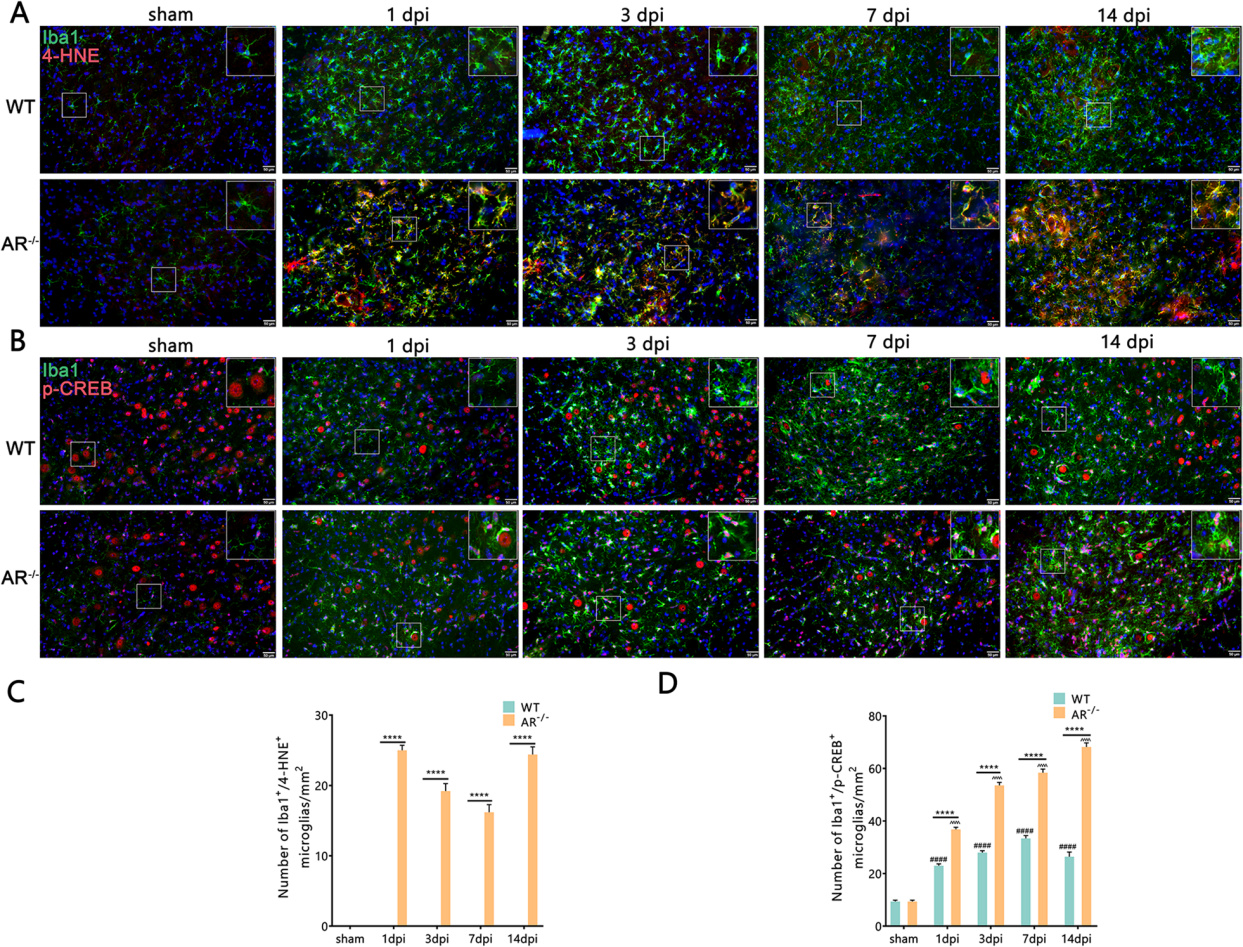
Upregulation of 4-HNE-p-CREB in AR^−/−^ mice after BPRA injury. **A**, **B** Representative images of 4-HNE and p-CREB IF staining with microglia. **C**, **D** Number change in ipsilateral 4-HNE^+^ and p-CREB^+^ cells colocalization-positive to microglia between AR^−/−^ and WT mice. Data were analyzed by two-way ANOVA and are presented as the mean ± SEM, n = 5 mice/group, ^####^*p* < 0.0001, WT vs. sham group, ^^^^^^*p* < 0.0001, AR^−/−^ vs. sham group, ****p < 0.0001, WT vs. sham group

Considering cyclic-AMP response binding protein (CREB) plays a key role in anti-inflammatory microglial phenotypes and AR can reduce 4-HNE into GS-DHN, a macrophage inflammatory mediator, we estimated whether genetic ablation of AR acts via 4-HNE–p-CREB signaling to exert its “phenotype switching” effect following BPRA injury [26, 27]. Therefore, we sought to find out the expression of 4-HNE–p-CREB signal axis following BPRA in WT and AR^−/−^ mice. Compared with the WT mice, the AR^−/−^ mice had dramatically increased number of 4-HNE^+^/Iba1^+^ microglia at 1–14 dpi and number of p-CREB^+^/Iba1^+^ microglia at 1–14 dpi on the ipsilateral ventral horn of the spinal cord after BPRA injury (Fig. 8A–D). These results suggest that genetic ablation of AR exerts its anti-inflammatory effects via the 4-HNE–p-CREB signaling pathway.

### Testing the efficacy of pharmacological inhibition of AR in attenuating BPRA-induced autophagy disruption, neuroinflammation and MNs death in mice

**Fig. 9:**
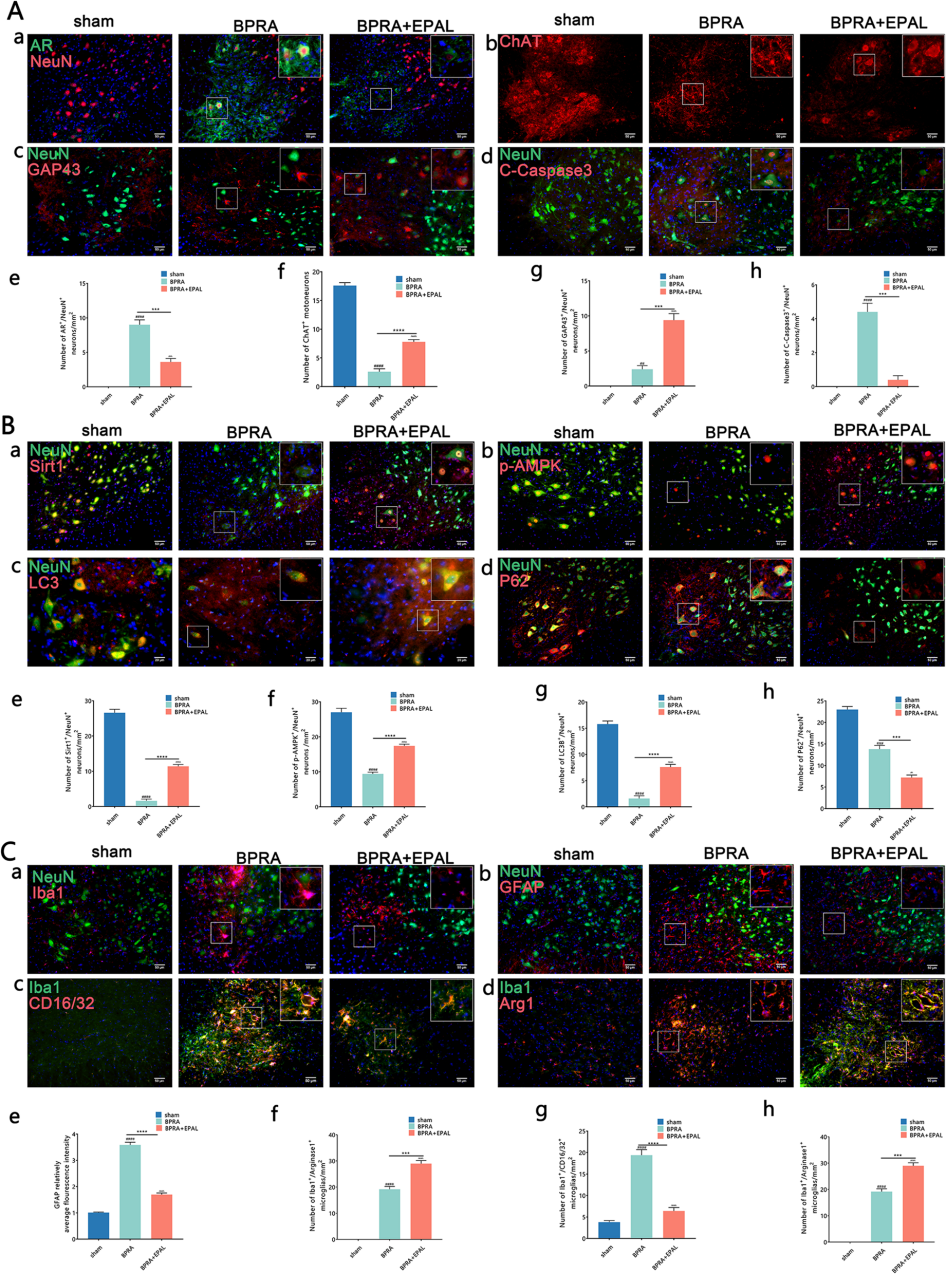
Testing the efficacy of pharmacological inhibition of AR in attenuating BPRA-induced MNs death in mice. **A** (**a**–**d**) Representative IF staining images of AR, ChAT, GAP43, and C-Caspase3. (**e**–**h**) Number changes in ipsilateral ChAT^+^ MNs and AR^+^/NeuN^+^, GAP43^+^/NeuN^+^ and C-Caspase3^+^/NeuN^+^ colocalization-positive neurons between the BPRA and BPRA + EPAL groups in WT mice. **B** (**a**–**d**) Representative images of SIRT1, p-AMPK, LC3B and P62 IF staining with NeuN. (**e**–**h**) Number changes in SIRT1^+^/NeuN^+^, p-AMPK^+^/NeuN^+^, LC3B^+^/NeuN^+^ and P62^+^/NeuN^+^ colocalization-positive neurons between the BPRA and BPRA + EPAL groups in WT mice. **C** (**a**–**d**) Representative images of Iba1 and GFAP IF staining with NeuN and CD16/32 and Arg1 IF staining with Iba1. (**e**–**h**) Changes in Iba1 and GFAP average fluorescence intensity and number changes CD16/32^+^ and Arg1^+^ cells colocalization to Iba-1^+^ cells between the BPRA and BPRA + EPAL groups in WT mice. All data were analyzed by two-way ANOVA and are presented as the mean ± SEM, *n* = 5 mice/group, ^####^*p* < 0.0001, BPRA vs. sham group, ^^^^^^*p* < 0.0001, BPRA + EPAL vs. sham + EPAL group, ****p < 0.0001, BPRA + EPAL vs. BPRA group. Scale bar = 50 μm

**Fig. 10:**
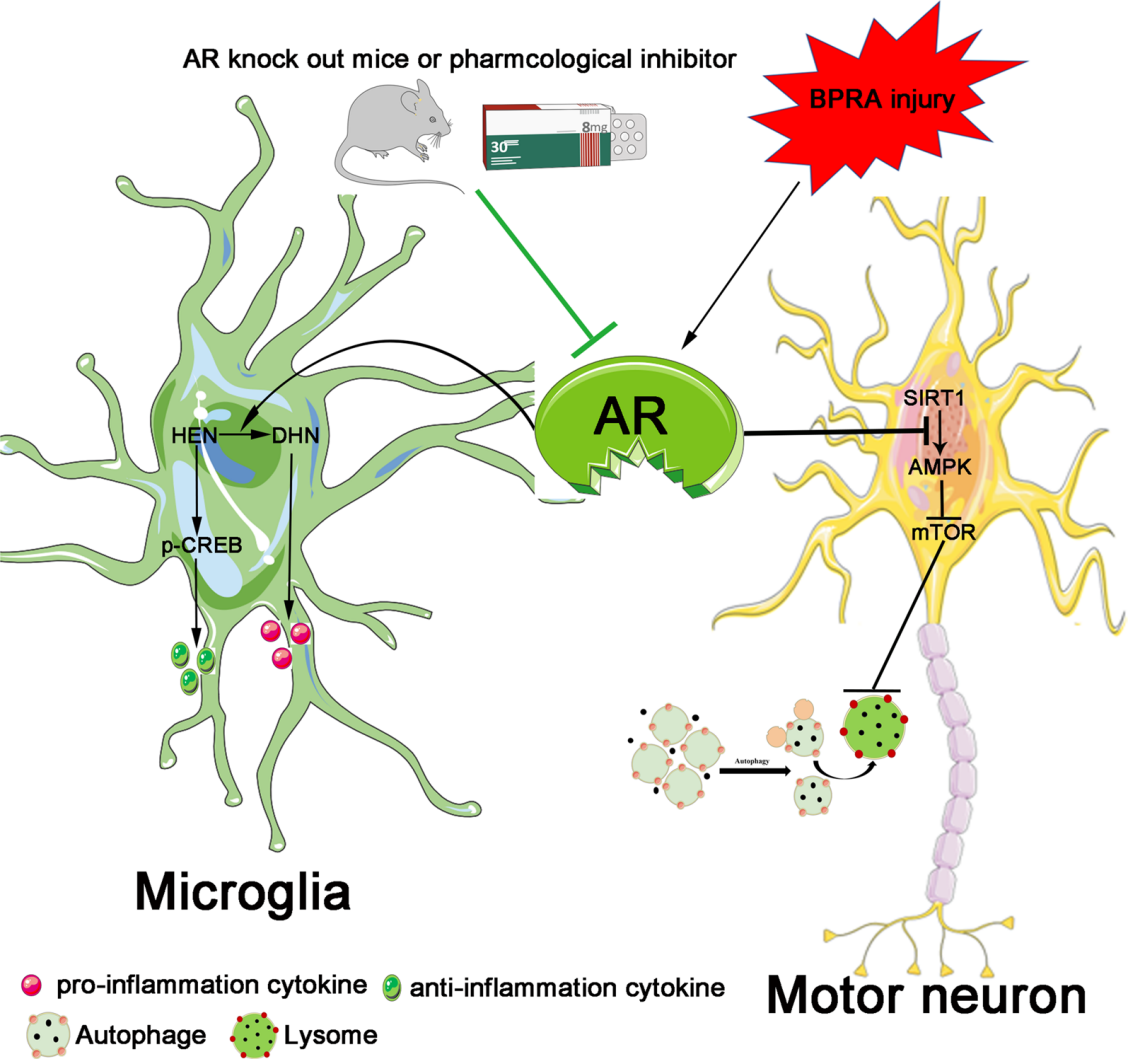
Schematic representation of the mechanistic role of AR in BPRA injury

Epalrestat (EPAL), a pharmacological AR inhibitor with protective effects against diabetic complications in mouse models and human clinical trials [16], was used to evaluate the neuroprotective potential of AR suppression following BPRA injury. Significantly reduced AR, C-Caspase3 expression and increased ChAT, GAP43 expression on the ipsilateral ventral horn of the spinal cord, as evaluated using IF, were observed with pharmacological inhibition of AR by epalrestat at 14 dpi following BPRA injury (Fig. 9A). Furthermore, the administration of epalrestat significantly reduced the expression of P62, but greatly upregulated LC3B, SIRT1, p-AMPK expression on the ipsilateral ventral horn of the spinal cord at 14 dpi following BPRA injury (Fig. 9B). In addition, BPRA mice significantly reduced the expression of Iba1, GFAP and CD16/32 while greatly upregulating Arginase 1 expression on the ipsilateral ventral horn of the spinal cord at 14 dpi after treatment with epalrestat (Fig. 9C). Therefore, these results were similar to those of genetic knockout, suggesting that with use of epalrestat, pharmacological suppression of AR showed significant neuroprotective of MNs effects by attenuating BPRA-induced autophagy disruption and neuroinflammation (Fig. 10).

## Discussion

BPRA injury is mainly caused by motor vehicle accident and birth trauma. Severe BPRA can lead to partial or complete loss of motor and sensory functions of the upper limb because of massive MNs death, motor axon degeneration and de-innervation of targeted muscles. The novel finding of our study is that AR expression is markedly upregulated in the BPRA mice model. Importantly, the role of AR following BPRA is investigated by using AR gene knockout mice and inhibiting its activity pharmacologically with AR inhibitor epalrestat. Our date reveals that genetic knockout or epalrestat pharmacological suppression of AR contributed to better MNs survival by attenuating BPRA-induced autophagy disruption and neuroinflammation. These findings cast AR as a potential therapeutic target in BPRA injury treatments and that AR inhibitors could be a promising strategy for mitigating against BPRA caused MNs death.

Autophagy is a lysosome-dependent intracellular degradation process that eliminates long-lived proteins as well as damaged organelles. An increasing number of evidence suggests that dysregulation of autophagy plays a pivotal role in the progression of neurodegenerative diseases including motor neuron disorders. Our data suggest that BPRA will cause MNs autophagy dysregulation and MNs death. Moreover, heightened levels of P62 and reduced levels of LC3II found in BPRA mice model were associated with MNs death. Notably, autophagy could prevent the accumulation of damaged macromolecules and organelles to maintain function and survival of MNs. Importantly, in AR-deficient mice (AR-/- or EPAL treatment), the ratio of LC3BII to LC3BI was significantly increased, while the level of P62 was significantly decreased, suggesting that AR suppression may enhance autophagy level and maintain MNs survival. In an earlier study, SIRT1 could protected cardiomyocytes against hypoxia-induced apoptotic death in a mechanism via autophagy and AMPK activation. AMPK, a serine/threonine kinase, senses changes in, and regulates cellular energy homeostasis. When activated, it promotes autophagy by inhibiting mTOR [28]. Both AMPK and SIRT1 are fuel-sensing molecules regulate each other. The AMPK activation are regulated by two upstream kinases, LKB1 (the primary AMPK kinase) and CaMKKβ (an AMPK kinase). Regulation of SIRT1 has been attributed to changes in NAD^+^ abundance and the NAD^+^/NADH ratio. The actual relationship between AMPK and SIRT1 need us examine the LKB1 activity and NAD^+^ abundance in the future study. At present, our findings implied that suppression of AR could enhance SIRT1–AMPK–mTOR signaling and autophagy level following BPRA injury.

Our IHC studies come from ipsilateral ventral horn only, while WB studies were used to analyze the protein expression levels in all the cells (neurons, microglia, astrocytes and oligodendrocytes) from ventral horn and dorsal horn on the ipsilateral side of spinal cord. Although our results showed elevated autophagy level in AR^−/−^ mice after BPRA injury, the exact mechanism of autophagy, its regions and cell-type specificity remained unknown. In a previous spinal cord injury study [29], the expressions of LC3 vary widely between different cell types. There would be high accumulation of LC3 in ventral horn motor neurons preferentially, which was similar in most of activated CD11b^+^ microglia according their cell morphology. In the white matter, LC3 accumulated in CC1-positive oligodendrocytes both in dorsal and ventral white area. However, unlike microglia and oligodendrocytes, the number of LC3-positive astrocytes remained very low in all regions at all time points examined. According to our previous and present study [9], BPRA injury causes microglia proliferation and activation in the injured spinal ventral horn and dorsal horn, with the peaks at 14 day post BPRA injury, which may be partly relevant with our western blot result. However, to understand the effect of AR on cell-type specificity autophagy function, further studies with cell-specific AR knockout mice are necessary.

Inflammation is among the very crucial pathological processes in the secondary injury phase after BPRA injury. Resident microglia and hematogenous macrophages, invade the injury site shortly after the primary injury [30]. The inflammatory reactions that occur after spinal cord injury is a “double-edged sword”, which can cause both neuroprotective and neurotoxic effects depending on the dichotomous polarity of microglia/macrophages [31]. Cellular injury causes the release of pro-inflammatory cytokines and other mediators. Cytokine production and iNOS activation typifies the pro-inflammatory phenotypes macrophage and microglial in earlier phases of the spinal cord injury [32]. The anti-inflammatory phenotypes existence following spinal cord injuries have not been widely reported. In a contusion model of spinal cord injury in mice, the major type of microglia/macrophages are pro-inflammatory phenotype while the anti-inflammatory phenotype is transient and a minority. The overexpression of one of the classic markers of the anti-inflammatory phenotype, Arg1, appears to be transient and returns to normal expression levels by day 14 post-injury [32]. Our results also showed more pro-inflammatory phenotype microglia/macrophages in the WT mice following BPRA injury. However, the anti-inflammatory phenotype microglia/macrophages were predominant within the injured spinal cord of AR^−/−^ mice. These findings demonstrate that AR plays an important role in the divergence of microglial/macrophage functions after BPRA injury in mice.

Recently, several reports revealed that AR mediates LPS-induced inflammatory signals in macrophages [33]. Suppression of AR by several pharmacological inhibitors including sorbinil, tolrestat, and zopolrestat, attenuates LPS-induced production of TNF-α, IL-6, IL-1β, and IFN-γ and MCP-1 in murine peritoneal macrophages [34]. Results from these studies further showed that AR suppression or ablation averts macrophage transformation into the pro-inflammatory phenotype. In this study, we found that AR deficiency or suppression reduced the number of pro-inflammatory phenotype microglia/macrophages following BPRA. The reduction of 4-HNE to DHN, happens efficiently because of their much lower K_m_ values (in the micromolar range of 10 to 30 μM) compared with that of glucose (50 to 100 mM) [35]. Therefore, suppression of AR averts macrophages polarization to the pro-inflammatory phenotype. This is possibly due to the reduced conversion of 4-HNE to DHN. Furthermore, inhibiting AR expression can avert LPS-induced knockdown of cAMP response element modulator (CREM), phosphorylation of CREB, and DNA binding of CREB in macrophages [36]. Therefore, we suggest that AR regulates this polarization switch. Specifically, 4-HNE would be reduced to DHN if there is sufficient AR, and it would cause the inflammation cytokines release thereby favoring microglial pro-inflammatory phenotype formation. However, in AR inhibition or knockout models, 4-HNE tends to accumulate in the cytoplasm where it activates CREB to favor the anti-inflammatory microglial phenotype formation (Additional file 1, Additional file 2).

AR inhibitors have been under investigation for approximately four decades, although most of them are used in diabetic neuropathy and retinopathy [37]. Epalrestat, a post-market AR inhibitor (ARI) approved in Bangladesh, India, and China, is one of the most common ARIs for patients who suffer from diabetes mellitus [38]. Although clinical studies of epalrestat’s effects on diabetic neuropathy have been carried out, it is still controversial [39]. In this study, our data demonstrated that spinal AR upregulation and consequent elevation of neuroinflammation, aberrant autophagy caused motor neuron death after BPRA. Our study provides new insights regarding the mechanisms underlying the role of AR in BPRA pathogenesis. Moreover, our data imply that AR may be a novel therapeutic target in brachial plexus roots avulsion and provides a rationale for further testing the utility of epalrestat, for the treatment of BPRA. Exploring the roles of AR-mediated signaling pathways following BPRA and spinal cord injuries may be critical for increasing our understanding of efficacy and safety profiles of ARIs.

These findings reveal that AR is a contributor to the BPRA pathogenesis, and AR deficiency is neuroprotective in mice subjected to BPRA. In the future, the cell type-specific AR knockouts could enhance our understanding of the actual function AR in those cells or tissues following BPRA or other neurodegeneration diseases. On the basis of results, we also consider epalrestat as a feasible pharmacological agent to attenuate MNs death after BPRA. Further biomedical studies and clinical trials are required to test the safety and efficacy of such AR inhibitors in BPRA patients.

## Supplementary Information

**Additional file 1: Figure S1. **The AR−/− mice genotyping and the expression level of AR in AR−/− mice. (A) AR null allele were identified by northern blot analysis. The expected bands of ∼119 bp is observed in the wild-type mice while not seen in AR−/− mice. (B) Western blot analyze the AR expression in the AR−/− mice. (C) Immunofluorescence analyze the AR expression in the spinal cord of AR−/− mice at 3 day following BPRA injury.

**Additional file 2.** Raw data of western blot.

## Data Availability

The datasets used and/or analyzed during the current study are available from the corresponding author upon reasonable request.

## Abbreviations

BPRA: Brachial plexus root avulsion
AR: Aldose reductase
AR^−^^/^^−^: Aldose reductase knockout
NADPH: Reduced form of nicotinamide adenine dinucleotide phosphate
4-HNE: 4-Hydroxynonenal
PCR: Polymerase chain reaction
WT: Wild type
EPAL: Epalrestat
ANOVA: Analysis of variance
