# Correction: Motor neuron survival is associated with reduced neuroinflammation and increased autophagy after brachial plexus avulsion injury in aldose reductase-deficient mice

**DOI:** 10.1186/s12974-022-02683-9

**Published:** 2023-01-02

**Authors:** Ke Zhong, Yu Huang, Prince last Mudenda Zilundu, Yaqiong Wang, Yingying Zhou, Guangyin Yu, Rao Fu, Sookja Kim Chung, Yamei Tang, Xiao Cheng, Lihua Zhou

**Affiliations:** 1 Department of Pharmacy, Sun-Yat-Sen Memorial Hospital, Sun Yat-Sen University, 107 Yanjiang West Road, Guangzhou, 510120 Guangdong China; 2 Department of Anatomy, School of Medicine (Shenzhen), Sun Yat-Sen University, Shenzhen, 518000 Guangdong China; 3 Department of Anatomy, Zhongshan School of Medicine, Sun Yat-Sen University, Guangzhou, Guangdong China; 4 Department of Electron Microscope, Zhongshan School of Medicine, Sun Yat-Sen University, Guangzhou, Guangdong China; 5 Department of Anatomy, Neuroscience Laboratory for Cognitive and Developmental Disorders, Medical College of Jinan University, Guangzhou, Guangdong China; 6 Faculty of Medicine, Macau University of Science and Technology, Macau, China; 7 Department of Neurology, Sun Yat-Sen Memorial Hospital, Sun Yat-Sen University, Guangzhou, China; 8 Key Laboratory of Malignant Tumor Gene Regulation and Target Therapy of Guangdong Higher Education Institutes, Sun Yat-Sen University, Guangzhou, China; 9 Guangdong Province Key Laboratory of Brain Function and Disease, Zhongshan School of Medicine, Sun Yat-Sen University, Guangzhou, China; 10 Guangdong Provincial Chinese Emergency Key Laboratory, Guangzhou, Guangdong China; 11 State Key Laboratory of Dampness, Syndrome of Traditional Chinese Medicine, Guangzhou, Guangdong China; 12 Department of Neurology, Guangdong Provincial Hospital of Traditional Chinese Medicine, 111 Dade Road, Guangzhou, Guangdong China

**Correction: Journal of Neuroinflammation (2022) 19:271** 10.1186/s12974-022-02632-6

Following publication of the original article [1], the authors identified that the article note about co-first author and equal contribution were missing.

That is, the author Ke Zhong and Yu Huang belong to co-first author and contributed equally.

It has been added in this correction and the original article [1] has been updated.
