# Supplementary figures and images for: Motor neuron survival is associated with reduced neuroinflammation and increased autophagy after brachial plexus avulsion injury in aldose reductase-deficient mice (part 1 of 2)
Source: J Neuroinflammation. 2022 Nov 9;19:271. doi: 10.1186/s12974-022-02632-6 (PMC9648007; doi:10.1186/s12974-022-02632-6)

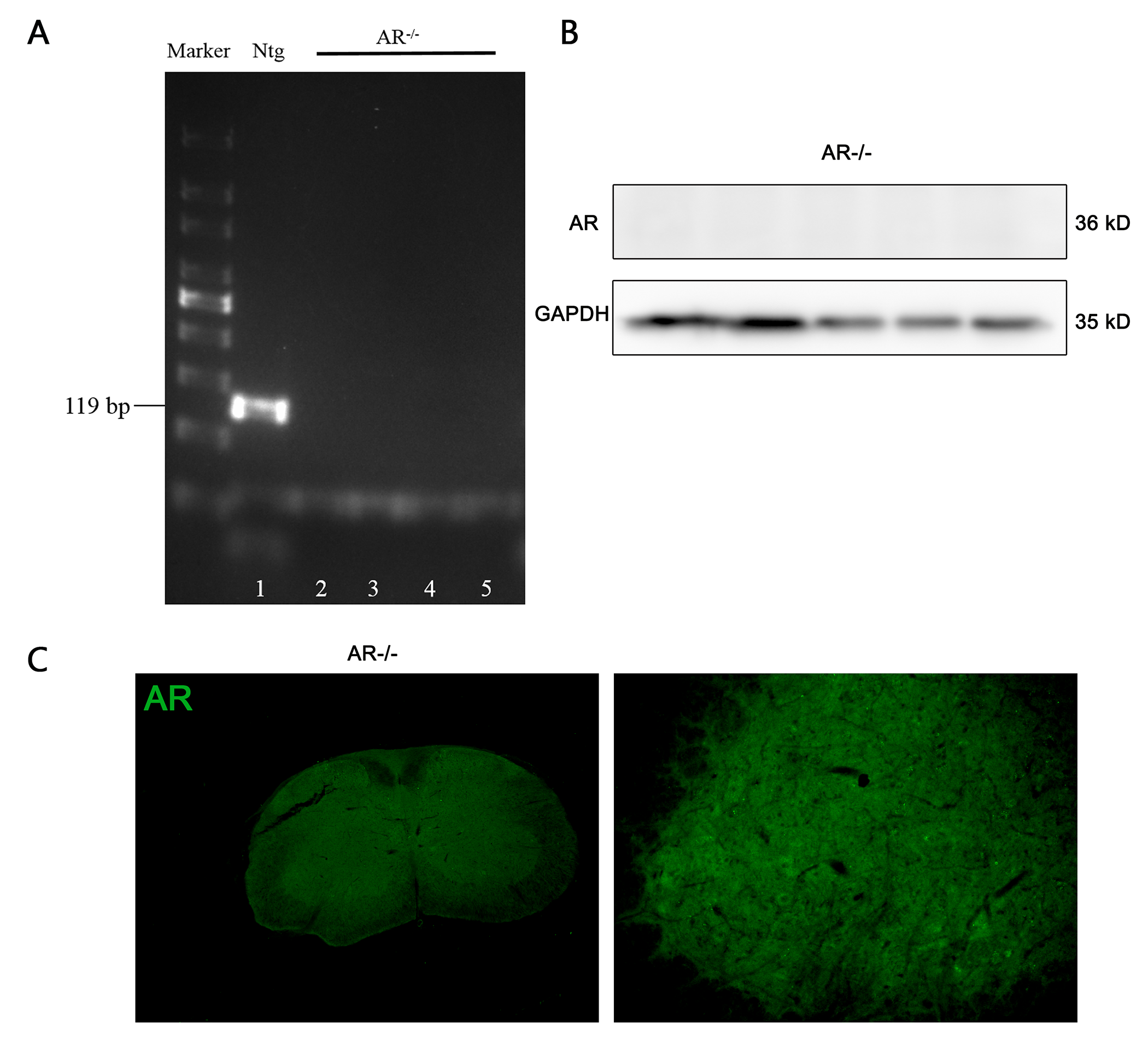

Supplement: Supplementary file 1 — Additional file 1: Figure S1. The AR−/− mice genotyping and the expression level of AR in AR−/− mice. (A) AR null allele were identified by northern blot analysis. The expected bands of ∼119 bp is observed in the wild-type mice while not seen in AR−/− mice. (B) Western blot analyze the AR expression in the AR−/− mice. (C) Immunofluorescence analyze the AR expression in the spinal cord of AR−/− mice at 3 day following BPRA injury. [file 12974_2022_2632_MOESM1_ESM.tif]

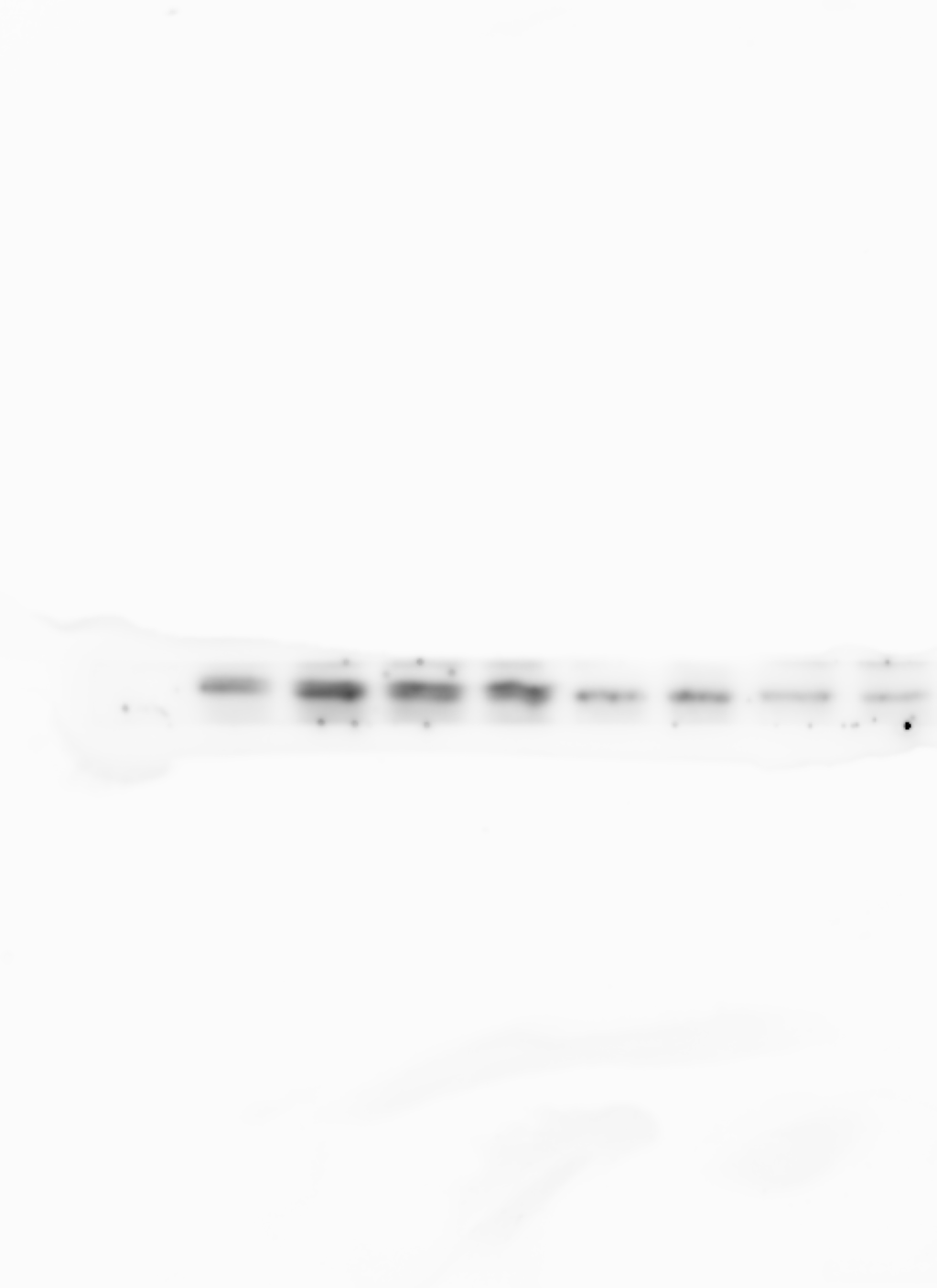

Supplement: Supplementary file 2 — Additional file 2. Raw data of western blot. [file 12974_2022_2632_MOESM2_ESM.zip › supplementary files/Figure1 WB/3ar 2020.01.18_14.13.25_Ch/3ar 2020.01.18_14.13.25_Ch.tif]

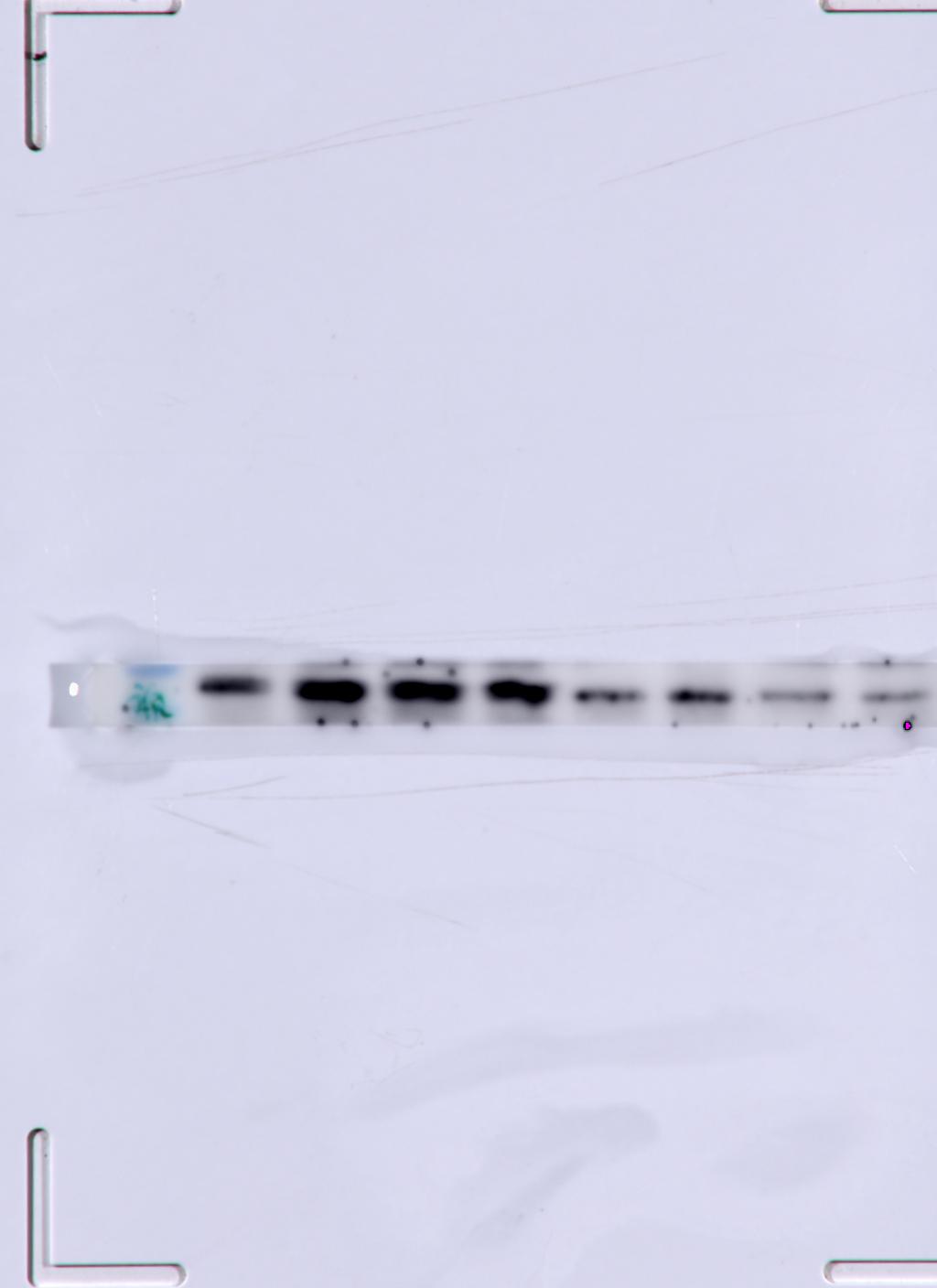

Supplement: Supplementary file 2 — Additional file 2. Raw data of western blot. [file 12974_2022_2632_MOESM2_ESM.zip › supplementary files/Figure1 WB/3ar 2020.01.18_14.13.25_Ch/3ar 2020.01.18_14.13.25_Ch+Marker.jpg]

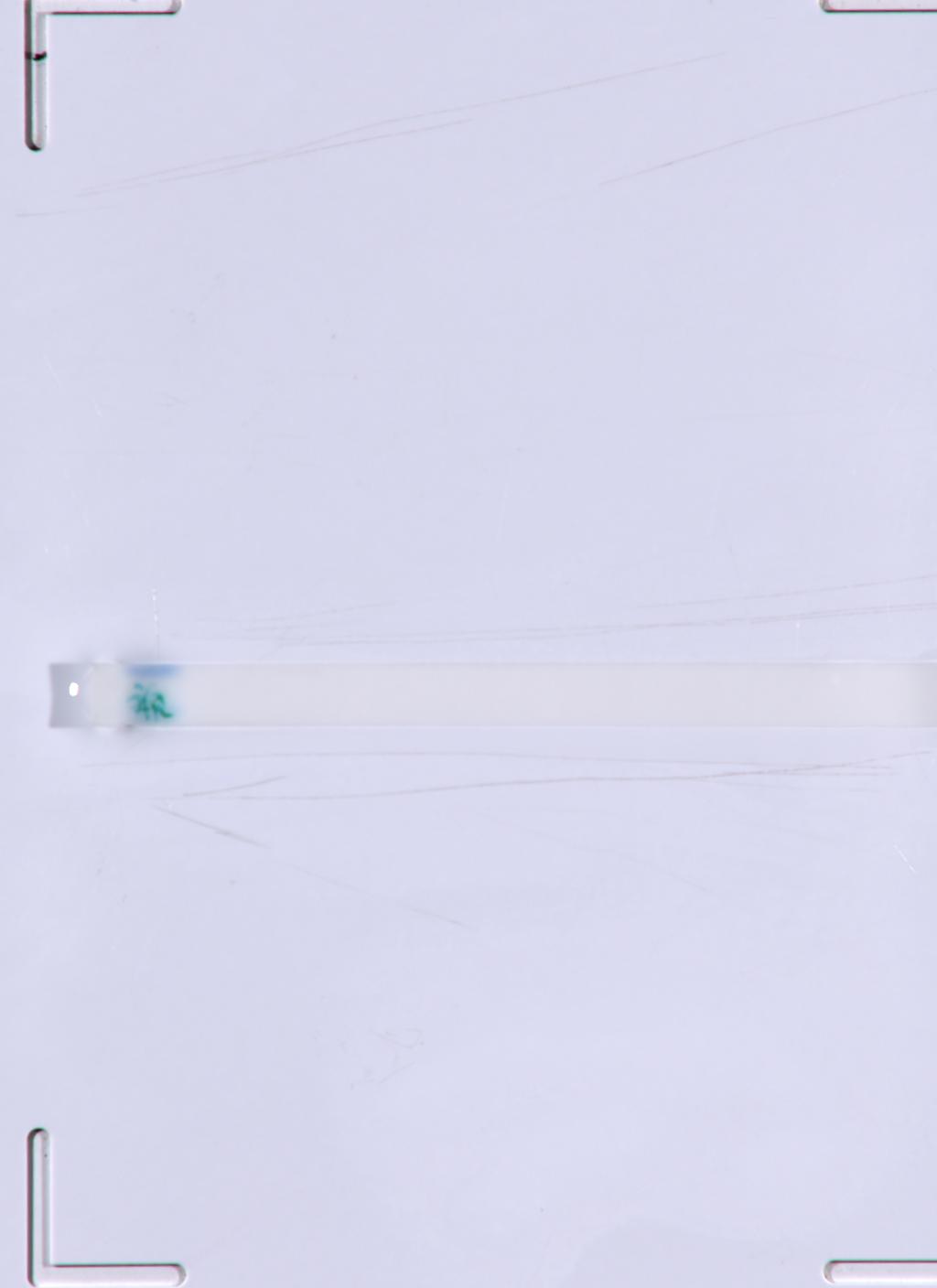

Supplement: Supplementary file 2 — Additional file 2. Raw data of western blot. [file 12974_2022_2632_MOESM2_ESM.zip › supplementary files/Figure1 WB/3ar 2020.01.18_14.13.25_Ch/3ar 2020.01.18_14.13.25_Ch-Marker.jpg]

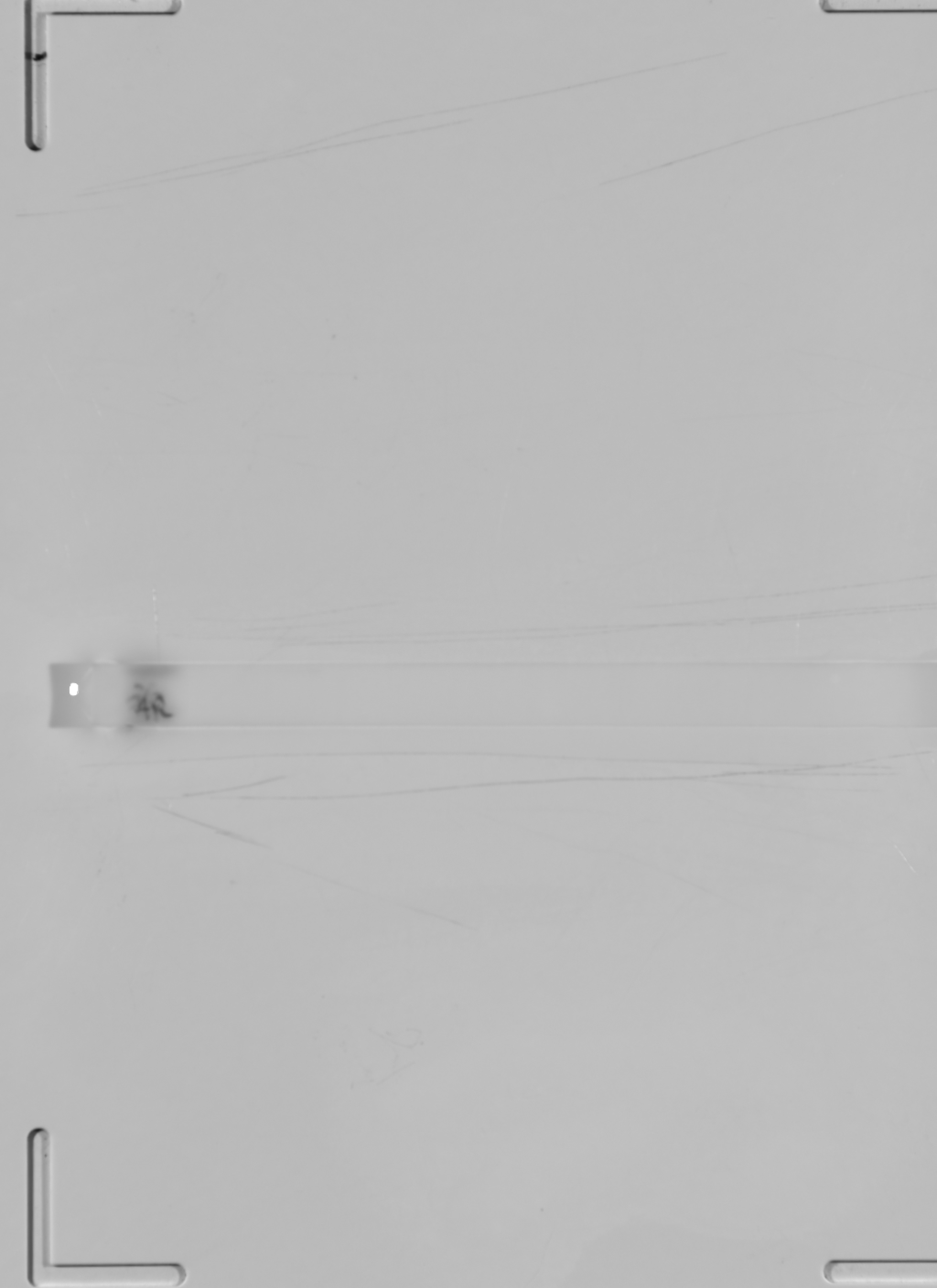

Supplement: Supplementary file 2 — Additional file 2. Raw data of western blot. [file 12974_2022_2632_MOESM2_ESM.zip › supplementary files/Figure1 WB/3ar 2020.01.18_14.13.25_Ch/3ar 2020.01.18_14.13.25_Ch-Marker.tif]

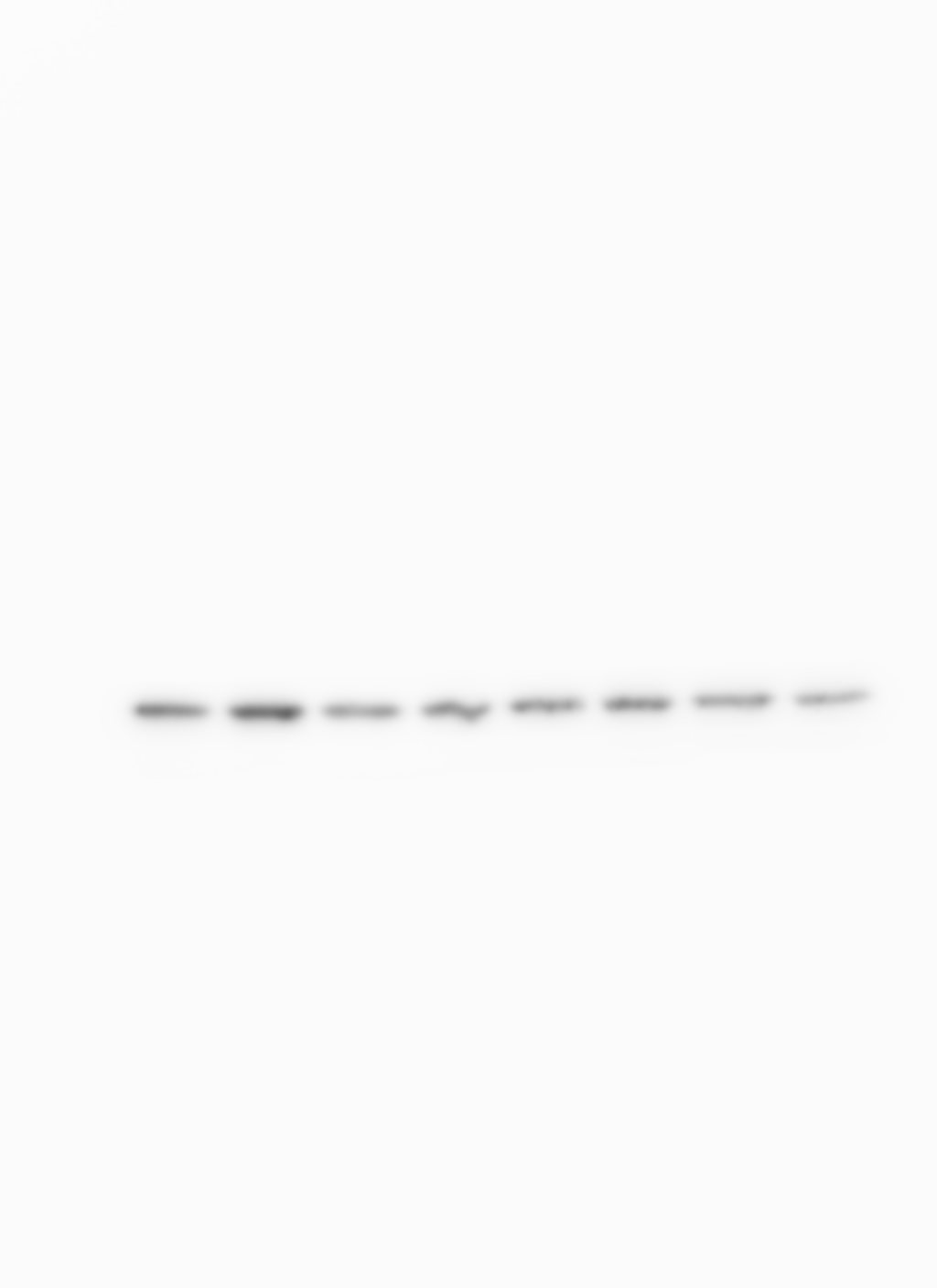

Supplement: Supplementary file 2 — Additional file 2. Raw data of western blot. [file 12974_2022_2632_MOESM2_ESM.zip › supplementary files/Figure1 WB/3gapdh 2020.01.16_15.29.14_Ch/3gapdh 2020.01.16_15.29.14_Ch.tif]

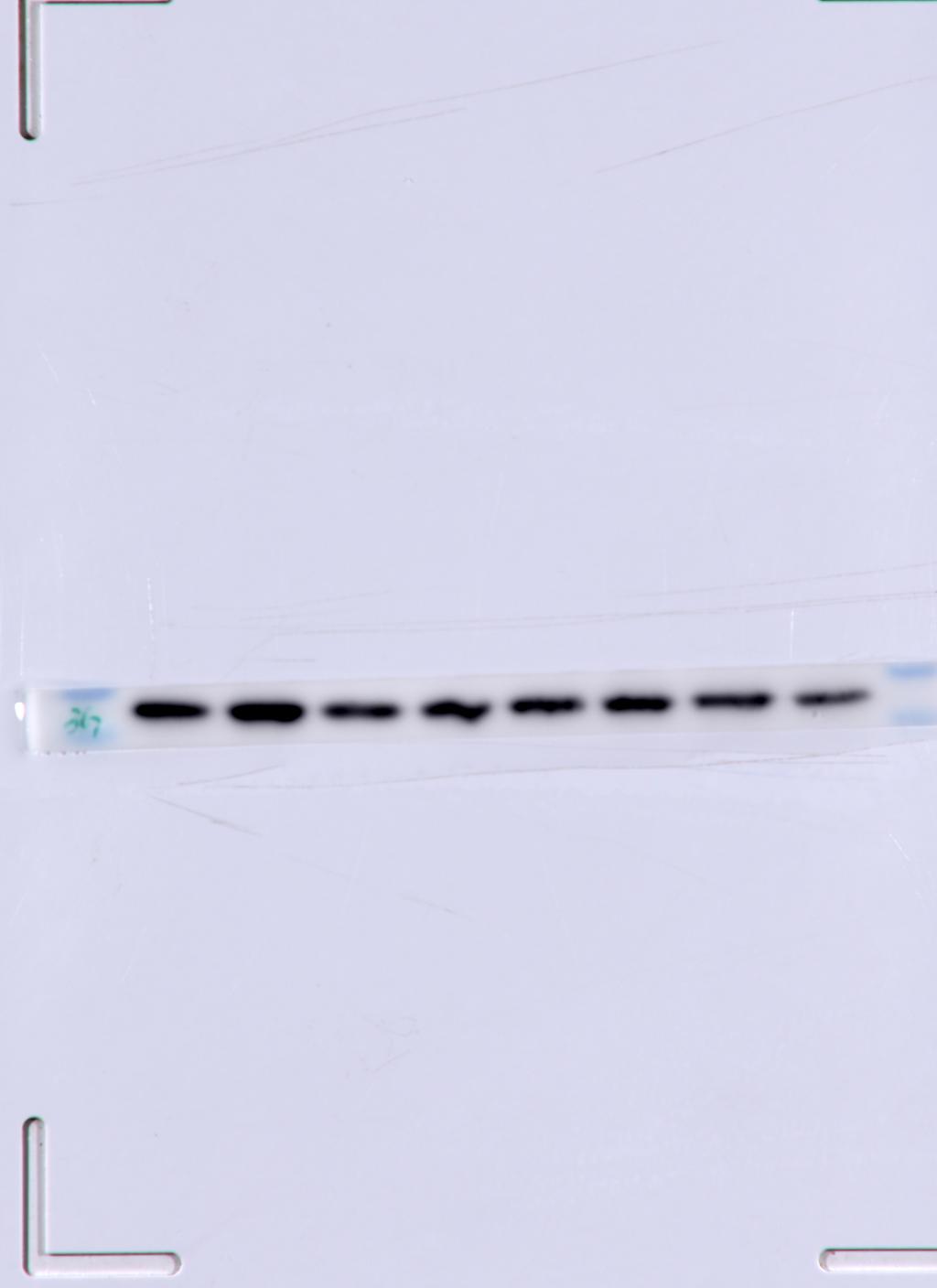

Supplement: Supplementary file 2 — Additional file 2. Raw data of western blot. [file 12974_2022_2632_MOESM2_ESM.zip › supplementary files/Figure1 WB/3gapdh 2020.01.16_15.29.14_Ch/3gapdh 2020.01.16_15.29.14_Ch+Marker.jpg]

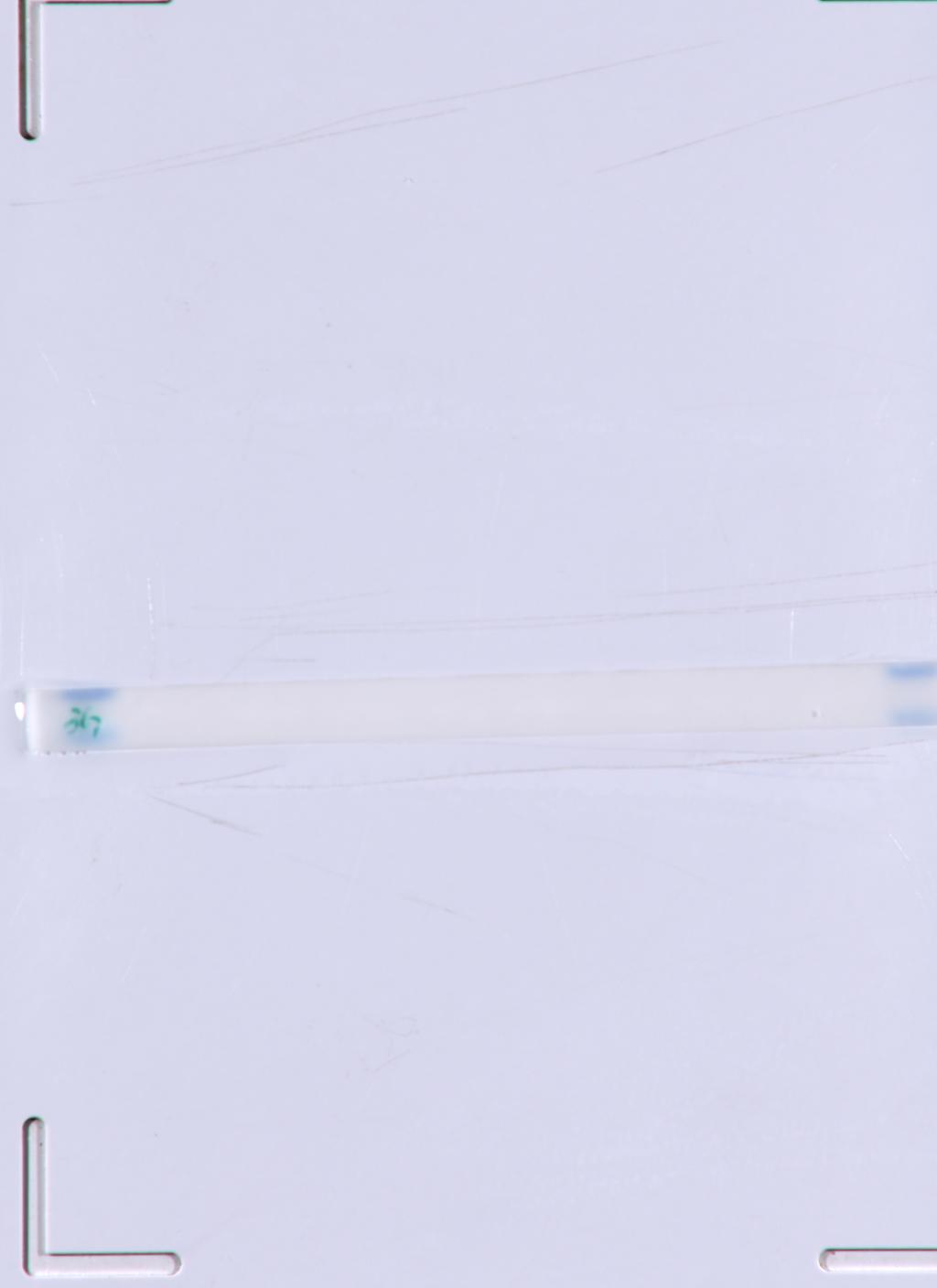

Supplement: Supplementary file 2 — Additional file 2. Raw data of western blot. [file 12974_2022_2632_MOESM2_ESM.zip › supplementary files/Figure1 WB/3gapdh 2020.01.16_15.29.14_Ch/3gapdh 2020.01.16_15.29.14_Ch-Marker.jpg]

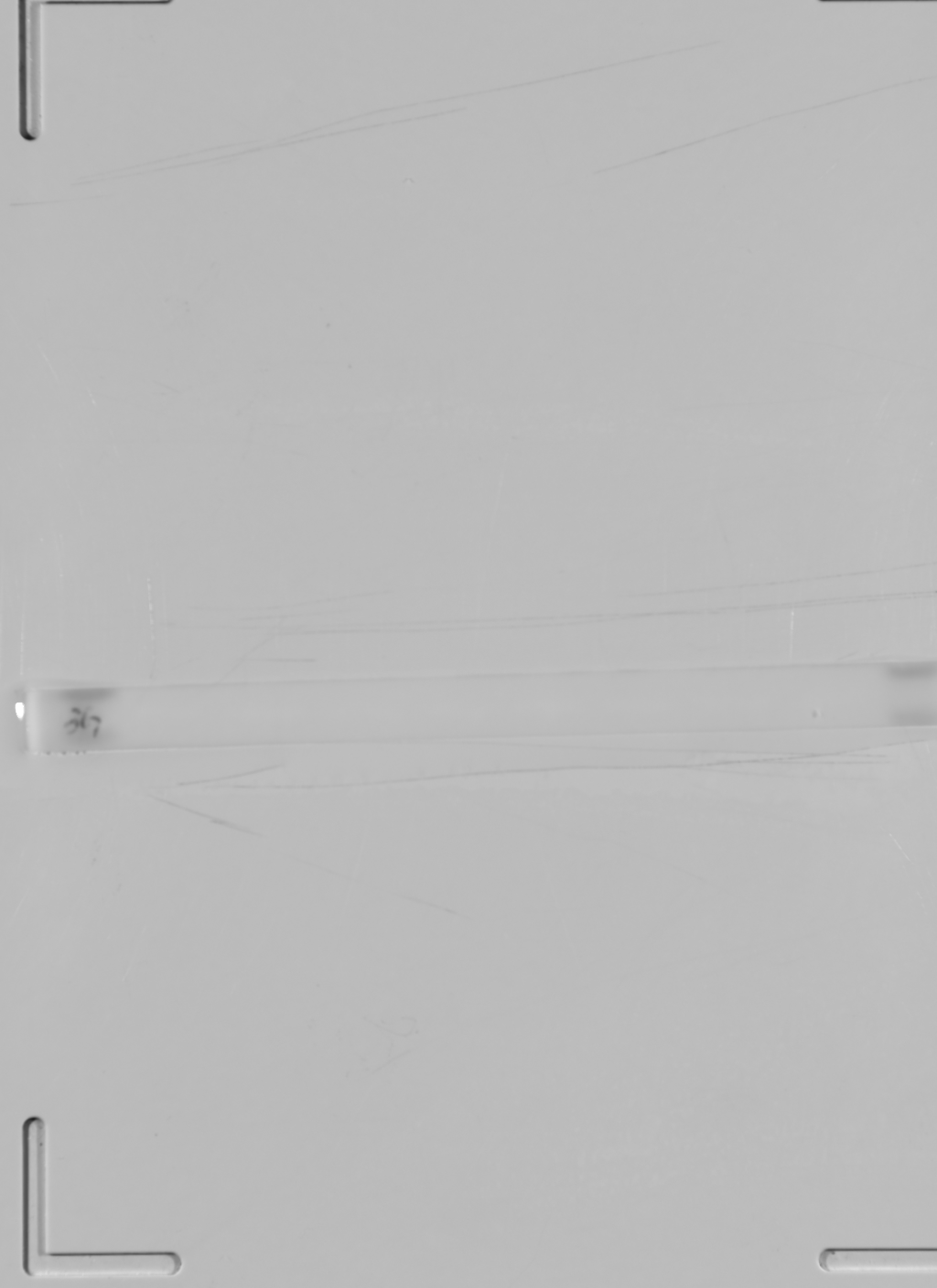

Supplement: Supplementary file 2 — Additional file 2. Raw data of western blot. [file 12974_2022_2632_MOESM2_ESM.zip › supplementary files/Figure1 WB/3gapdh 2020.01.16_15.29.14_Ch/3gapdh 2020.01.16_15.29.14_Ch-Marker.tif]

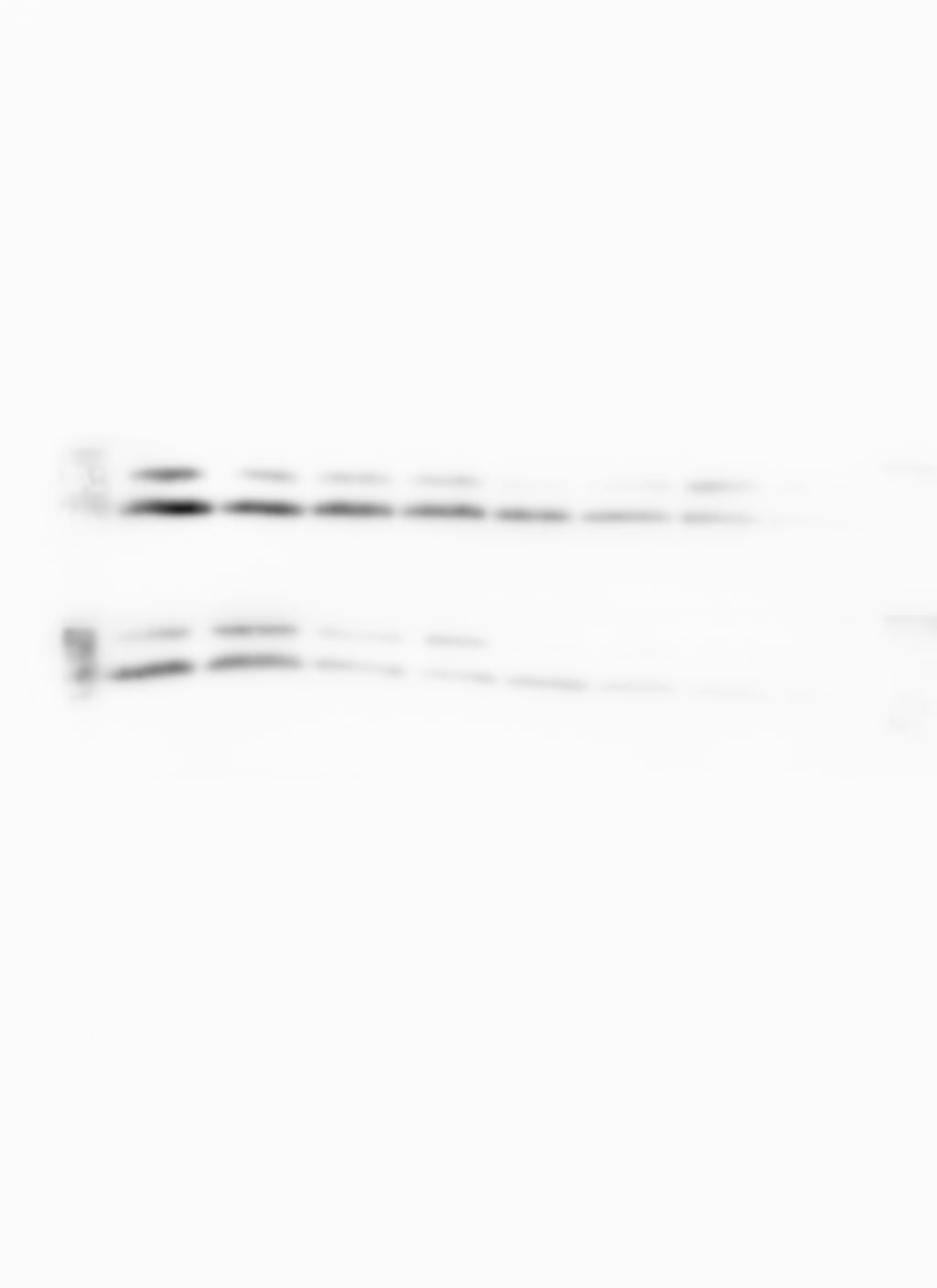

Supplement: Supplementary file 2 — Additional file 2. Raw data of western blot. [file 12974_2022_2632_MOESM2_ESM.zip › supplementary files/Figure2-2 WB/c-caspase3 new/c-c 2020.08.17_17.44.41_Ch/c-c 2020.08.17_17.44.41_Ch.tif]

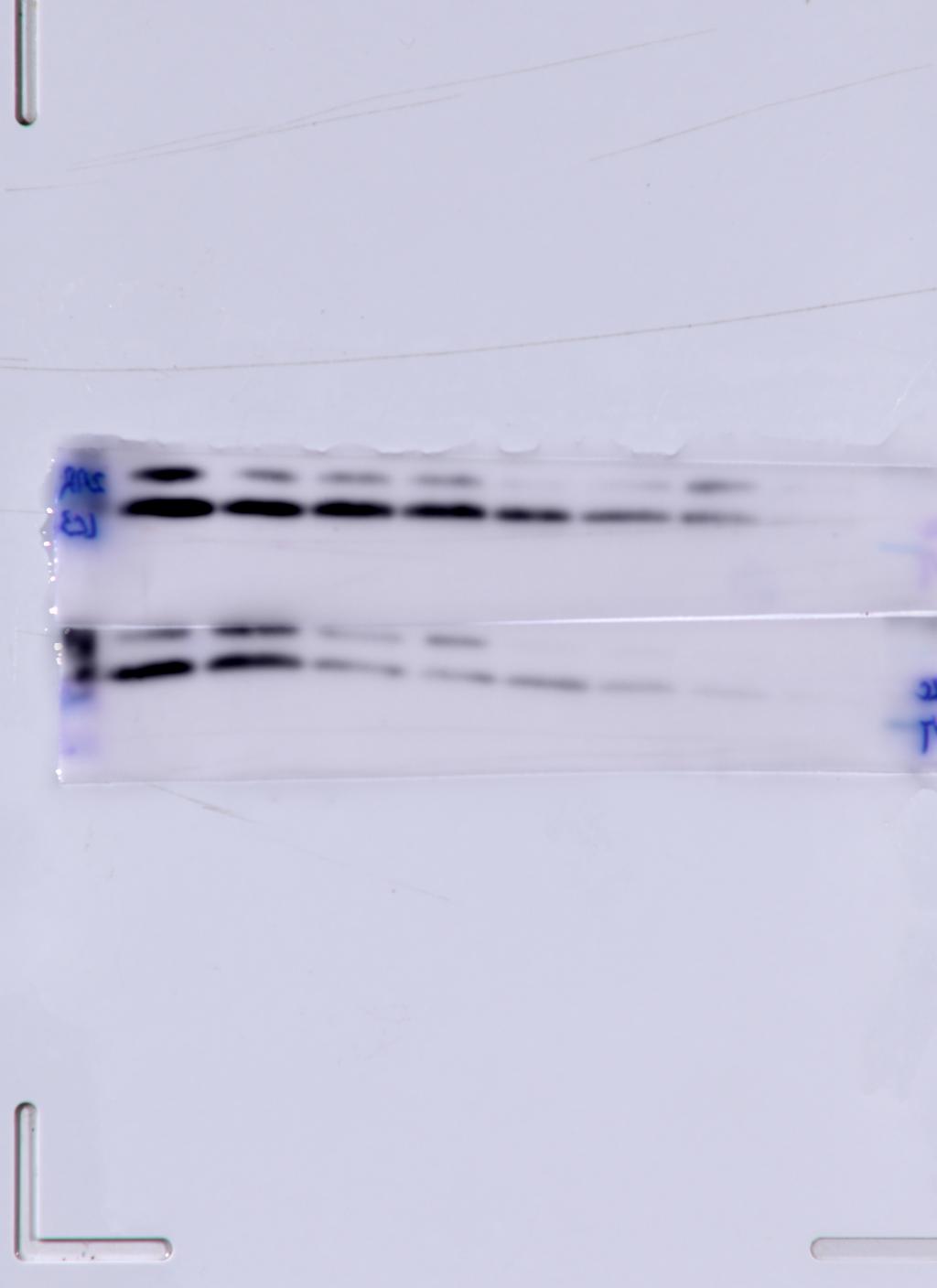

Supplement: Supplementary file 2 — Additional file 2. Raw data of western blot. [file 12974_2022_2632_MOESM2_ESM.zip › supplementary files/Figure2-2 WB/c-caspase3 new/c-c 2020.08.17_17.44.41_Ch/c-c 2020.08.17_17.44.41_Ch+Marker.jpg]

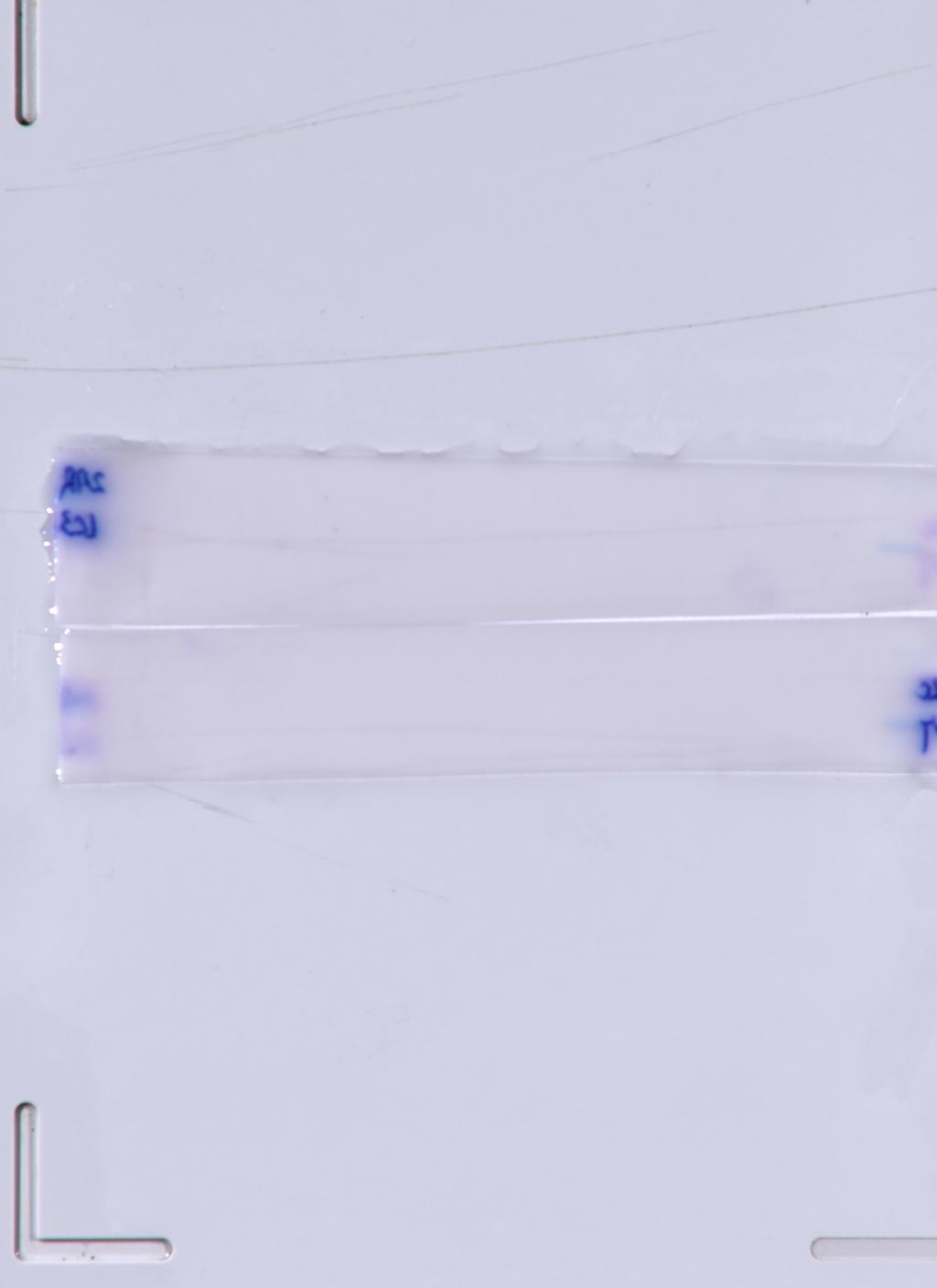

Supplement: Supplementary file 2 — Additional file 2. Raw data of western blot. [file 12974_2022_2632_MOESM2_ESM.zip › supplementary files/Figure2-2 WB/c-caspase3 new/c-c 2020.08.17_17.44.41_Ch/c-c 2020.08.17_17.44.41_Ch-Marker.jpg]

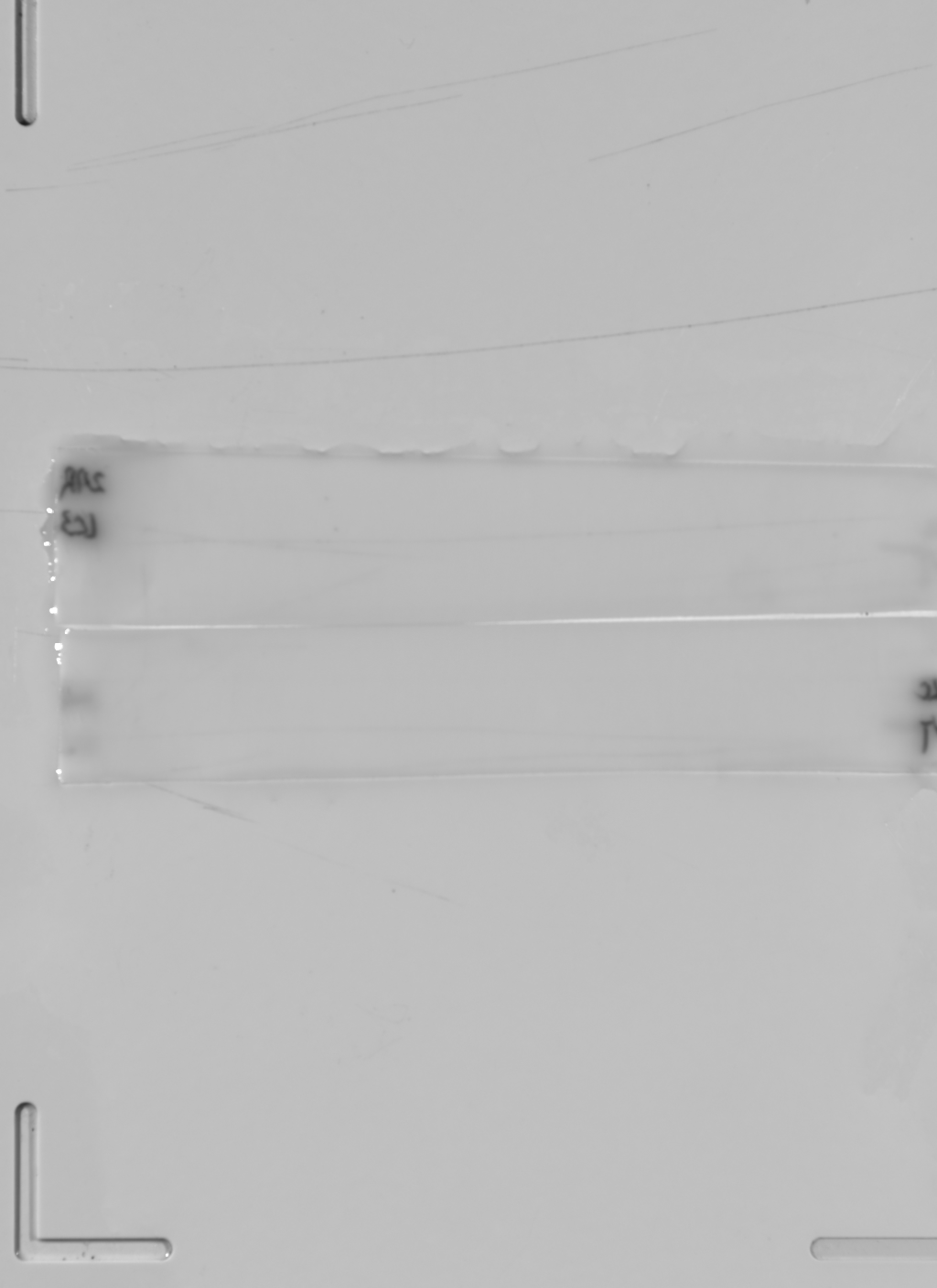

Supplement: Supplementary file 2 — Additional file 2. Raw data of western blot. [file 12974_2022_2632_MOESM2_ESM.zip › supplementary files/Figure2-2 WB/c-caspase3 new/c-c 2020.08.17_17.44.41_Ch/c-c 2020.08.17_17.44.41_Ch-Marker.tif]

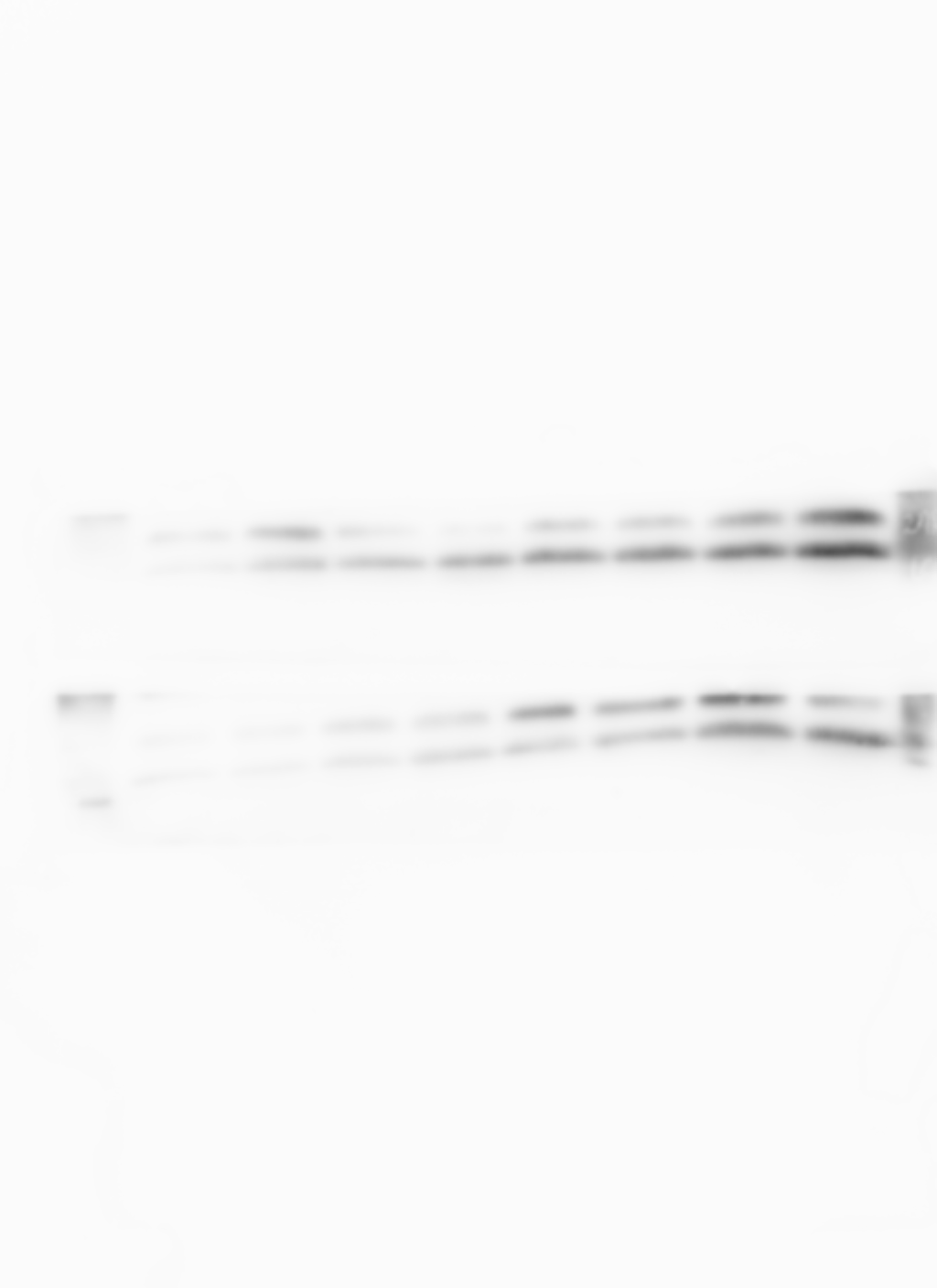

Supplement: Supplementary file 2 — Additional file 2. Raw data of western blot. [file 12974_2022_2632_MOESM2_ESM.zip › supplementary files/Figure2-2 WB/C-Caspase3/AR_Ch.tif]

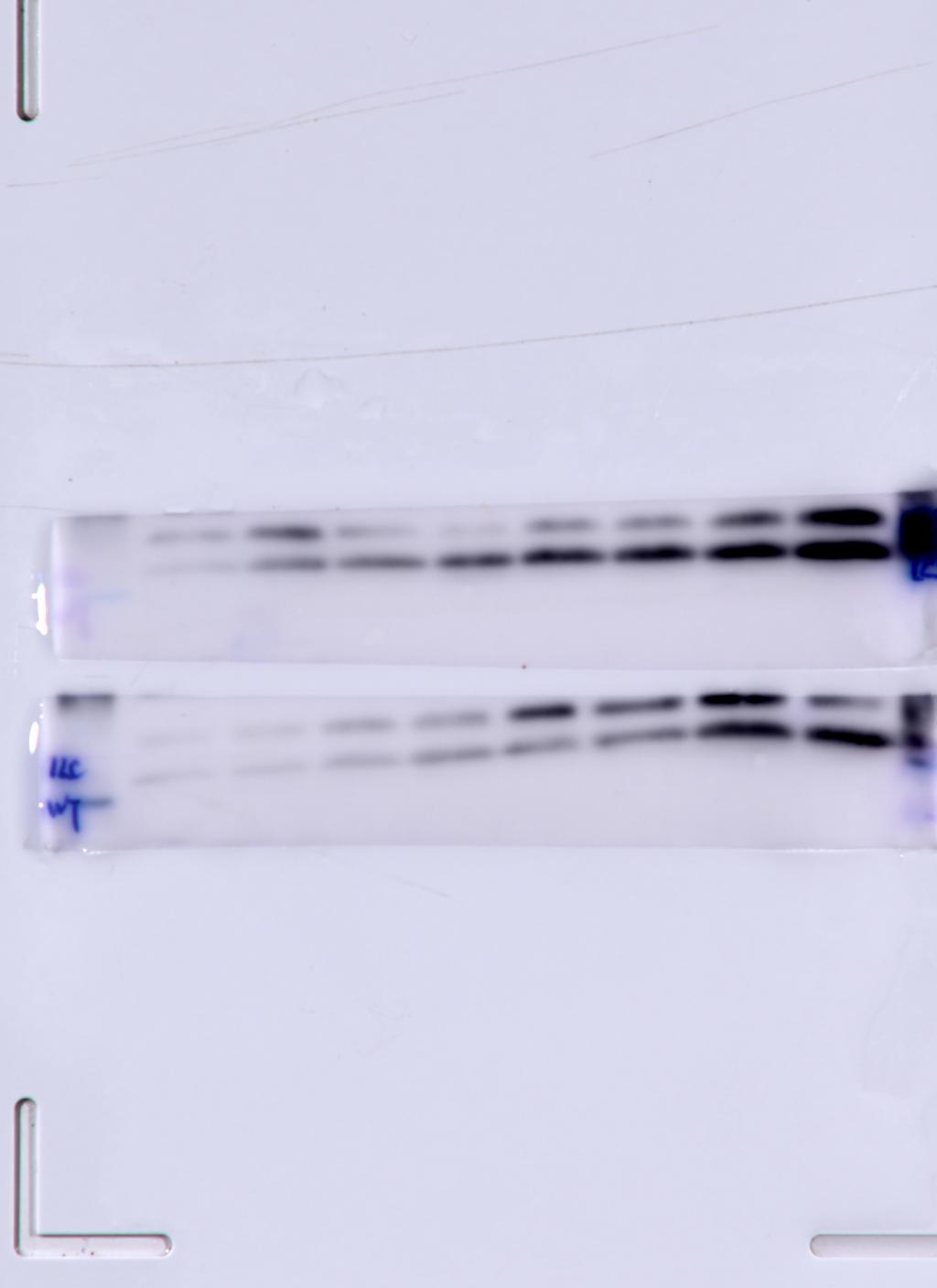

Supplement: Supplementary file 2 — Additional file 2. Raw data of western blot. [file 12974_2022_2632_MOESM2_ESM.zip › supplementary files/Figure2-2 WB/C-Caspase3/AR_Ch+Marker.jpg]

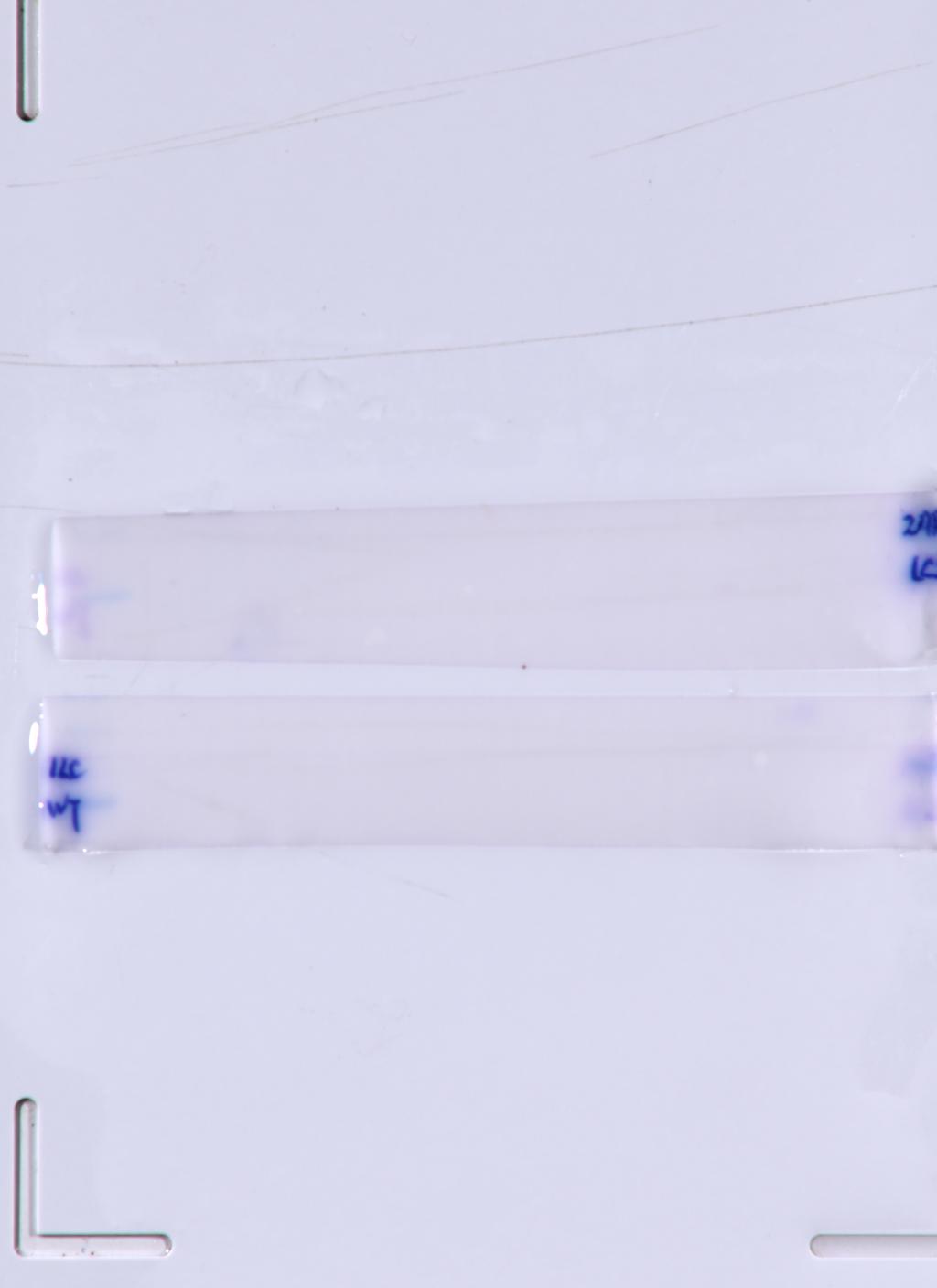

Supplement: Supplementary file 2 — Additional file 2. Raw data of western blot. [file 12974_2022_2632_MOESM2_ESM.zip › supplementary files/Figure2-2 WB/C-Caspase3/AR_Ch-Marker.jpg]

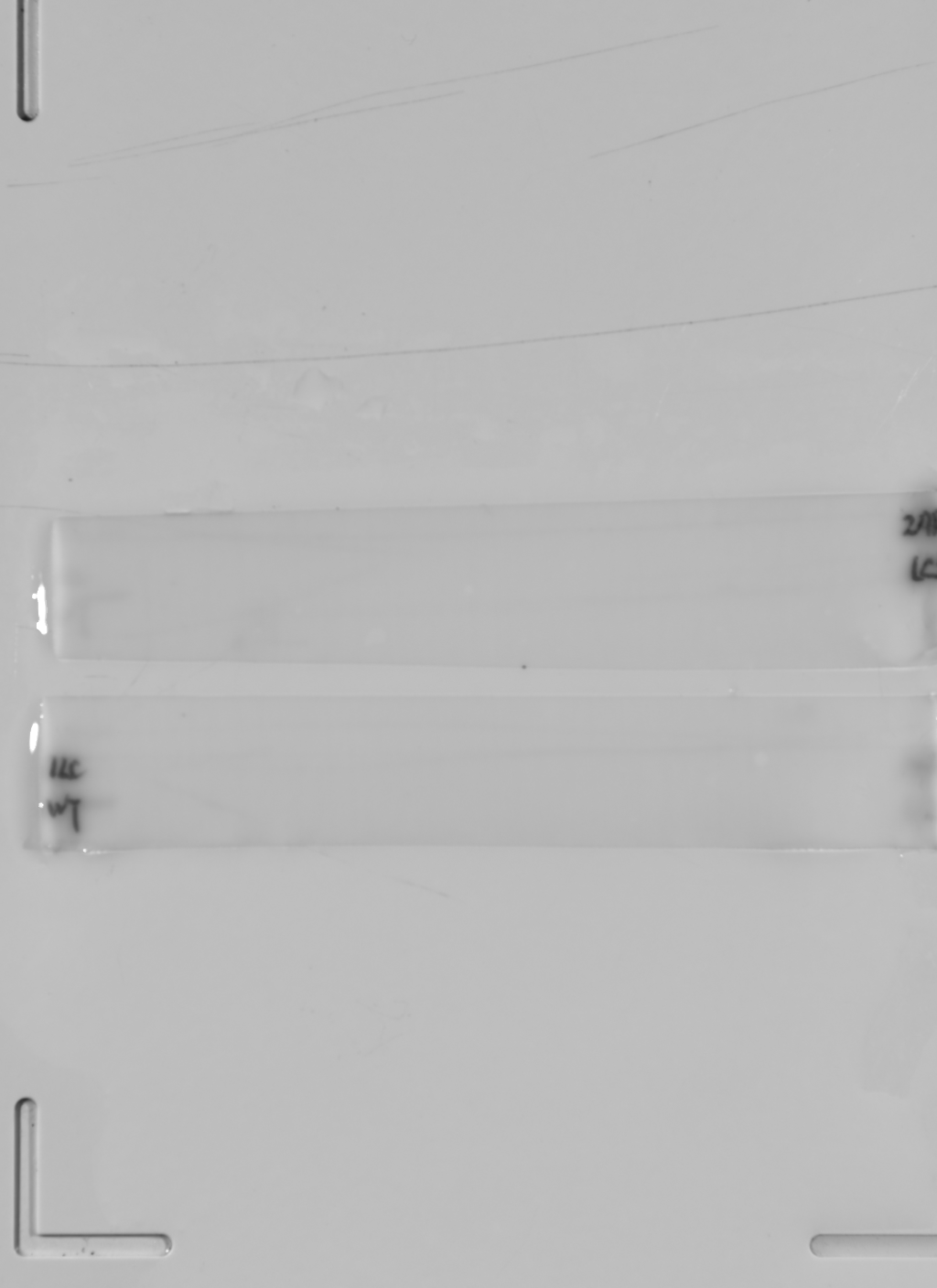

Supplement: Supplementary file 2 — Additional file 2. Raw data of western blot. [file 12974_2022_2632_MOESM2_ESM.zip › supplementary files/Figure2-2 WB/C-Caspase3/AR_Ch-Marker.tif]

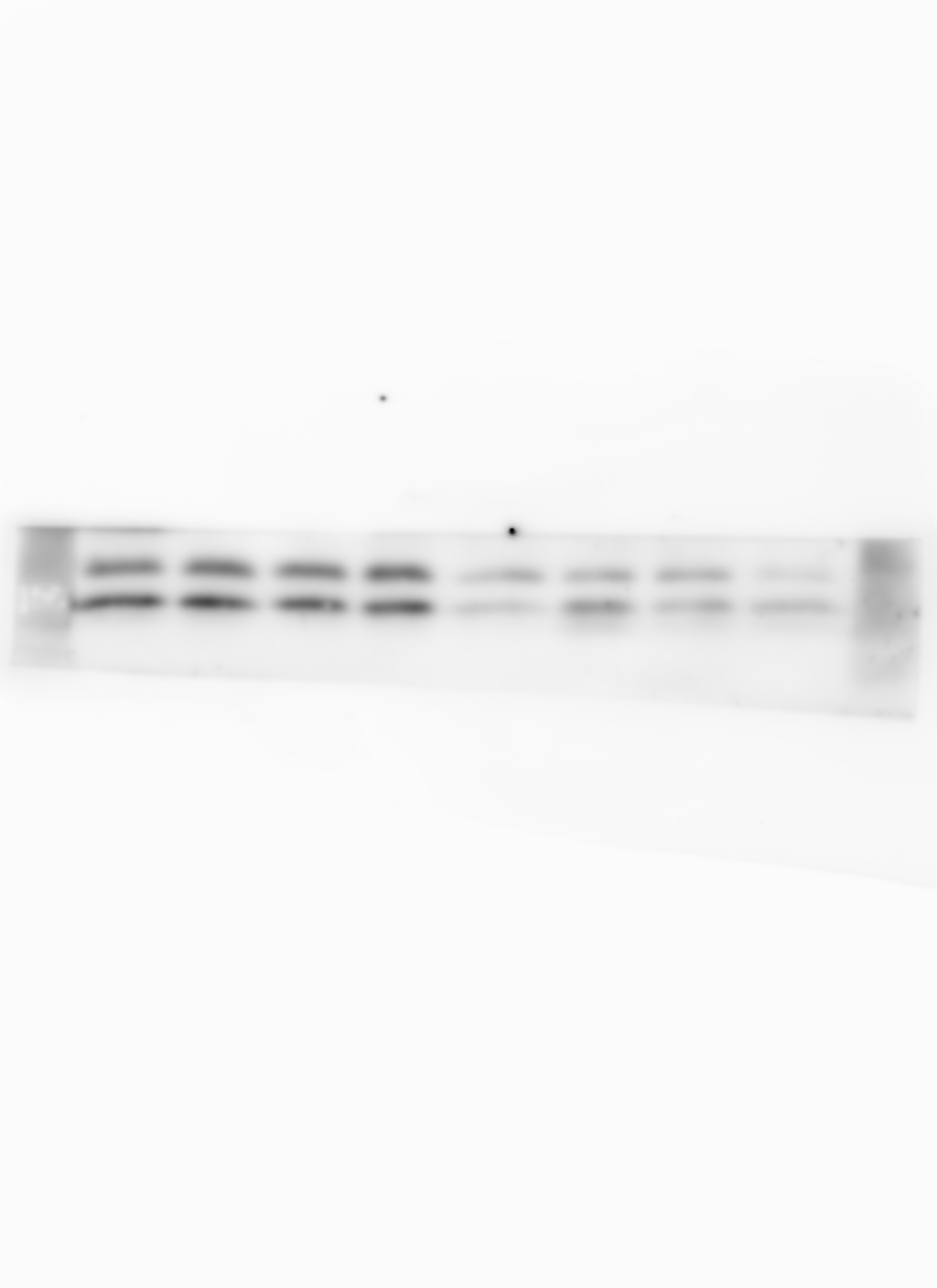

Supplement: Supplementary file 2 — Additional file 2. Raw data of western blot. [file 12974_2022_2632_MOESM2_ESM.zip › supplementary files/Figure2-2 WB/C-Caspase3/WT_Ch.tif]

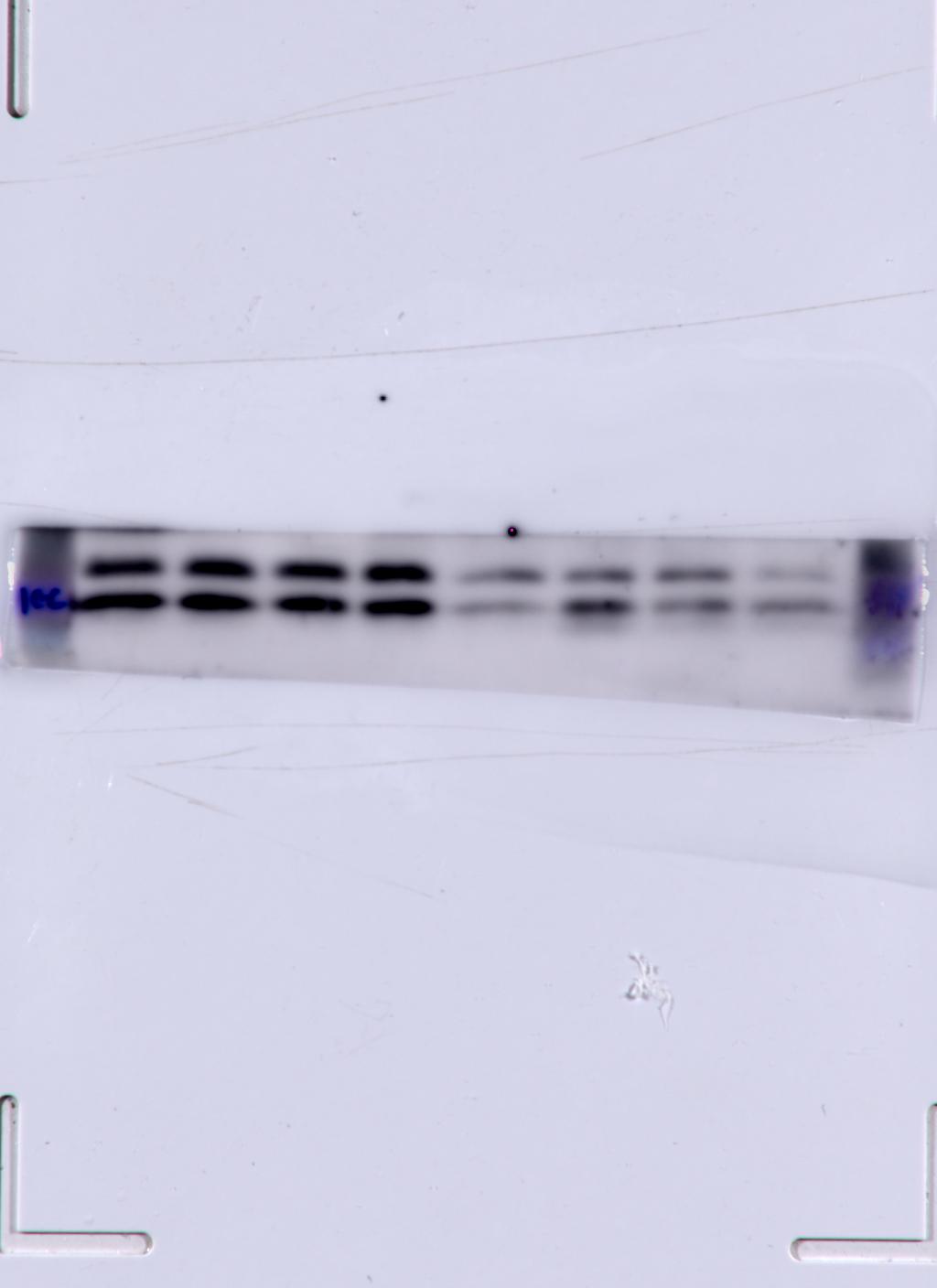

Supplement: Supplementary file 2 — Additional file 2. Raw data of western blot. [file 12974_2022_2632_MOESM2_ESM.zip › supplementary files/Figure2-2 WB/C-Caspase3/WT_Ch+Marker.jpg]

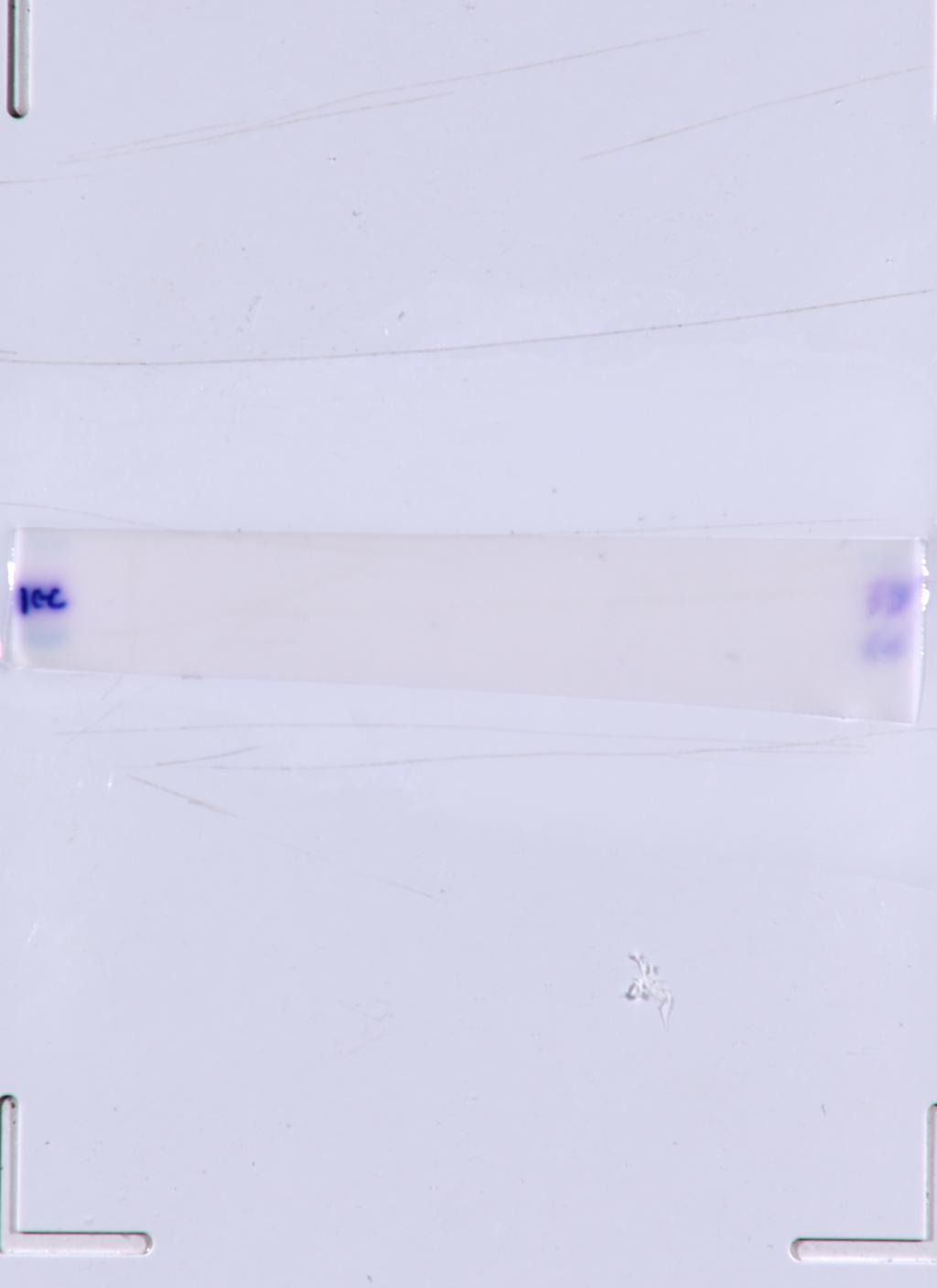

Supplement: Supplementary file 2 — Additional file 2. Raw data of western blot. [file 12974_2022_2632_MOESM2_ESM.zip › supplementary files/Figure2-2 WB/C-Caspase3/WT_Ch-Marker.jpg]

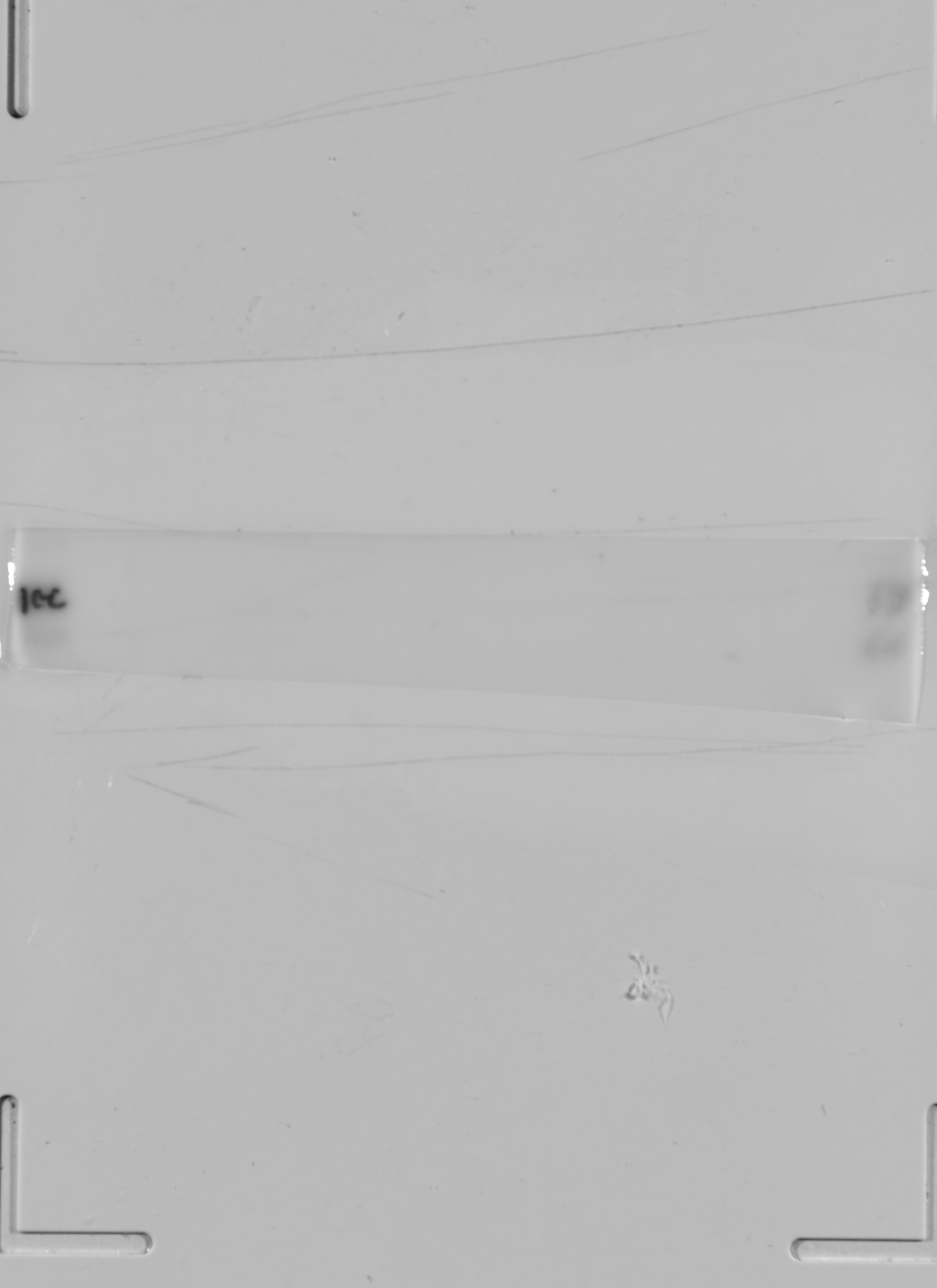

Supplement: Supplementary file 2 — Additional file 2. Raw data of western blot. [file 12974_2022_2632_MOESM2_ESM.zip › supplementary files/Figure2-2 WB/C-Caspase3/WT_Ch-Marker.tif]

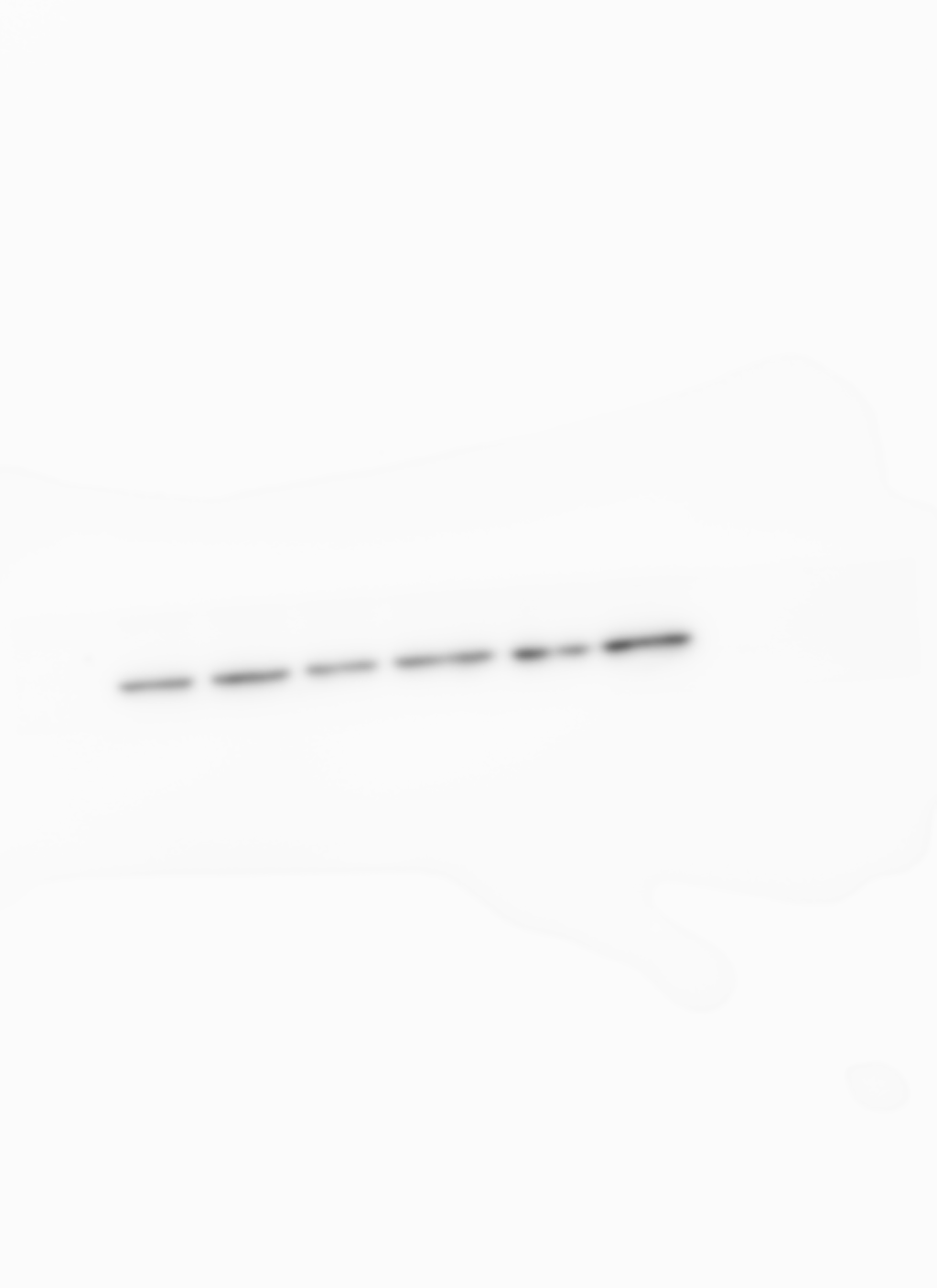

Supplement: Supplementary file 2 — Additional file 2. Raw data of western blot. [file 12974_2022_2632_MOESM2_ESM.zip › supplementary files/Figure2-2 WB/gap43 new.10_Ch/1gap43 2020.09.08_18.01.10_Ch.tif]

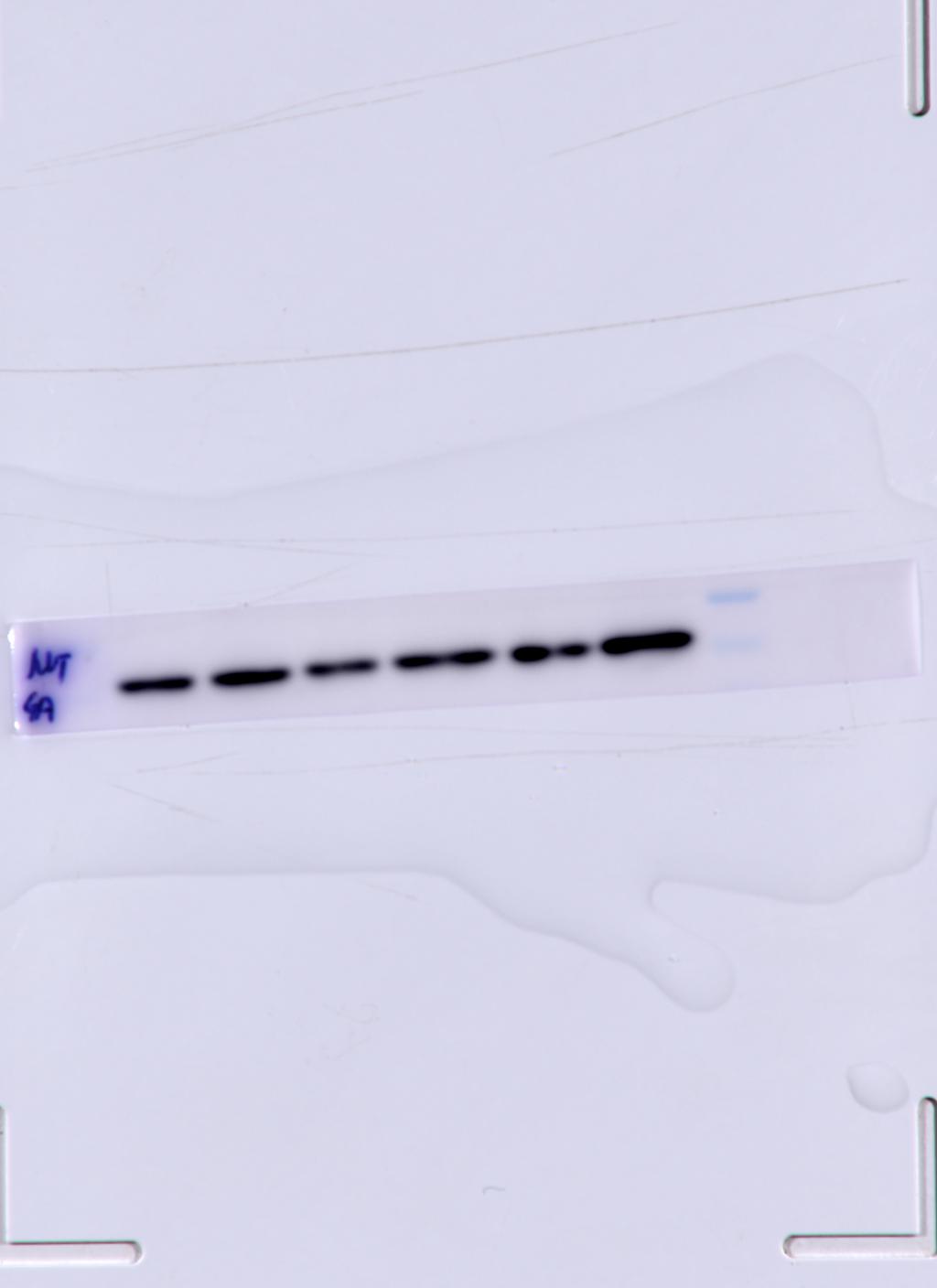

Supplement: Supplementary file 2 — Additional file 2. Raw data of western blot. [file 12974_2022_2632_MOESM2_ESM.zip › supplementary files/Figure2-2 WB/gap43 new.10_Ch/1gap43 2020.09.08_18.01.10_Ch+Marker.jpg]

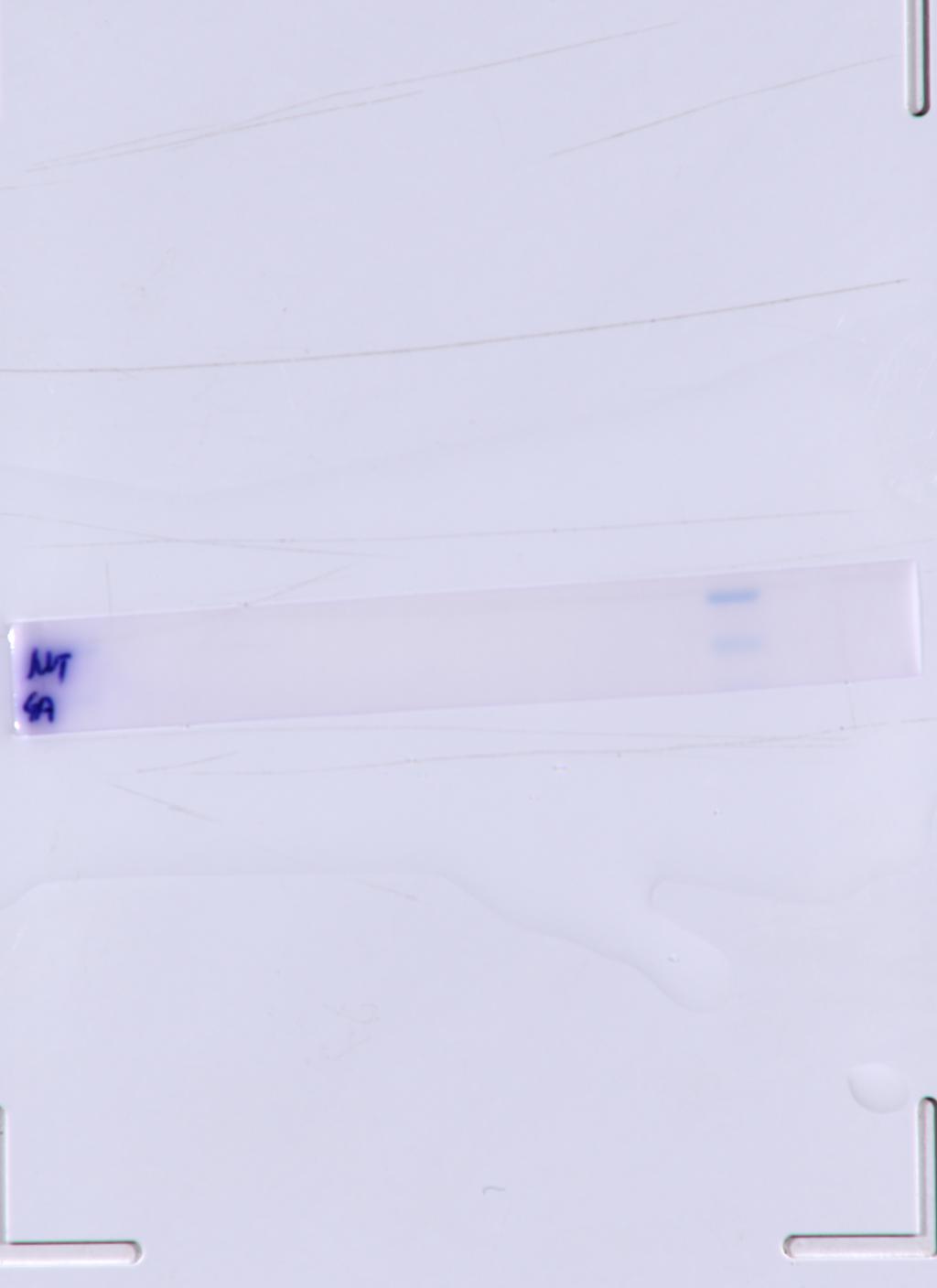

Supplement: Supplementary file 2 — Additional file 2. Raw data of western blot. [file 12974_2022_2632_MOESM2_ESM.zip › supplementary files/Figure2-2 WB/gap43 new.10_Ch/1gap43 2020.09.08_18.01.10_Ch-Marker.jpg]

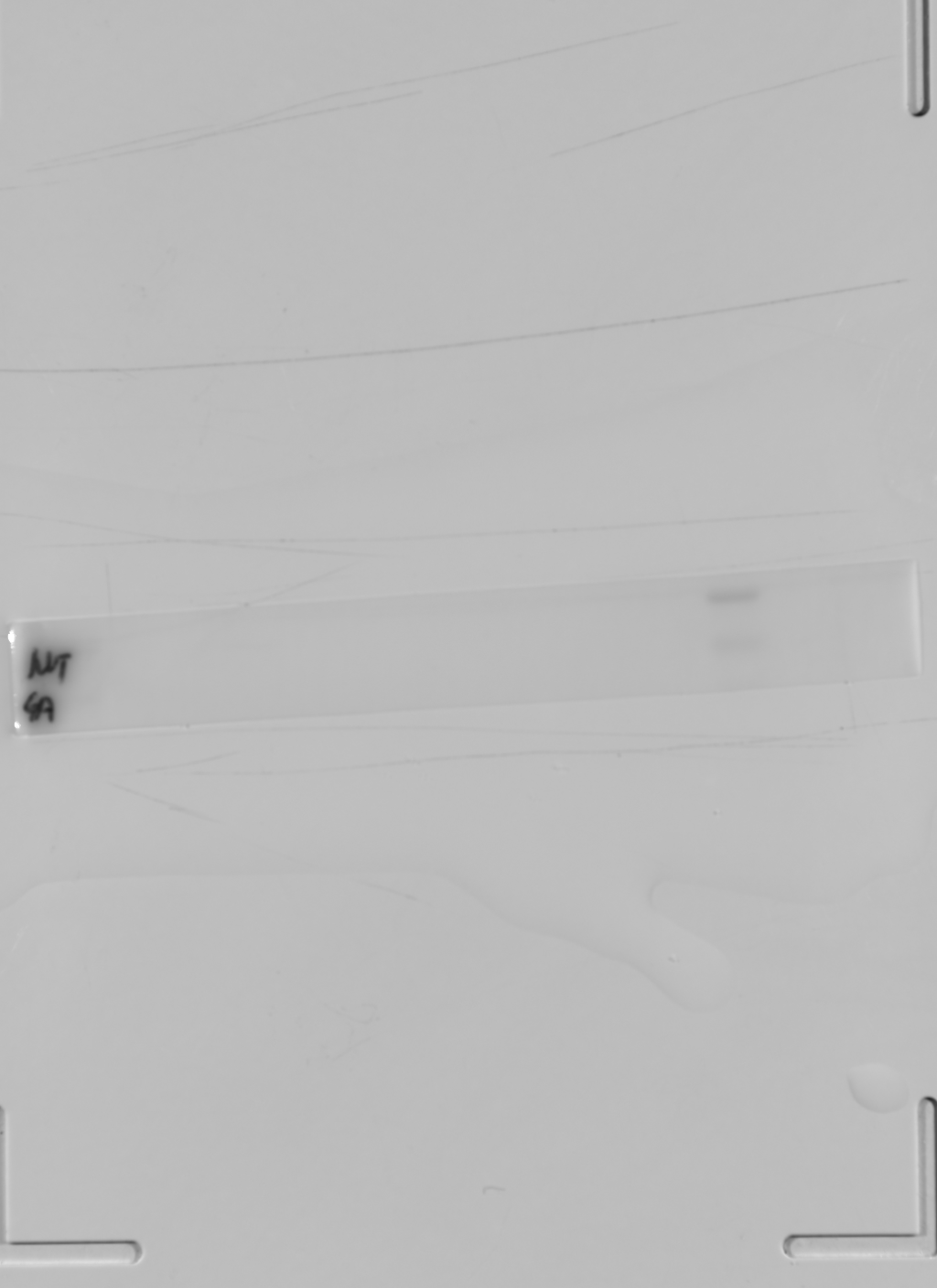

Supplement: Supplementary file 2 — Additional file 2. Raw data of western blot. [file 12974_2022_2632_MOESM2_ESM.zip › supplementary files/Figure2-2 WB/gap43 new.10_Ch/1gap43 2020.09.08_18.01.10_Ch-Marker.tif]

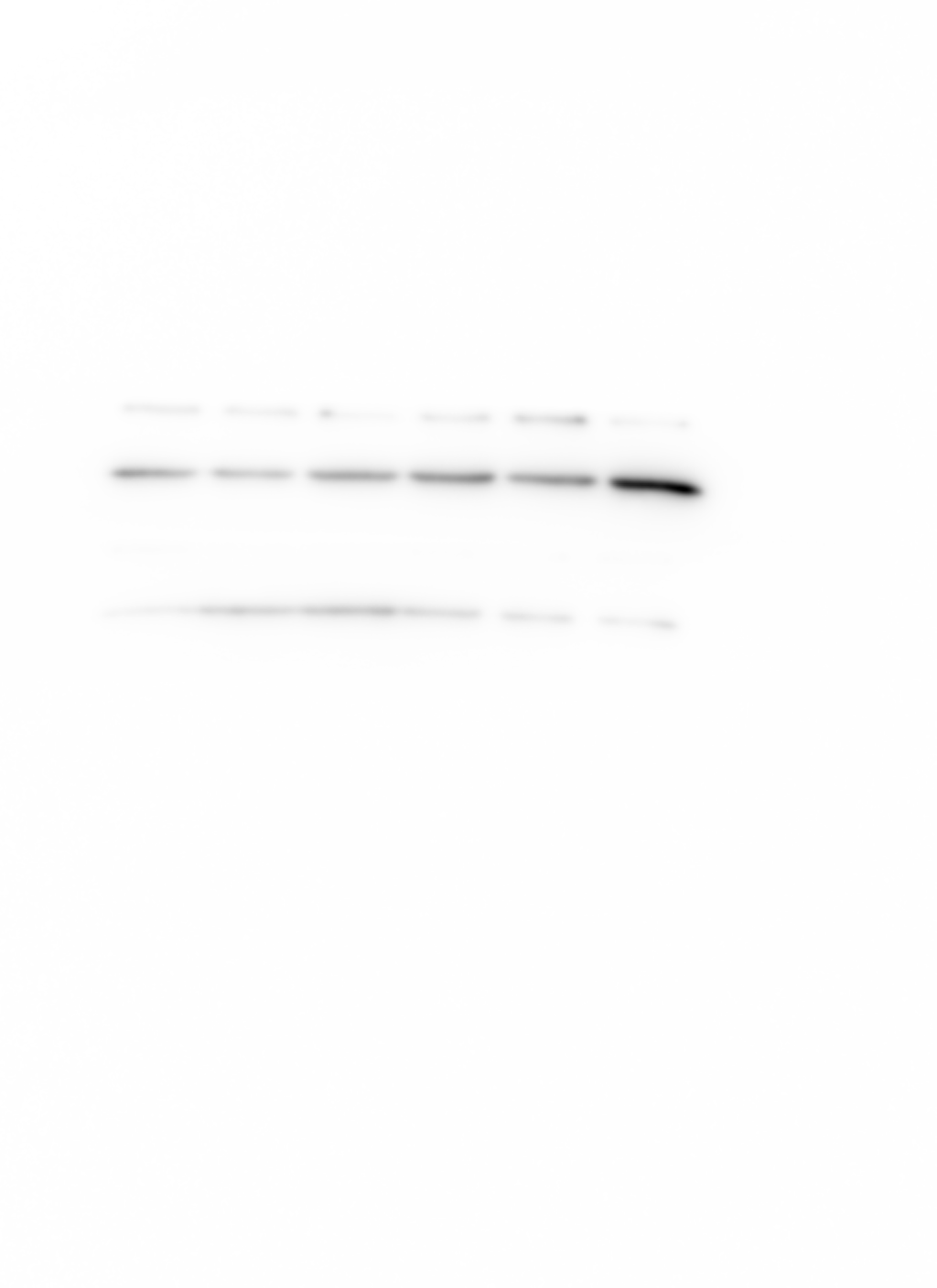

Supplement: Supplementary file 2 — Additional file 2. Raw data of western blot. [file 12974_2022_2632_MOESM2_ESM.zip › supplementary files/Figure2-2 WB/gap43 new.10_Ch/GAP43 2020.09.18_12.28.33_Ch.tif]

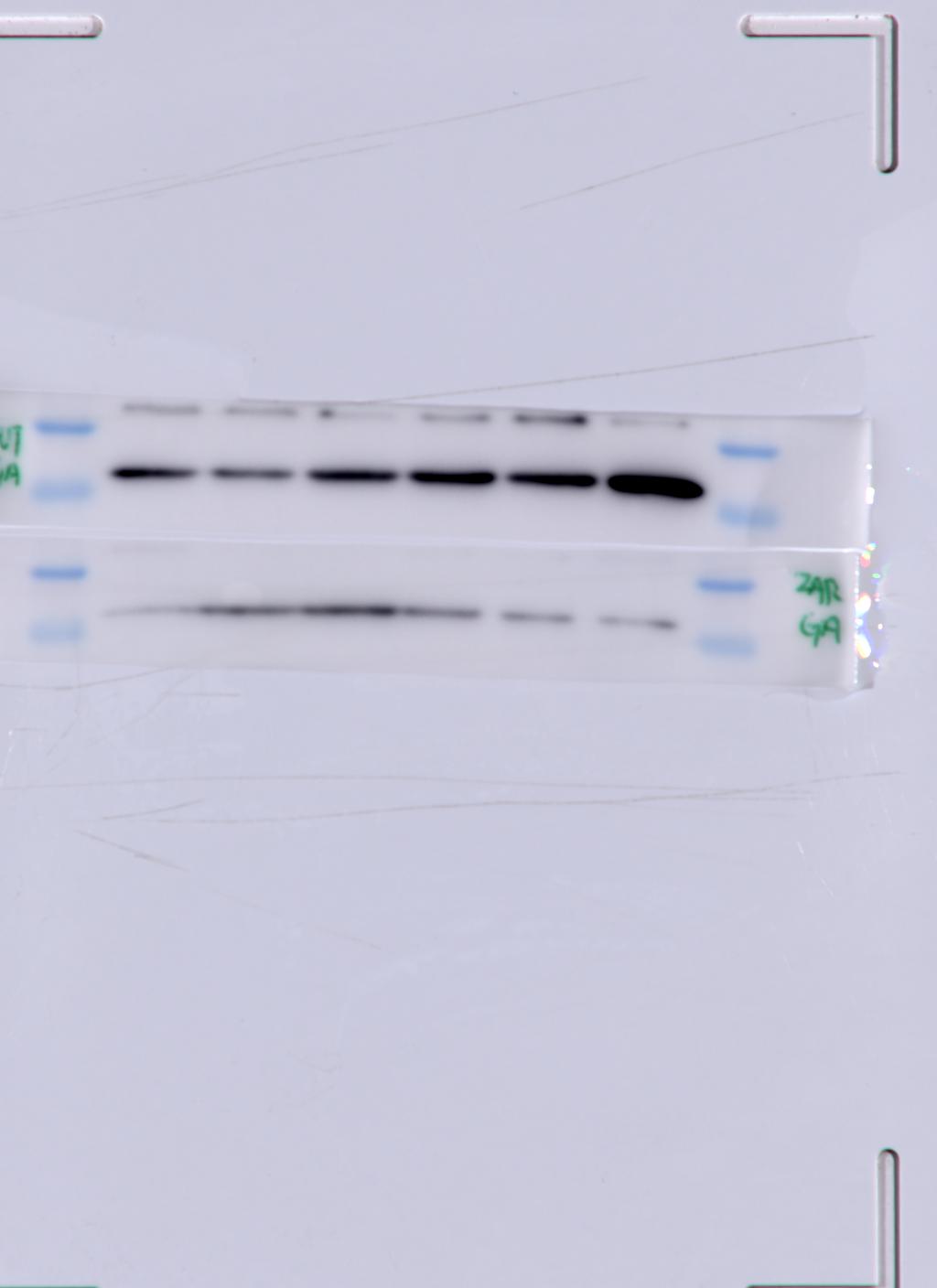

Supplement: Supplementary file 2 — Additional file 2. Raw data of western blot. [file 12974_2022_2632_MOESM2_ESM.zip › supplementary files/Figure2-2 WB/gap43 new.10_Ch/GAP43 2020.09.18_12.28.33_Ch+Marker.jpg]

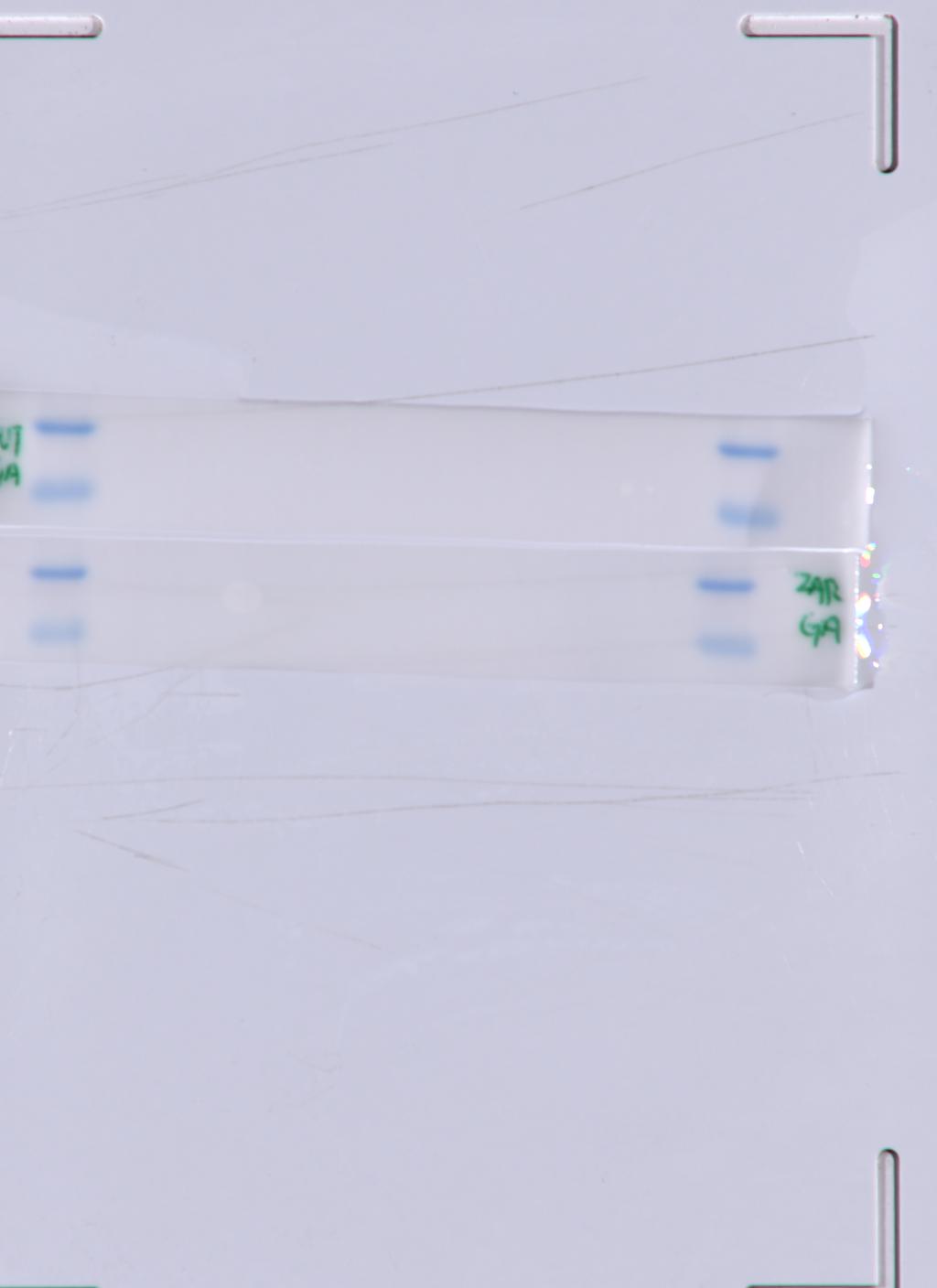

Supplement: Supplementary file 2 — Additional file 2. Raw data of western blot. [file 12974_2022_2632_MOESM2_ESM.zip › supplementary files/Figure2-2 WB/gap43 new.10_Ch/GAP43 2020.09.18_12.28.33_Ch-Marker.jpg]

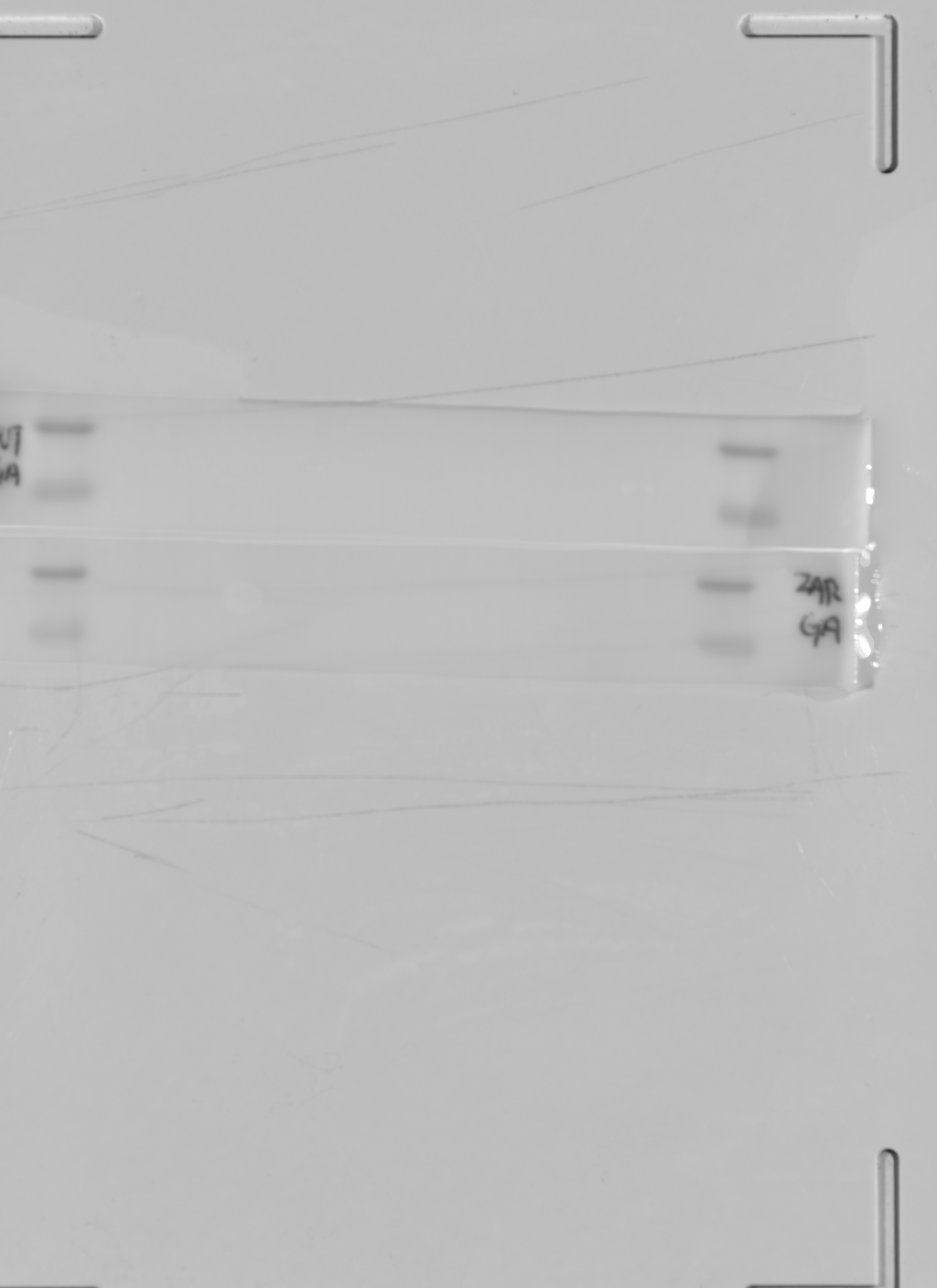

Supplement: Supplementary file 2 — Additional file 2. Raw data of western blot. [file 12974_2022_2632_MOESM2_ESM.zip › supplementary files/Figure2-2 WB/gap43 new.10_Ch/GAP43 2020.09.18_12.28.33_Ch-Marker.tif]

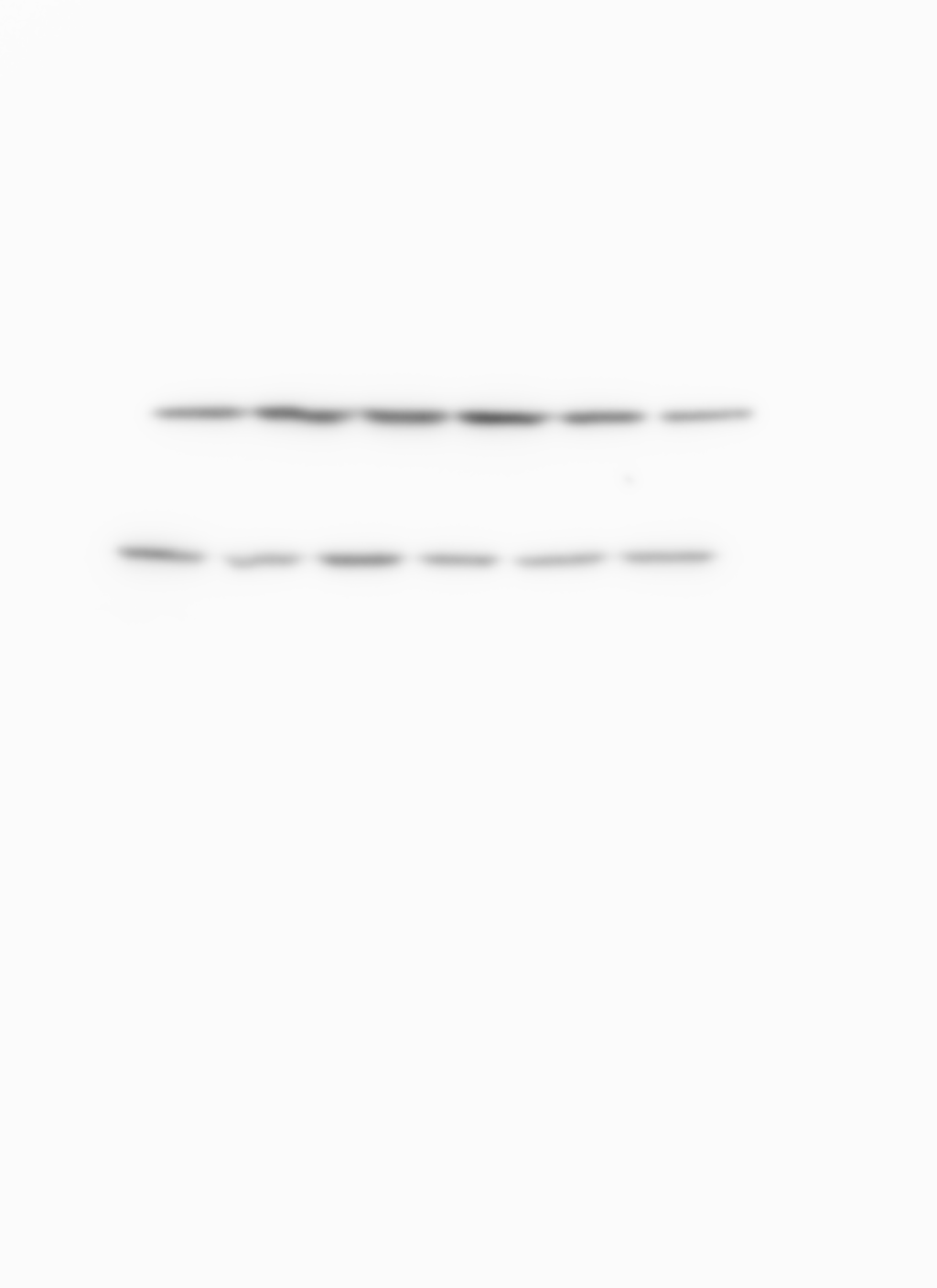

Supplement: Supplementary file 2 — Additional file 2. Raw data of western blot. [file 12974_2022_2632_MOESM2_ESM.zip › supplementary files/Figure2-2 WB/GAP43/gap4 2020.09.20_18.42.56_Ch.tif]

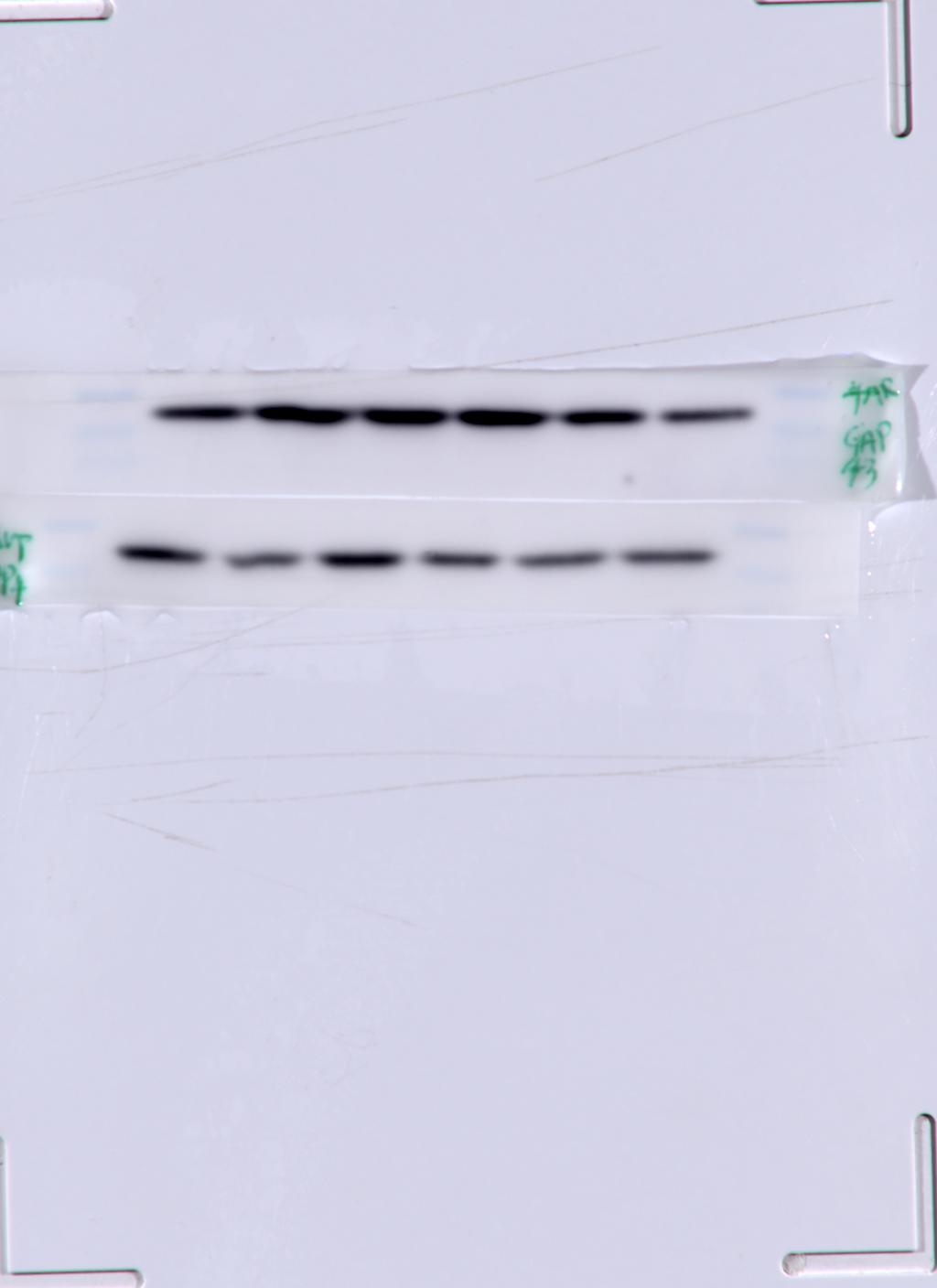

Supplement: Supplementary file 2 — Additional file 2. Raw data of western blot. [file 12974_2022_2632_MOESM2_ESM.zip › supplementary files/Figure2-2 WB/GAP43/gap4 2020.09.20_18.42.56_Ch+Marker.jpg]

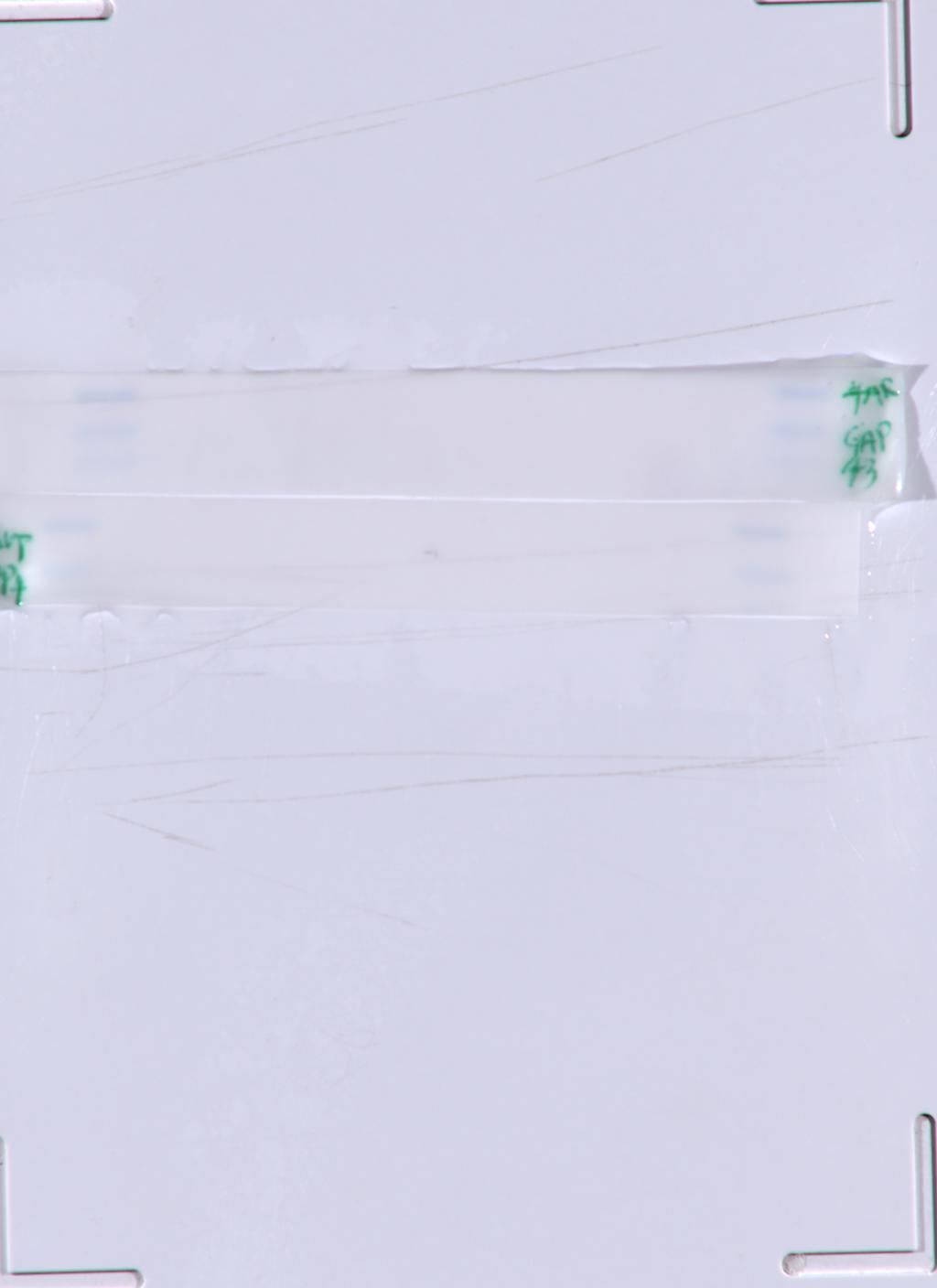

Supplement: Supplementary file 2 — Additional file 2. Raw data of western blot. [file 12974_2022_2632_MOESM2_ESM.zip › supplementary files/Figure2-2 WB/GAP43/gap4 2020.09.20_18.42.56_Ch-Marker.jpg]

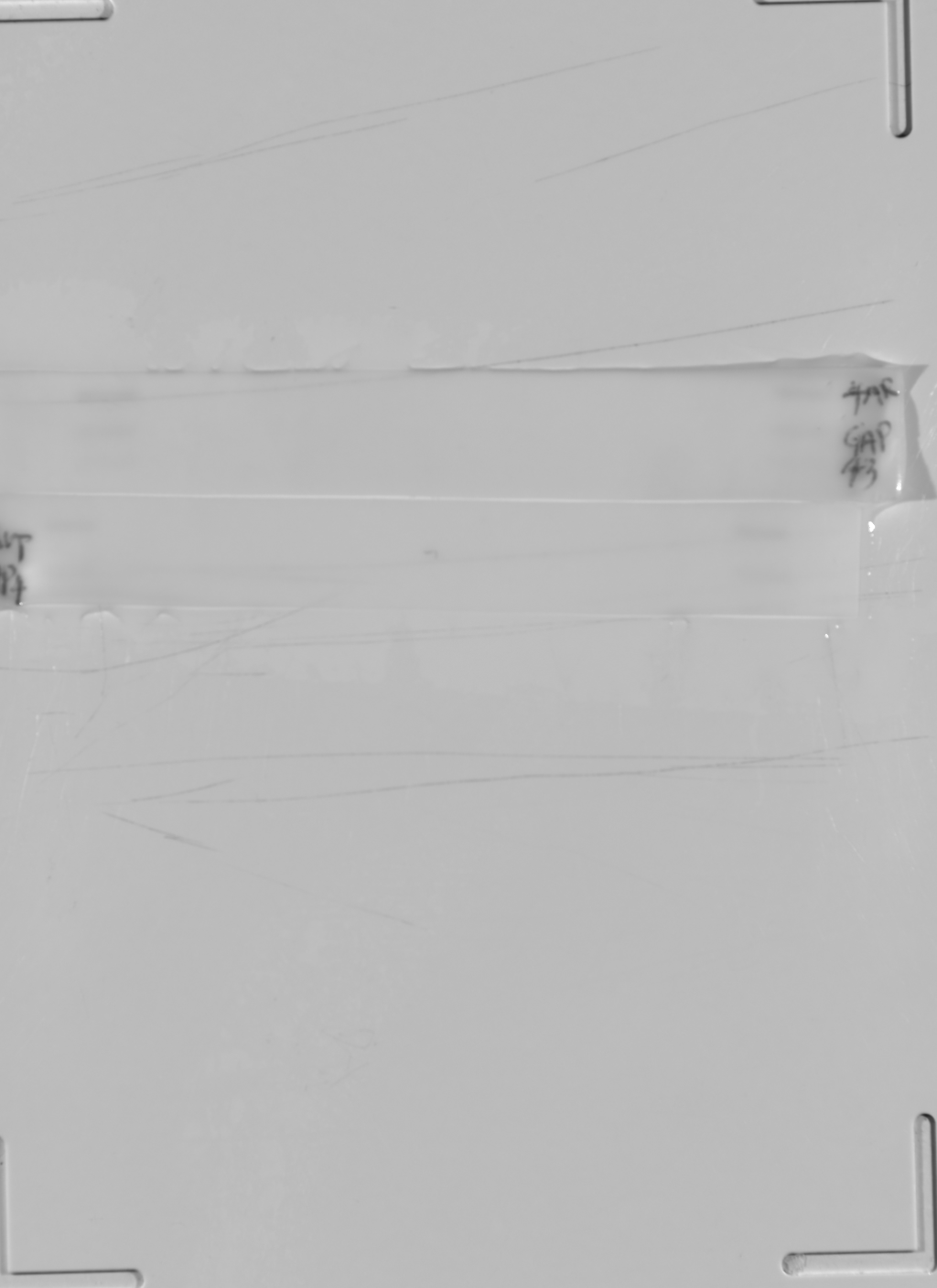

Supplement: Supplementary file 2 — Additional file 2. Raw data of western blot. [file 12974_2022_2632_MOESM2_ESM.zip › supplementary files/Figure2-2 WB/GAP43/gap4 2020.09.20_18.42.56_Ch-Marker.tif]

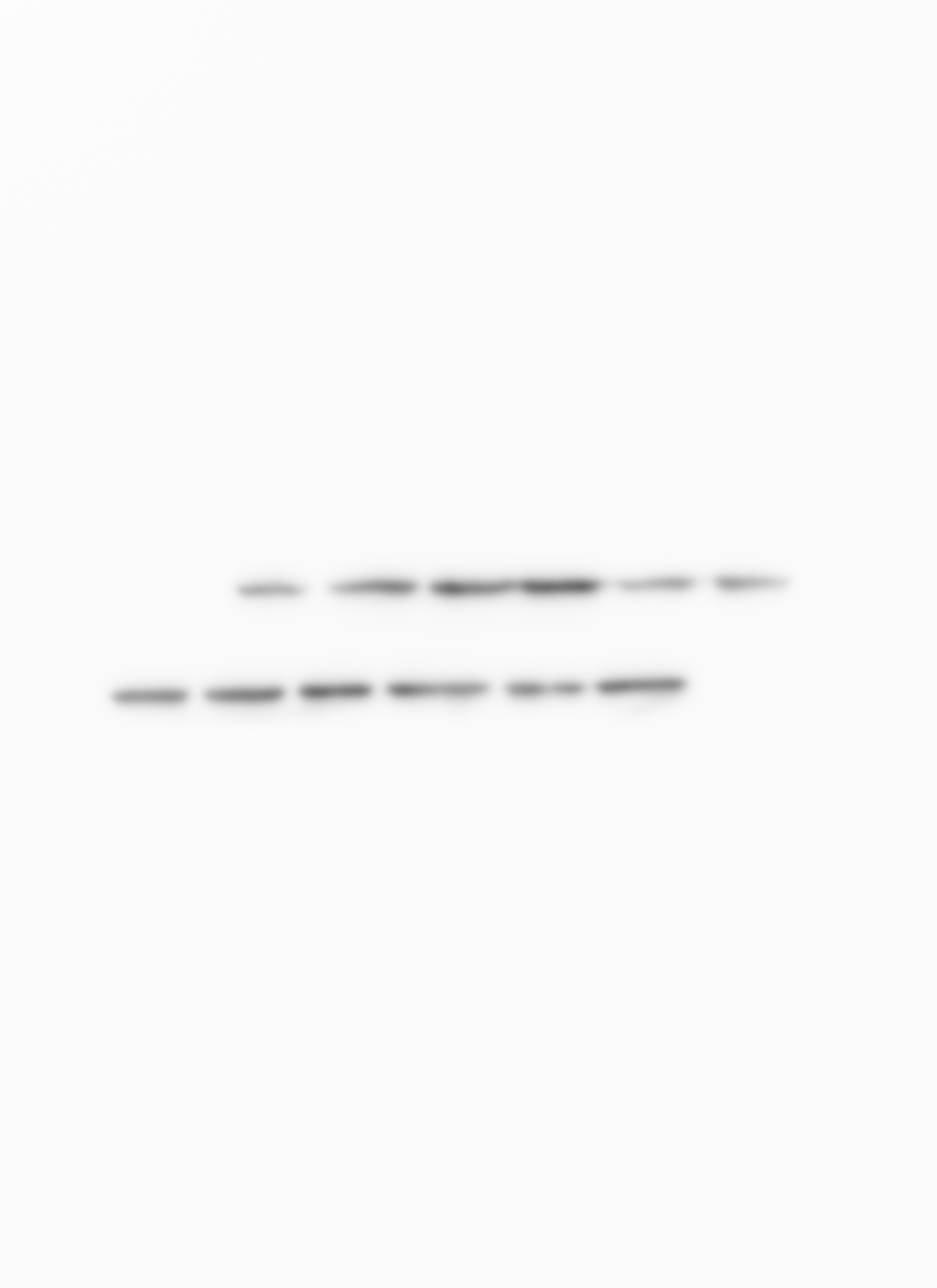

Supplement: Supplementary file 2 — Additional file 2. Raw data of western blot. [file 12974_2022_2632_MOESM2_ESM.zip › supplementary files/Figure2-2 WB/gapdh new.24_Ch/gapdh 2020.09.10_17.55.24_Ch.tif]

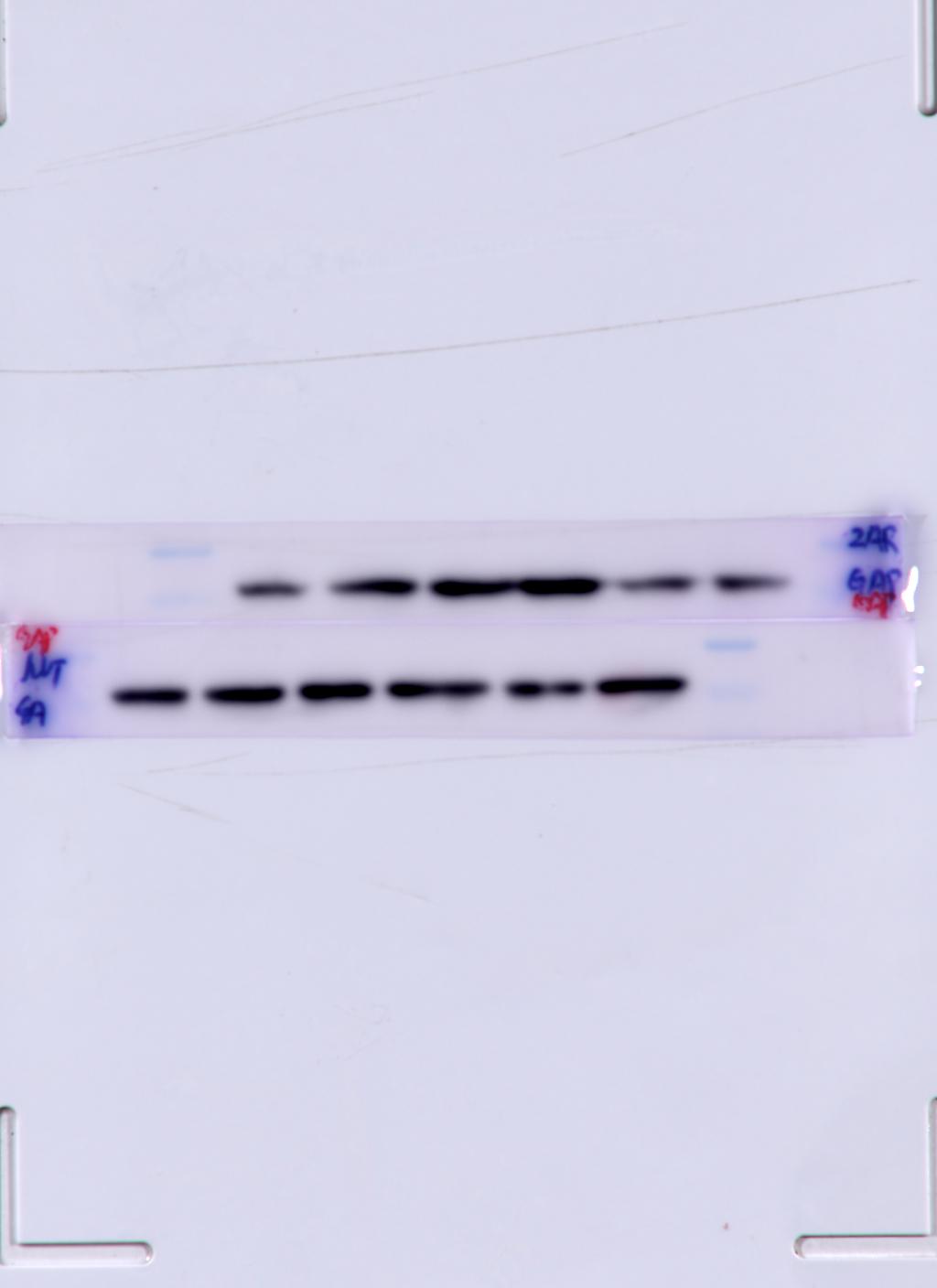

Supplement: Supplementary file 2 — Additional file 2. Raw data of western blot. [file 12974_2022_2632_MOESM2_ESM.zip › supplementary files/Figure2-2 WB/gapdh new.24_Ch/gapdh 2020.09.10_17.55.24_Ch+Marker.jpg]

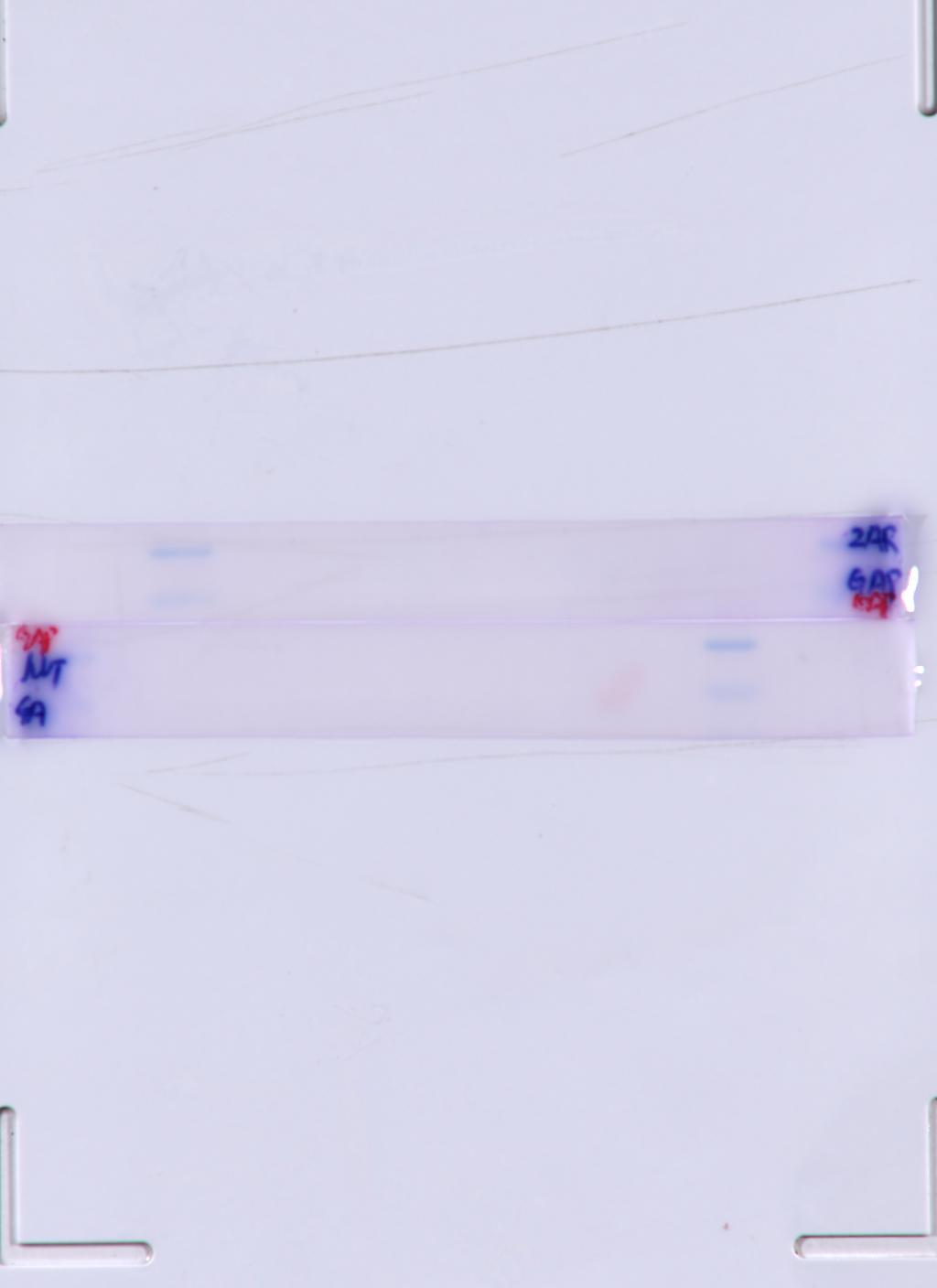

Supplement: Supplementary file 2 — Additional file 2. Raw data of western blot. [file 12974_2022_2632_MOESM2_ESM.zip › supplementary files/Figure2-2 WB/gapdh new.24_Ch/gapdh 2020.09.10_17.55.24_Ch-Marker.jpg]

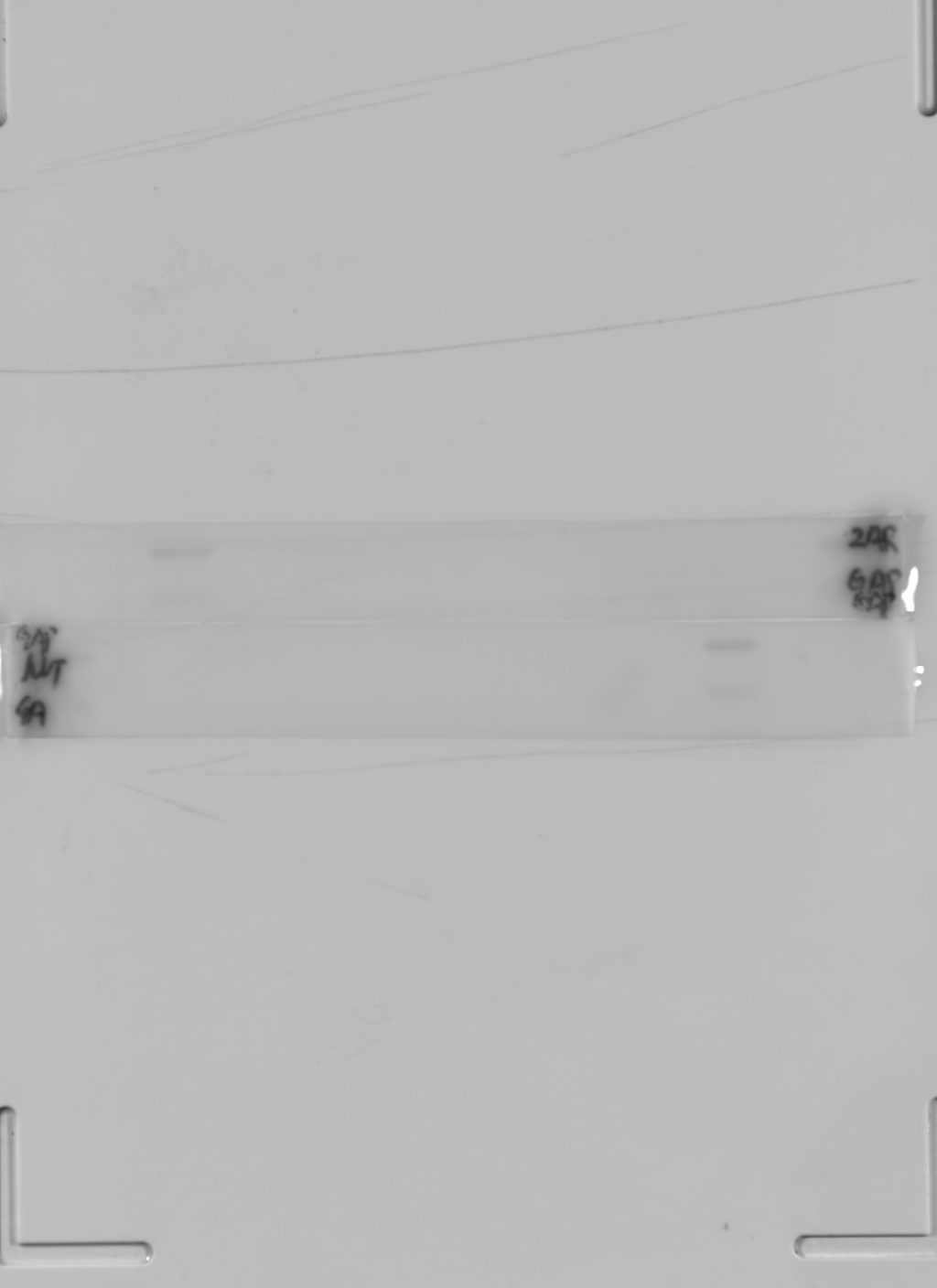

Supplement: Supplementary file 2 — Additional file 2. Raw data of western blot. [file 12974_2022_2632_MOESM2_ESM.zip › supplementary files/Figure2-2 WB/gapdh new.24_Ch/gapdh 2020.09.10_17.55.24_Ch-Marker.tif]

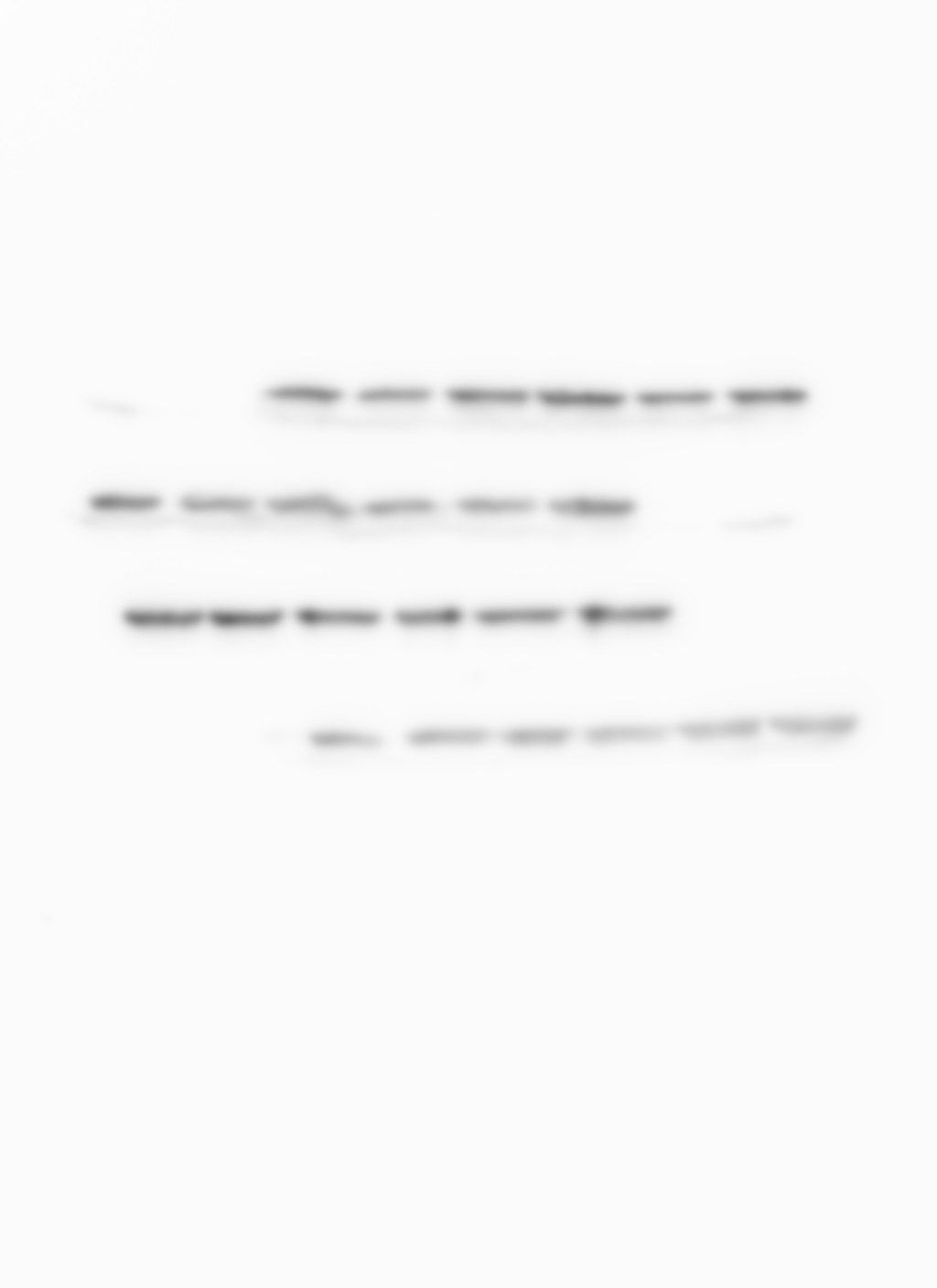

Supplement: Supplementary file 2 — Additional file 2. Raw data of western blot. [file 12974_2022_2632_MOESM2_ESM.zip › supplementary files/Figure2-2 WB/gapdh new.24_Ch/gapdh 2020.09.13_13.37.44_Ch.tif]

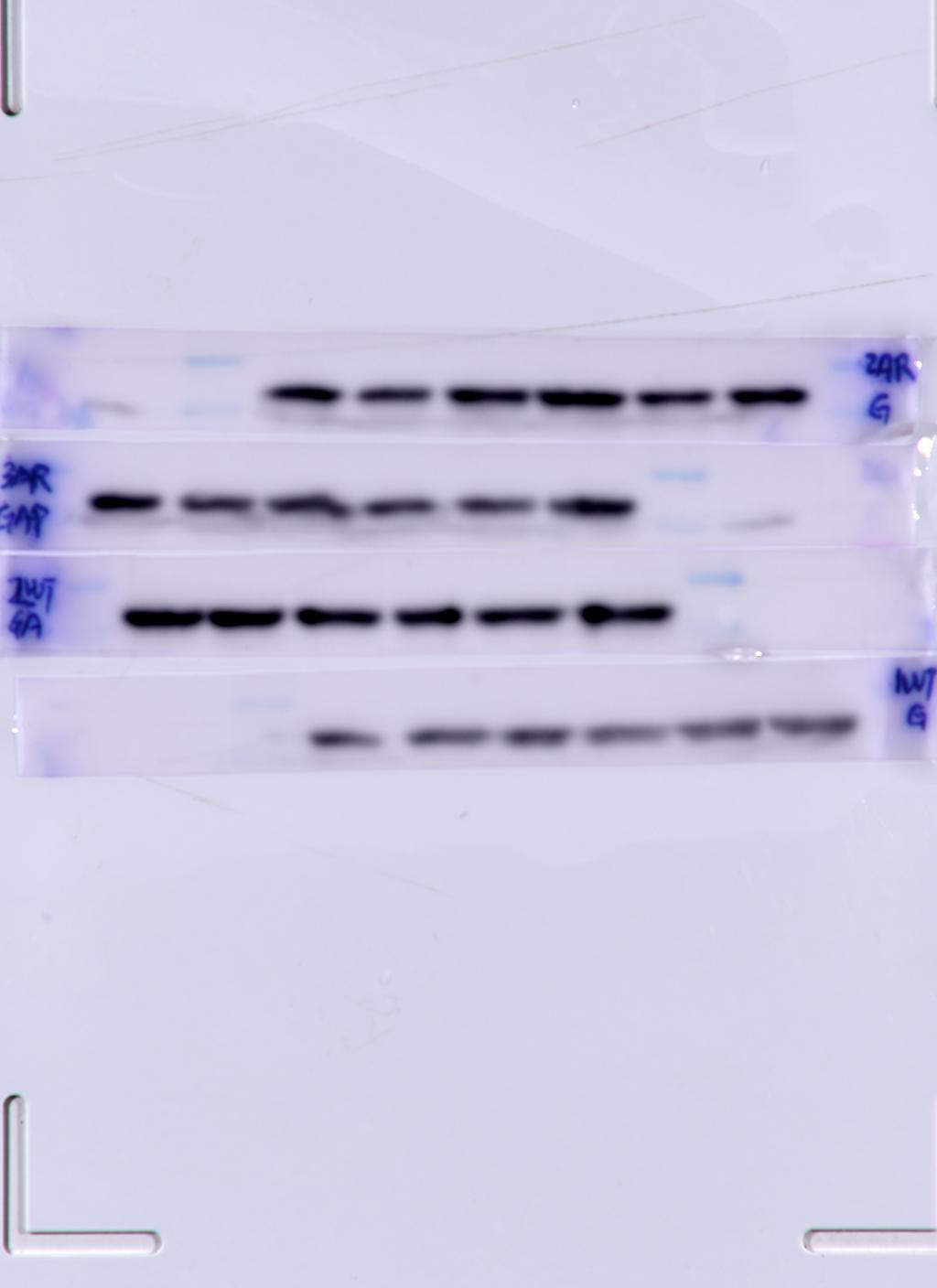

Supplement: Supplementary file 2 — Additional file 2. Raw data of western blot. [file 12974_2022_2632_MOESM2_ESM.zip › supplementary files/Figure2-2 WB/gapdh new.24_Ch/gapdh 2020.09.13_13.37.44_Ch+Marker.jpg]

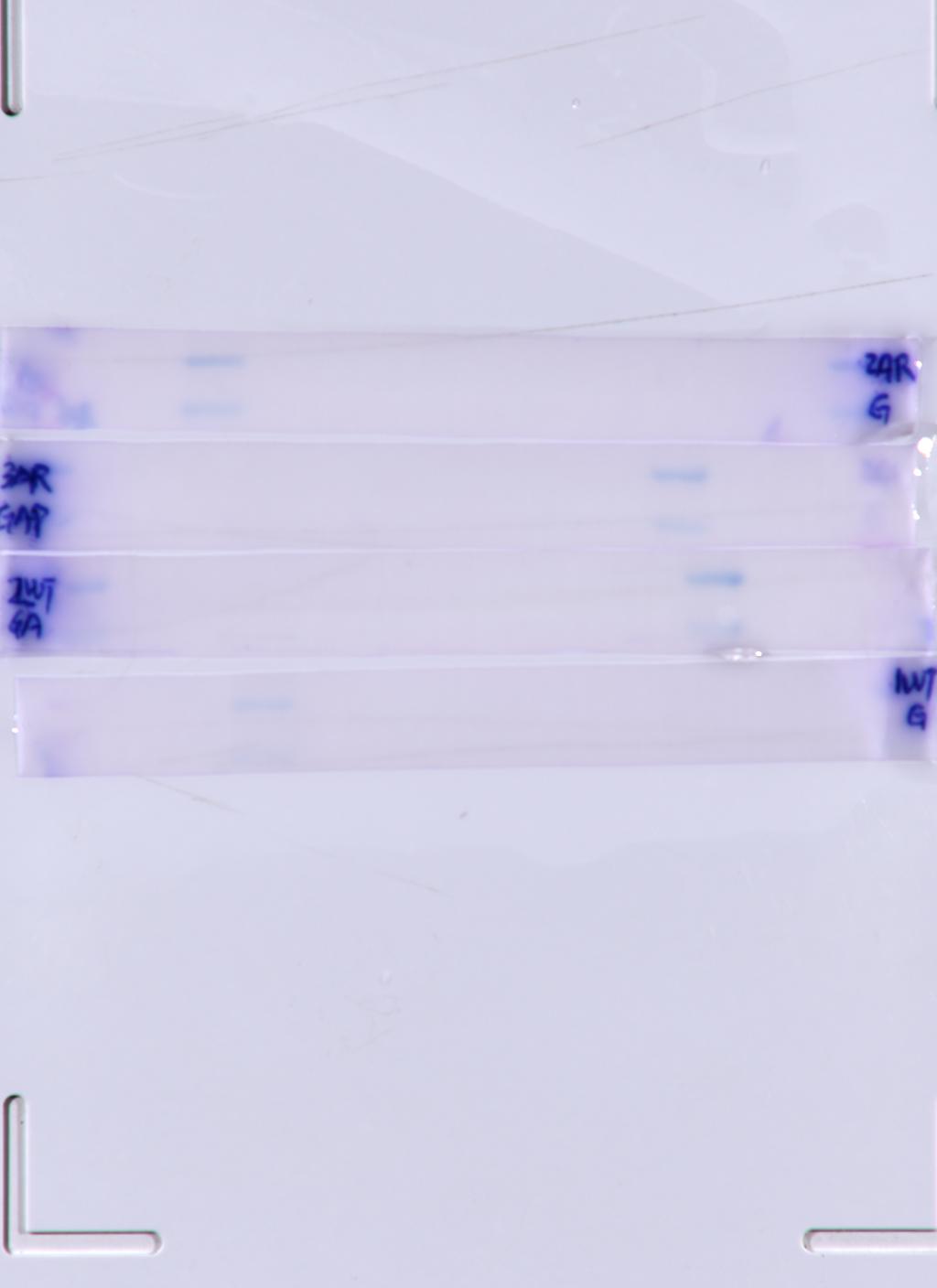

Supplement: Supplementary file 2 — Additional file 2. Raw data of western blot. [file 12974_2022_2632_MOESM2_ESM.zip › supplementary files/Figure2-2 WB/gapdh new.24_Ch/gapdh 2020.09.13_13.37.44_Ch-Marker.jpg]

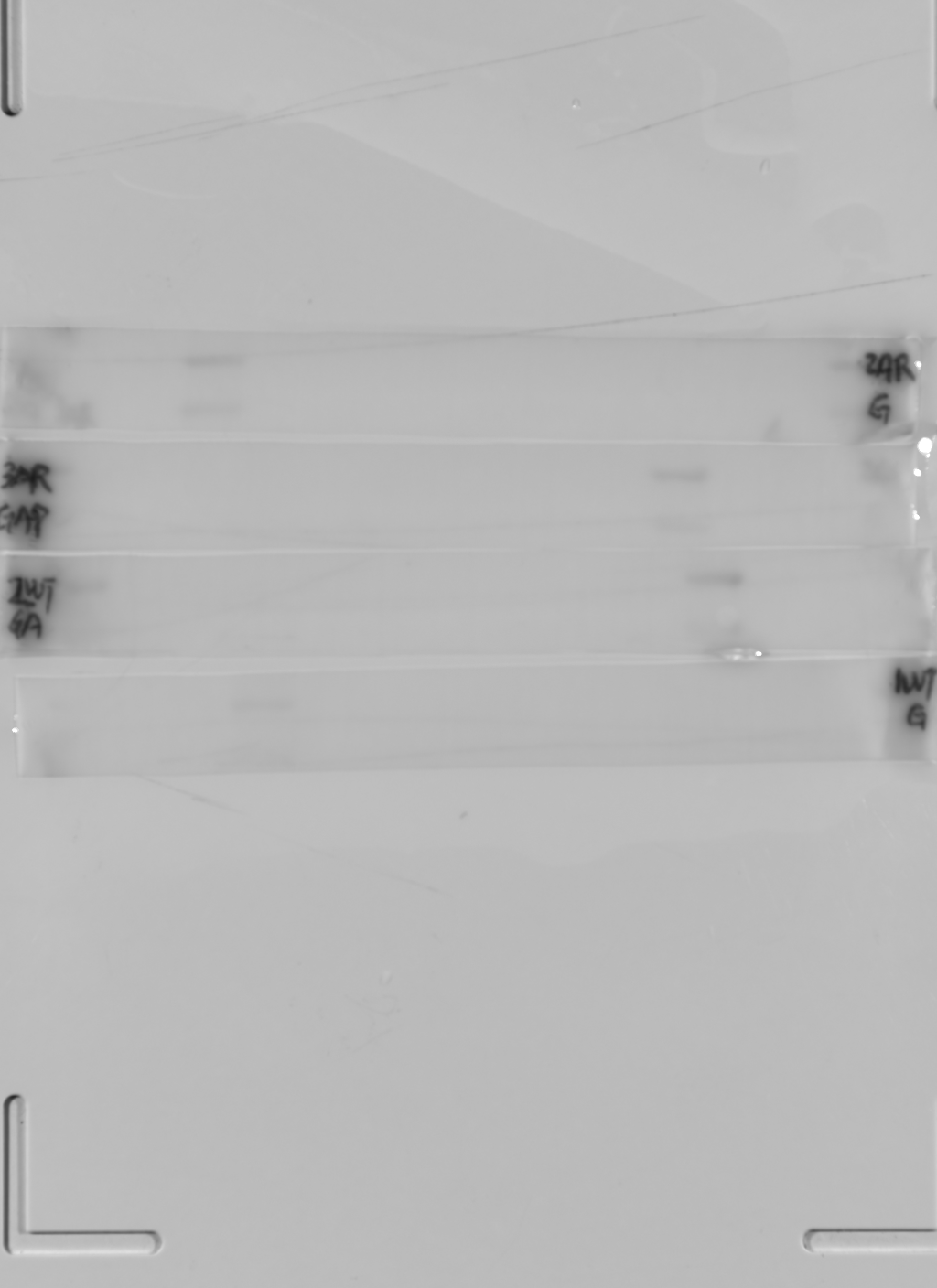

Supplement: Supplementary file 2 — Additional file 2. Raw data of western blot. [file 12974_2022_2632_MOESM2_ESM.zip › supplementary files/Figure2-2 WB/gapdh new.24_Ch/gapdh 2020.09.13_13.37.44_Ch-Marker.tif]

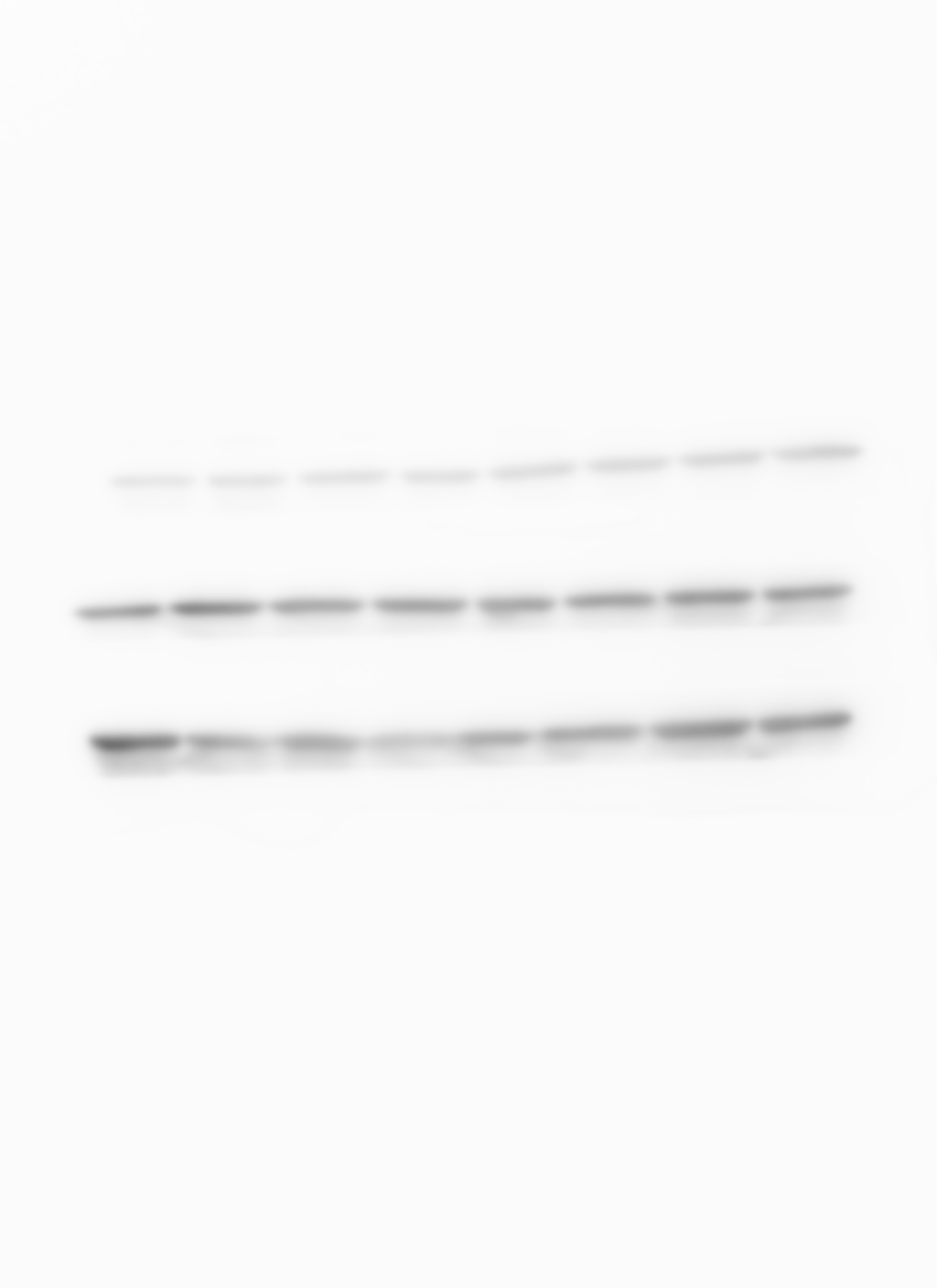

Supplement: Supplementary file 2 — Additional file 2. Raw data of western blot. [file 12974_2022_2632_MOESM2_ESM.zip › supplementary files/Figure2-2 WB/GAPDH/AR GAPDH/gapdh 2020.08.18_16.34.27_Ch.tif]

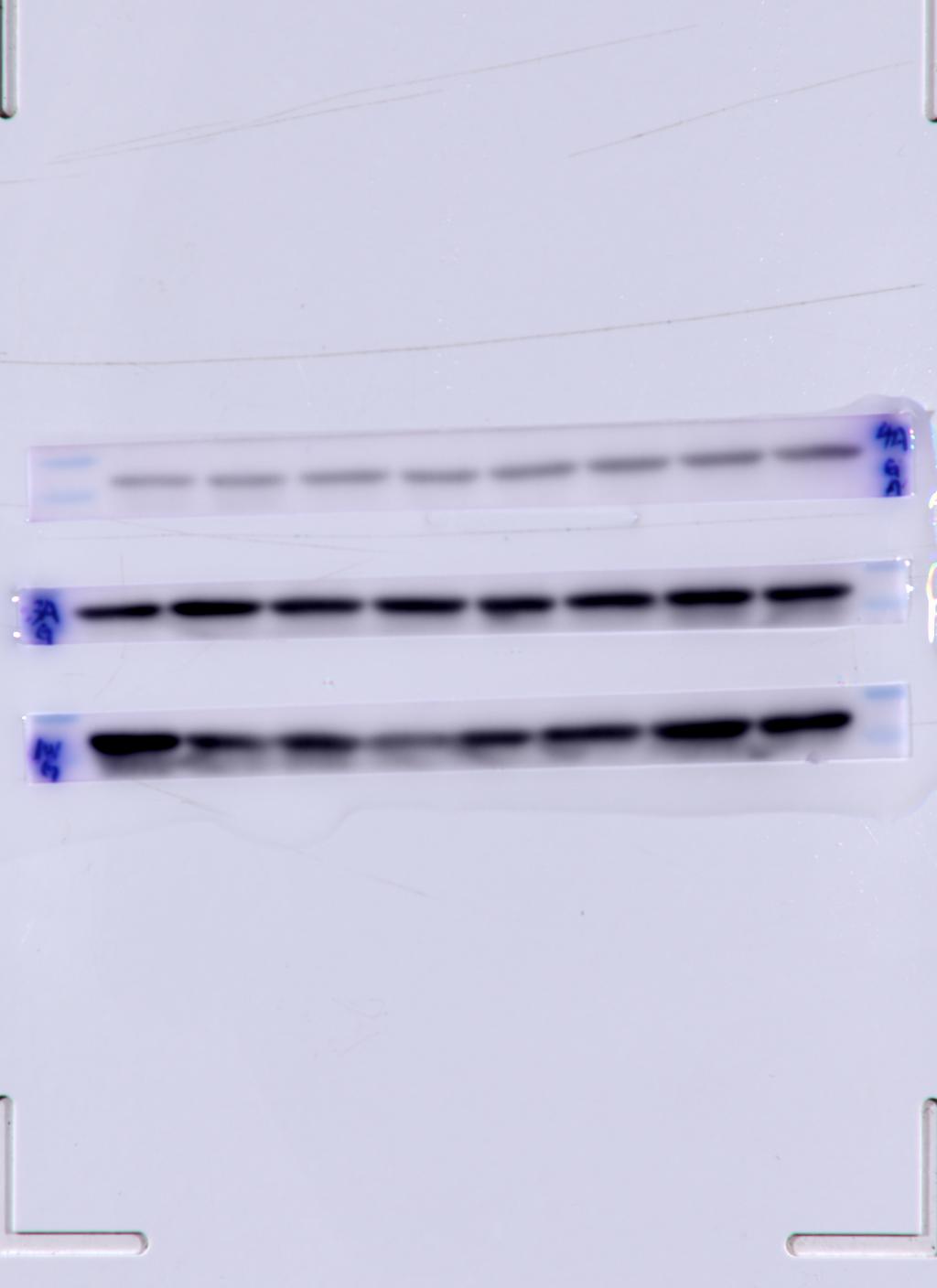

Supplement: Supplementary file 2 — Additional file 2. Raw data of western blot. [file 12974_2022_2632_MOESM2_ESM.zip › supplementary files/Figure2-2 WB/GAPDH/AR GAPDH/gapdh 2020.08.18_16.34.27_Ch+Marker.jpg]

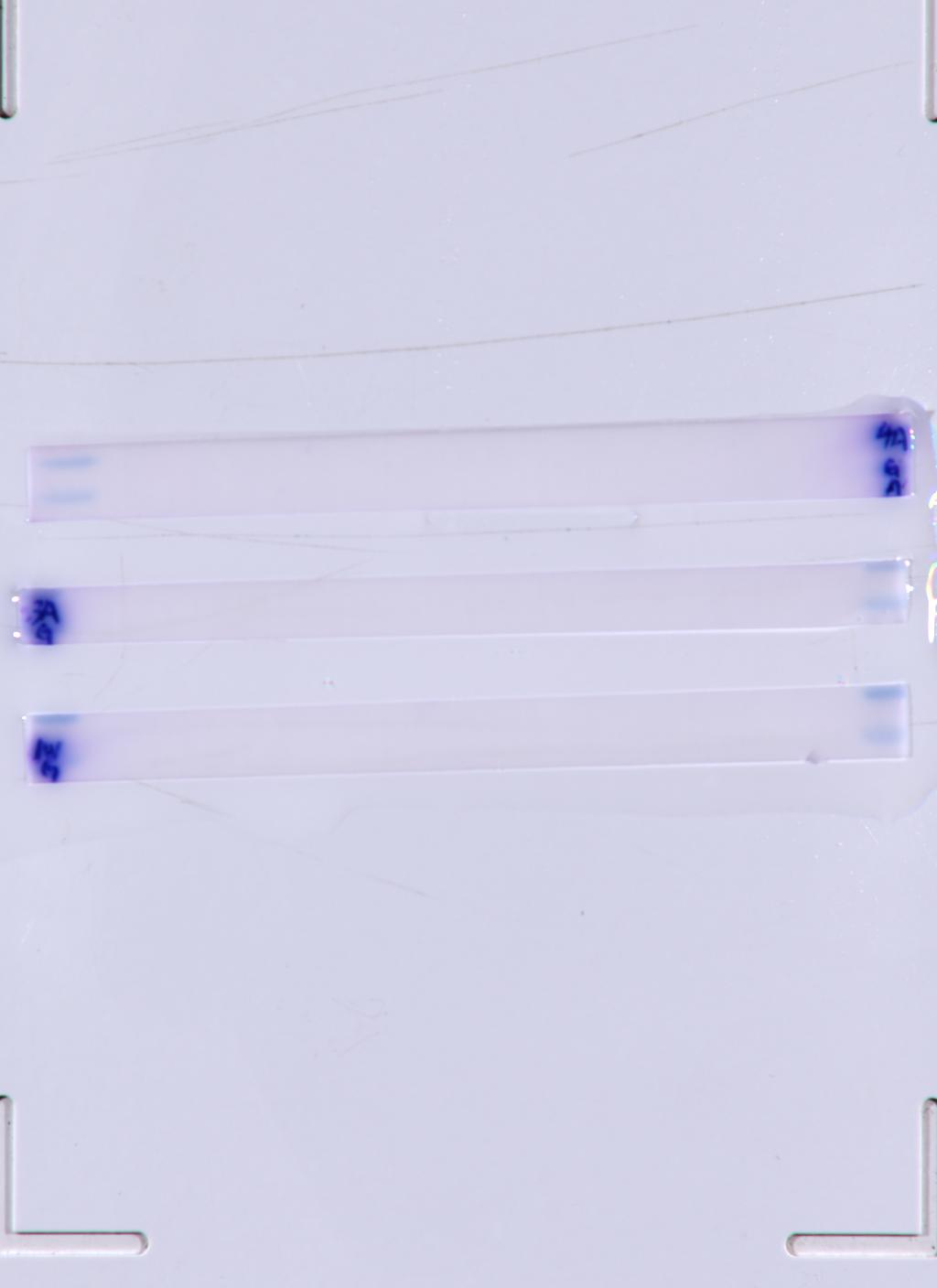

Supplement: Supplementary file 2 — Additional file 2. Raw data of western blot. [file 12974_2022_2632_MOESM2_ESM.zip › supplementary files/Figure2-2 WB/GAPDH/AR GAPDH/gapdh 2020.08.18_16.34.27_Ch-Marker.jpg]

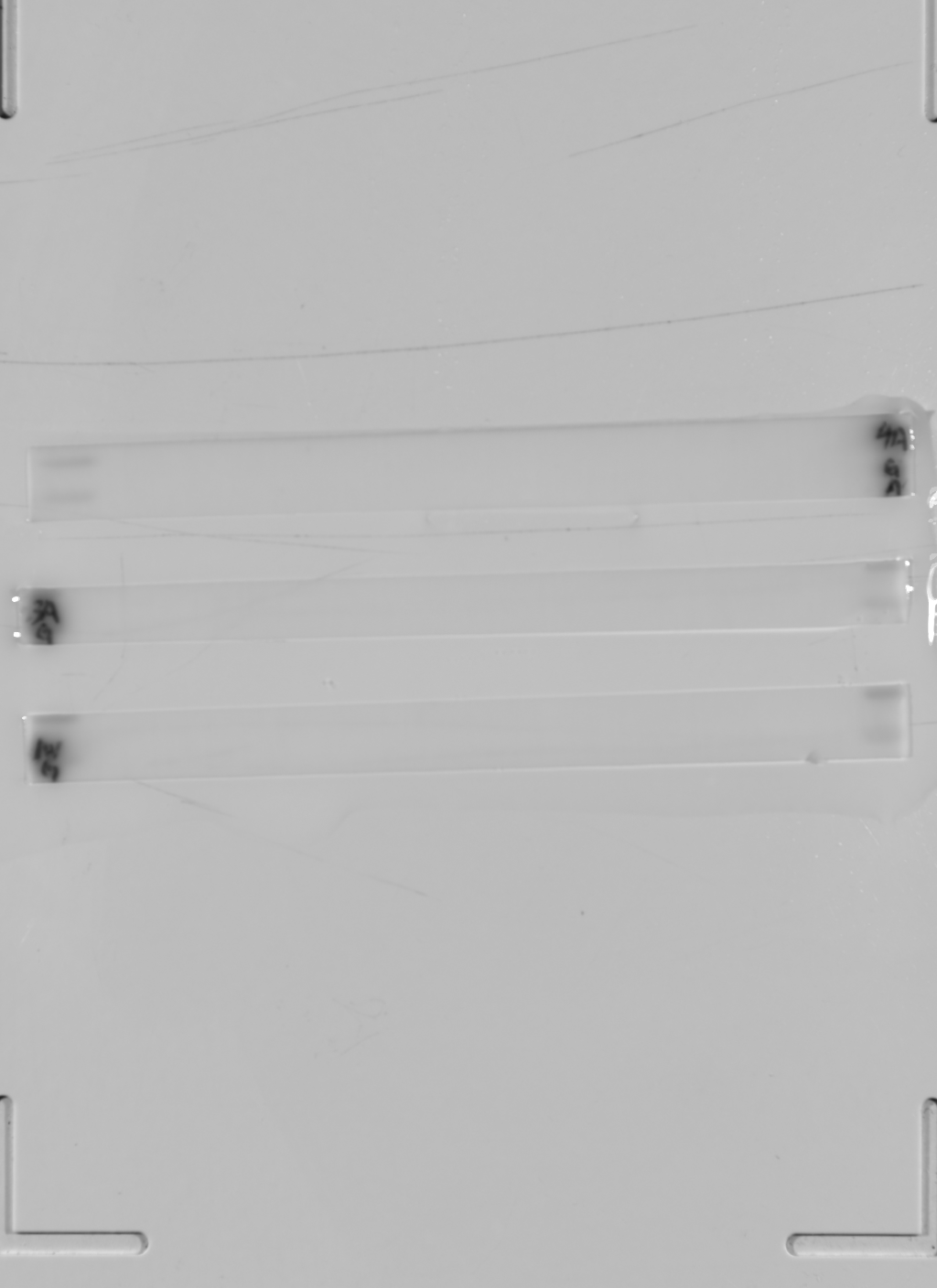

Supplement: Supplementary file 2 — Additional file 2. Raw data of western blot. [file 12974_2022_2632_MOESM2_ESM.zip › supplementary files/Figure2-2 WB/GAPDH/AR GAPDH/gapdh 2020.08.18_16.34.27_Ch-Marker.tif]

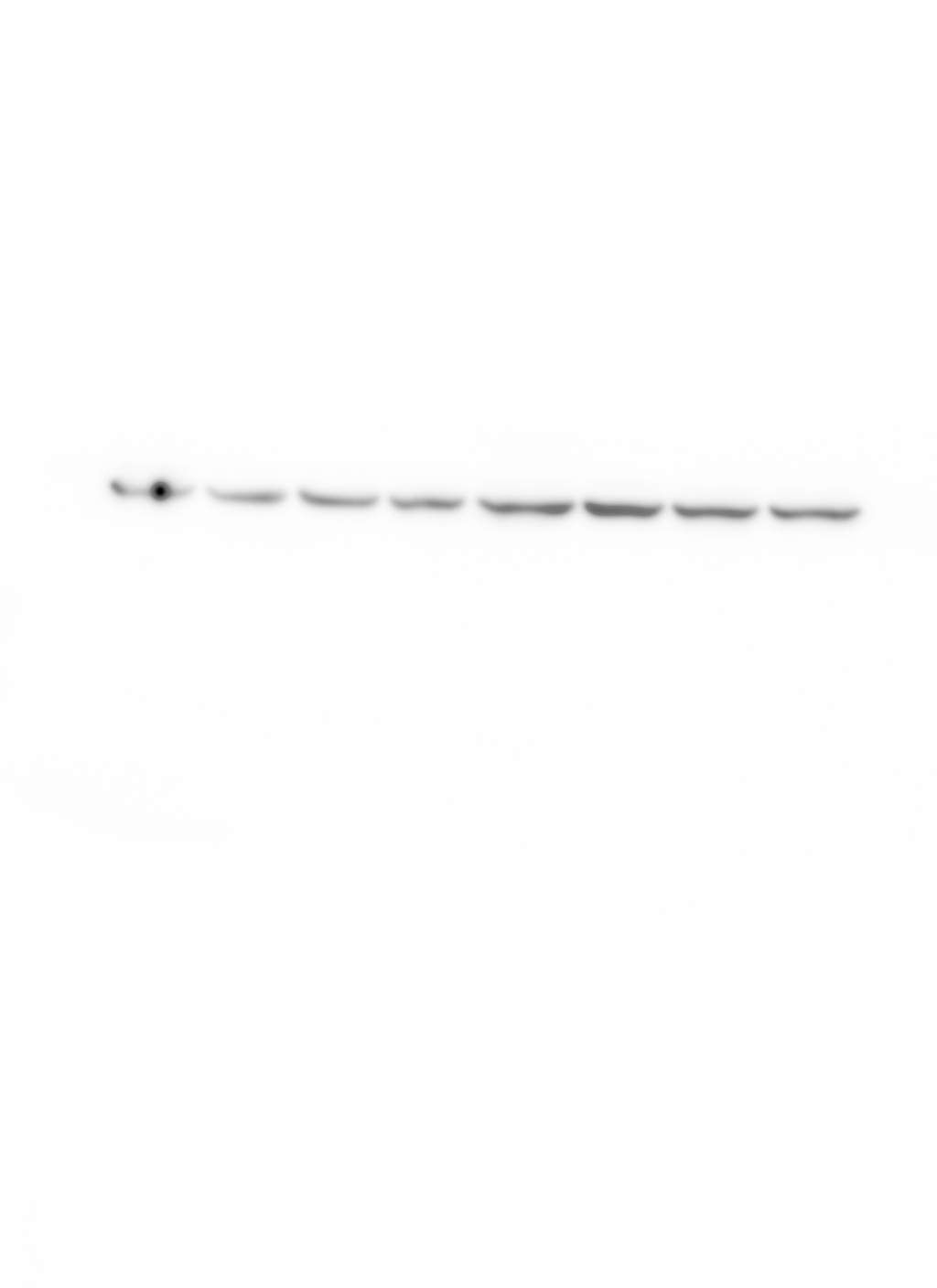

Supplement: Supplementary file 2 — Additional file 2. Raw data of western blot. [file 12974_2022_2632_MOESM2_ESM.zip › supplementary files/Figure2-2 WB/GAPDH/WT GAPDH/betaactin 2020.08.09_18.49.56_Ch.tif]

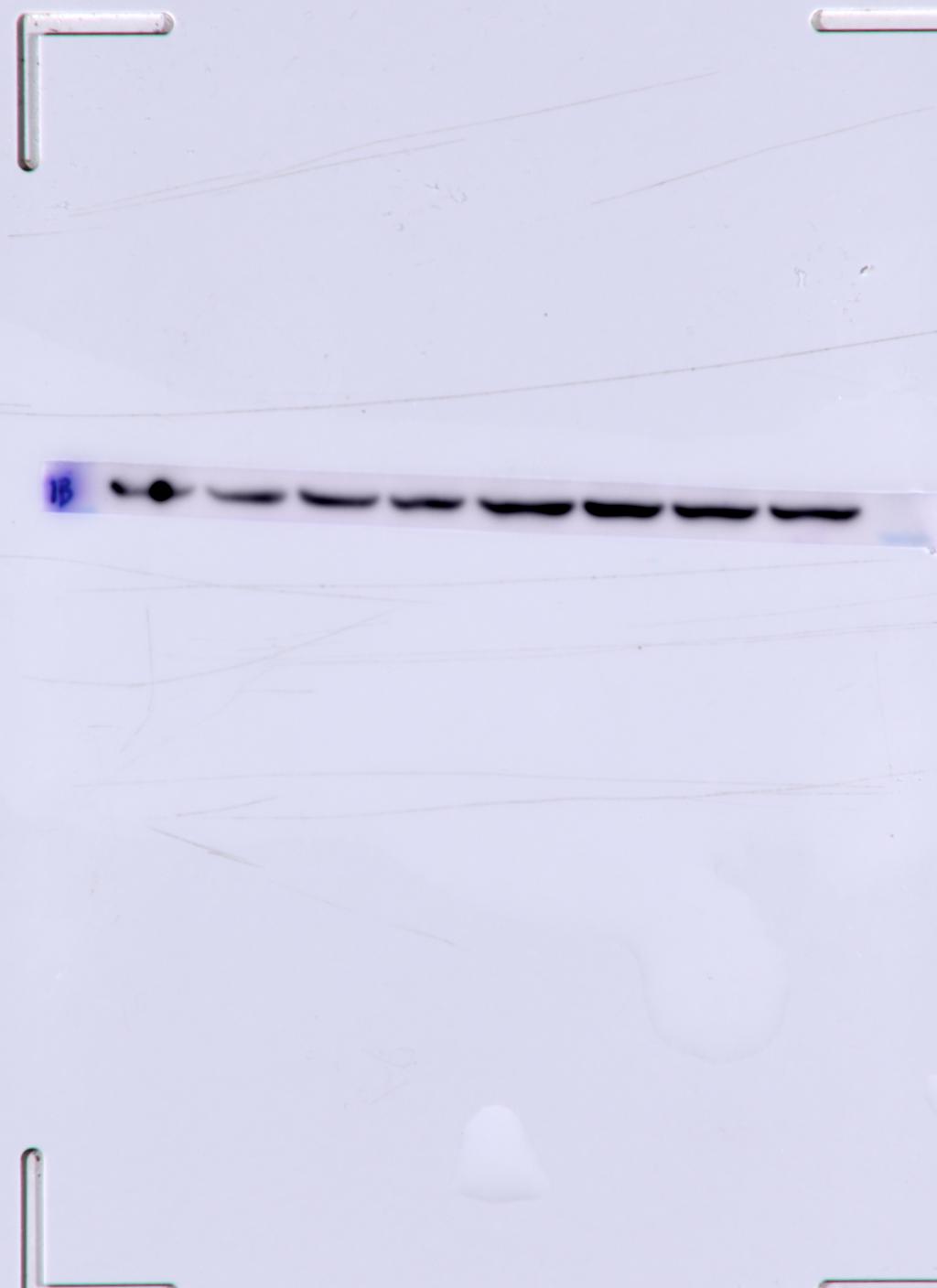

Supplement: Supplementary file 2 — Additional file 2. Raw data of western blot. [file 12974_2022_2632_MOESM2_ESM.zip › supplementary files/Figure2-2 WB/GAPDH/WT GAPDH/betaactin 2020.08.09_18.49.56_Ch+Marker.jpg]

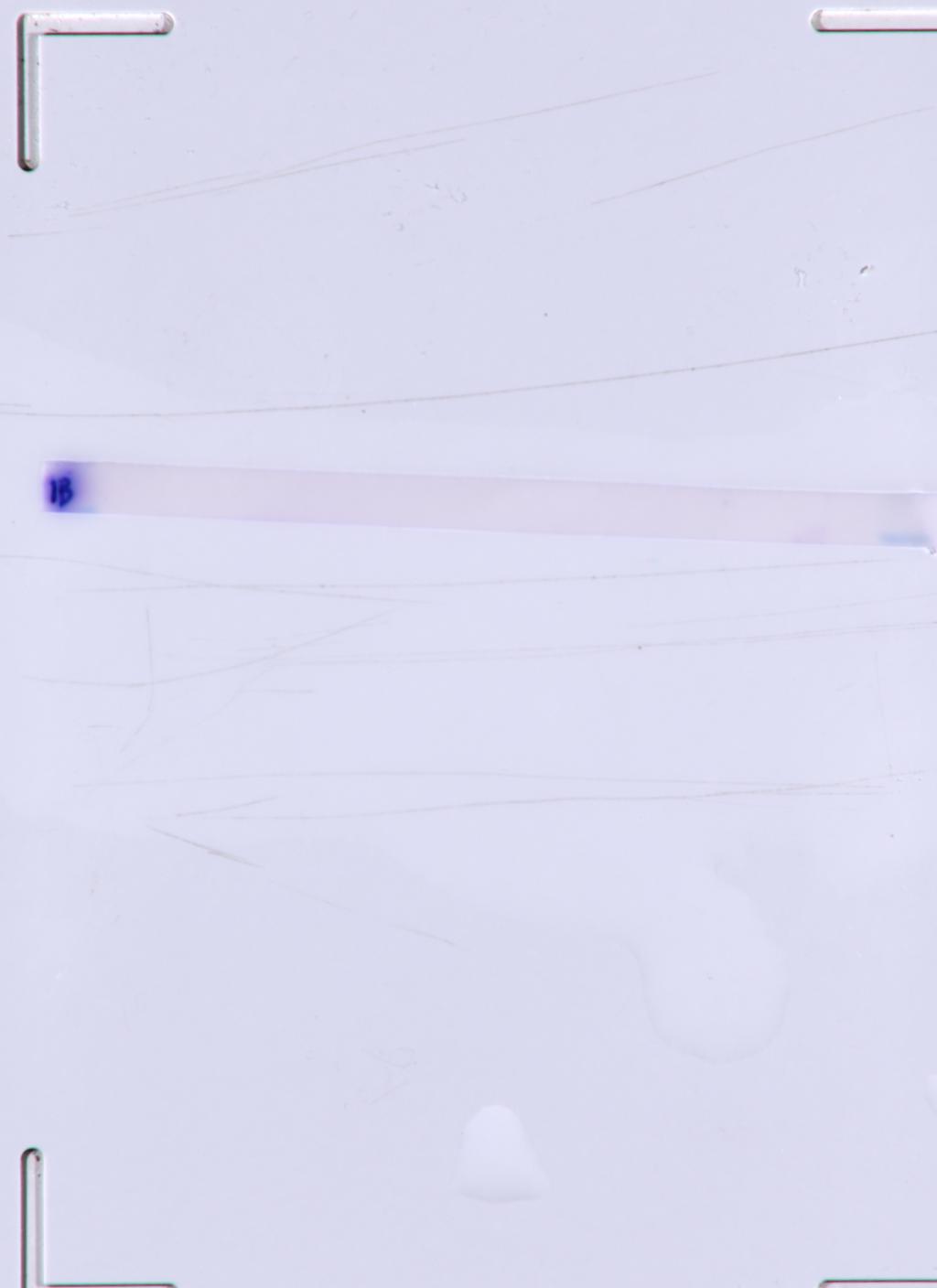

Supplement: Supplementary file 2 — Additional file 2. Raw data of western blot. [file 12974_2022_2632_MOESM2_ESM.zip › supplementary files/Figure2-2 WB/GAPDH/WT GAPDH/betaactin 2020.08.09_18.49.56_Ch-Marker.jpg]

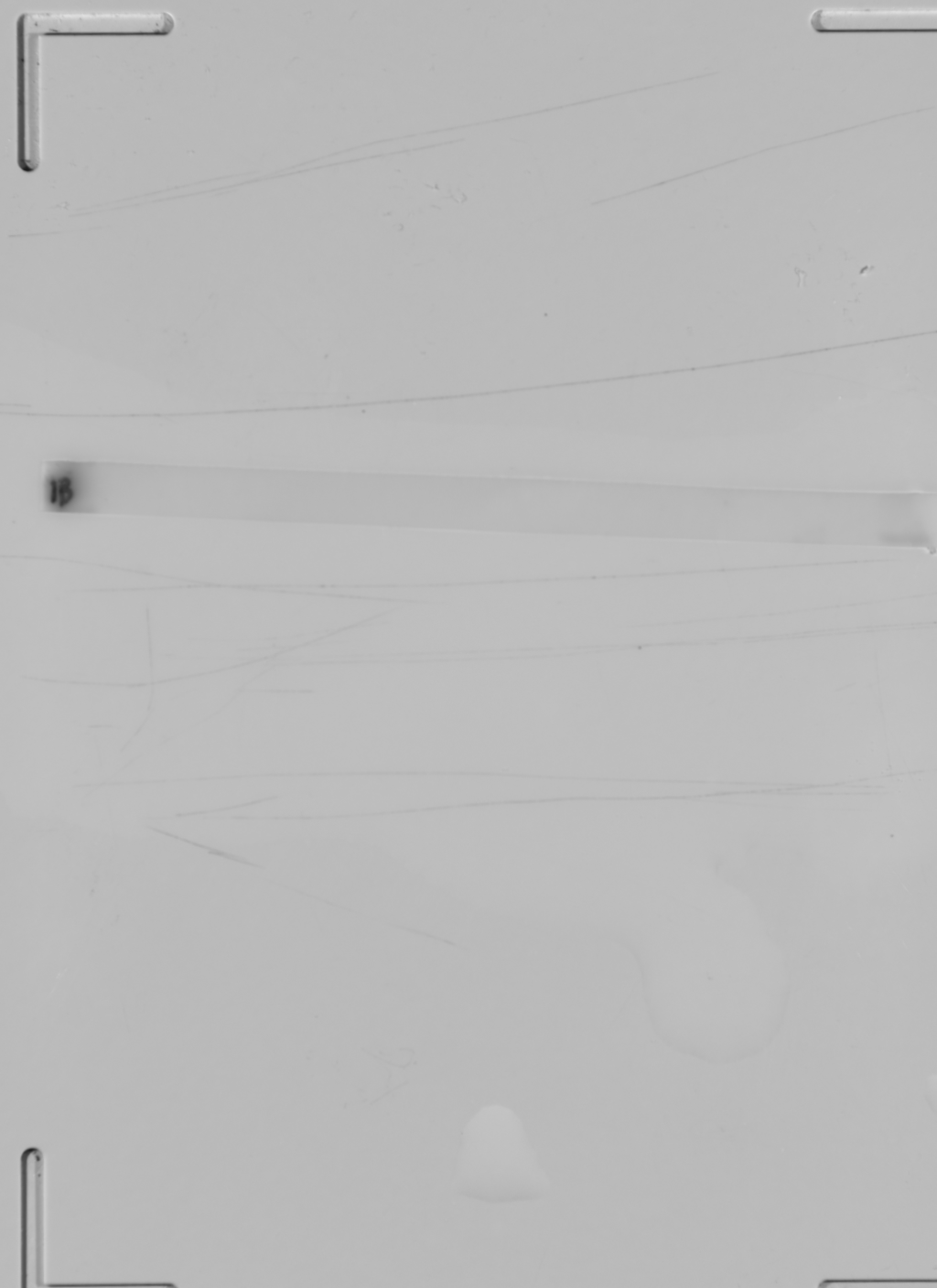

Supplement: Supplementary file 2 — Additional file 2. Raw data of western blot. [file 12974_2022_2632_MOESM2_ESM.zip › supplementary files/Figure2-2 WB/GAPDH/WT GAPDH/betaactin 2020.08.09_18.49.56_Ch-Marker.tif]

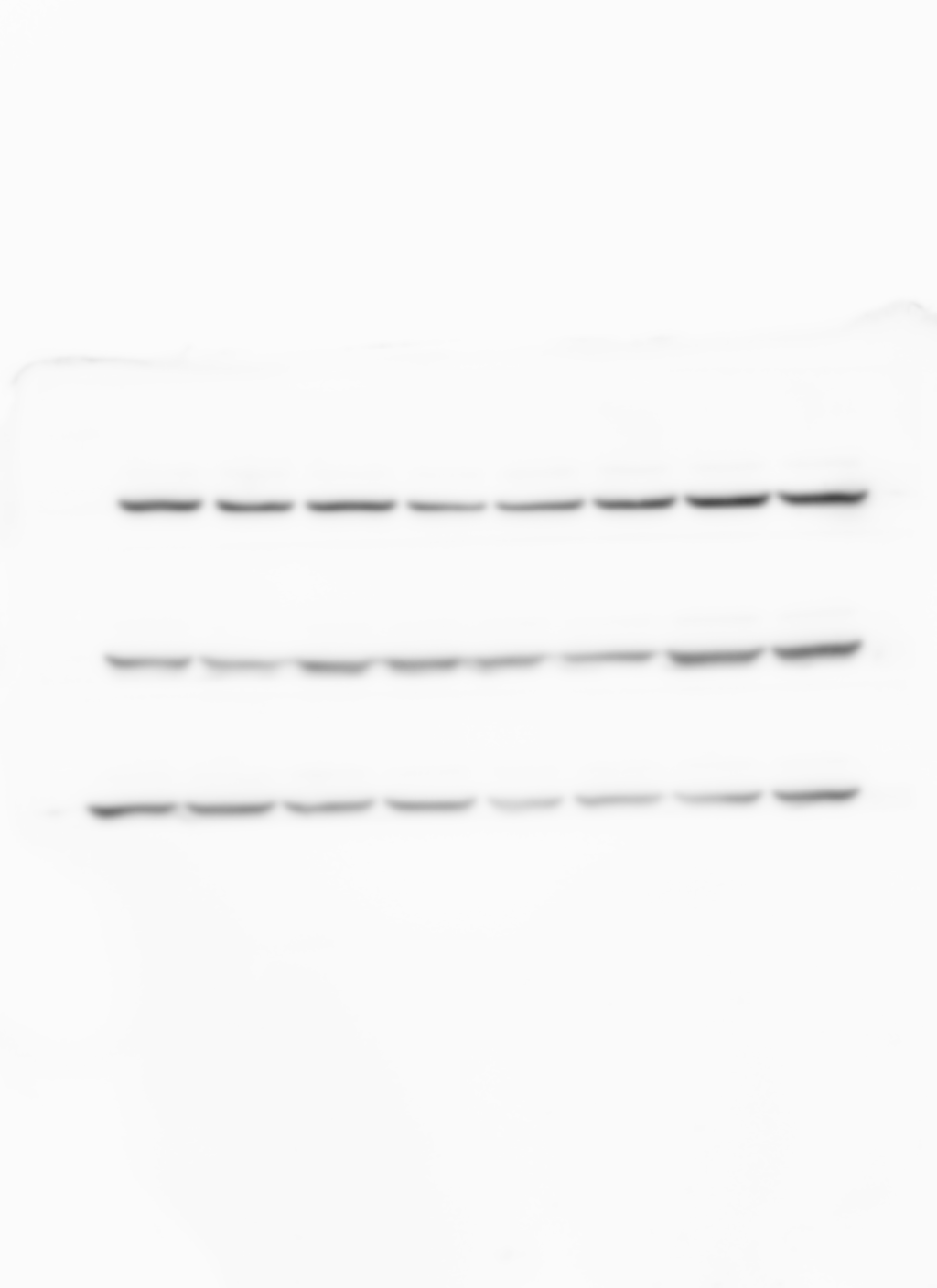

Supplement: Supplementary file 2 — Additional file 2. Raw data of western blot. [file 12974_2022_2632_MOESM2_ESM.zip › supplementary files/Figure3 WB/Beclin new/belin1 2020.08.19_18.02.00_Ch.tif]

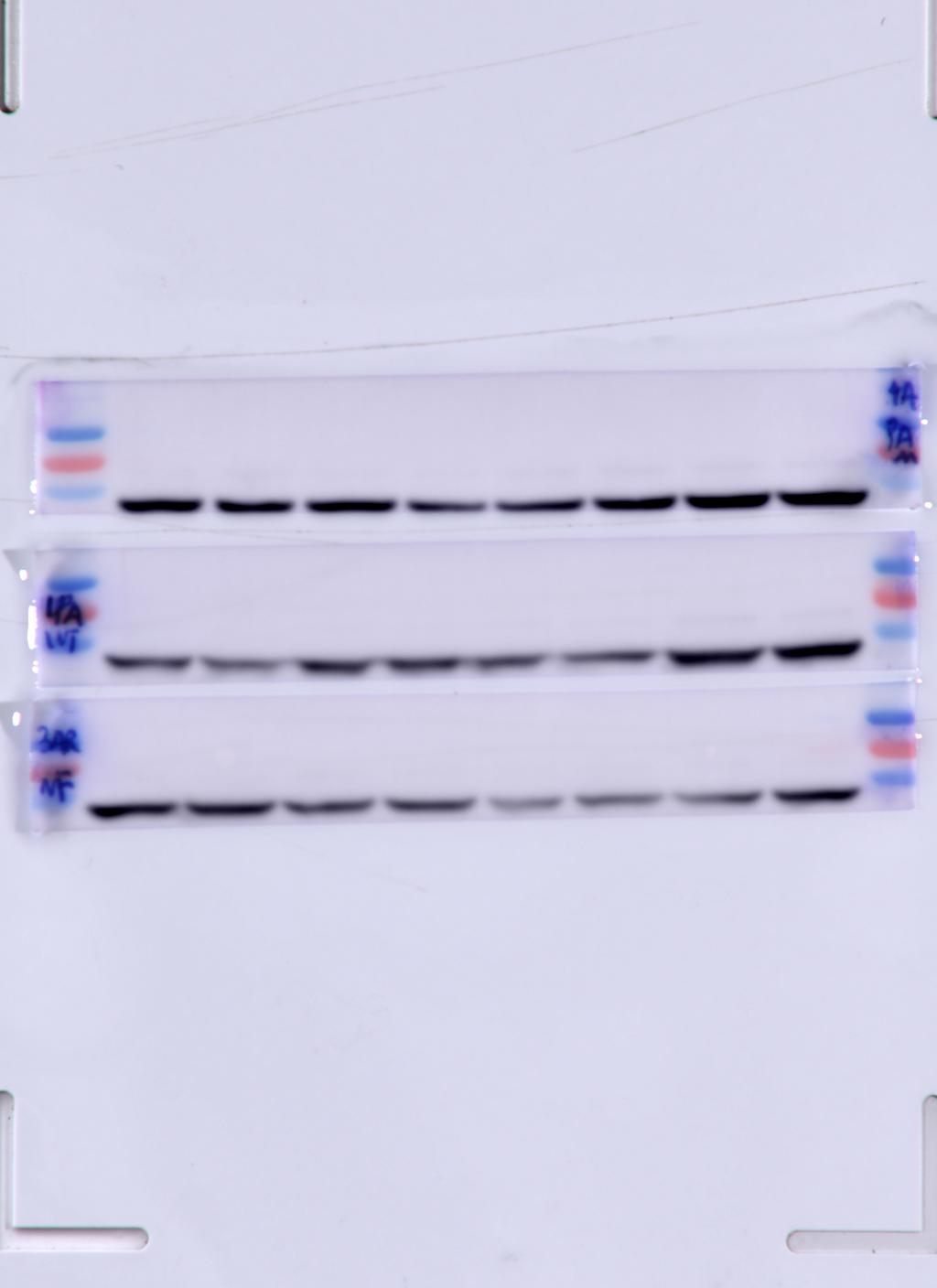

Supplement: Supplementary file 2 — Additional file 2. Raw data of western blot. [file 12974_2022_2632_MOESM2_ESM.zip › supplementary files/Figure3 WB/Beclin new/belin1 2020.08.19_18.02.00_Ch+Marker.jpg]

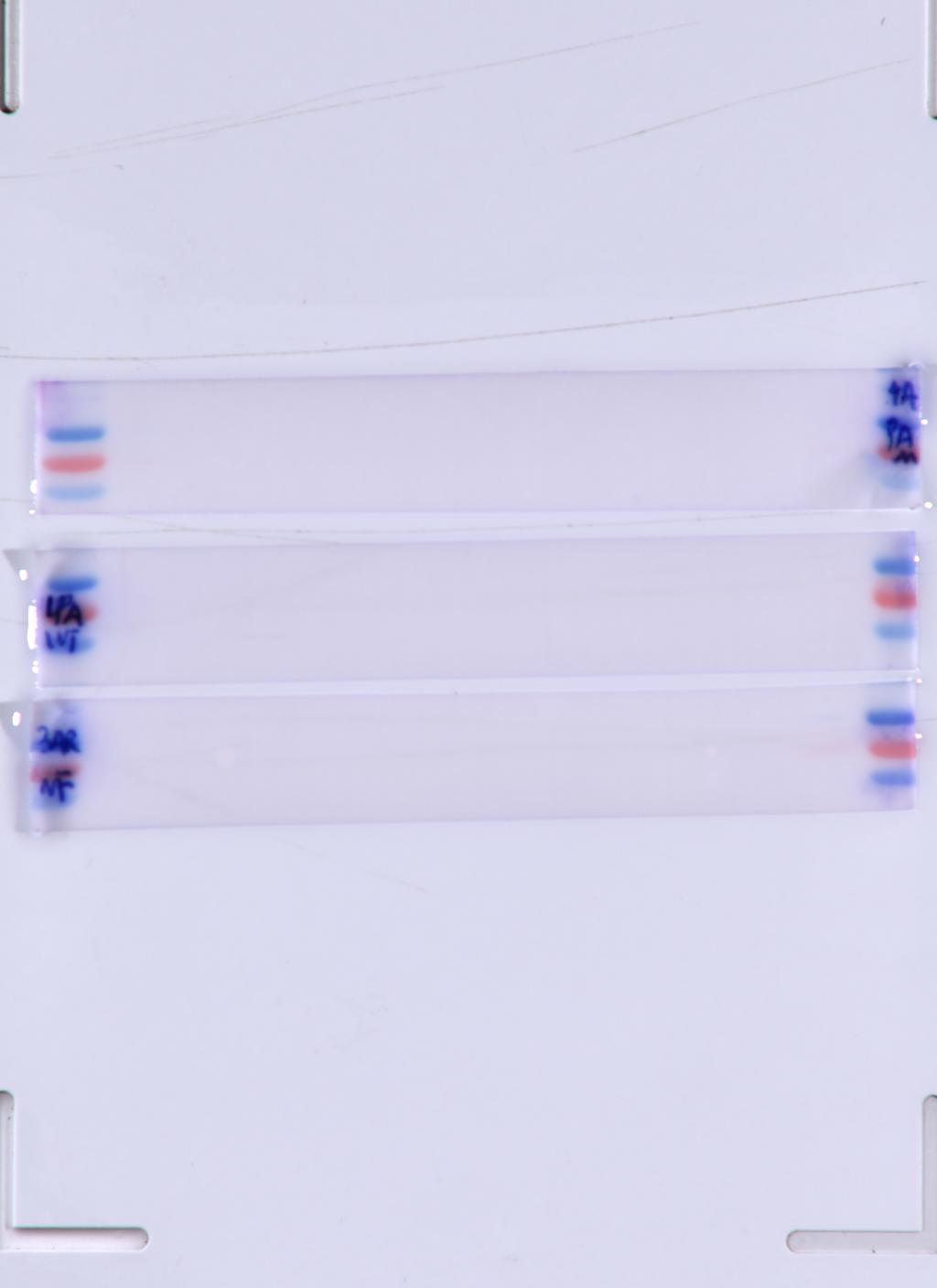

Supplement: Supplementary file 2 — Additional file 2. Raw data of western blot. [file 12974_2022_2632_MOESM2_ESM.zip › supplementary files/Figure3 WB/Beclin new/belin1 2020.08.19_18.02.00_Ch-Marker.jpg]

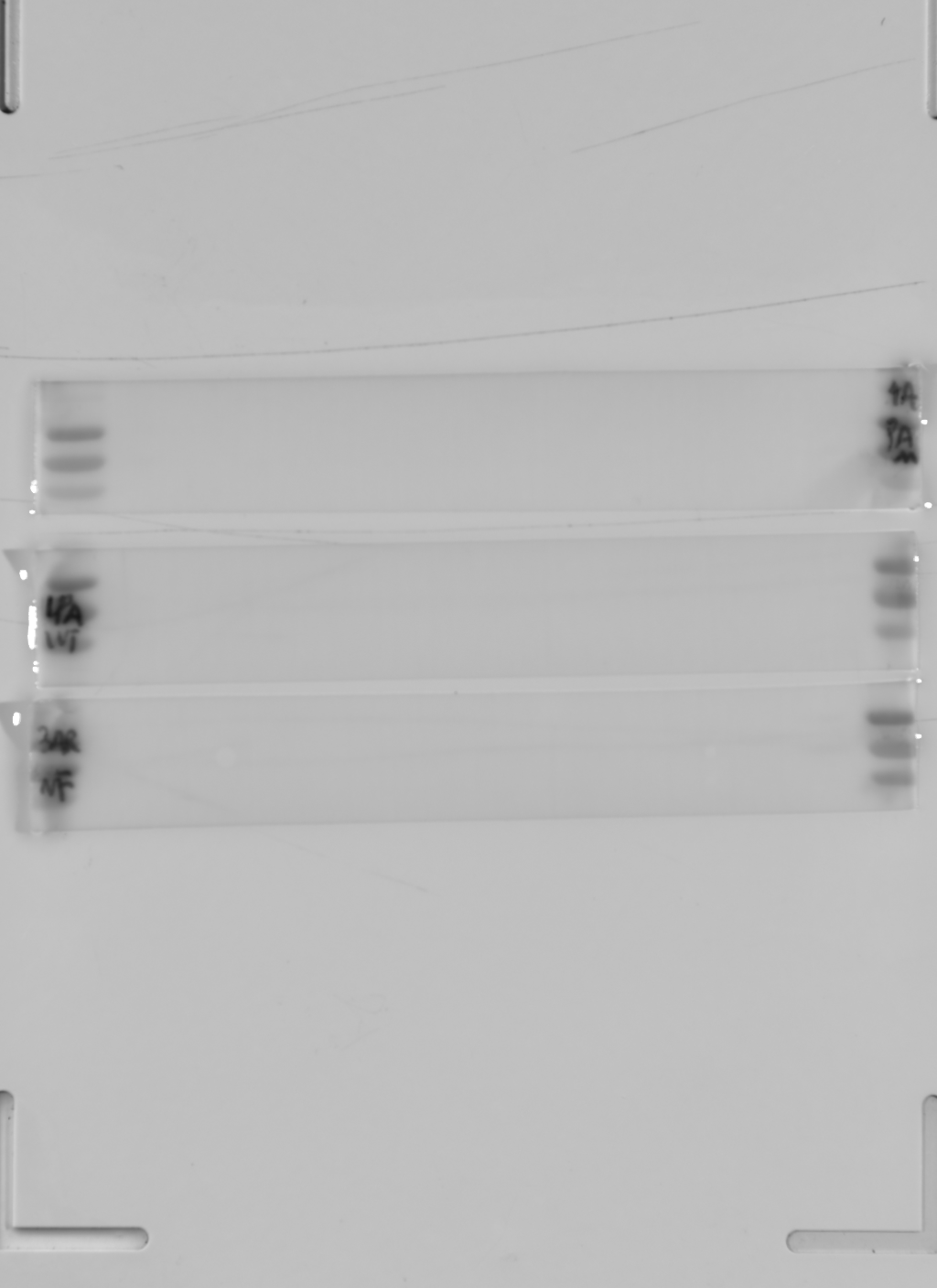

Supplement: Supplementary file 2 — Additional file 2. Raw data of western blot. [file 12974_2022_2632_MOESM2_ESM.zip › supplementary files/Figure3 WB/Beclin new/belin1 2020.08.19_18.02.00_Ch-Marker.tif]

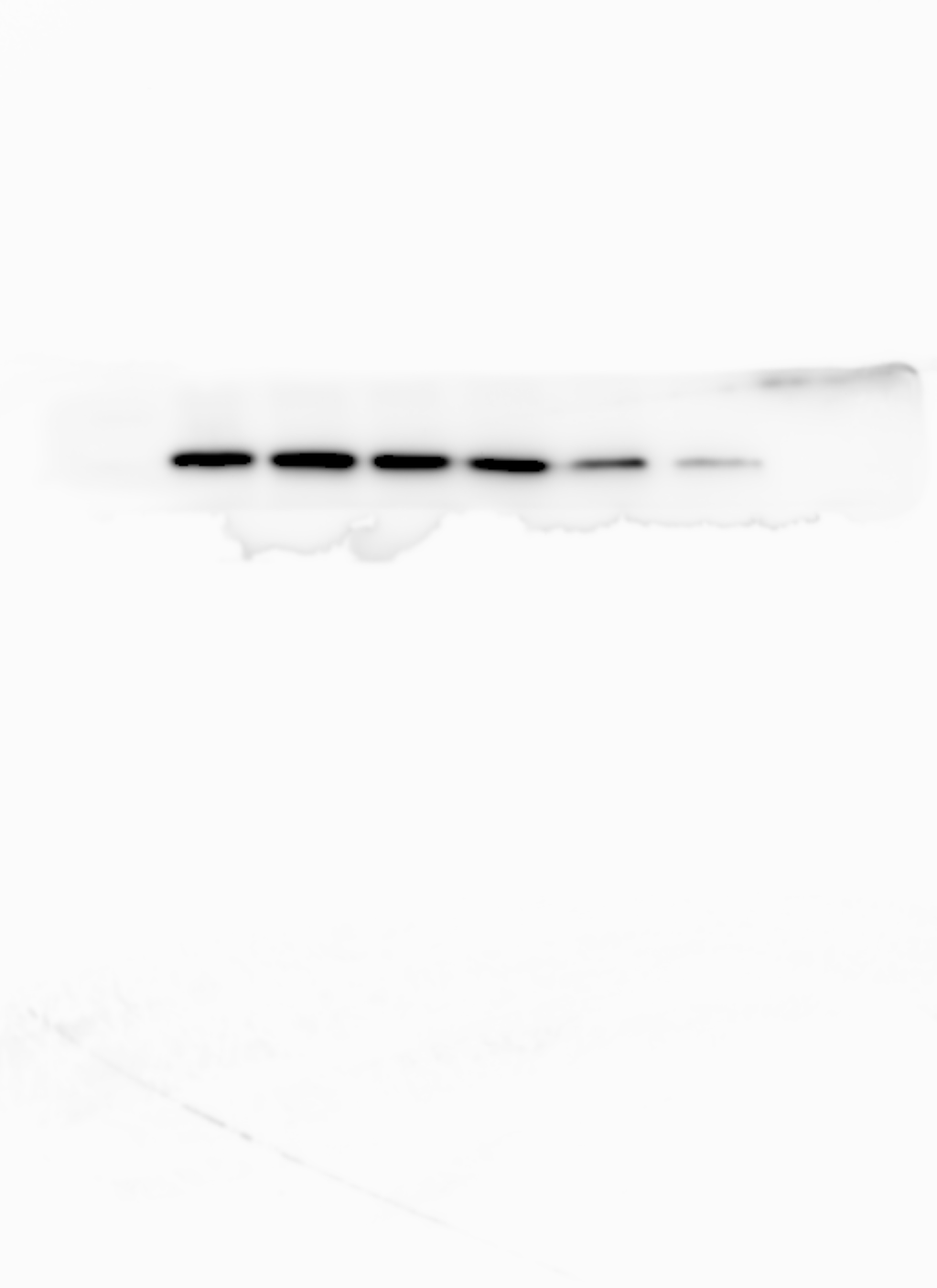

Supplement: Supplementary file 2 — Additional file 2. Raw data of western blot. [file 12974_2022_2632_MOESM2_ESM.zip › supplementary files/Figure3 WB/Beclin1 AR/belin 2020.09.20_18.31.35_Ch.tif]

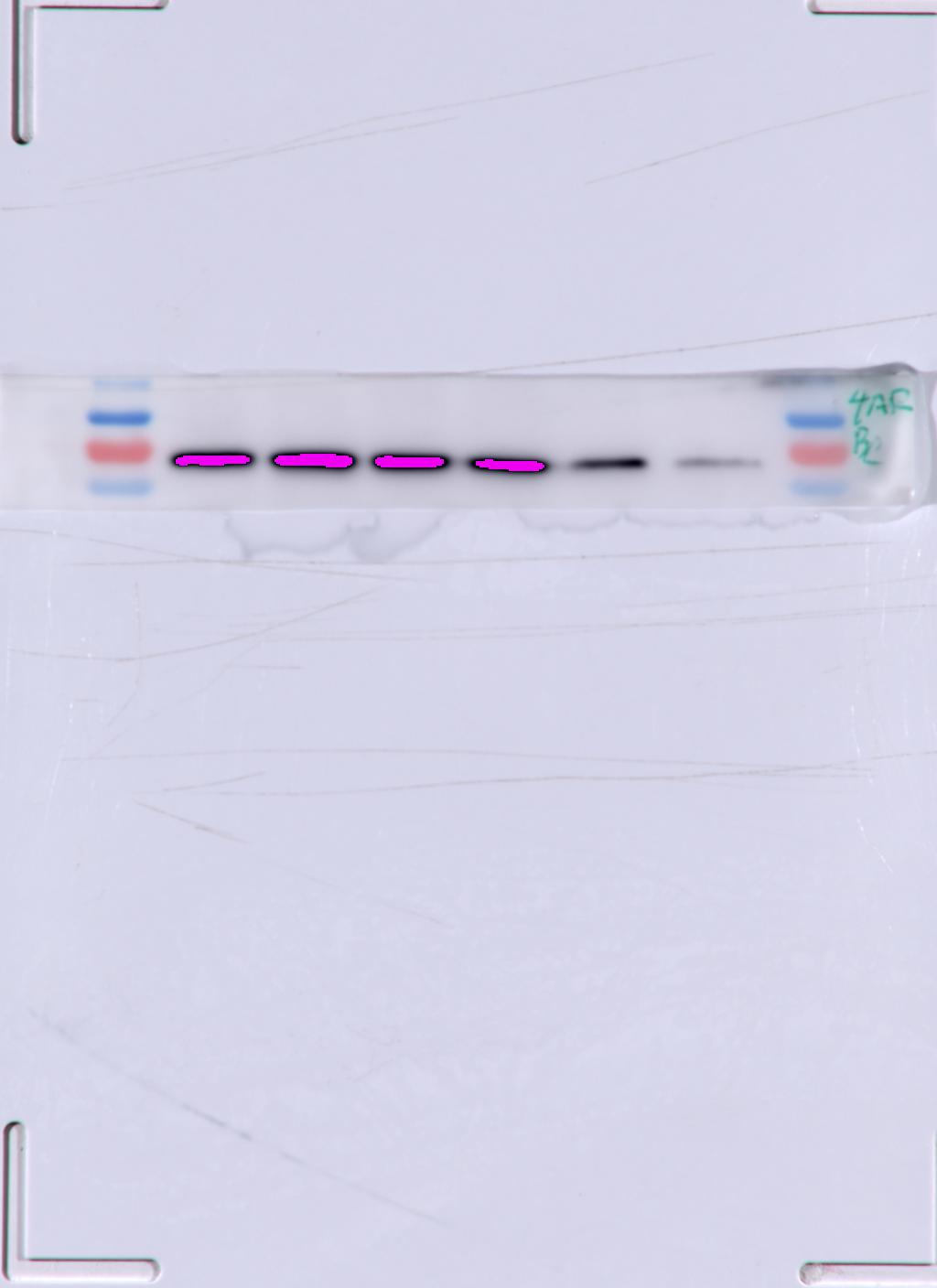

Supplement: Supplementary file 2 — Additional file 2. Raw data of western blot. [file 12974_2022_2632_MOESM2_ESM.zip › supplementary files/Figure3 WB/Beclin1 AR/belin 2020.09.20_18.31.35_Ch+Marker.jpg]

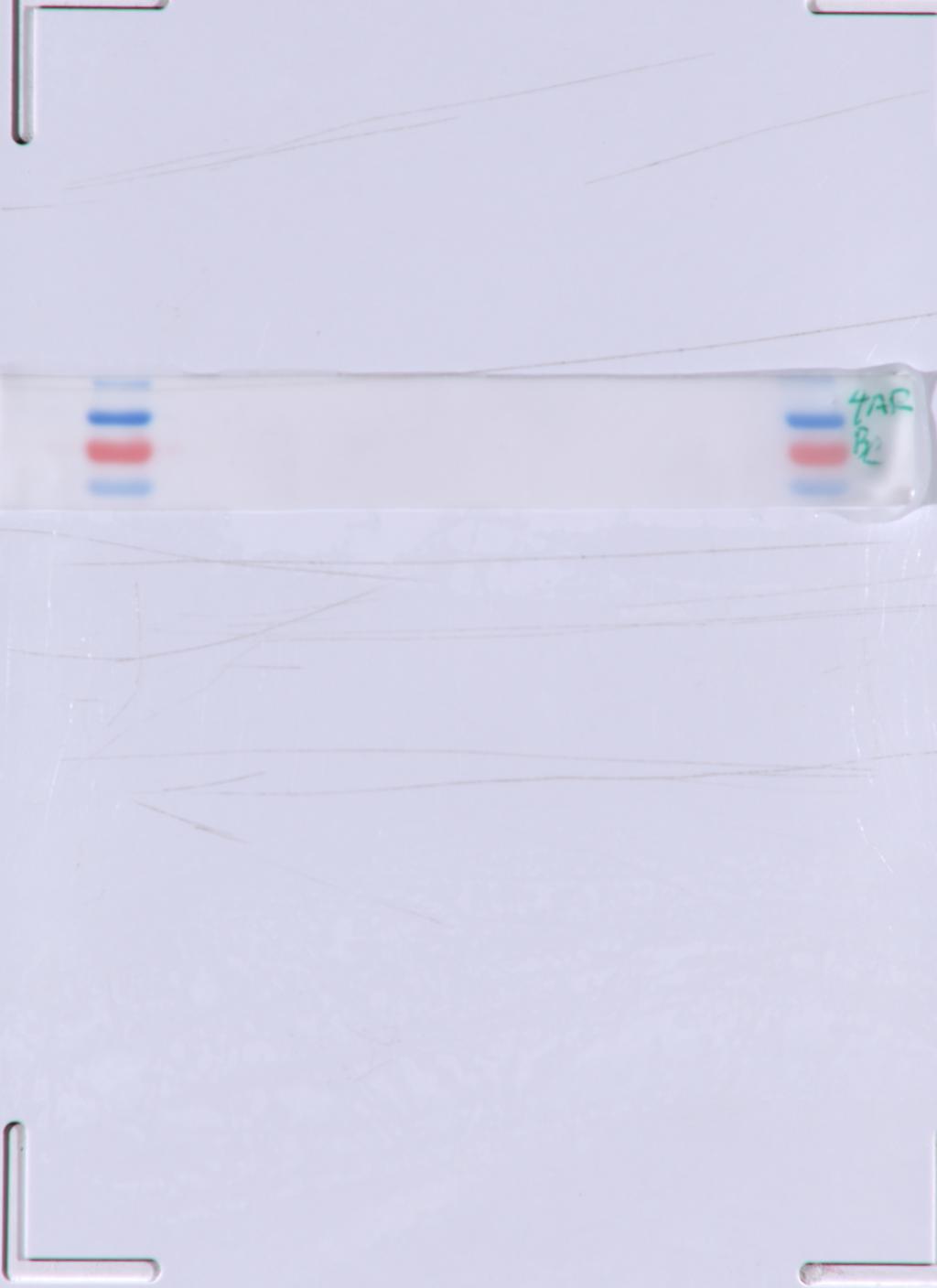

Supplement: Supplementary file 2 — Additional file 2. Raw data of western blot. [file 12974_2022_2632_MOESM2_ESM.zip › supplementary files/Figure3 WB/Beclin1 AR/belin 2020.09.20_18.31.35_Ch-Marker.jpg]

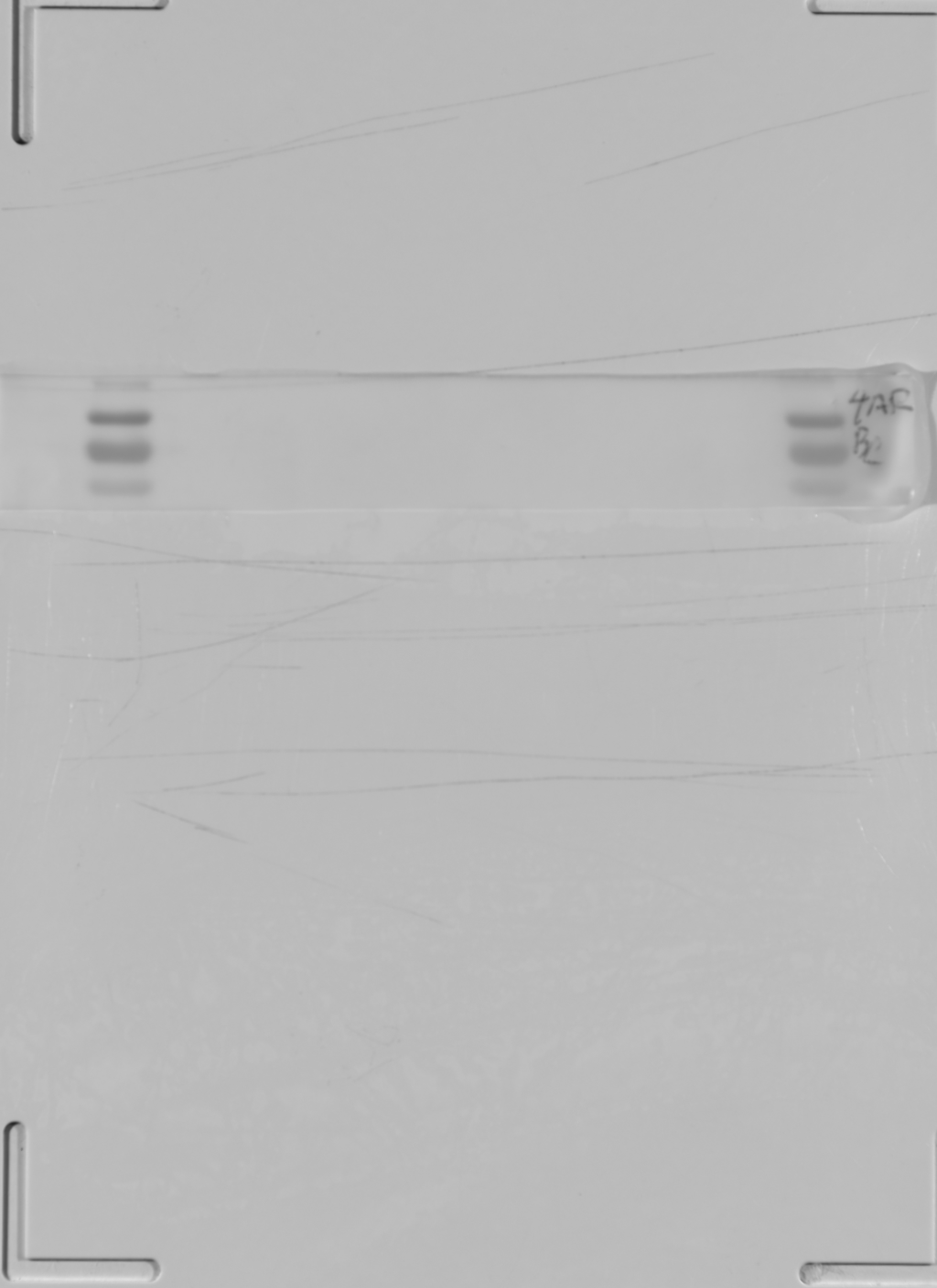

Supplement: Supplementary file 2 — Additional file 2. Raw data of western blot. [file 12974_2022_2632_MOESM2_ESM.zip › supplementary files/Figure3 WB/Beclin1 AR/belin 2020.09.20_18.31.35_Ch-Marker.tif]

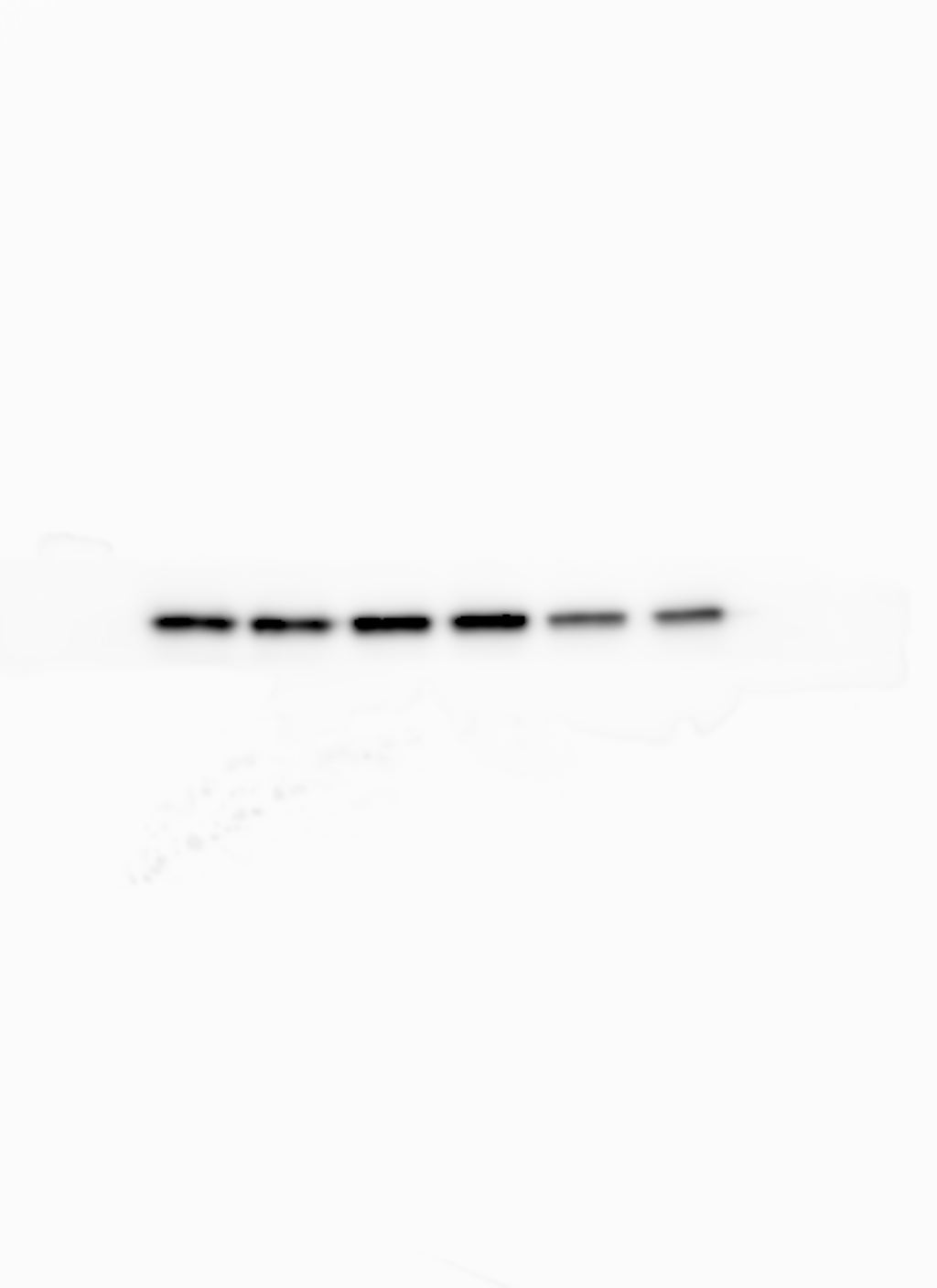

Supplement: Supplementary file 2 — Additional file 2. Raw data of western blot. [file 12974_2022_2632_MOESM2_ESM.zip › supplementary files/Figure3 WB/Beclin1 WT/belin 2020.09.20_17.19.06_Ch.tif]

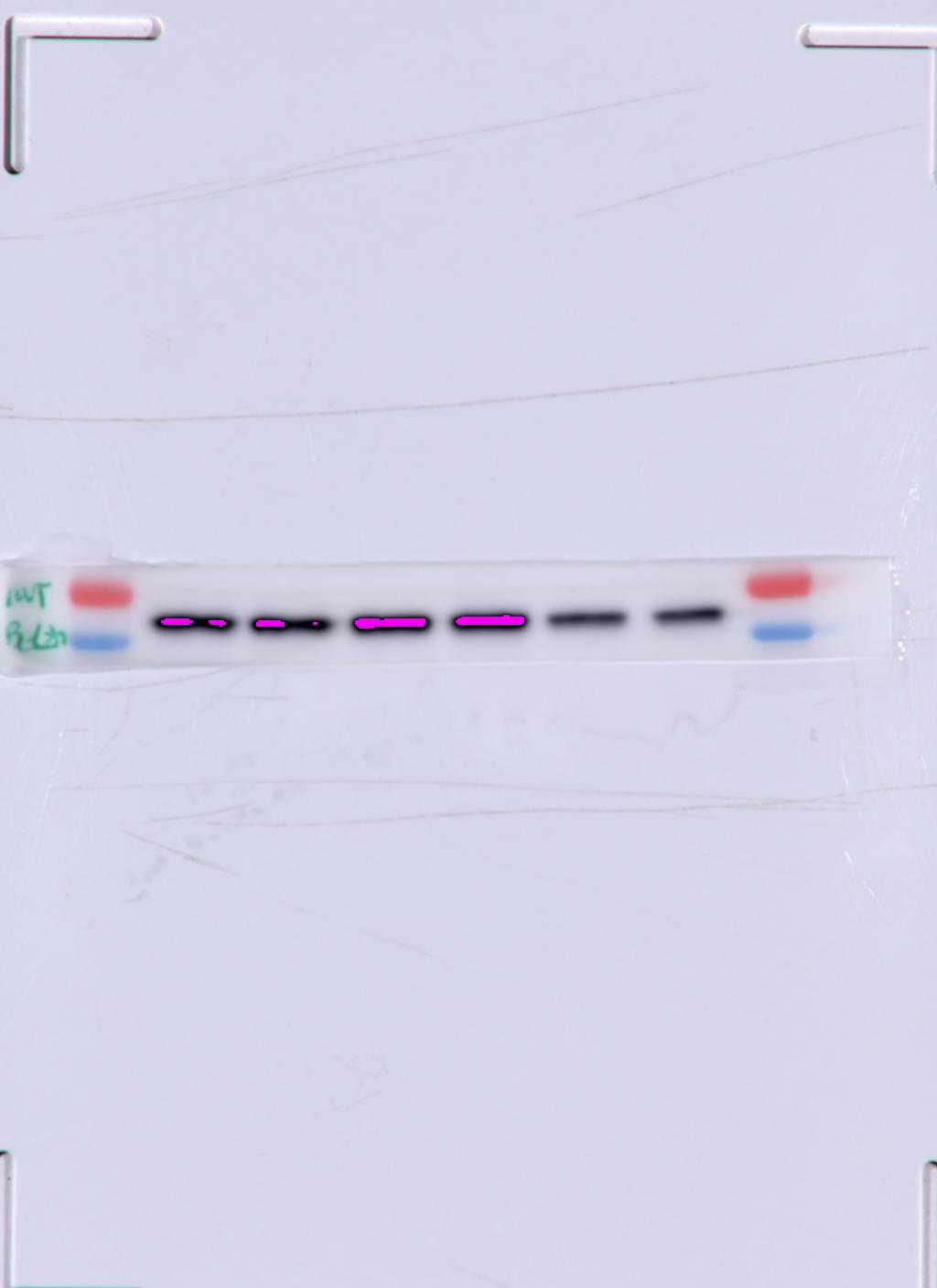

Supplement: Supplementary file 2 — Additional file 2. Raw data of western blot. [file 12974_2022_2632_MOESM2_ESM.zip › supplementary files/Figure3 WB/Beclin1 WT/belin 2020.09.20_17.19.06_Ch+Marker.jpg]

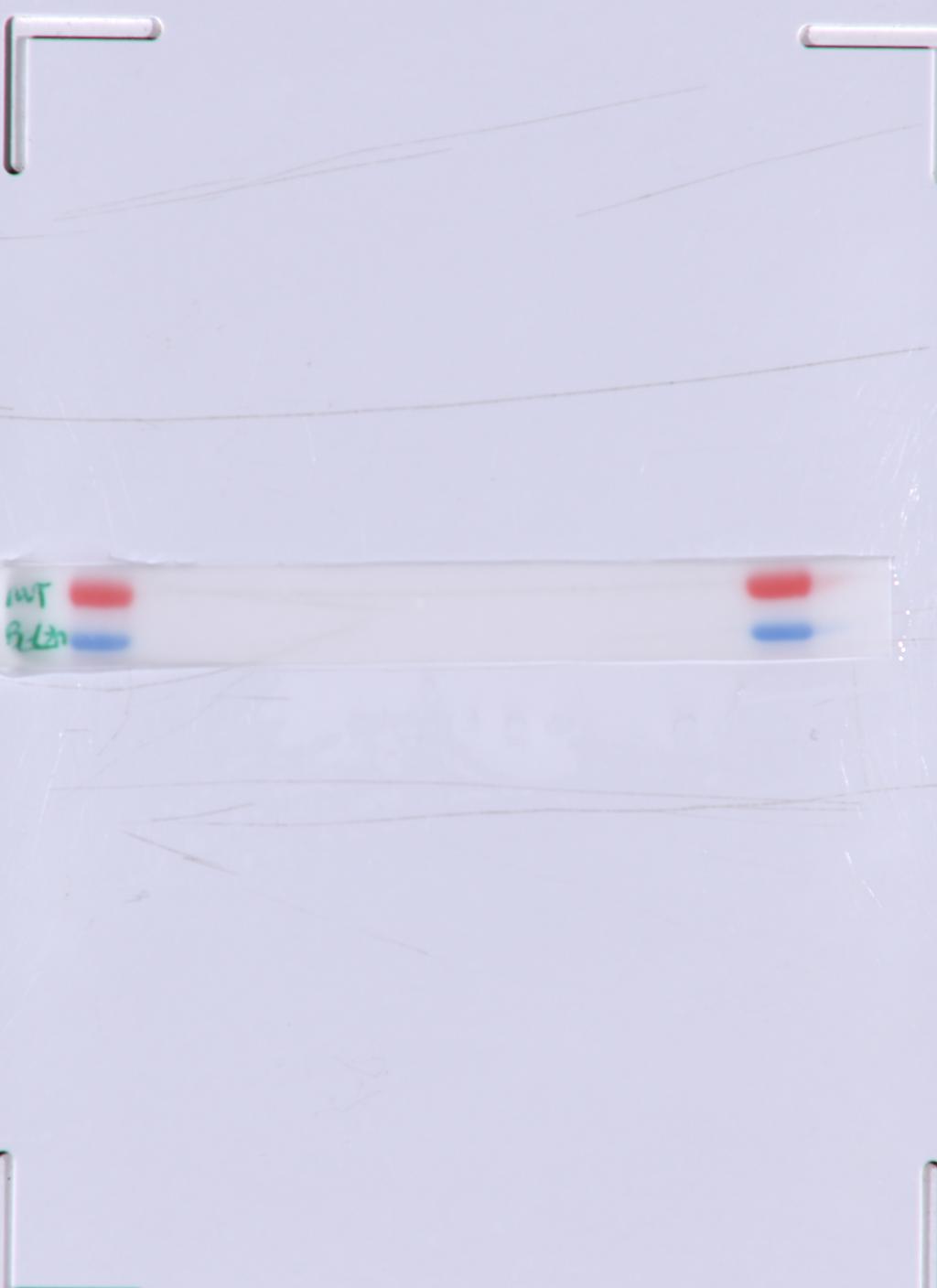

Supplement: Supplementary file 2 — Additional file 2. Raw data of western blot. [file 12974_2022_2632_MOESM2_ESM.zip › supplementary files/Figure3 WB/Beclin1 WT/belin 2020.09.20_17.19.06_Ch-Marker.jpg]

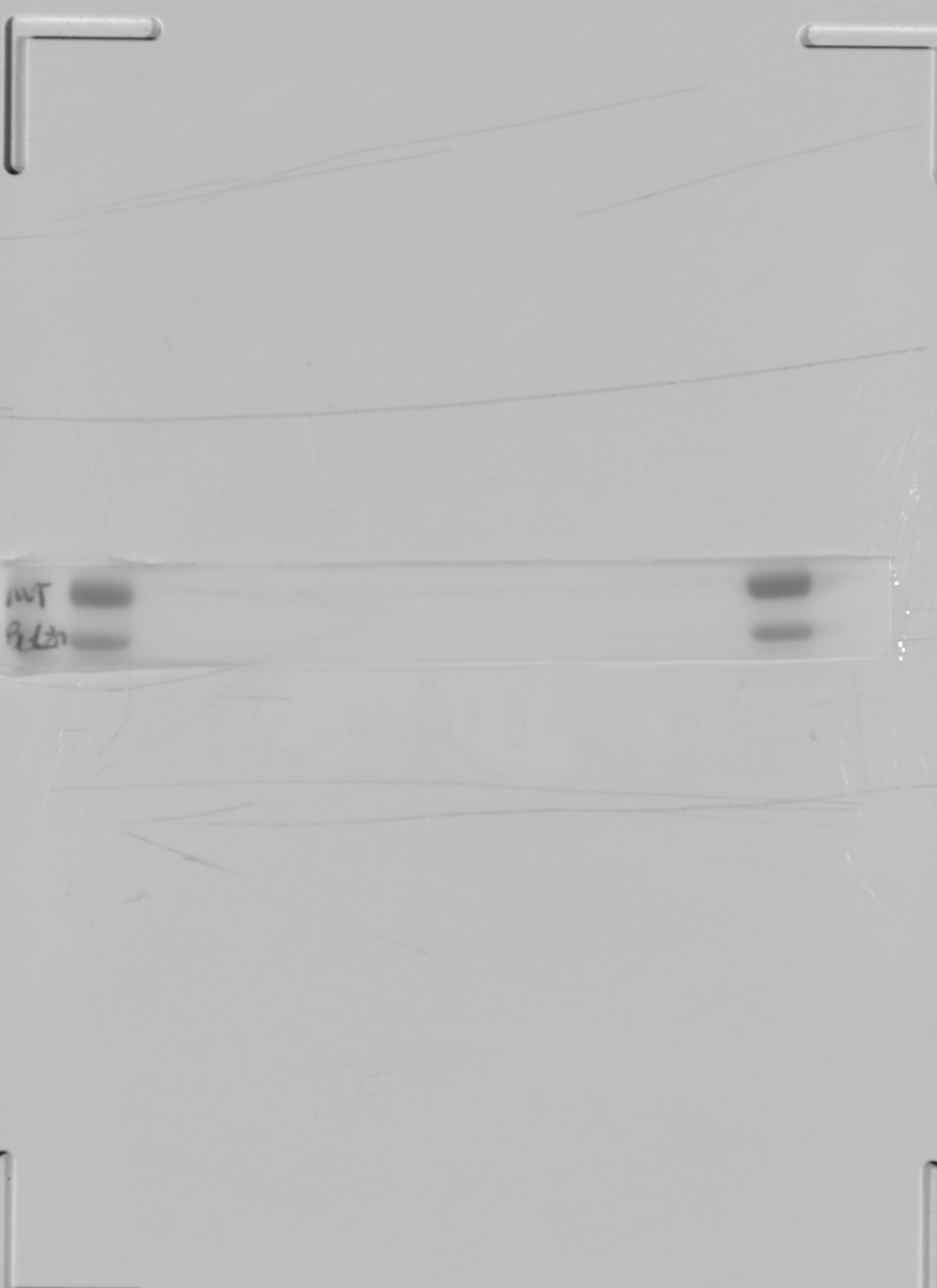

Supplement: Supplementary file 2 — Additional file 2. Raw data of western blot. [file 12974_2022_2632_MOESM2_ESM.zip › supplementary files/Figure3 WB/Beclin1 WT/belin 2020.09.20_17.19.06_Ch-Marker.tif]

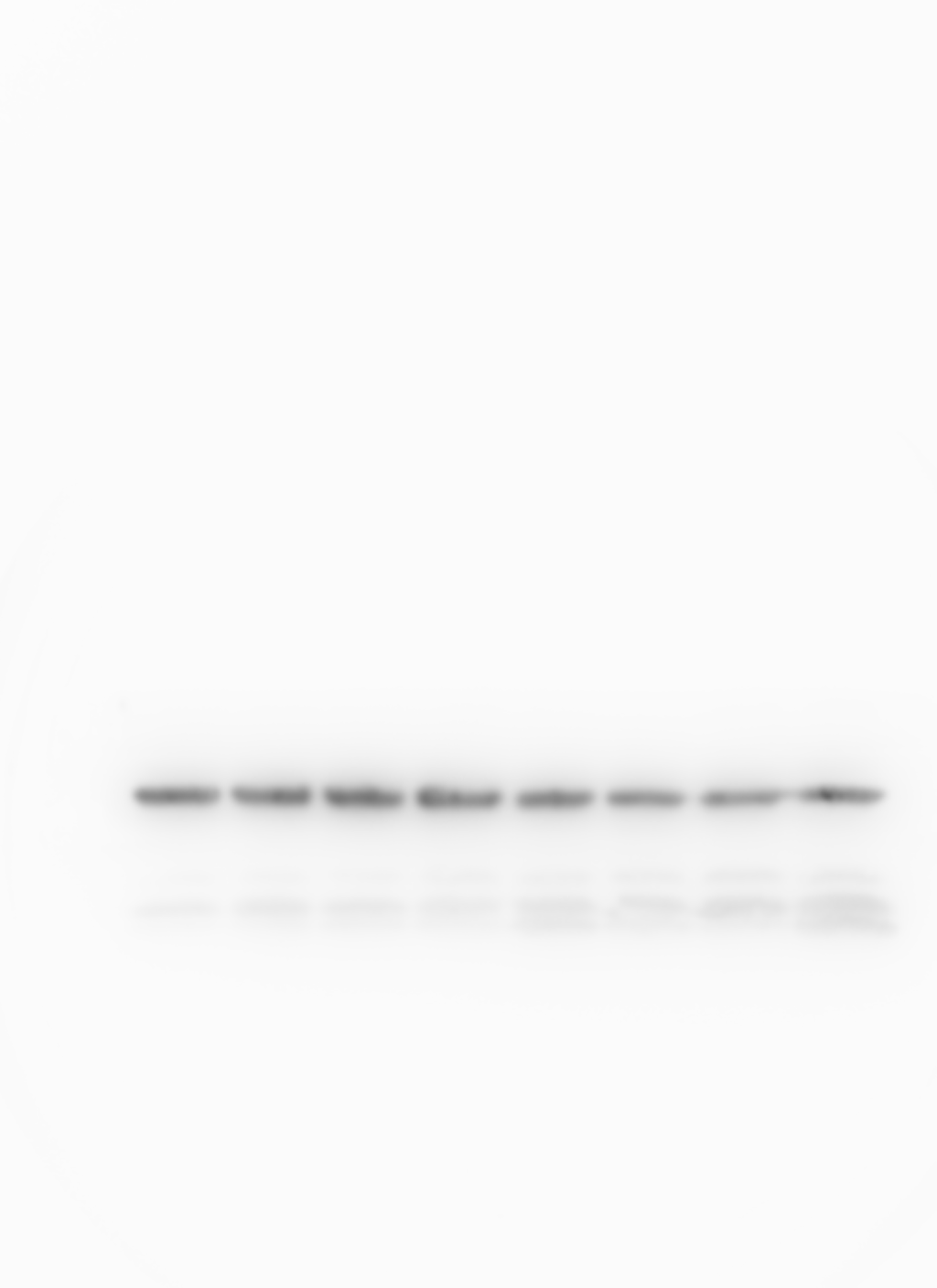

Supplement: Supplementary file 2 — Additional file 2. Raw data of western blot. [file 12974_2022_2632_MOESM2_ESM.zip › supplementary files/Figure3 WB/GAPDH AR/zklc3 2020.01.02_16.05.39_Ch.tif]

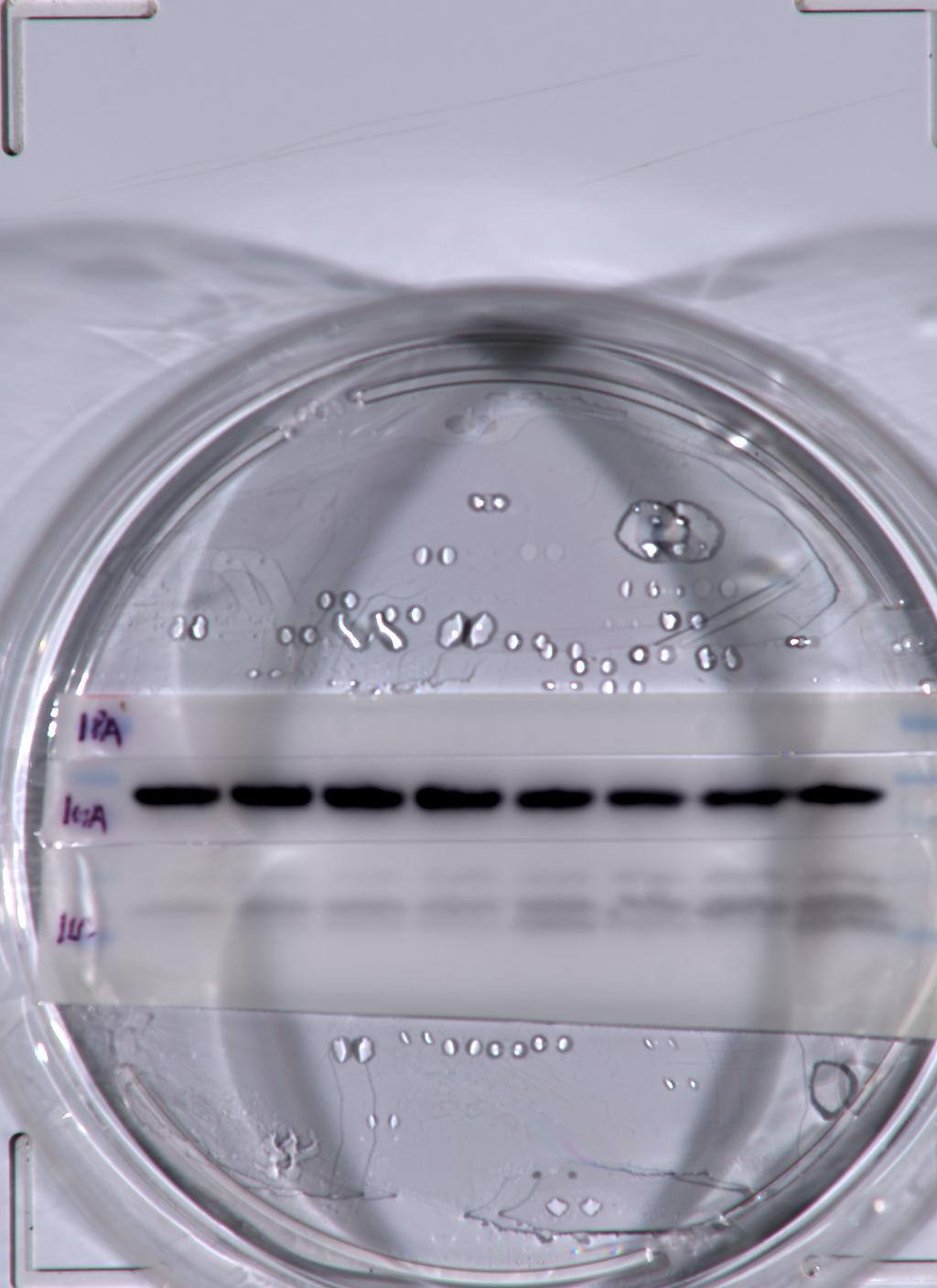

Supplement: Supplementary file 2 — Additional file 2. Raw data of western blot. [file 12974_2022_2632_MOESM2_ESM.zip › supplementary files/Figure3 WB/GAPDH AR/zklc3 2020.01.02_16.05.39_Ch+Marker.jpg]

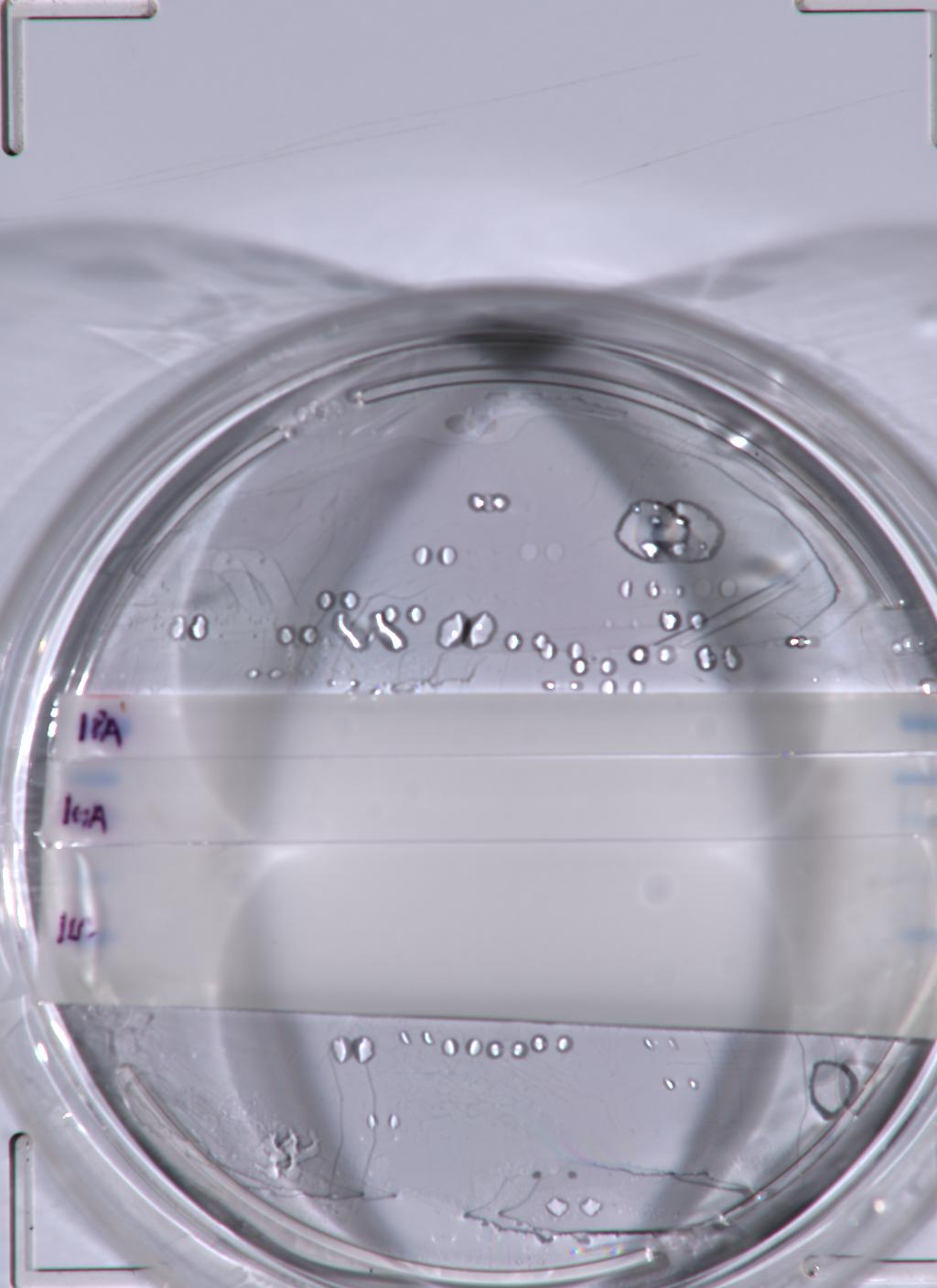

Supplement: Supplementary file 2 — Additional file 2. Raw data of western blot. [file 12974_2022_2632_MOESM2_ESM.zip › supplementary files/Figure3 WB/GAPDH AR/zklc3 2020.01.02_16.05.39_Ch-Marker.jpg]

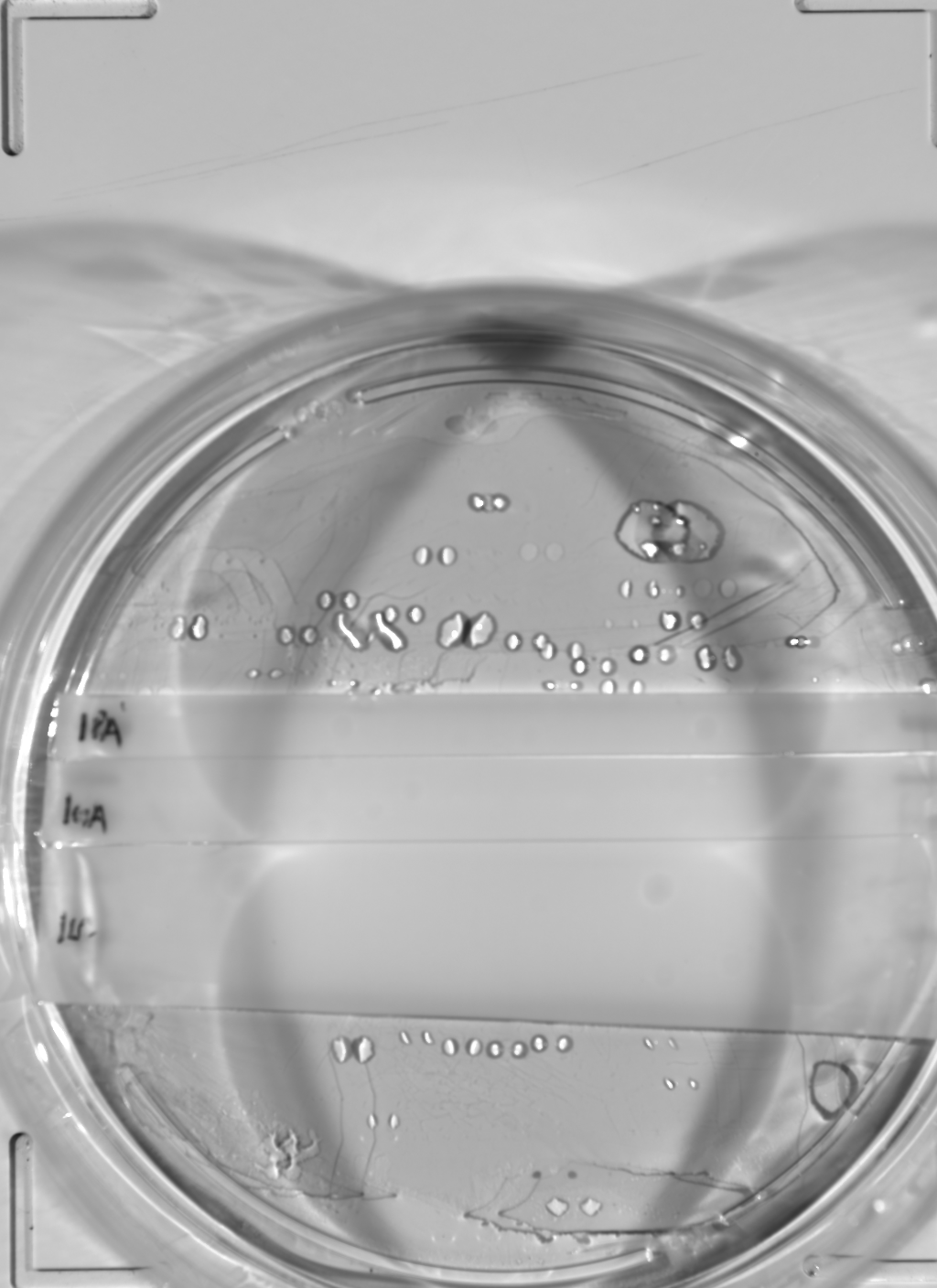

Supplement: Supplementary file 2 — Additional file 2. Raw data of western blot. [file 12974_2022_2632_MOESM2_ESM.zip › supplementary files/Figure3 WB/GAPDH AR/zklc3 2020.01.02_16.05.39_Ch-Marker.tif]

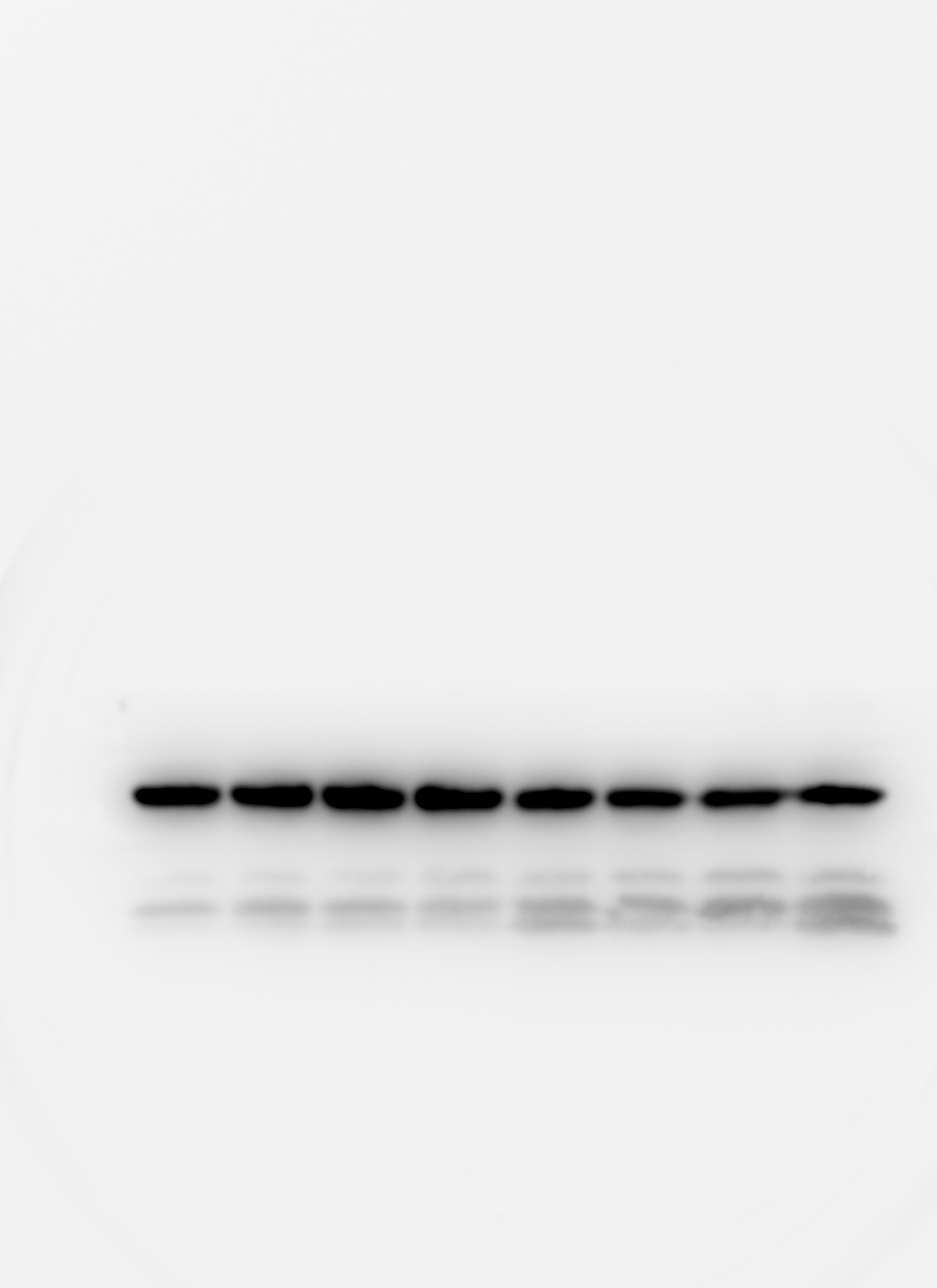

Supplement: Supplementary file 2 — Additional file 2. Raw data of western blot. [file 12974_2022_2632_MOESM2_ESM.zip › supplementary files/Figure3 WB/GAPDH AR/zklc3 2020.01.02_16.05.39_Ch╨▐╕─.tif]

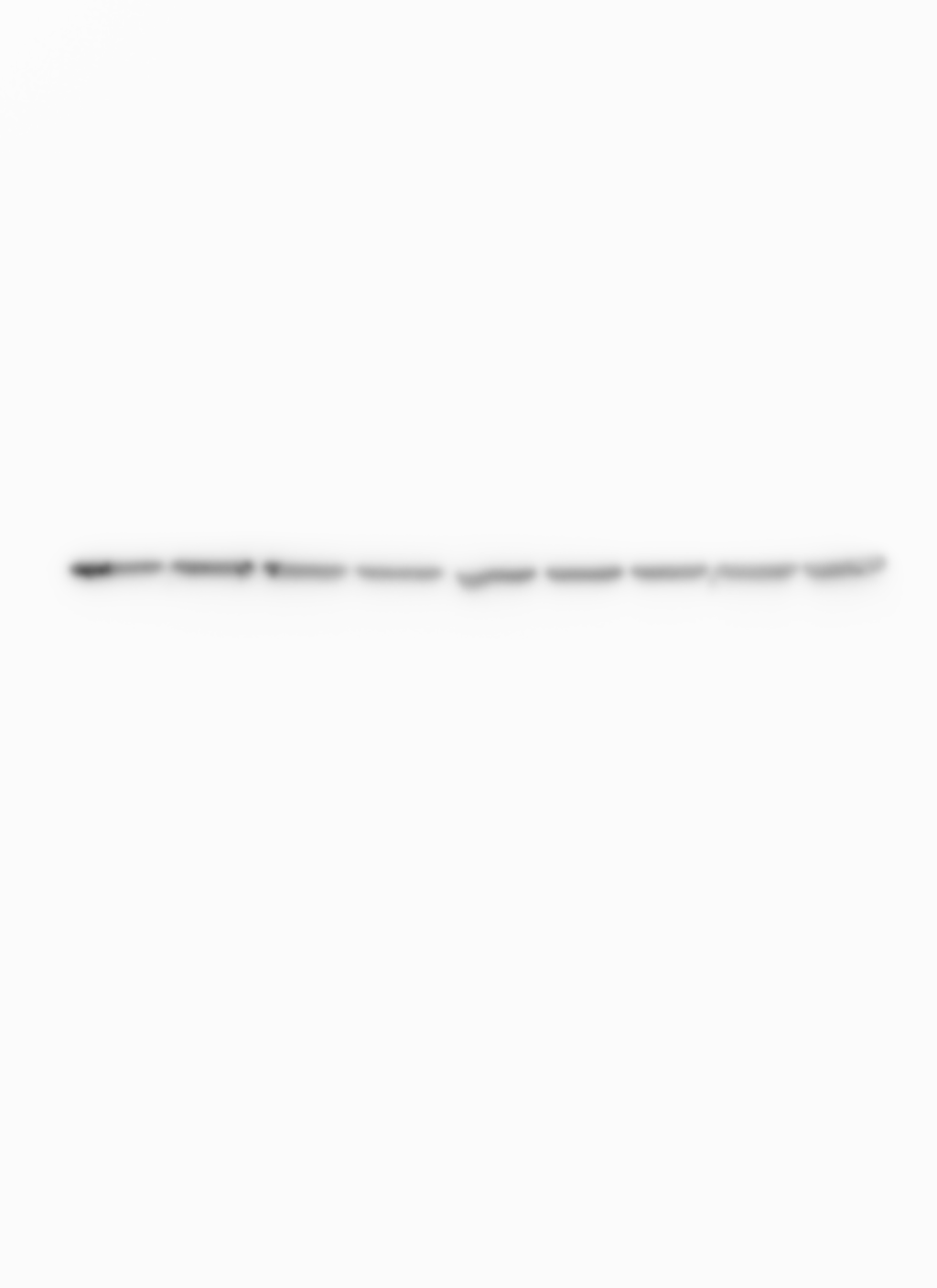

Supplement: Supplementary file 2 — Additional file 2. Raw data of western blot. [file 12974_2022_2632_MOESM2_ESM.zip › supplementary files/Figure3 WB/GAPDH new/3-gapdh 2020.05.16_16.58.34_Ch.tif]

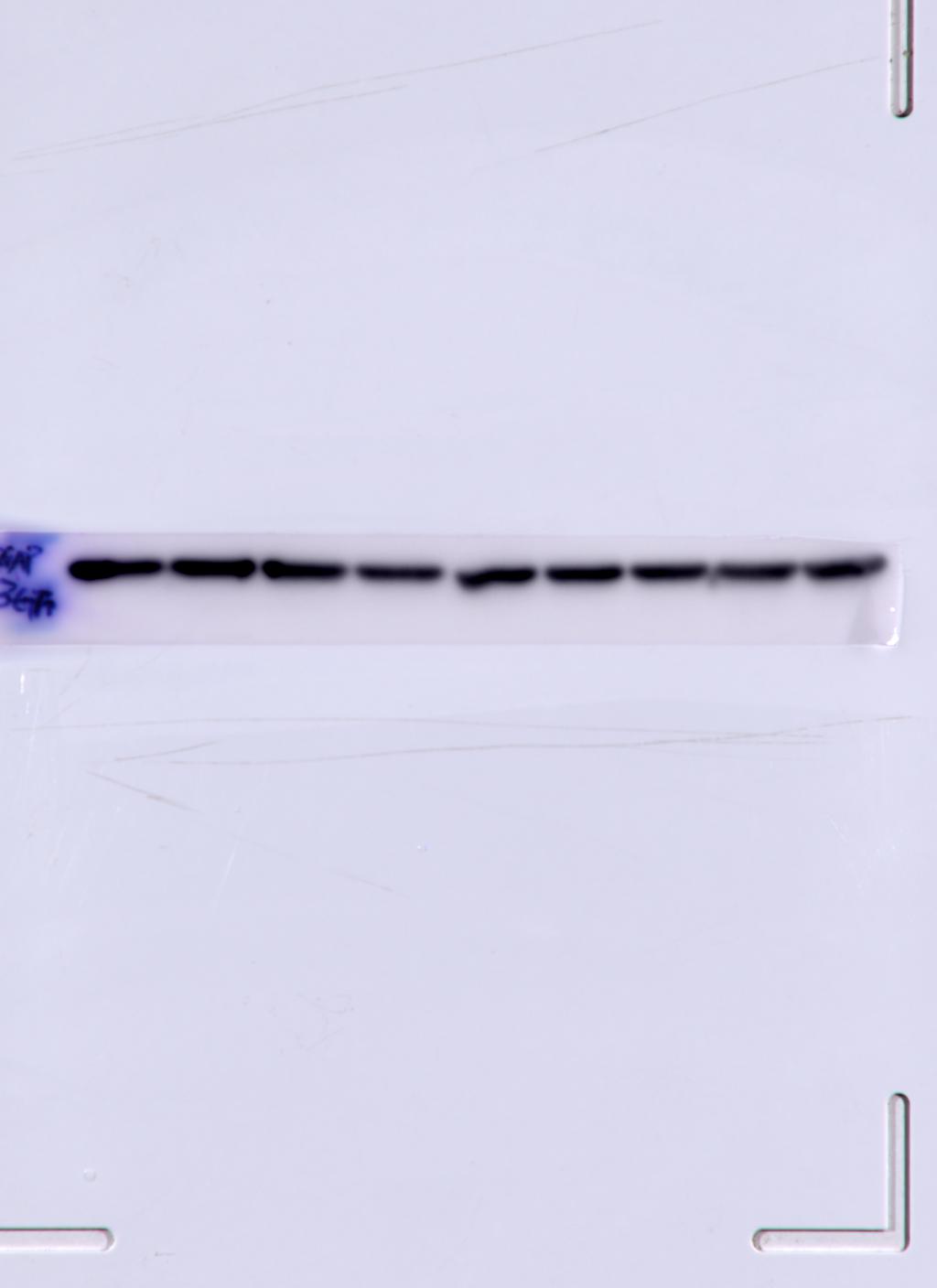

Supplement: Supplementary file 2 — Additional file 2. Raw data of western blot. [file 12974_2022_2632_MOESM2_ESM.zip › supplementary files/Figure3 WB/GAPDH new/3-gapdh 2020.05.16_16.58.34_Ch+Marker.jpg]

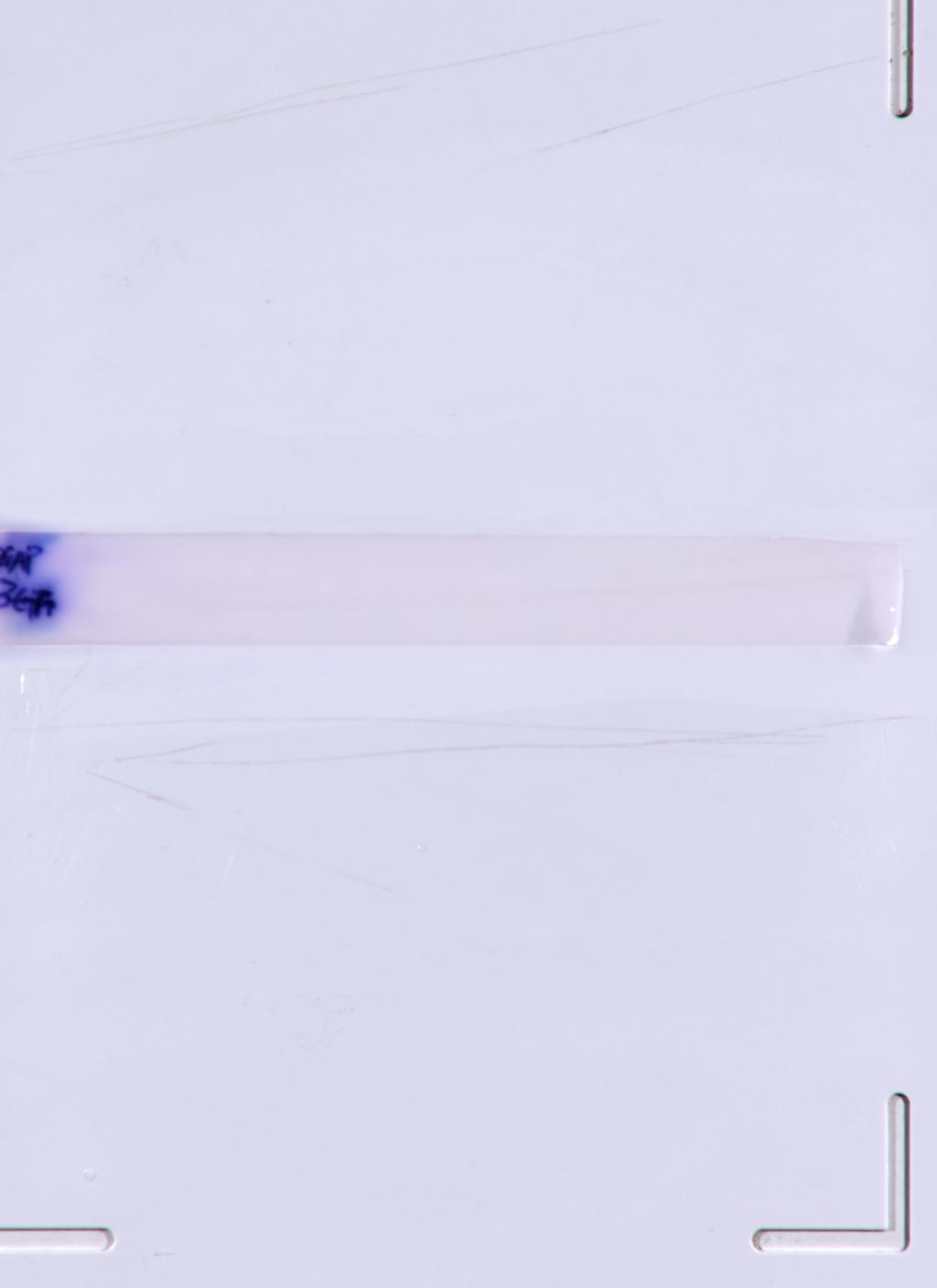

Supplement: Supplementary file 2 — Additional file 2. Raw data of western blot. [file 12974_2022_2632_MOESM2_ESM.zip › supplementary files/Figure3 WB/GAPDH new/3-gapdh 2020.05.16_16.58.34_Ch-Marker.jpg]

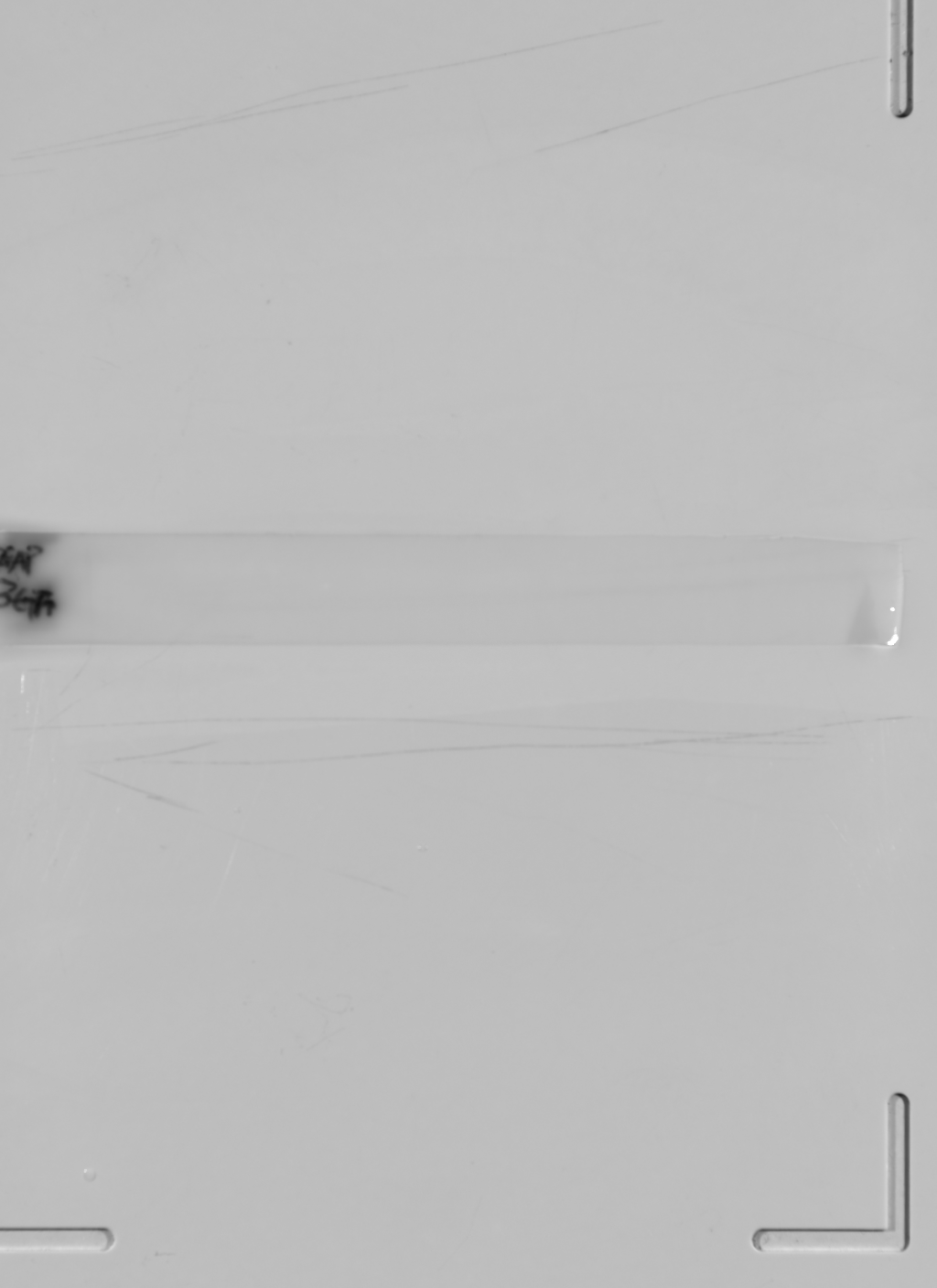

Supplement: Supplementary file 2 — Additional file 2. Raw data of western blot. [file 12974_2022_2632_MOESM2_ESM.zip › supplementary files/Figure3 WB/GAPDH new/3-gapdh 2020.05.16_16.58.34_Ch-Marker.tif]

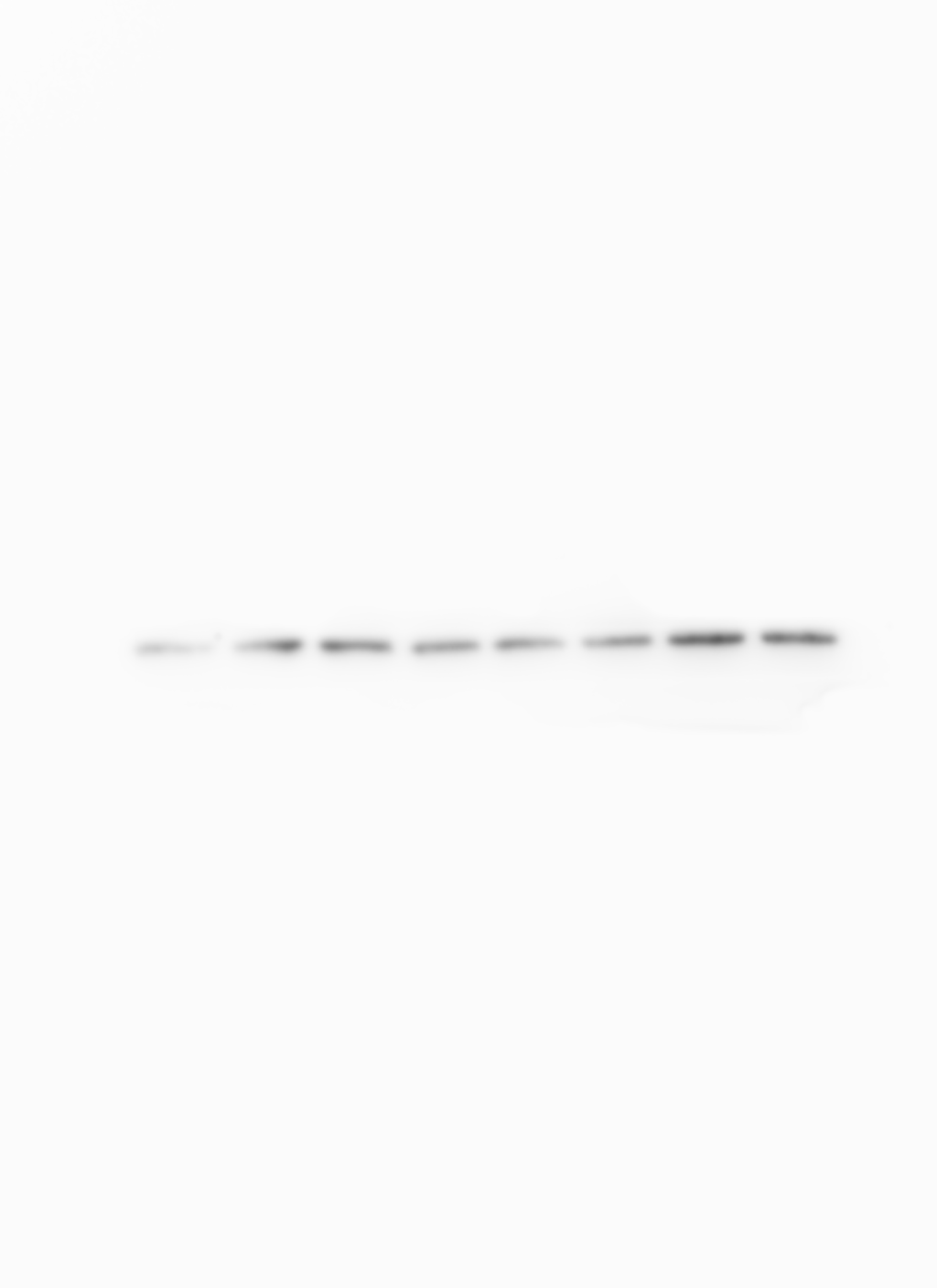

Supplement: Supplementary file 2 — Additional file 2. Raw data of western blot. [file 12974_2022_2632_MOESM2_ESM.zip › supplementary files/Figure3 WB/GAPDH new/4gapdh 2020.01.10_15.48.47_Ch.tif]

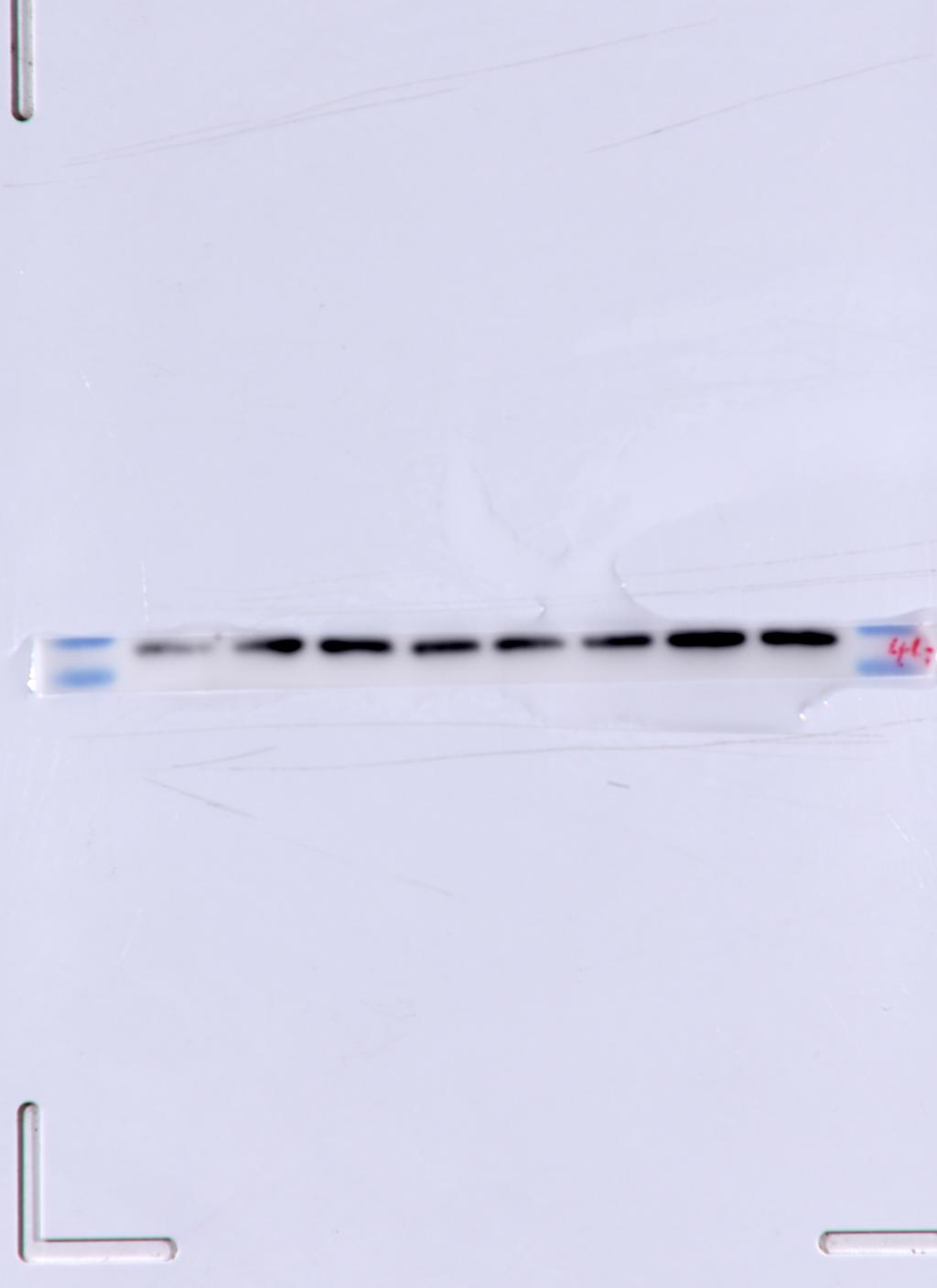

Supplement: Supplementary file 2 — Additional file 2. Raw data of western blot. [file 12974_2022_2632_MOESM2_ESM.zip › supplementary files/Figure3 WB/GAPDH new/4gapdh 2020.01.10_15.48.47_Ch+Marker.jpg]

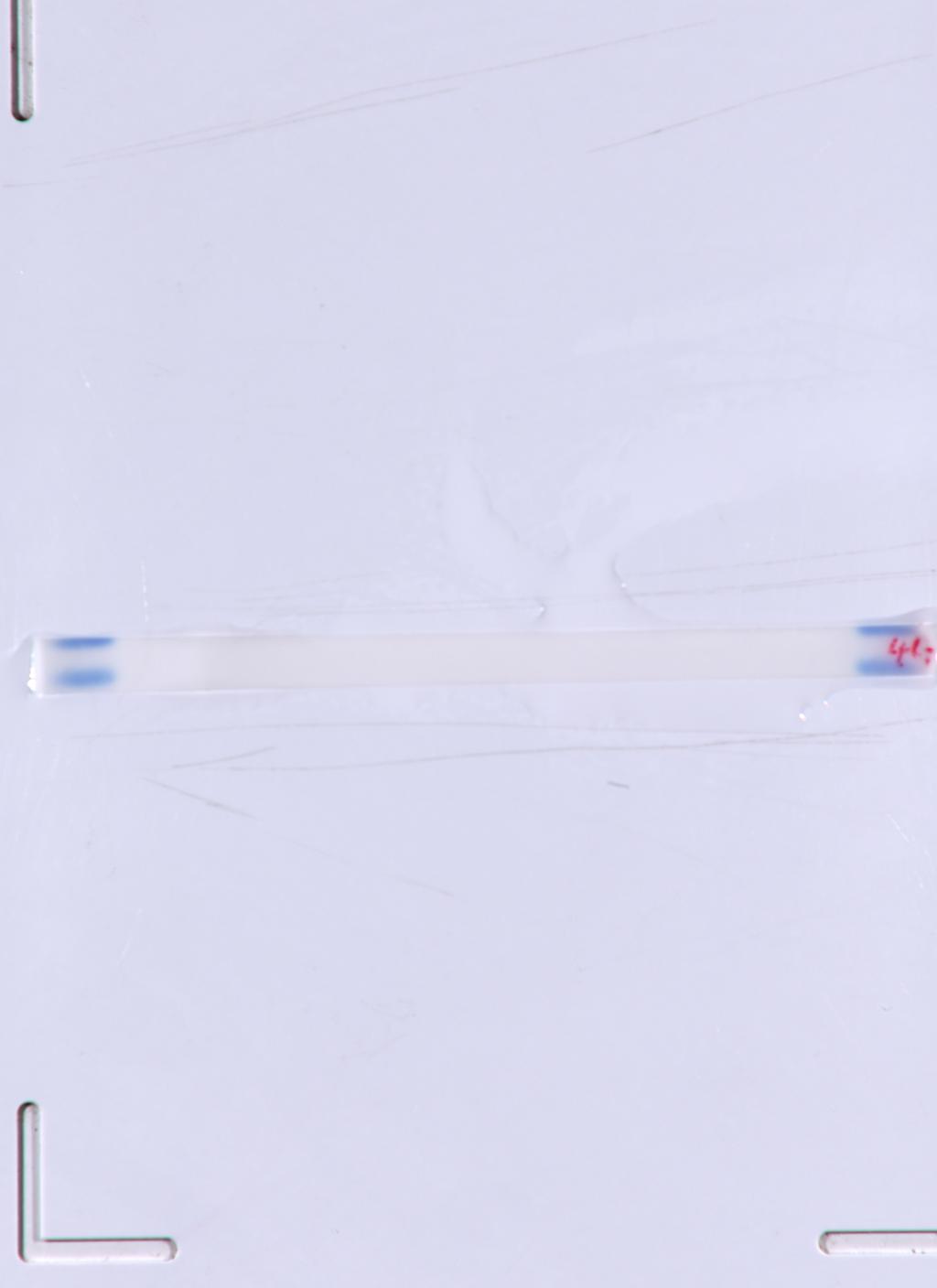

Supplement: Supplementary file 2 — Additional file 2. Raw data of western blot. [file 12974_2022_2632_MOESM2_ESM.zip › supplementary files/Figure3 WB/GAPDH new/4gapdh 2020.01.10_15.48.47_Ch-Marker.jpg]

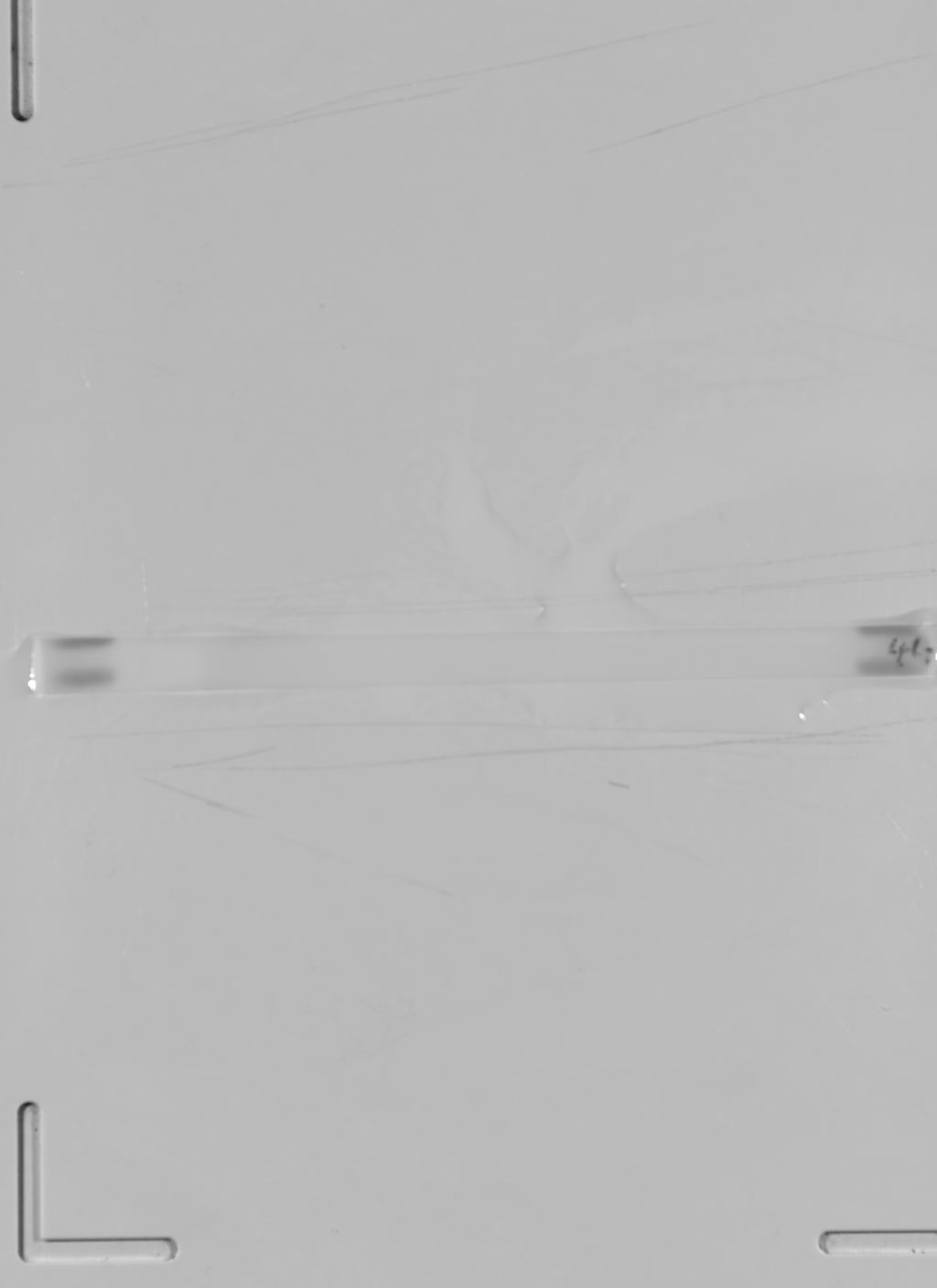

Supplement: Supplementary file 2 — Additional file 2. Raw data of western blot. [file 12974_2022_2632_MOESM2_ESM.zip › supplementary files/Figure3 WB/GAPDH new/4gapdh 2020.01.10_15.48.47_Ch-Marker.tif]

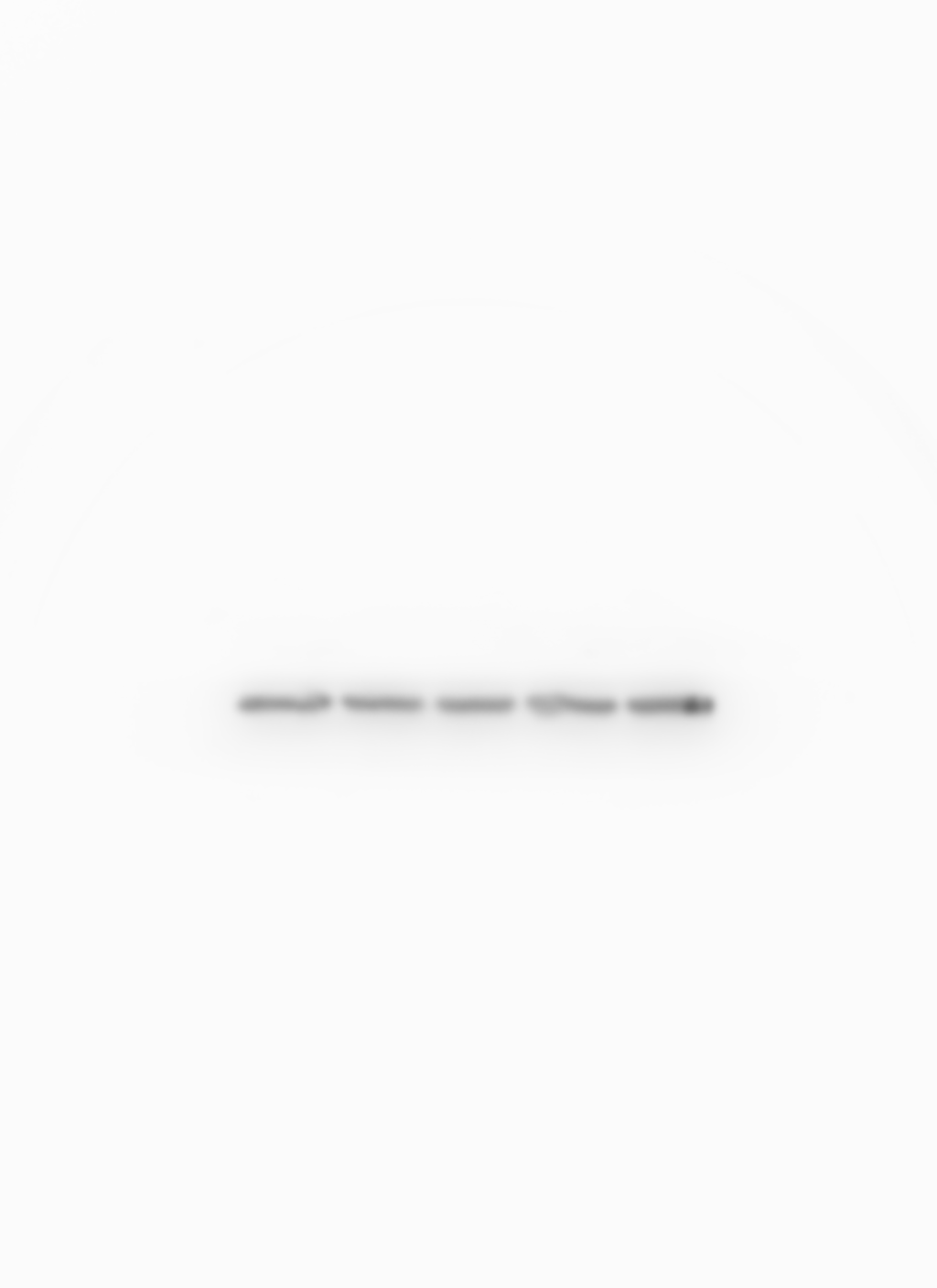

Supplement: Supplementary file 2 — Additional file 2. Raw data of western blot. [file 12974_2022_2632_MOESM2_ESM.zip › supplementary files/Figure3 WB/GAPDH WT/zkgapdh 2020.01.02_15.46.47_Ch.tif]

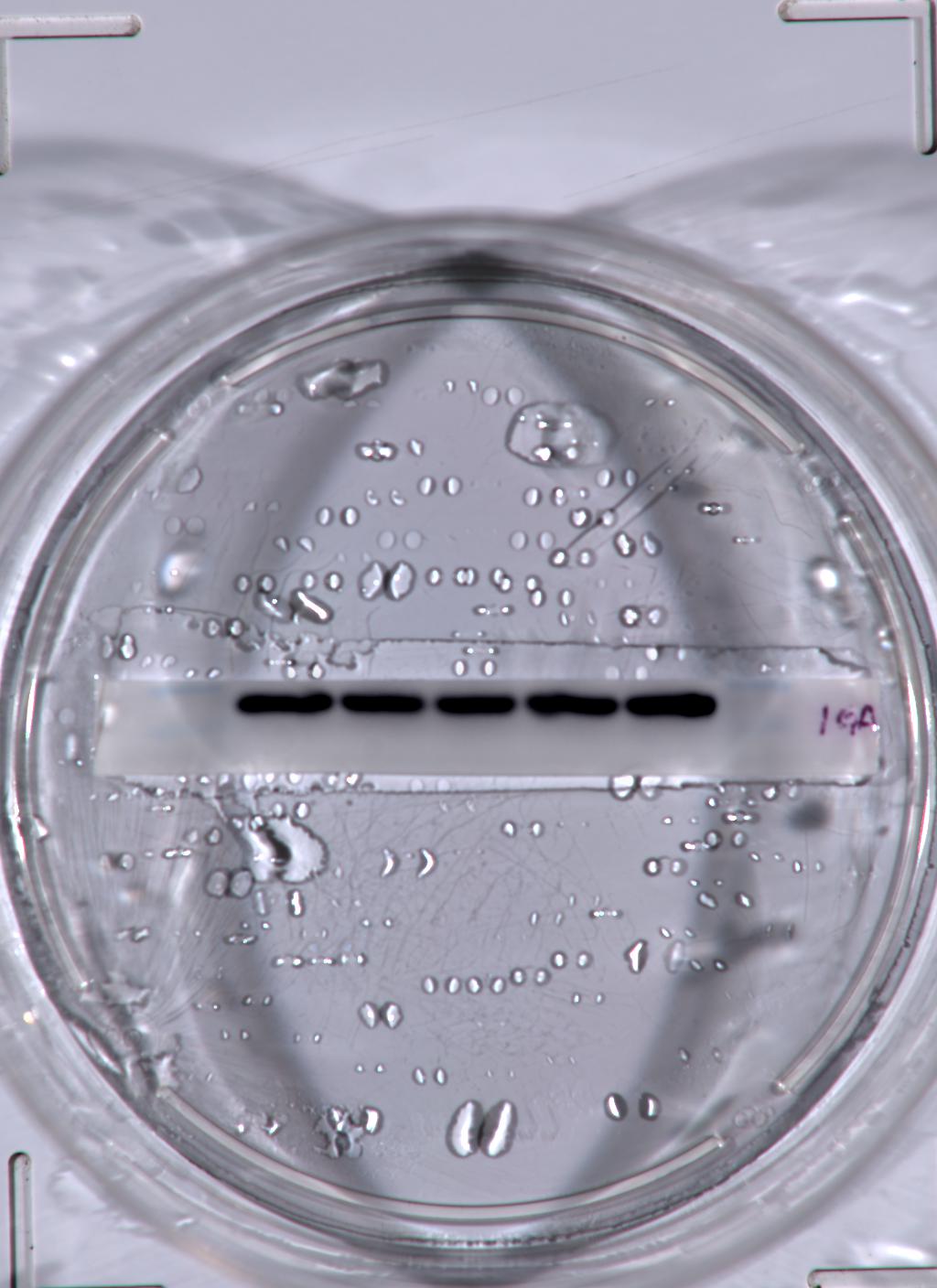

Supplement: Supplementary file 2 — Additional file 2. Raw data of western blot. [file 12974_2022_2632_MOESM2_ESM.zip › supplementary files/Figure3 WB/GAPDH WT/zkgapdh 2020.01.02_15.46.47_Ch+Marker.jpg]

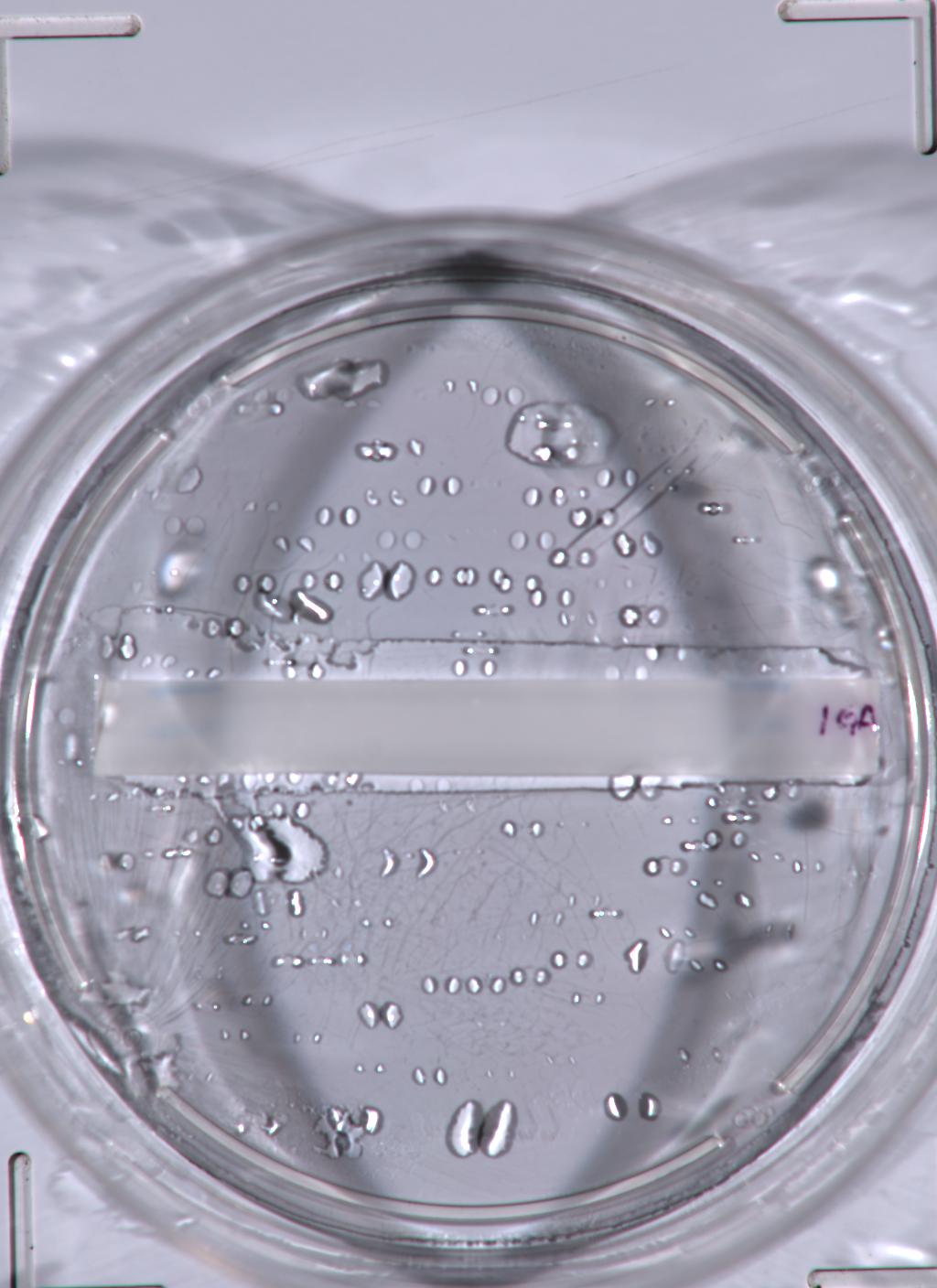

Supplement: Supplementary file 2 — Additional file 2. Raw data of western blot. [file 12974_2022_2632_MOESM2_ESM.zip › supplementary files/Figure3 WB/GAPDH WT/zkgapdh 2020.01.02_15.46.47_Ch-Marker.jpg]

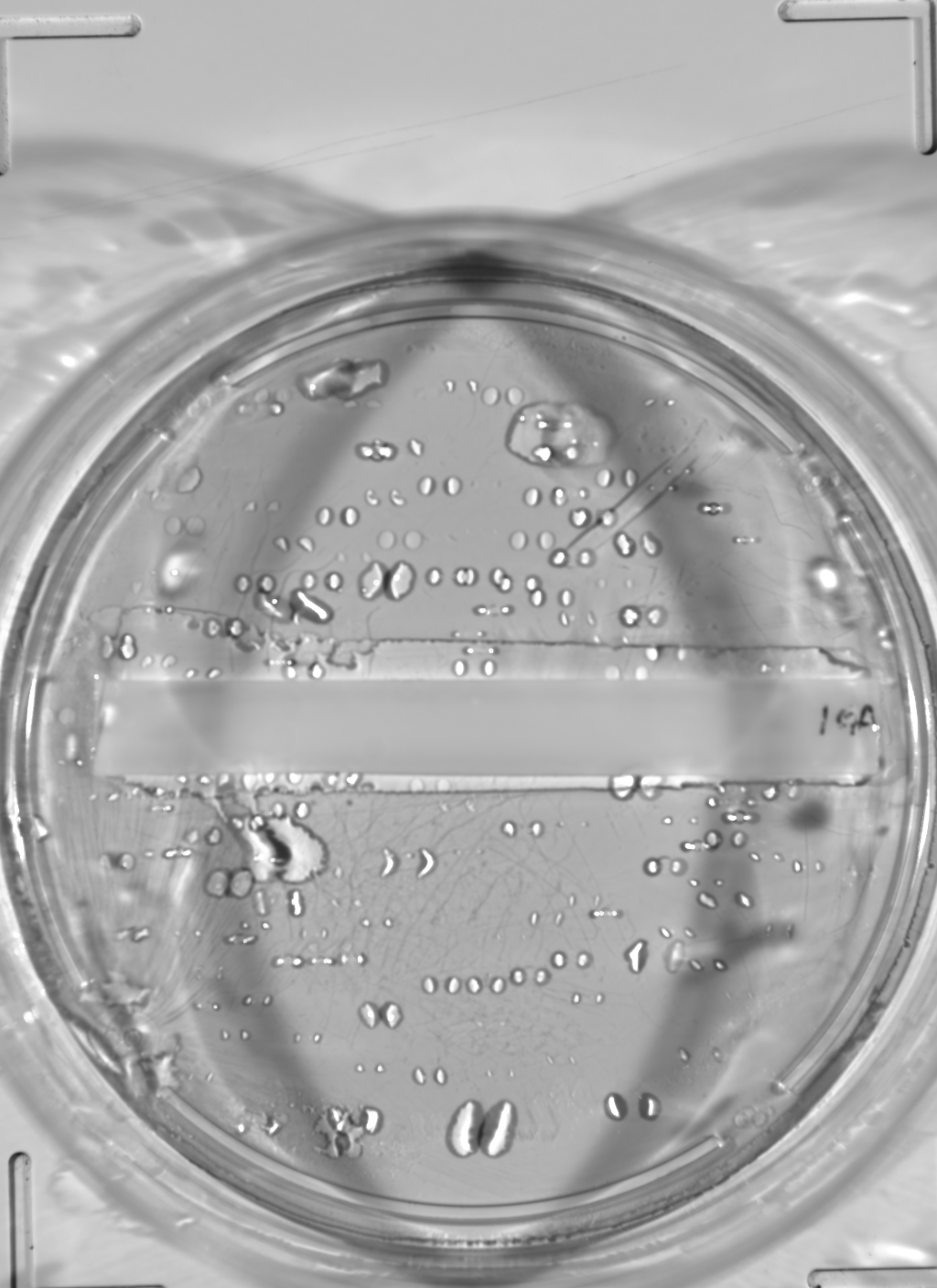

Supplement: Supplementary file 2 — Additional file 2. Raw data of western blot. [file 12974_2022_2632_MOESM2_ESM.zip › supplementary files/Figure3 WB/GAPDH WT/zkgapdh 2020.01.02_15.46.47_Ch-Marker.tif]

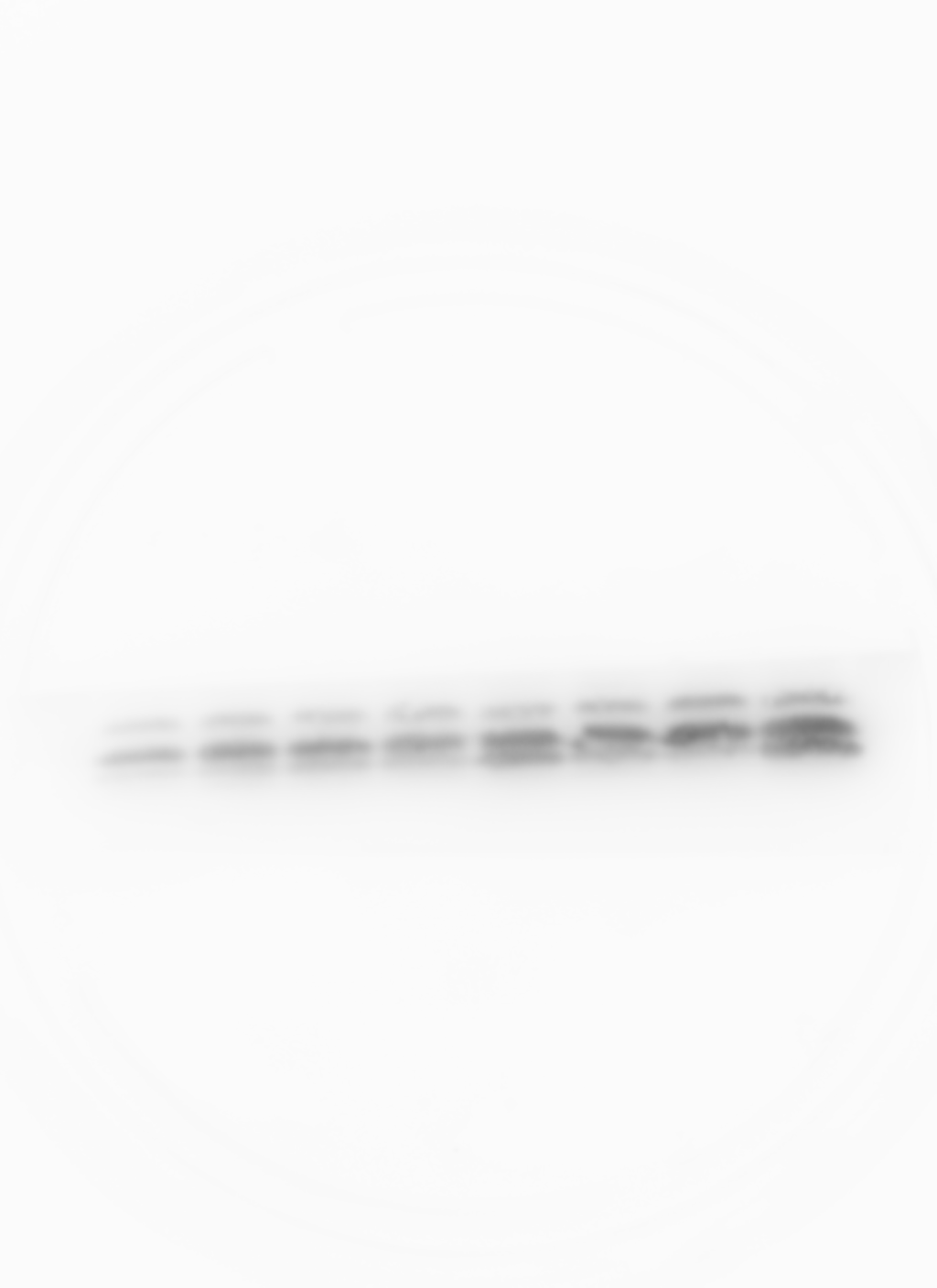

Supplement: Supplementary file 2 — Additional file 2. Raw data of western blot. [file 12974_2022_2632_MOESM2_ESM.zip › supplementary files/Figure3 WB/LC3 AR WB/zklc3 2020.01.02_16.07.35_Ch.tif]

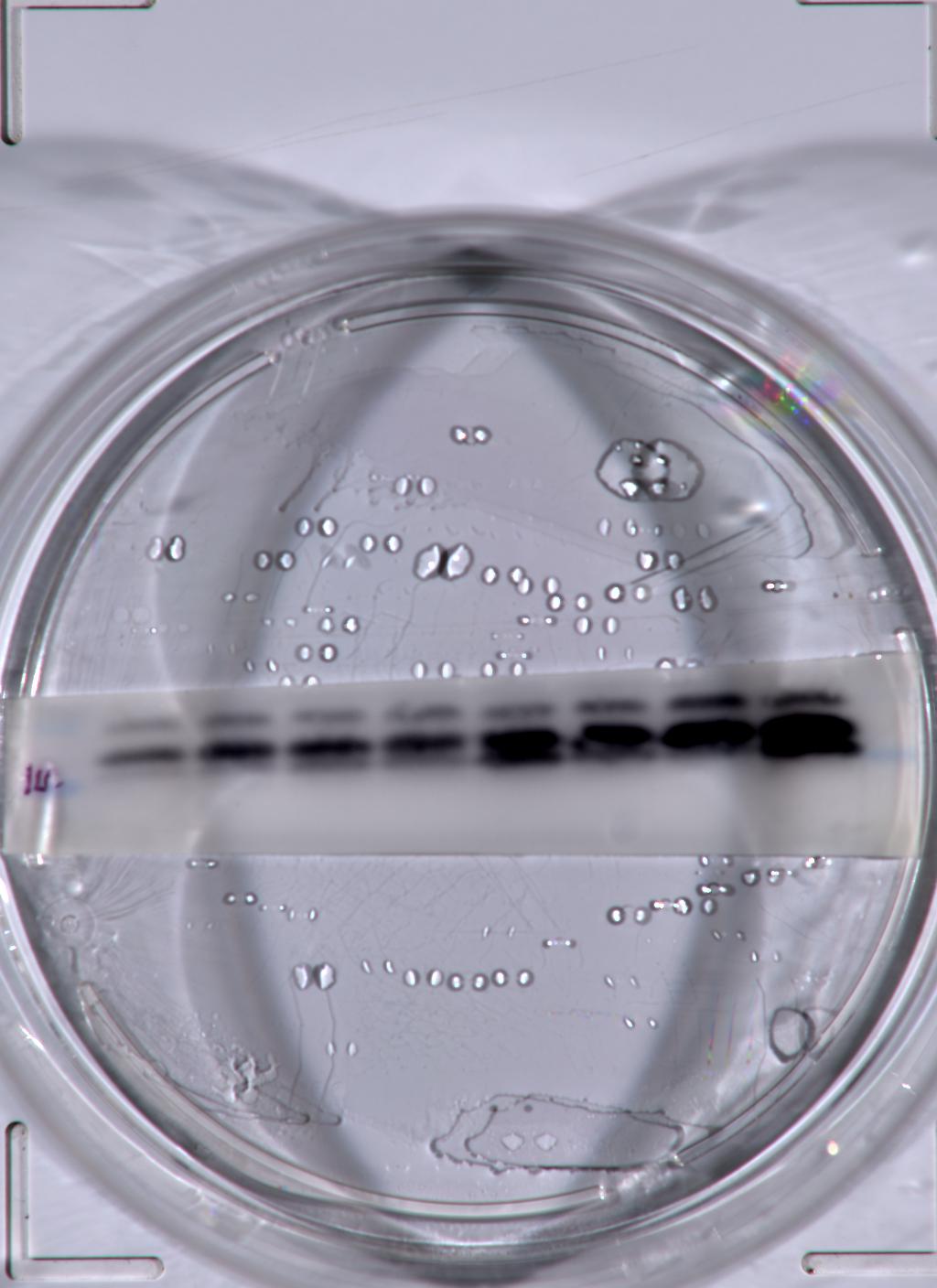

Supplement: Supplementary file 2 — Additional file 2. Raw data of western blot. [file 12974_2022_2632_MOESM2_ESM.zip › supplementary files/Figure3 WB/LC3 AR WB/zklc3 2020.01.02_16.07.35_Ch+Marker.jpg]

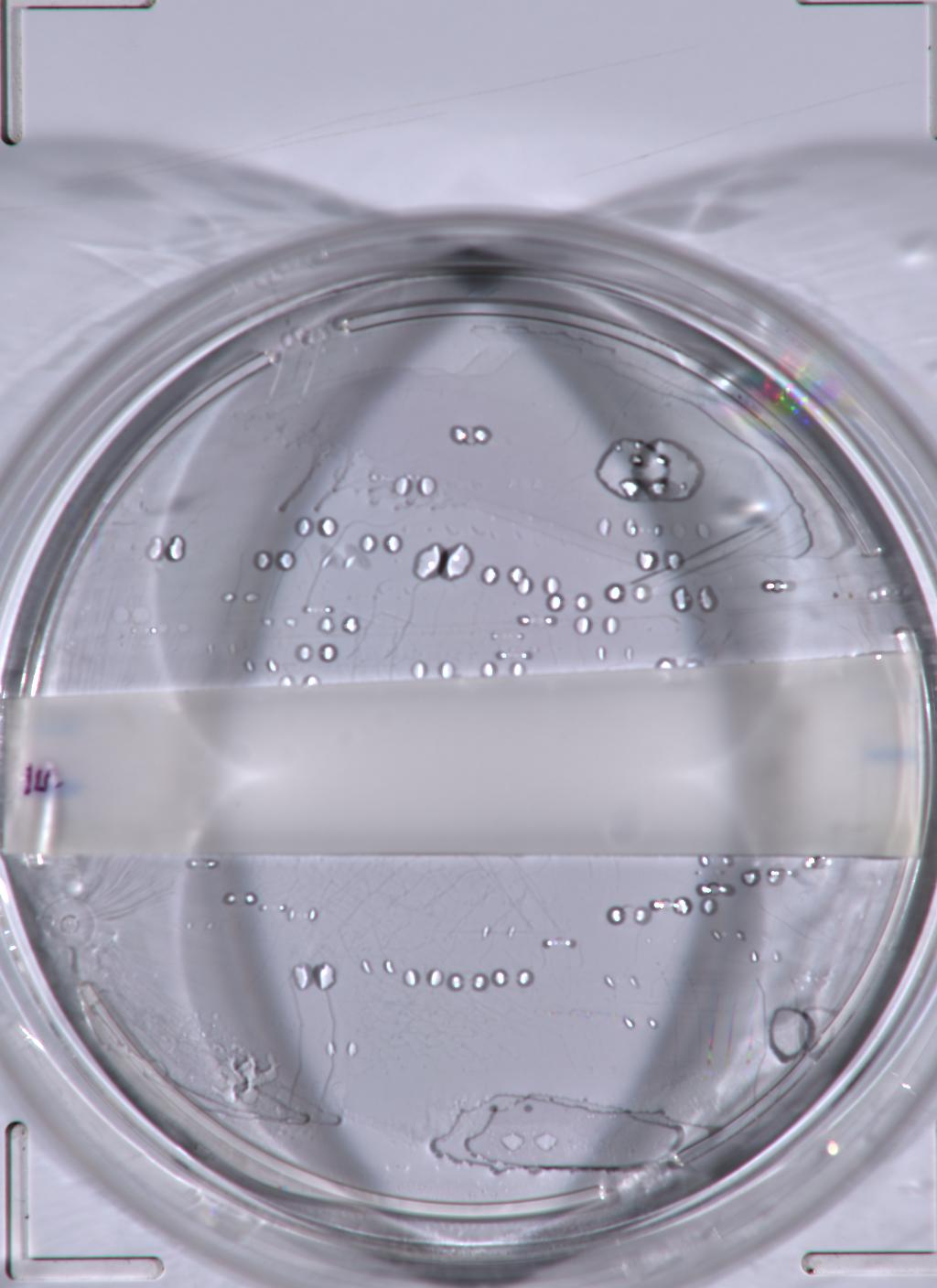

Supplement: Supplementary file 2 — Additional file 2. Raw data of western blot. [file 12974_2022_2632_MOESM2_ESM.zip › supplementary files/Figure3 WB/LC3 AR WB/zklc3 2020.01.02_16.07.35_Ch-Marker.jpg]

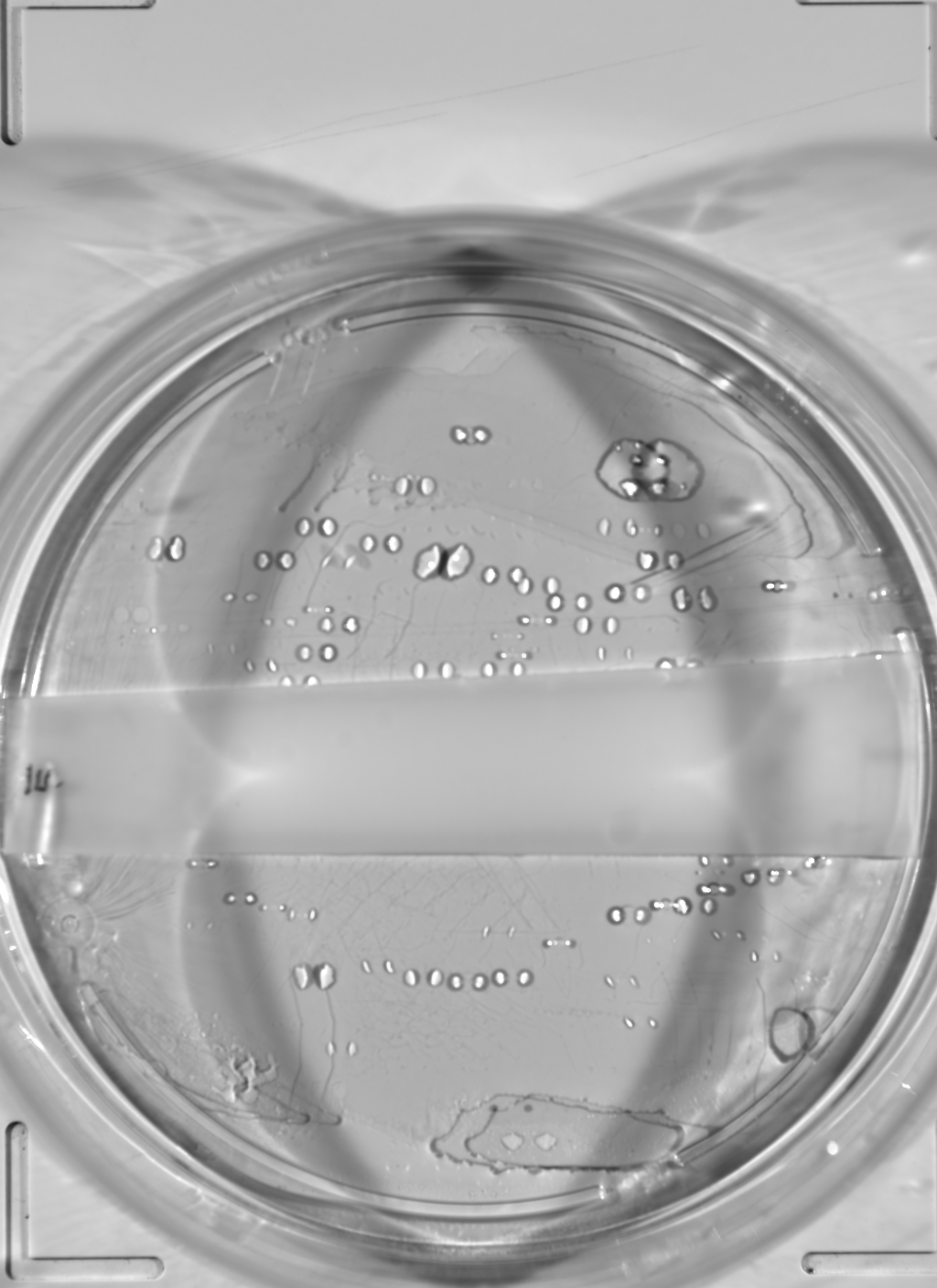

Supplement: Supplementary file 2 — Additional file 2. Raw data of western blot. [file 12974_2022_2632_MOESM2_ESM.zip › supplementary files/Figure3 WB/LC3 AR WB/zklc3 2020.01.02_16.07.35_Ch-Marker.tif]

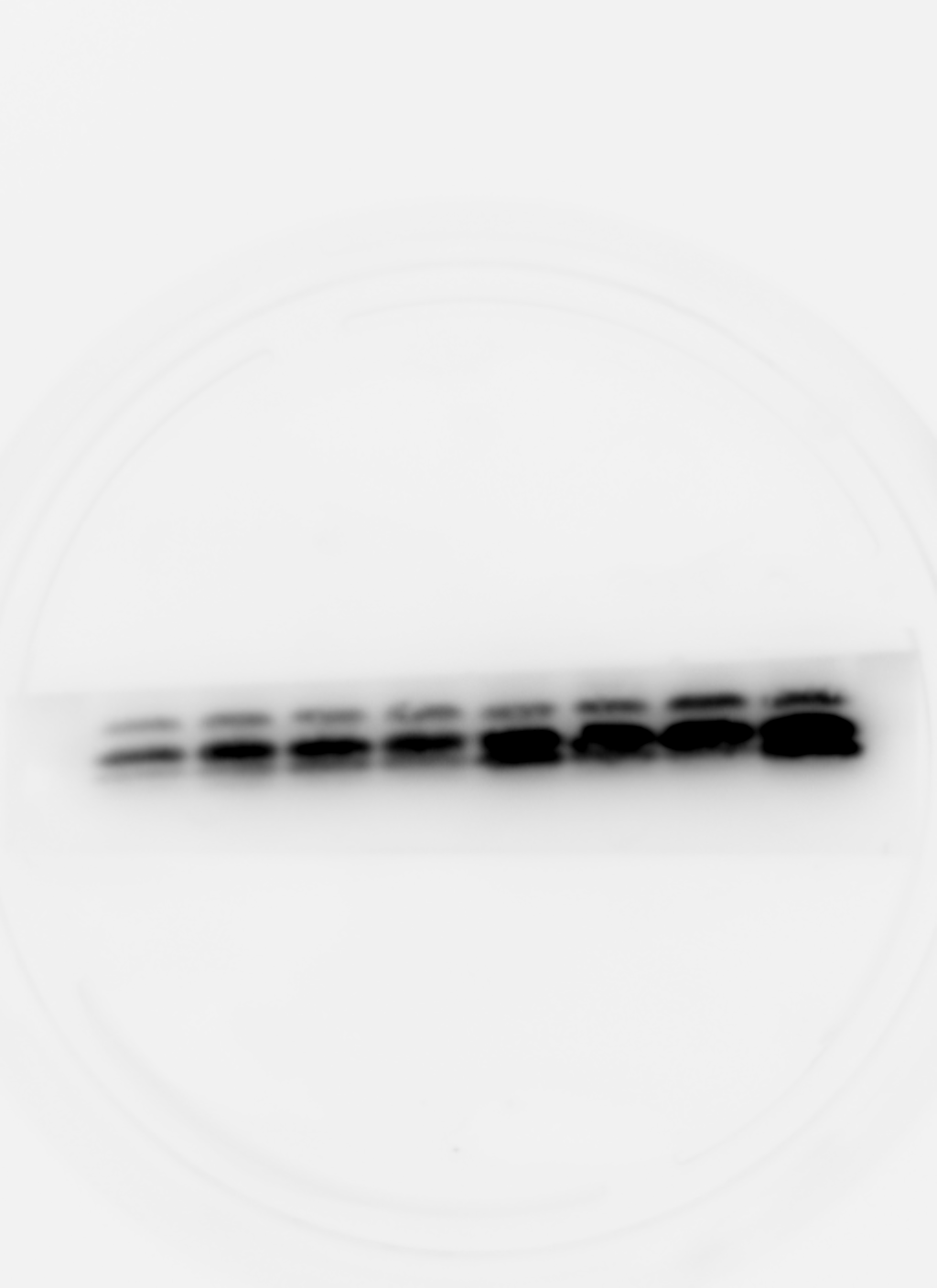

Supplement: Supplementary file 2 — Additional file 2. Raw data of western blot. [file 12974_2022_2632_MOESM2_ESM.zip › supplementary files/Figure3 WB/LC3 AR WB/zklc3 2020.01.02_16.07.35_Ch╨▐╕─.tif]

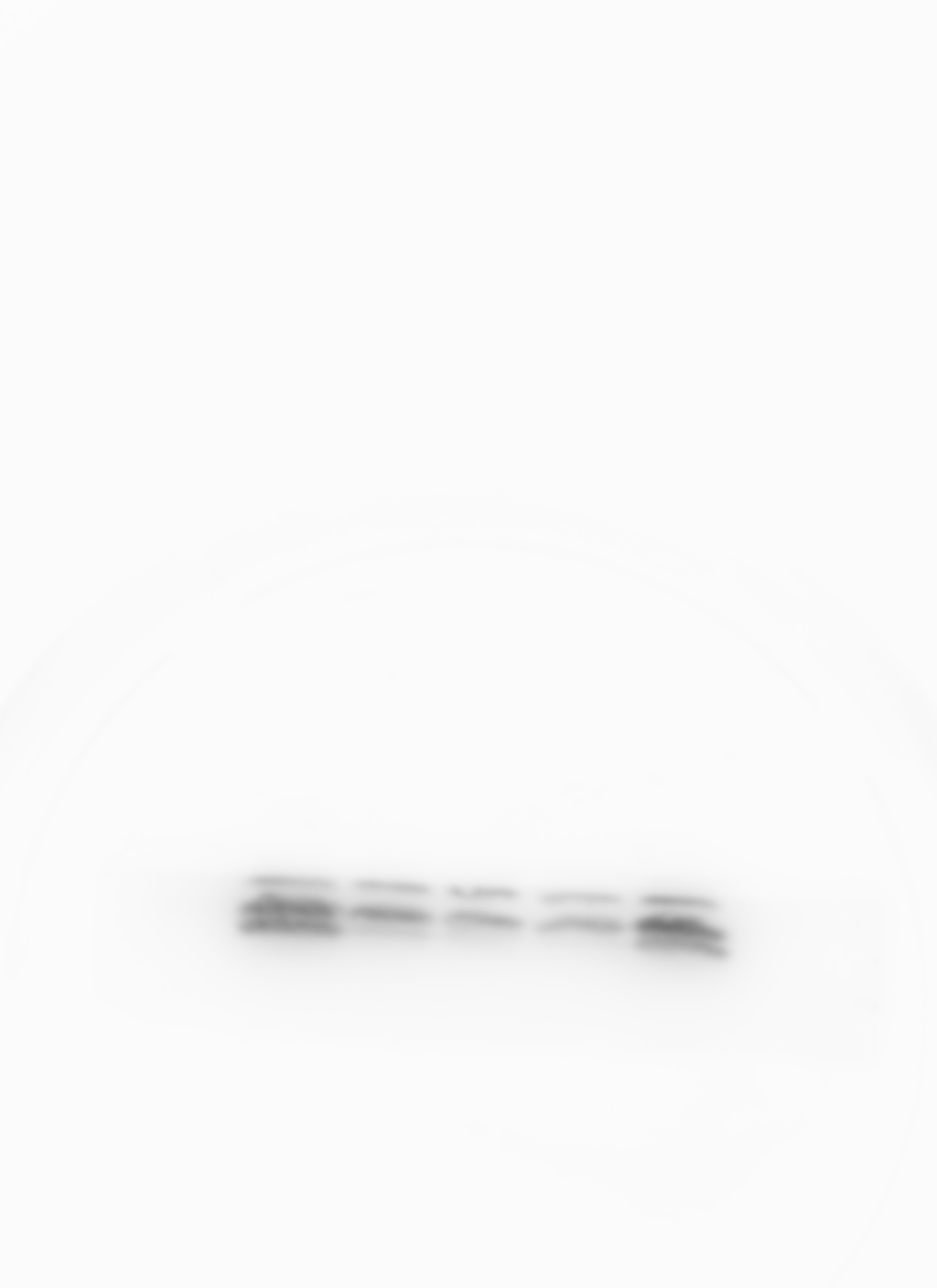

Supplement: Supplementary file 2 — Additional file 2. Raw data of western blot. [file 12974_2022_2632_MOESM2_ESM.zip › supplementary files/Figure3 WB/LC3 WT WB/zklc3 2020.01.02_15.50.31_Ch.tif]

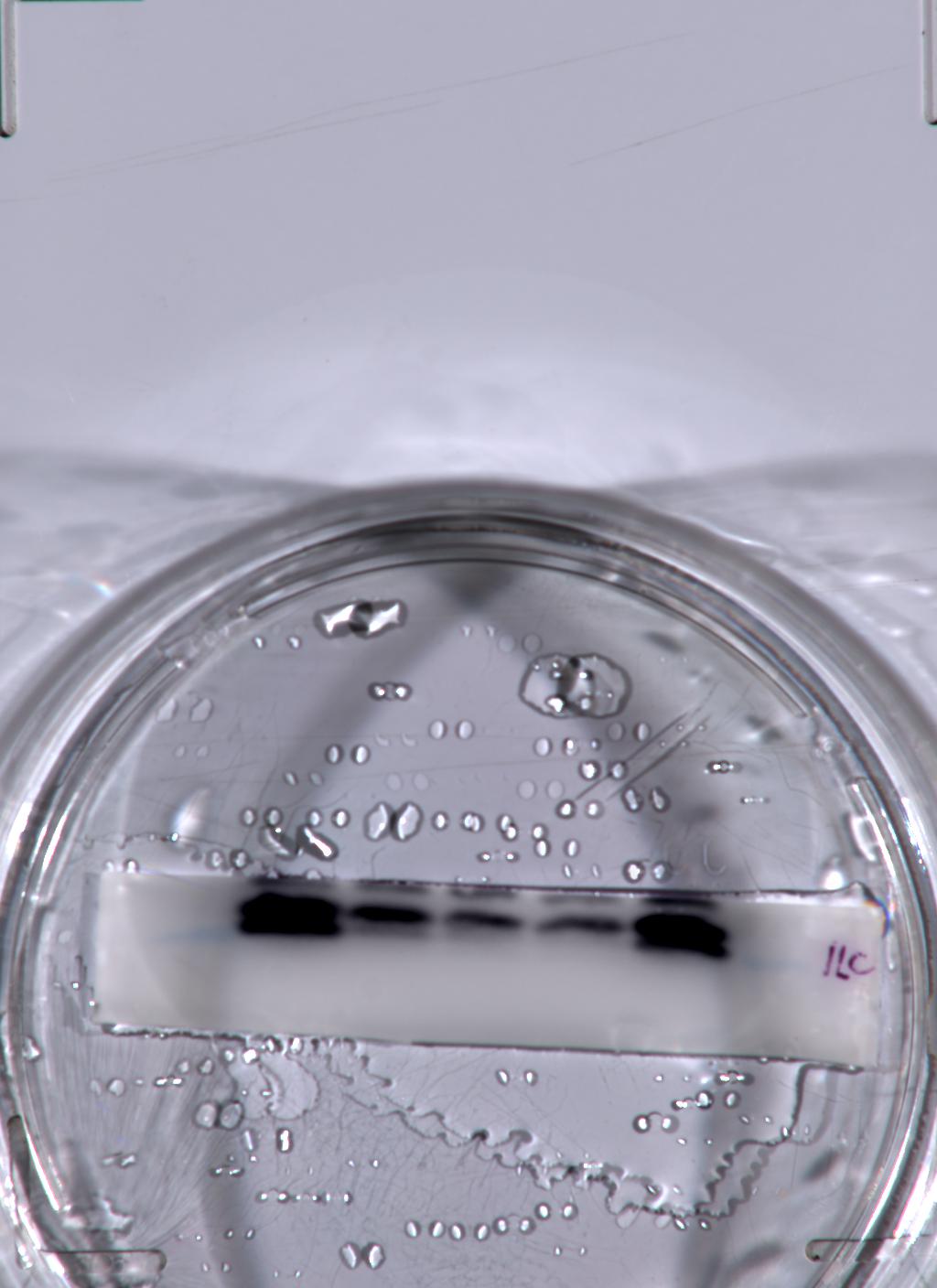

Supplement: Supplementary file 2 — Additional file 2. Raw data of western blot. [file 12974_2022_2632_MOESM2_ESM.zip › supplementary files/Figure3 WB/LC3 WT WB/zklc3 2020.01.02_15.50.31_Ch+Marker.jpg]

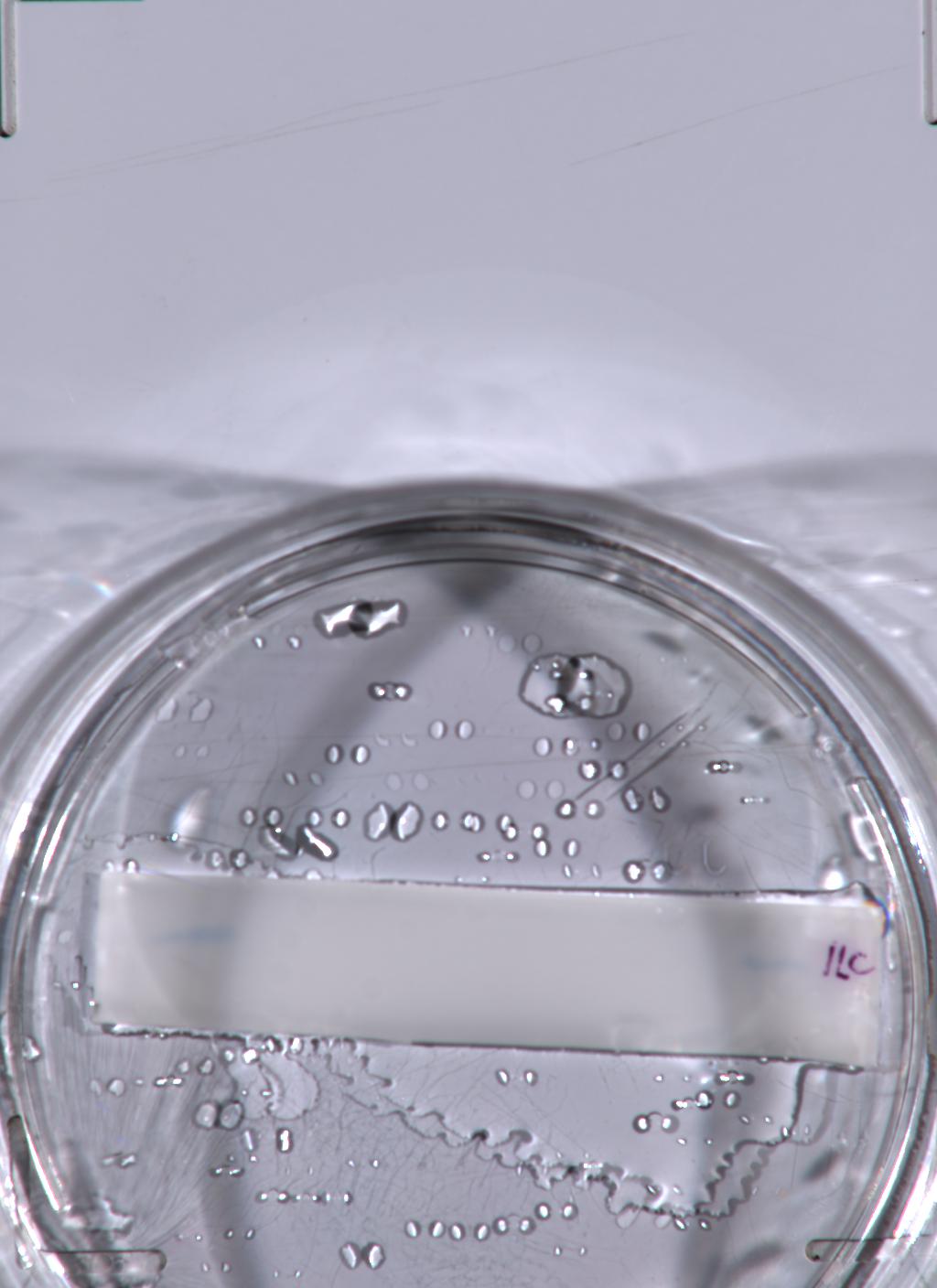

Supplement: Supplementary file 2 — Additional file 2. Raw data of western blot. [file 12974_2022_2632_MOESM2_ESM.zip › supplementary files/Figure3 WB/LC3 WT WB/zklc3 2020.01.02_15.50.31_Ch-Marker.jpg]

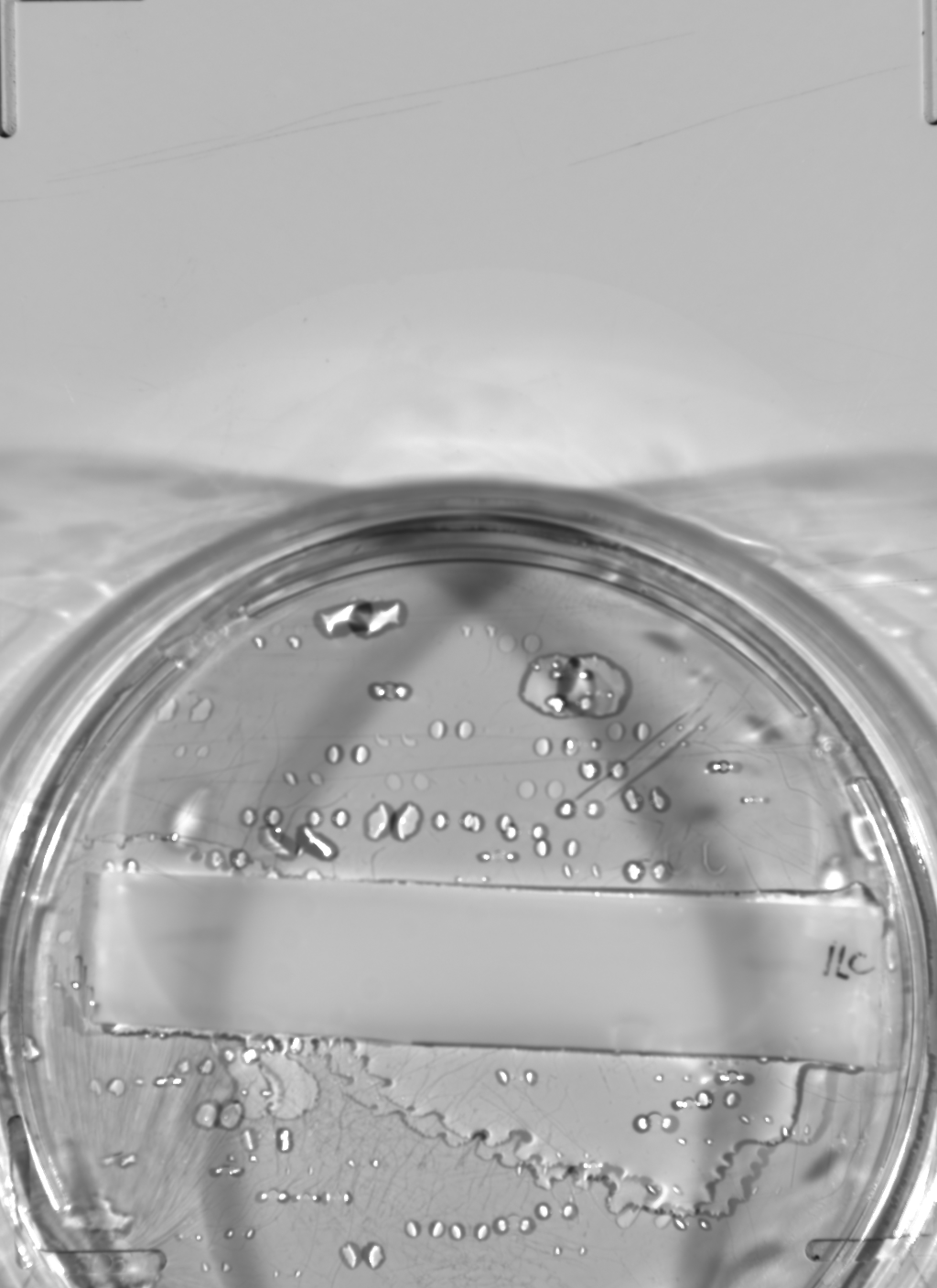

Supplement: Supplementary file 2 — Additional file 2. Raw data of western blot. [file 12974_2022_2632_MOESM2_ESM.zip › supplementary files/Figure3 WB/LC3 WT WB/zklc3 2020.01.02_15.50.31_Ch-Marker.tif]

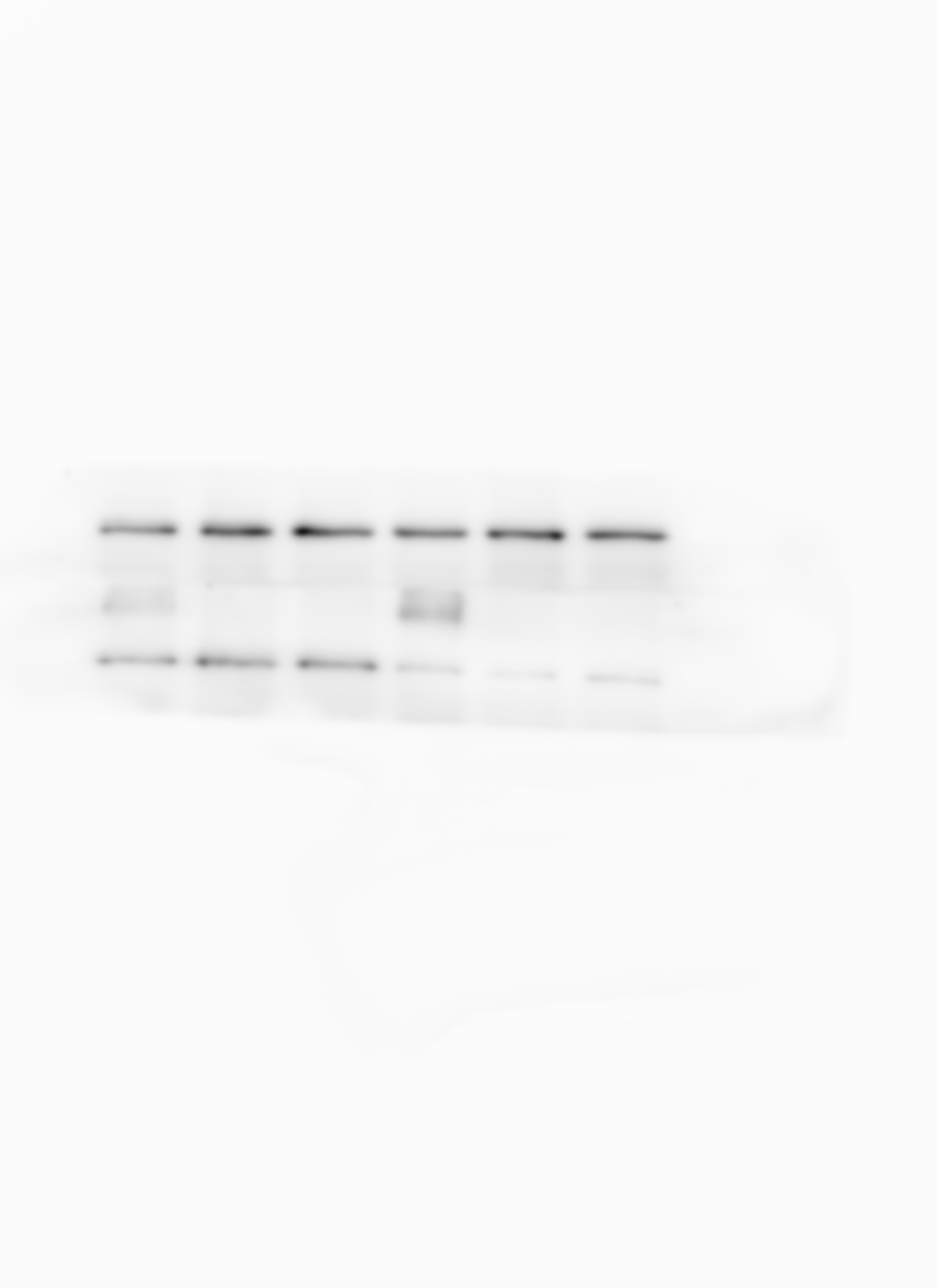

Supplement: Supplementary file 2 — Additional file 2. Raw data of western blot. [file 12974_2022_2632_MOESM2_ESM.zip › supplementary files/Figure3 WB/p62 WT AR WB/p62 2020.09.18_12.20.35_Ch.tif]

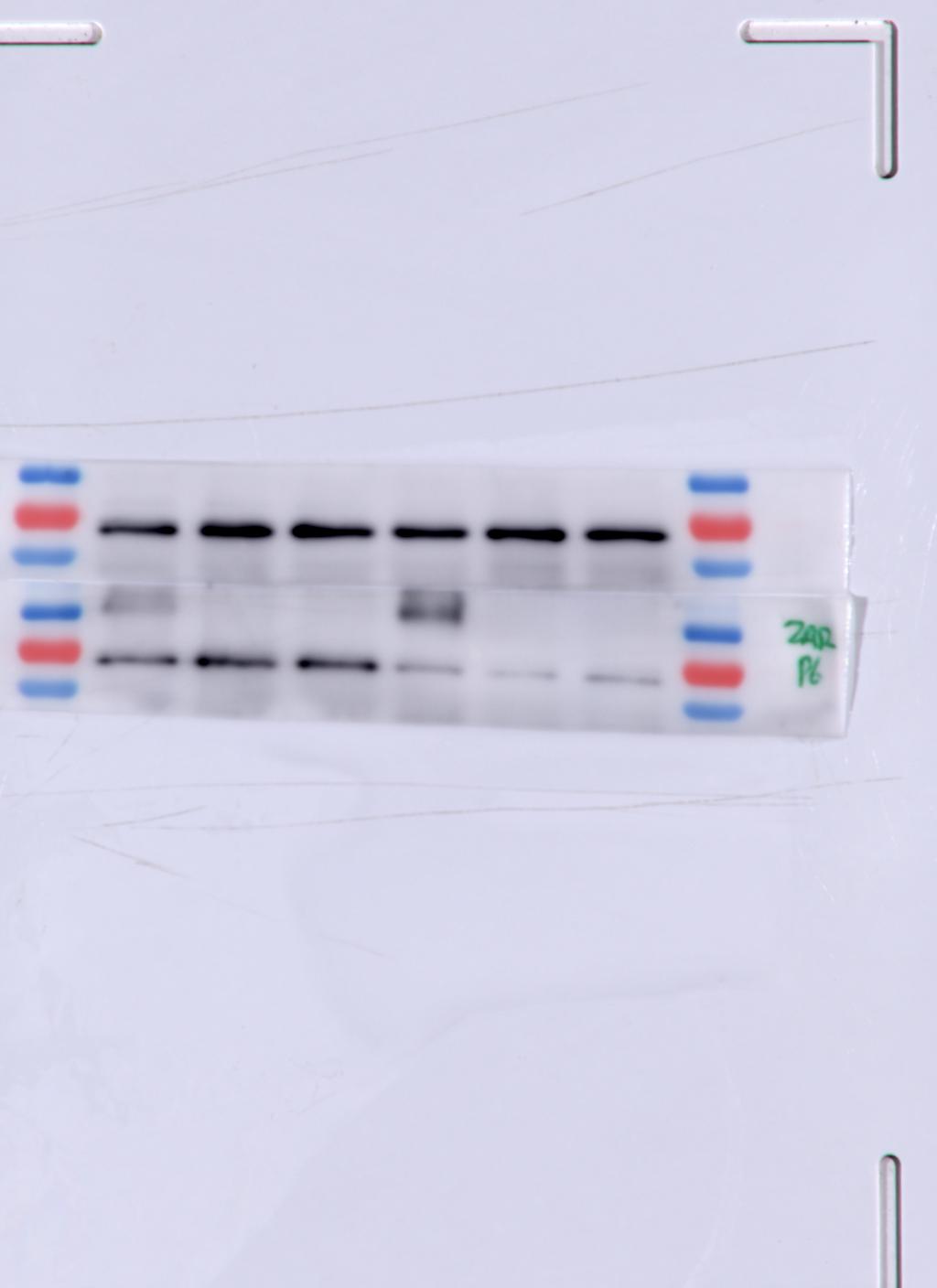

Supplement: Supplementary file 2 — Additional file 2. Raw data of western blot. [file 12974_2022_2632_MOESM2_ESM.zip › supplementary files/Figure3 WB/p62 WT AR WB/p62 2020.09.18_12.20.35_Ch+Marker.jpg]

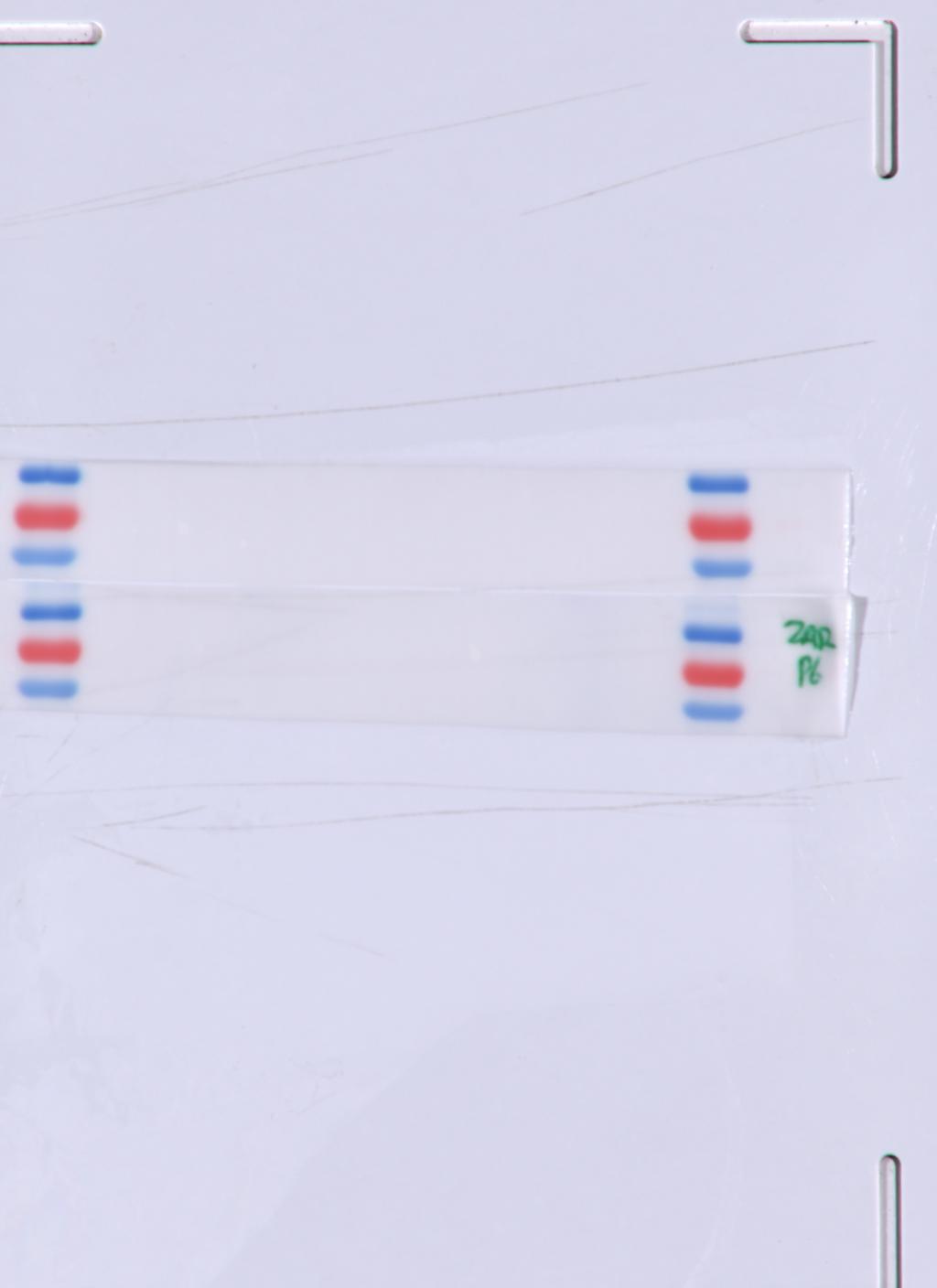

Supplement: Supplementary file 2 — Additional file 2. Raw data of western blot. [file 12974_2022_2632_MOESM2_ESM.zip › supplementary files/Figure3 WB/p62 WT AR WB/p62 2020.09.18_12.20.35_Ch-Marker.jpg]

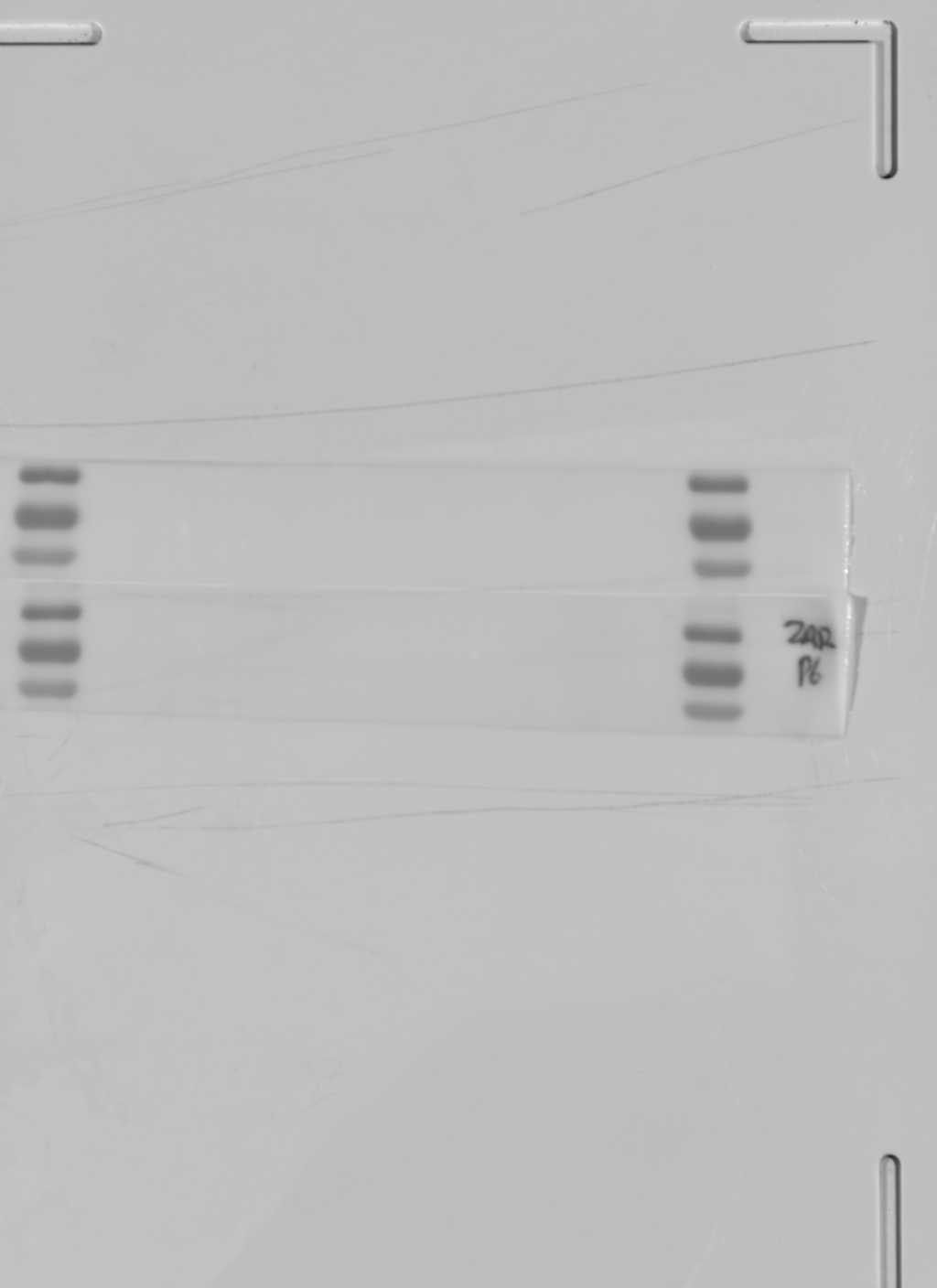

Supplement: Supplementary file 2 — Additional file 2. Raw data of western blot. [file 12974_2022_2632_MOESM2_ESM.zip › supplementary files/Figure3 WB/p62 WT AR WB/p62 2020.09.18_12.20.35_Ch-Marker.tif]

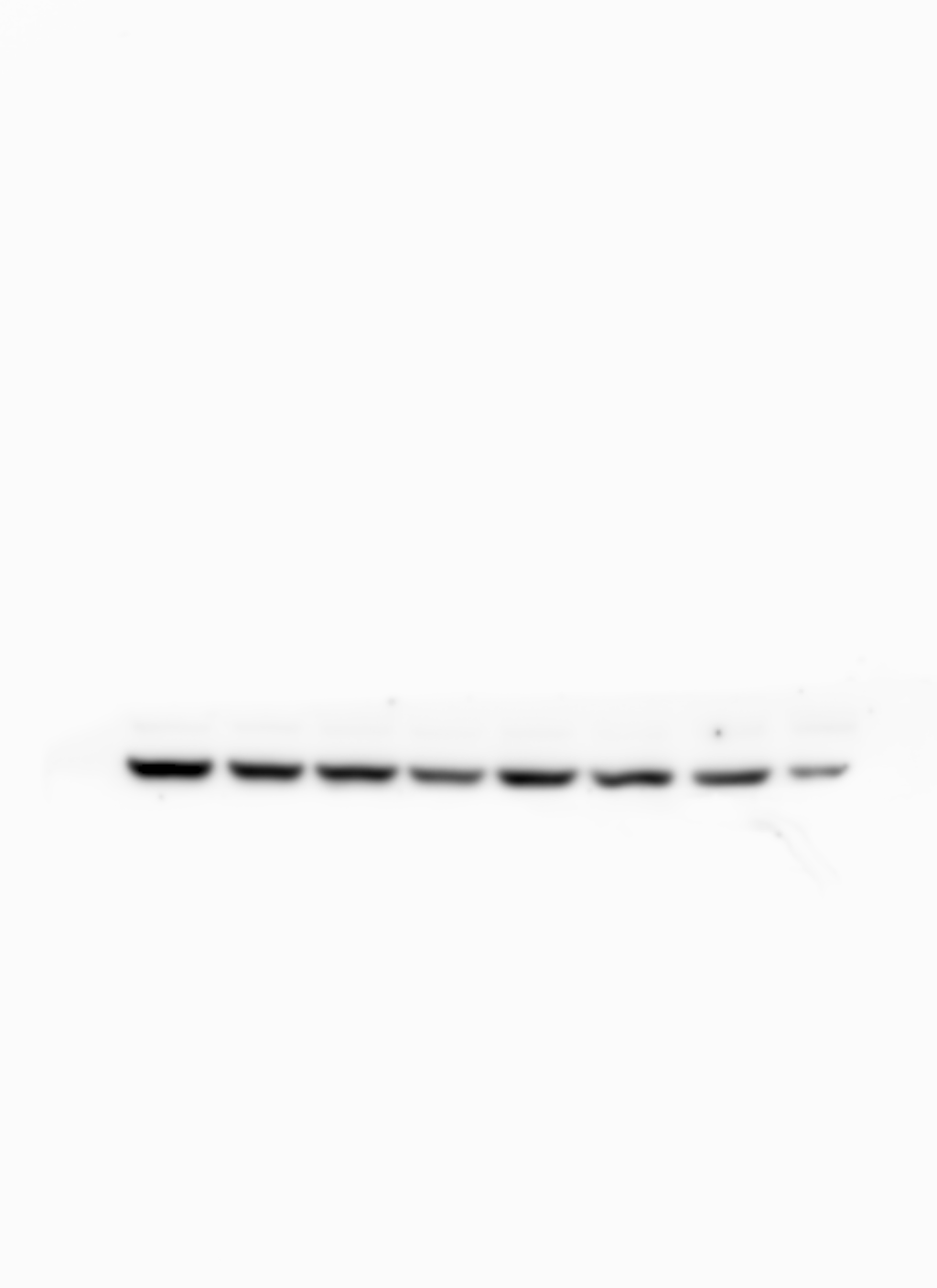

Supplement: Supplementary file 2 — Additional file 2. Raw data of western blot. [file 12974_2022_2632_MOESM2_ESM.zip › supplementary files/Figure5 WB/AR AMPK/4ampk 2020.01.18_12.49.18_Ch.tif]

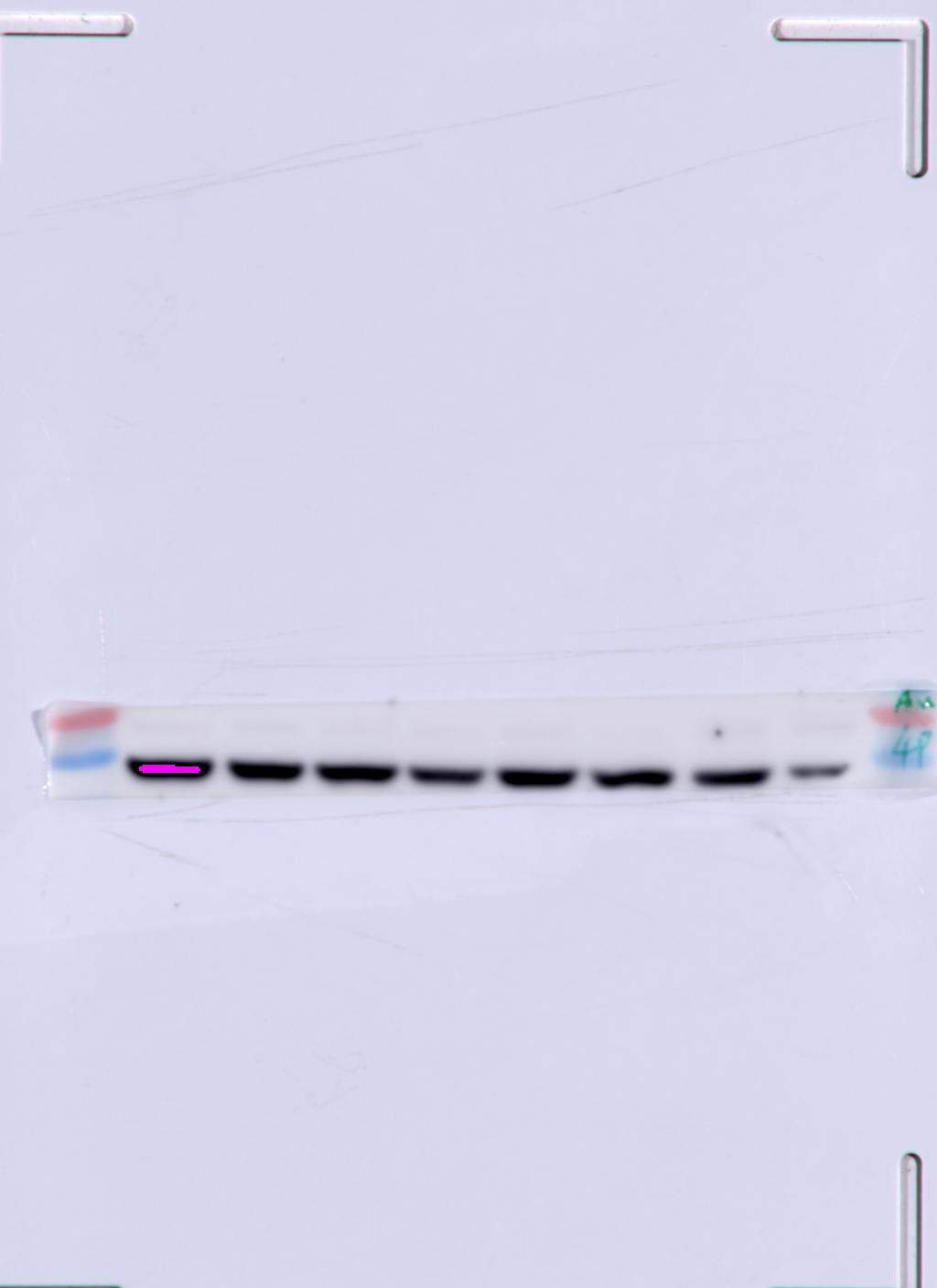

Supplement: Supplementary file 2 — Additional file 2. Raw data of western blot. [file 12974_2022_2632_MOESM2_ESM.zip › supplementary files/Figure5 WB/AR AMPK/4ampk 2020.01.18_12.49.18_Ch+Marker.jpg]

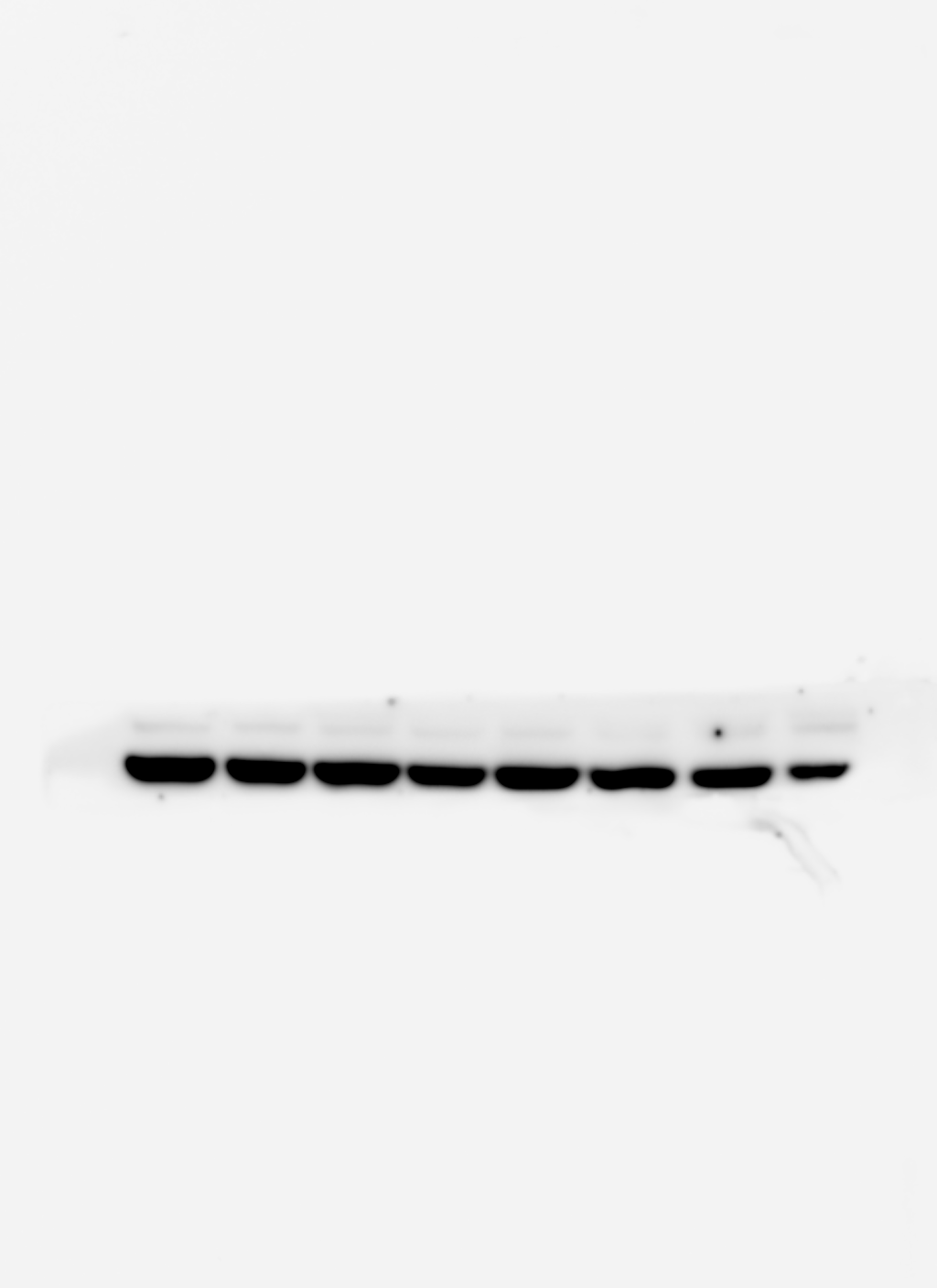

Supplement: Supplementary file 2 — Additional file 2. Raw data of western blot. [file 12974_2022_2632_MOESM2_ESM.zip › supplementary files/Figure5 WB/AR AMPK/4ampk 2020.01.18_12.49.18_Ch-1.tif]

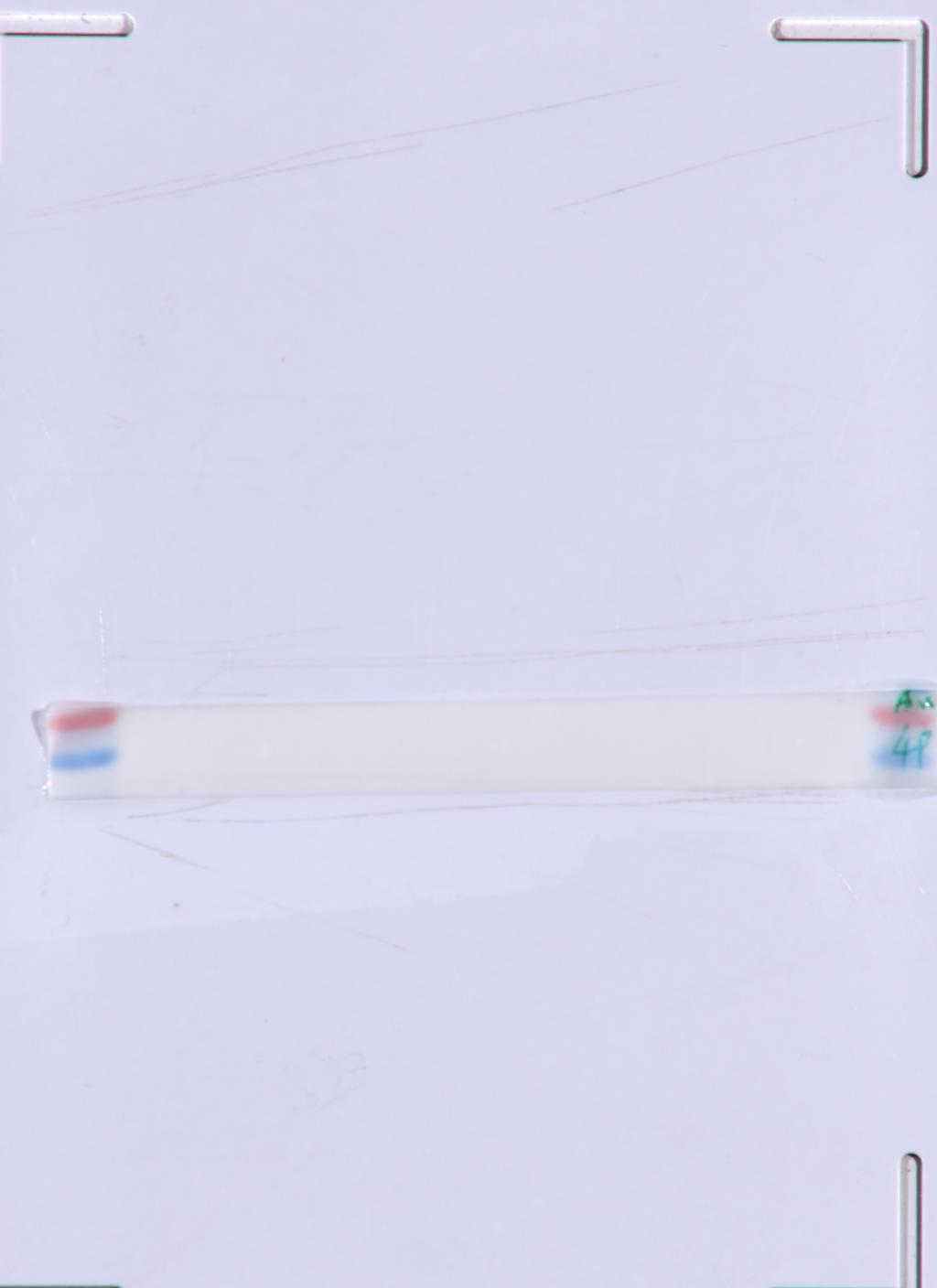

Supplement: Supplementary file 2 — Additional file 2. Raw data of western blot. [file 12974_2022_2632_MOESM2_ESM.zip › supplementary files/Figure5 WB/AR AMPK/4ampk 2020.01.18_12.49.18_Ch-Marker.jpg]

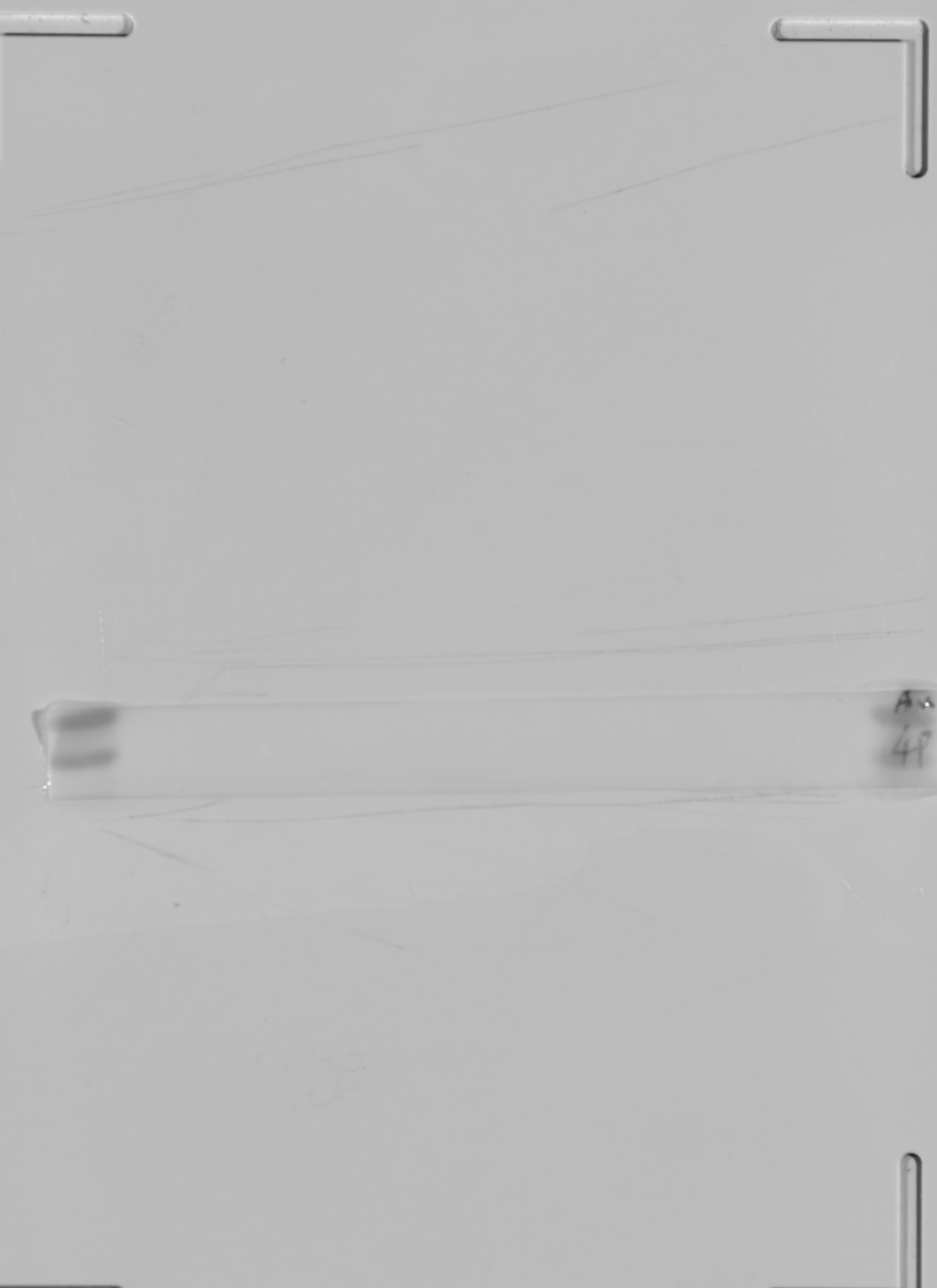

Supplement: Supplementary file 2 — Additional file 2. Raw data of western blot. [file 12974_2022_2632_MOESM2_ESM.zip › supplementary files/Figure5 WB/AR AMPK/4ampk 2020.01.18_12.49.18_Ch-Marker.tif]

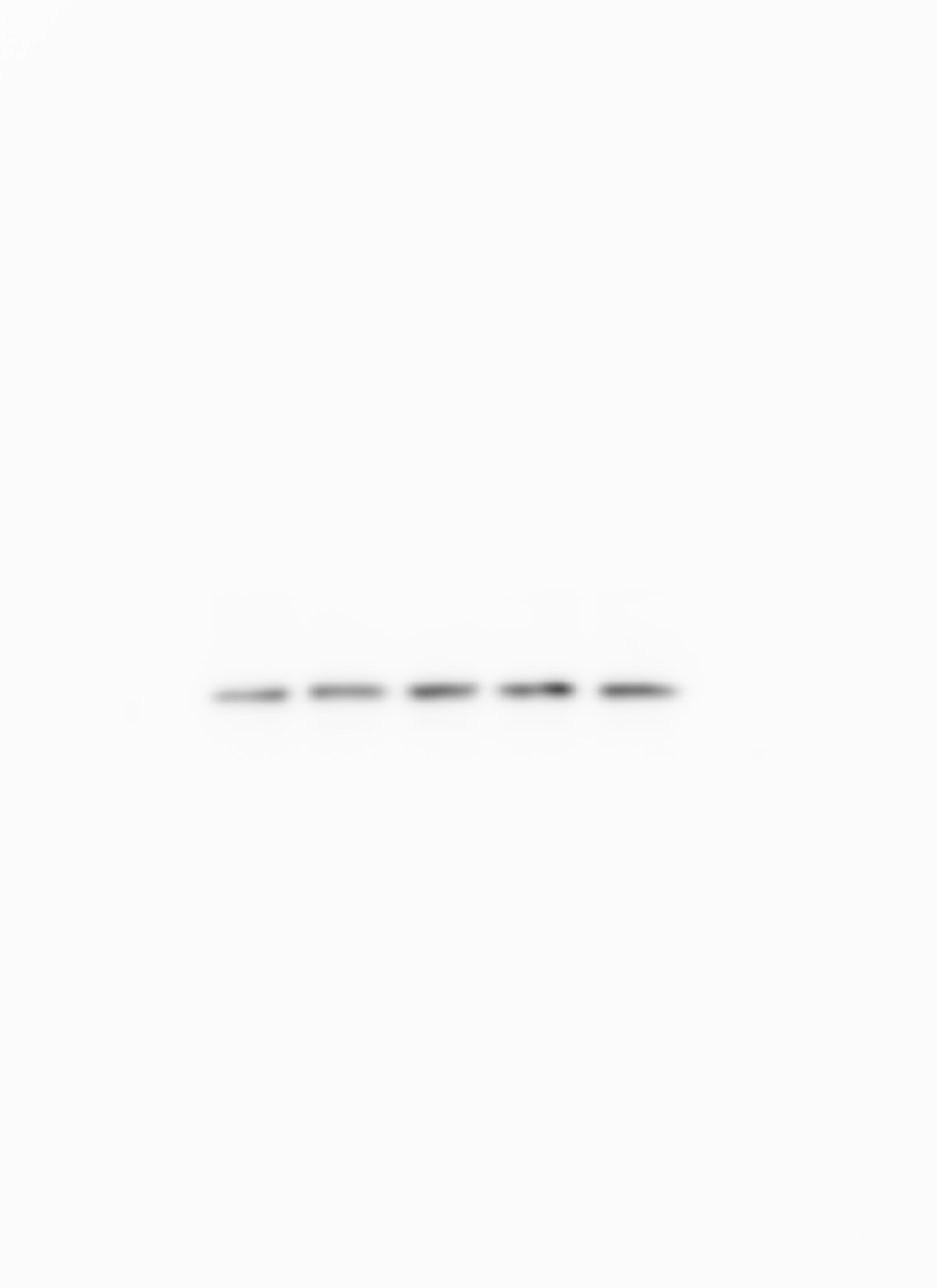

Supplement: Supplementary file 2 — Additional file 2. Raw data of western blot. [file 12974_2022_2632_MOESM2_ESM.zip › supplementary files/Figure5 WB/AR GAPDH/4gapdh 2021.04.24_16.59.31_Ch.tif]

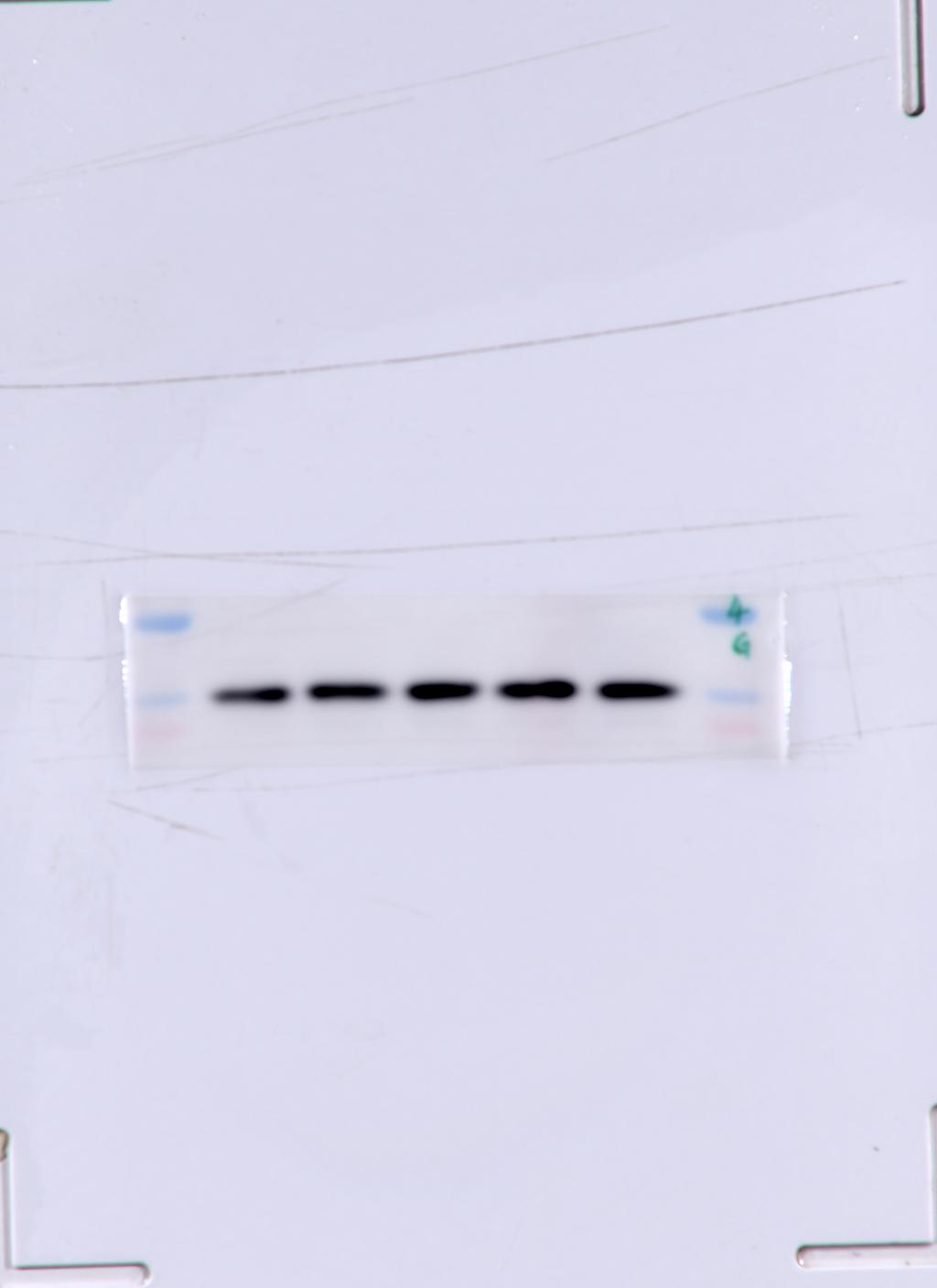

Supplement: Supplementary file 2 — Additional file 2. Raw data of western blot. [file 12974_2022_2632_MOESM2_ESM.zip › supplementary files/Figure5 WB/AR GAPDH/4gapdh 2021.04.24_16.59.31_Ch+Marker.jpg]

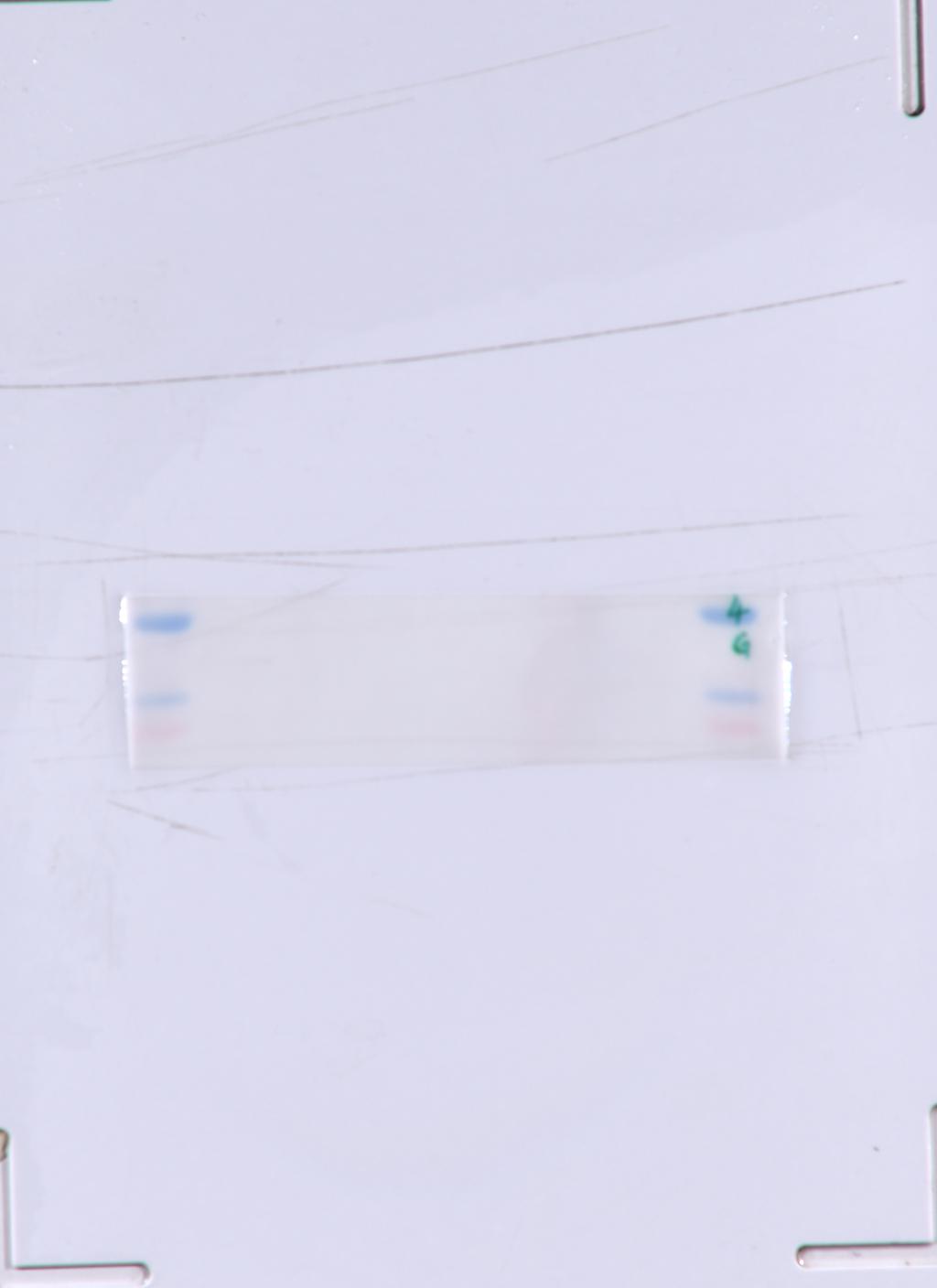

Supplement: Supplementary file 2 — Additional file 2. Raw data of western blot. [file 12974_2022_2632_MOESM2_ESM.zip › supplementary files/Figure5 WB/AR GAPDH/4gapdh 2021.04.24_16.59.31_Ch-Marker.jpg]

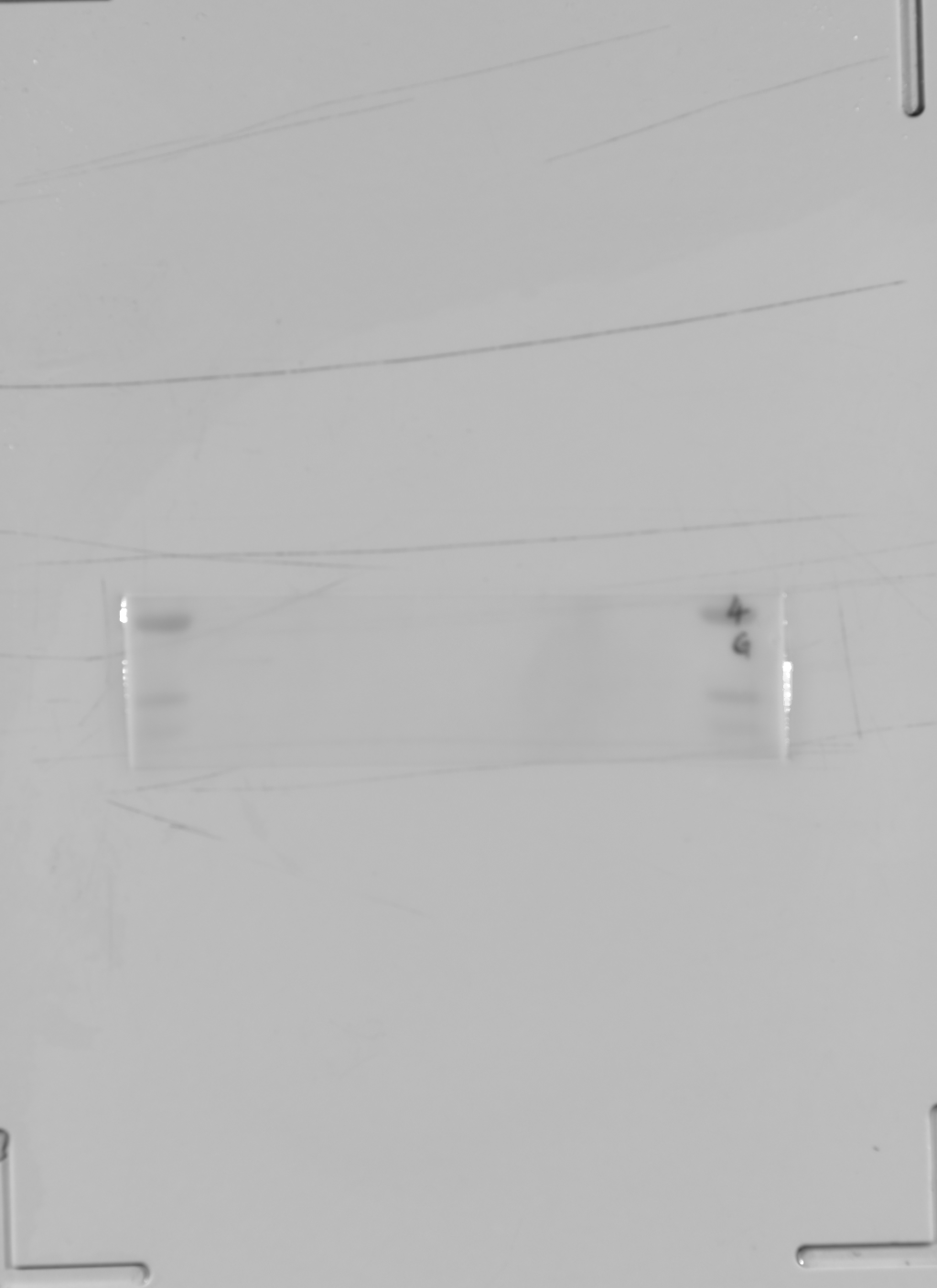

Supplement: Supplementary file 2 — Additional file 2. Raw data of western blot. [file 12974_2022_2632_MOESM2_ESM.zip › supplementary files/Figure5 WB/AR GAPDH/4gapdh 2021.04.24_16.59.31_Ch-Marker.tif]
