# Supplementary figures and images for: Motor neuron survival is associated with reduced neuroinflammation and increased autophagy after brachial plexus avulsion injury in aldose reductase-deficient mice (part 2 of 2)
Source: J Neuroinflammation. 2022 Nov 9;19:271. doi: 10.1186/s12974-022-02632-6 (PMC9648007; doi:10.1186/s12974-022-02632-6)

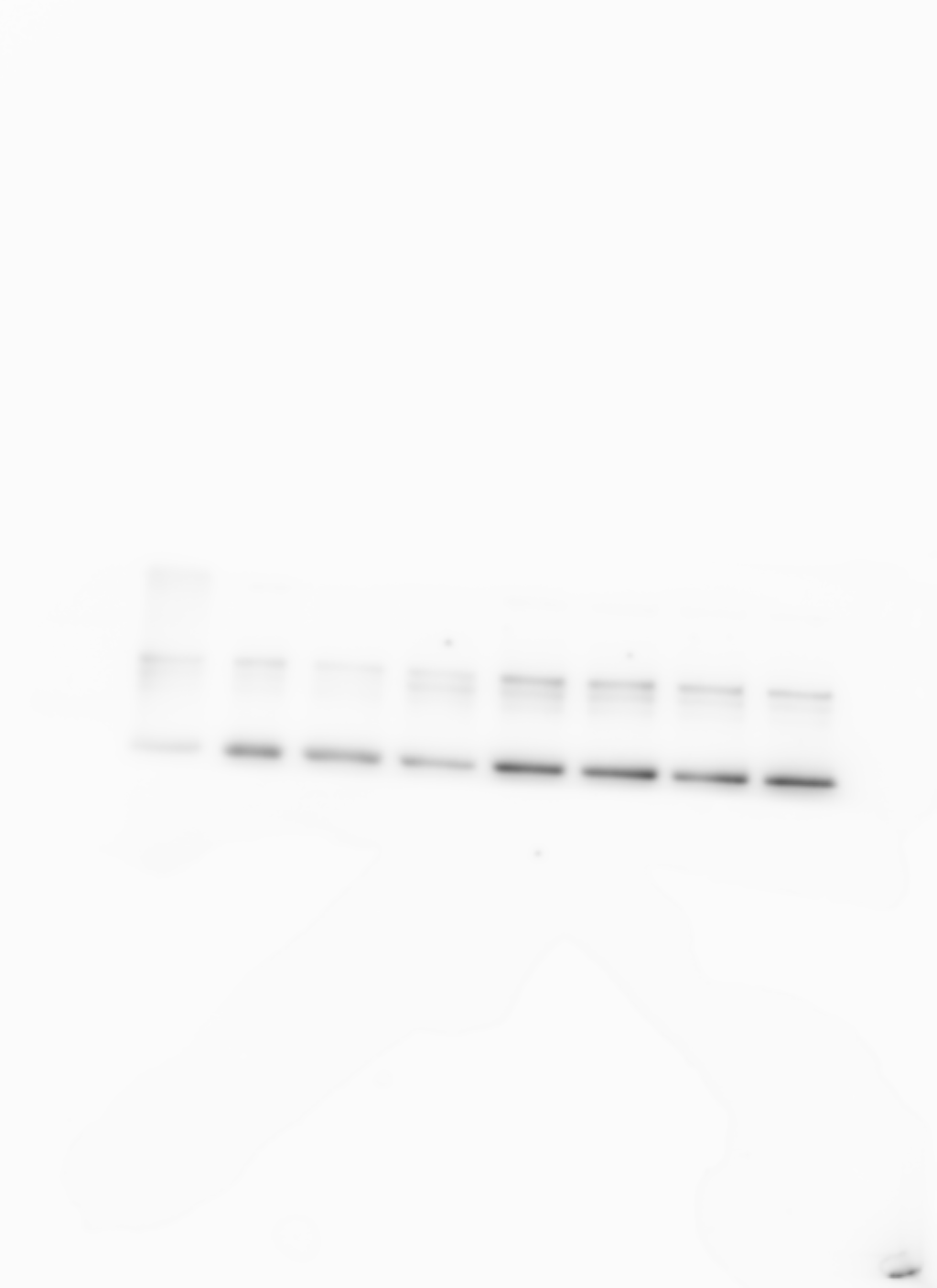

Supplement: Supplementary file 2 — Additional file 2. Raw data of western blot. [file 12974_2022_2632_MOESM2_ESM.zip › supplementary files/Figure5 WB/AR SIRT1/2020.08.05_16.52.44_Ch.tif]

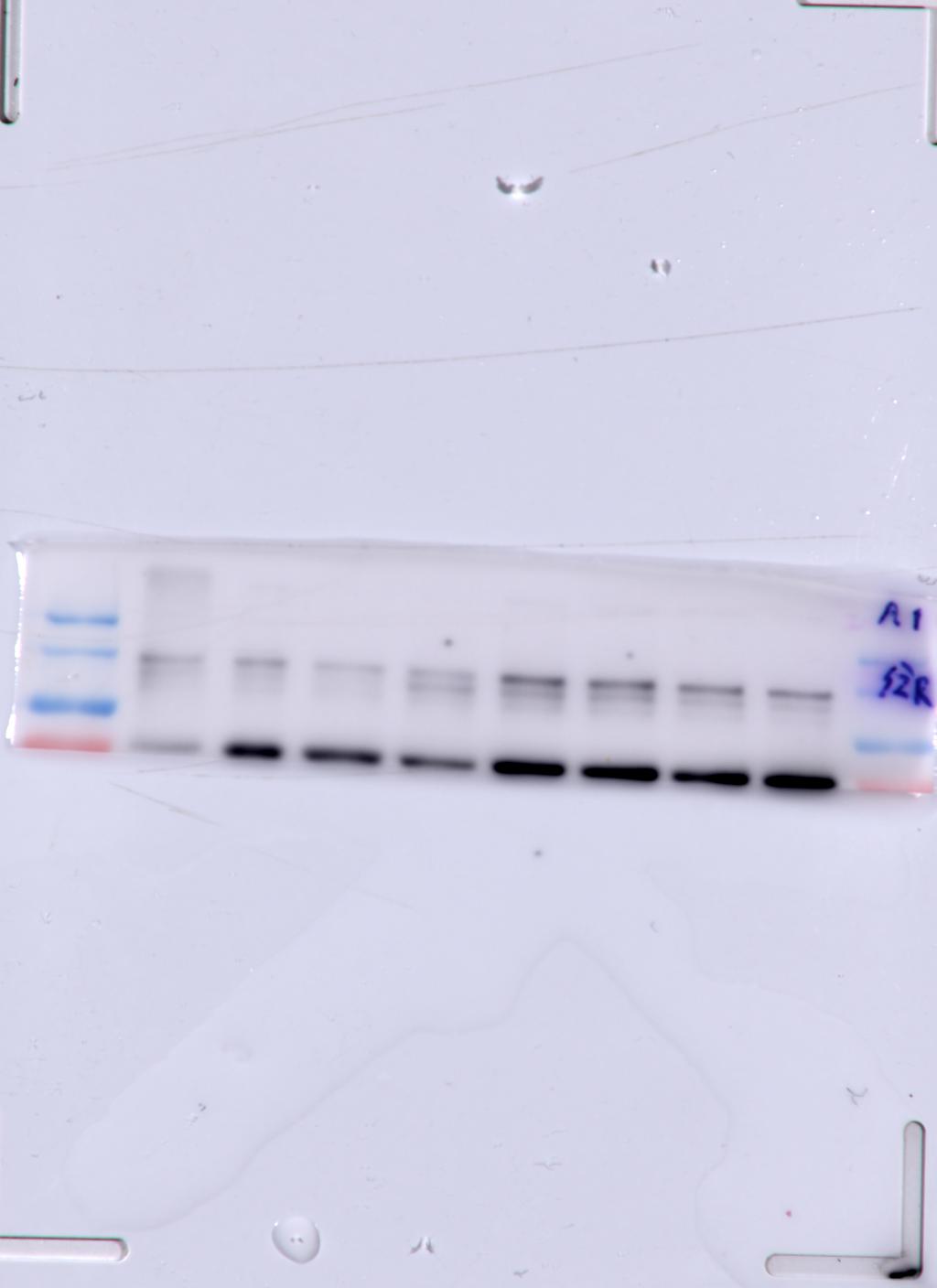

Supplement: Supplementary file 2 — Additional file 2. Raw data of western blot. [file 12974_2022_2632_MOESM2_ESM.zip › supplementary files/Figure5 WB/AR SIRT1/2020.08.05_16.52.44_Ch+Marker.jpg]

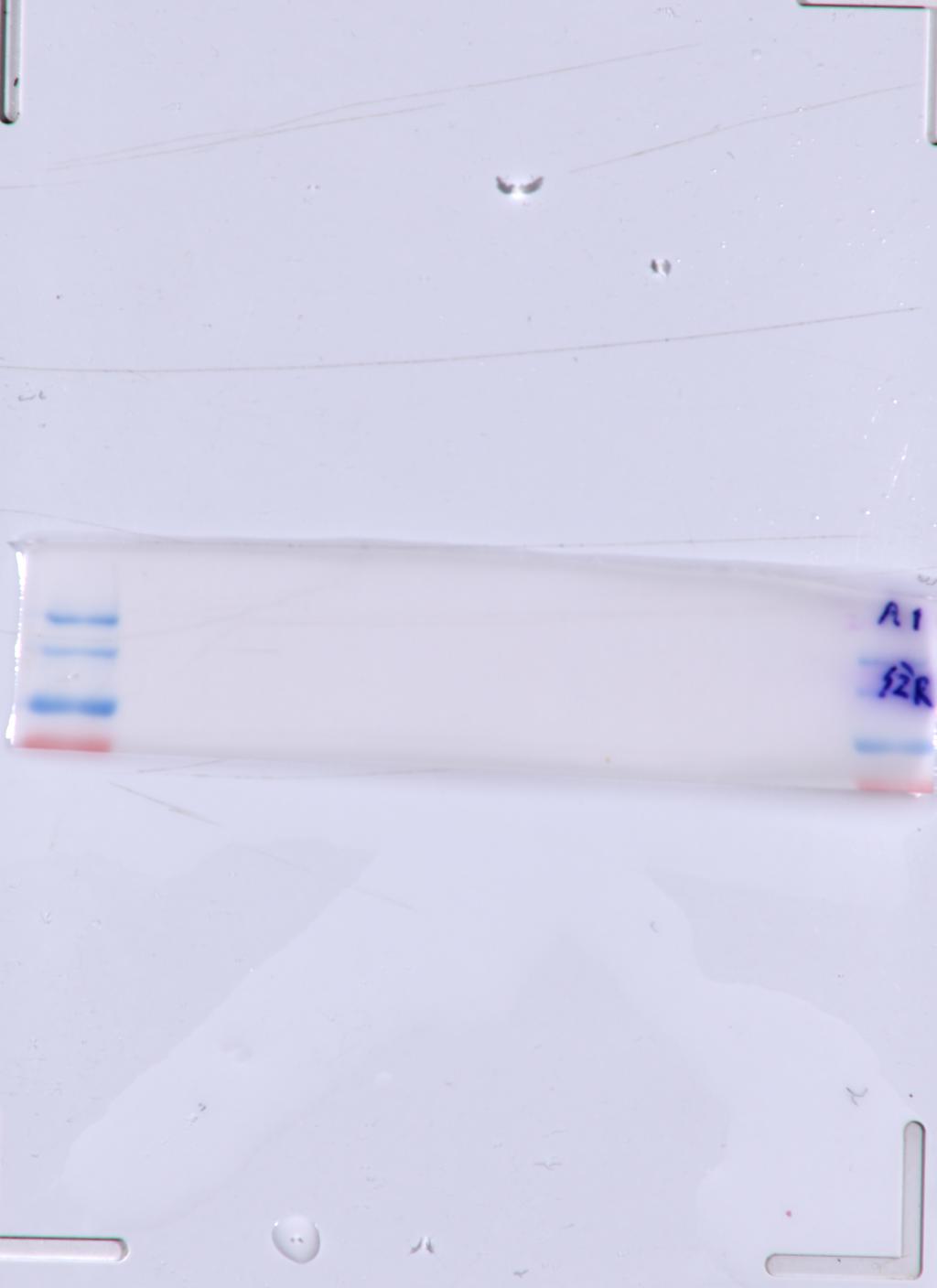

Supplement: Supplementary file 2 — Additional file 2. Raw data of western blot. [file 12974_2022_2632_MOESM2_ESM.zip › supplementary files/Figure5 WB/AR SIRT1/2020.08.05_16.52.44_Ch-Marker.jpg]

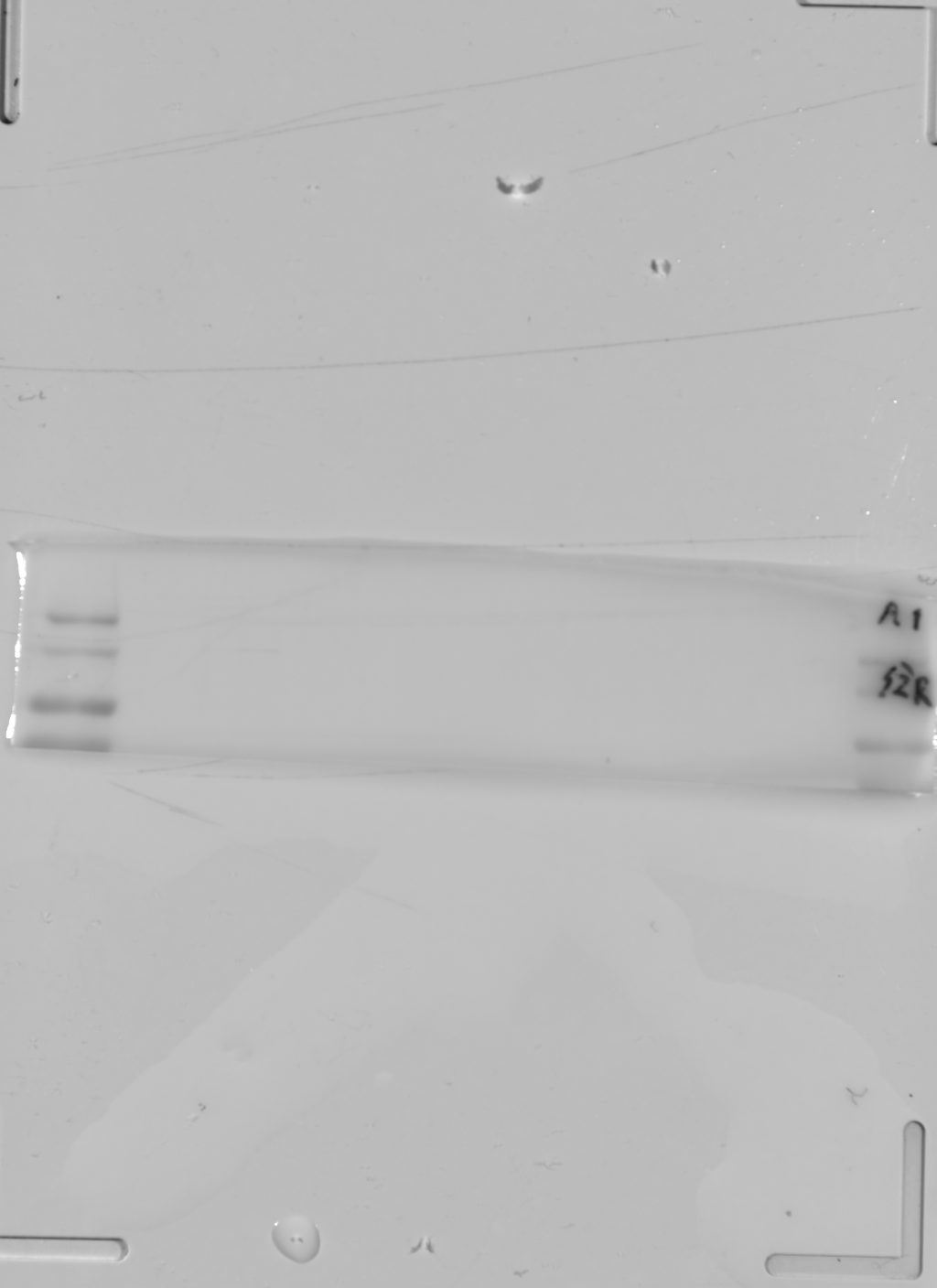

Supplement: Supplementary file 2 — Additional file 2. Raw data of western blot. [file 12974_2022_2632_MOESM2_ESM.zip › supplementary files/Figure5 WB/AR SIRT1/2020.08.05_16.52.44_Ch-Marker.tif]

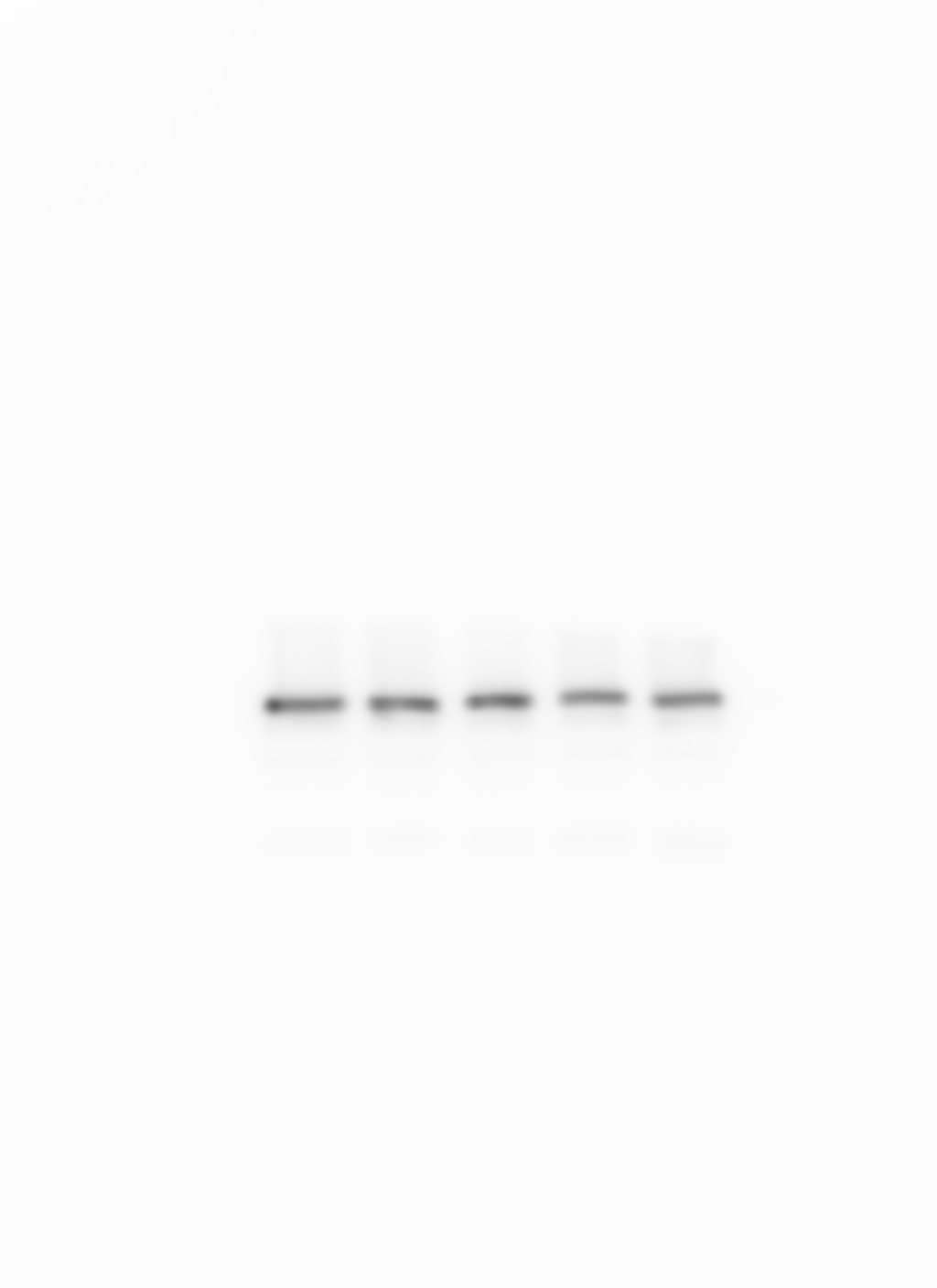

Supplement: Supplementary file 2 — Additional file 2. Raw data of western blot. [file 12974_2022_2632_MOESM2_ESM.zip › supplementary files/Figure5 WB/AR-mtor/4mtor 2021.04.24_17.01.43_Ch.tif]

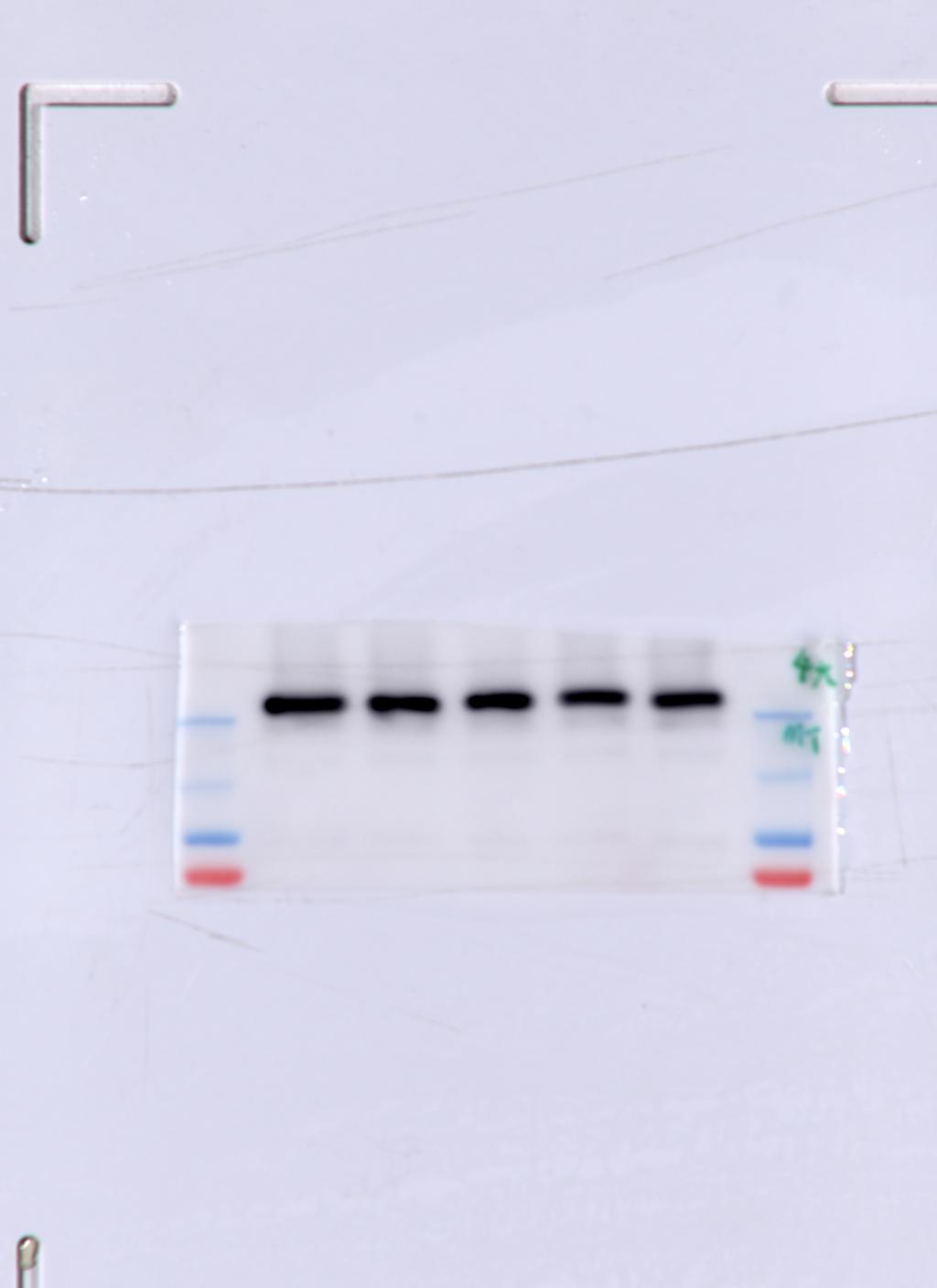

Supplement: Supplementary file 2 — Additional file 2. Raw data of western blot. [file 12974_2022_2632_MOESM2_ESM.zip › supplementary files/Figure5 WB/AR-mtor/4mtor 2021.04.24_17.01.43_Ch+Marker.jpg]

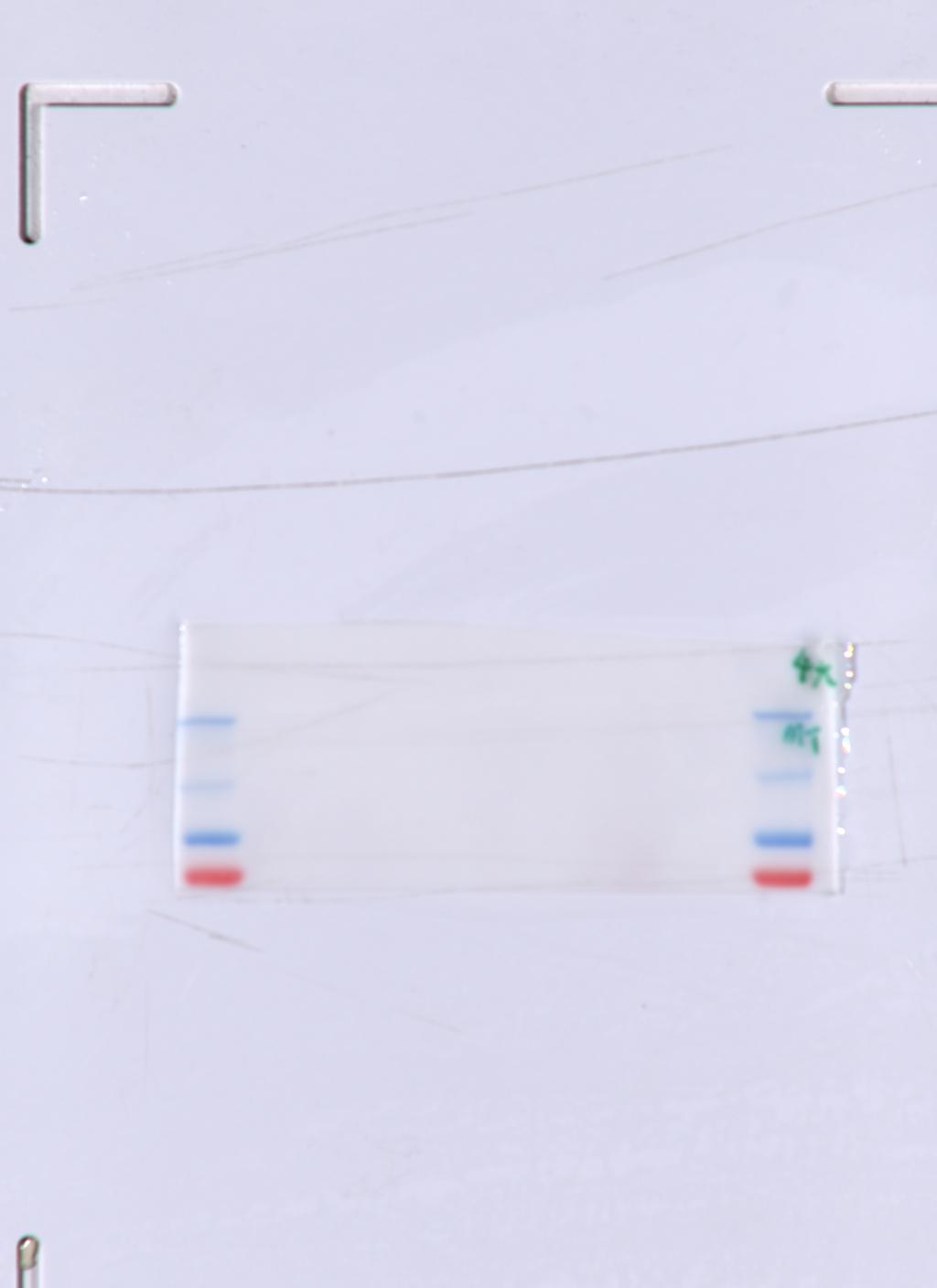

Supplement: Supplementary file 2 — Additional file 2. Raw data of western blot. [file 12974_2022_2632_MOESM2_ESM.zip › supplementary files/Figure5 WB/AR-mtor/4mtor 2021.04.24_17.01.43_Ch-Marker.jpg]

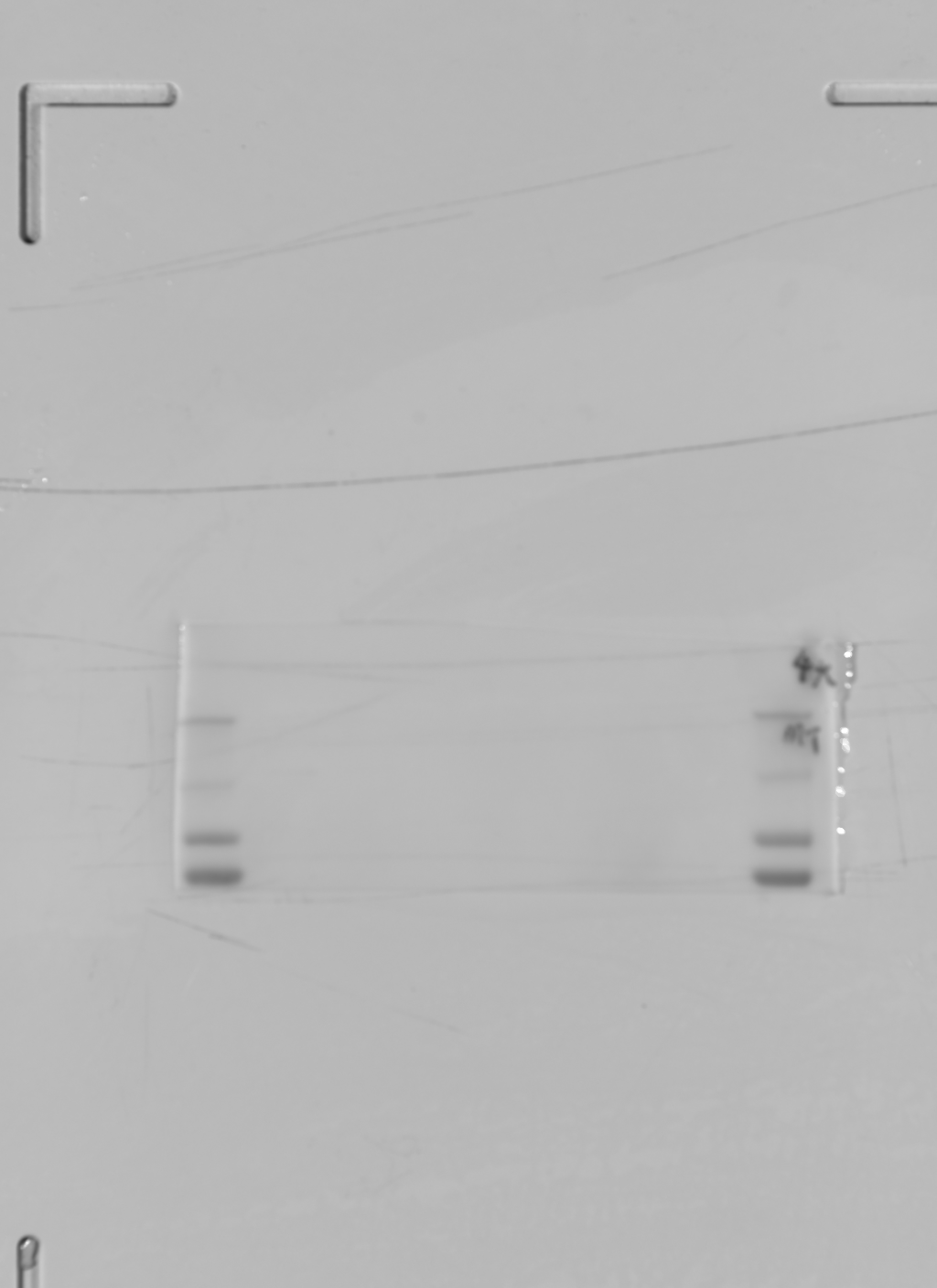

Supplement: Supplementary file 2 — Additional file 2. Raw data of western blot. [file 12974_2022_2632_MOESM2_ESM.zip › supplementary files/Figure5 WB/AR-mtor/4mtor 2021.04.24_17.01.43_Ch-Marker.tif]

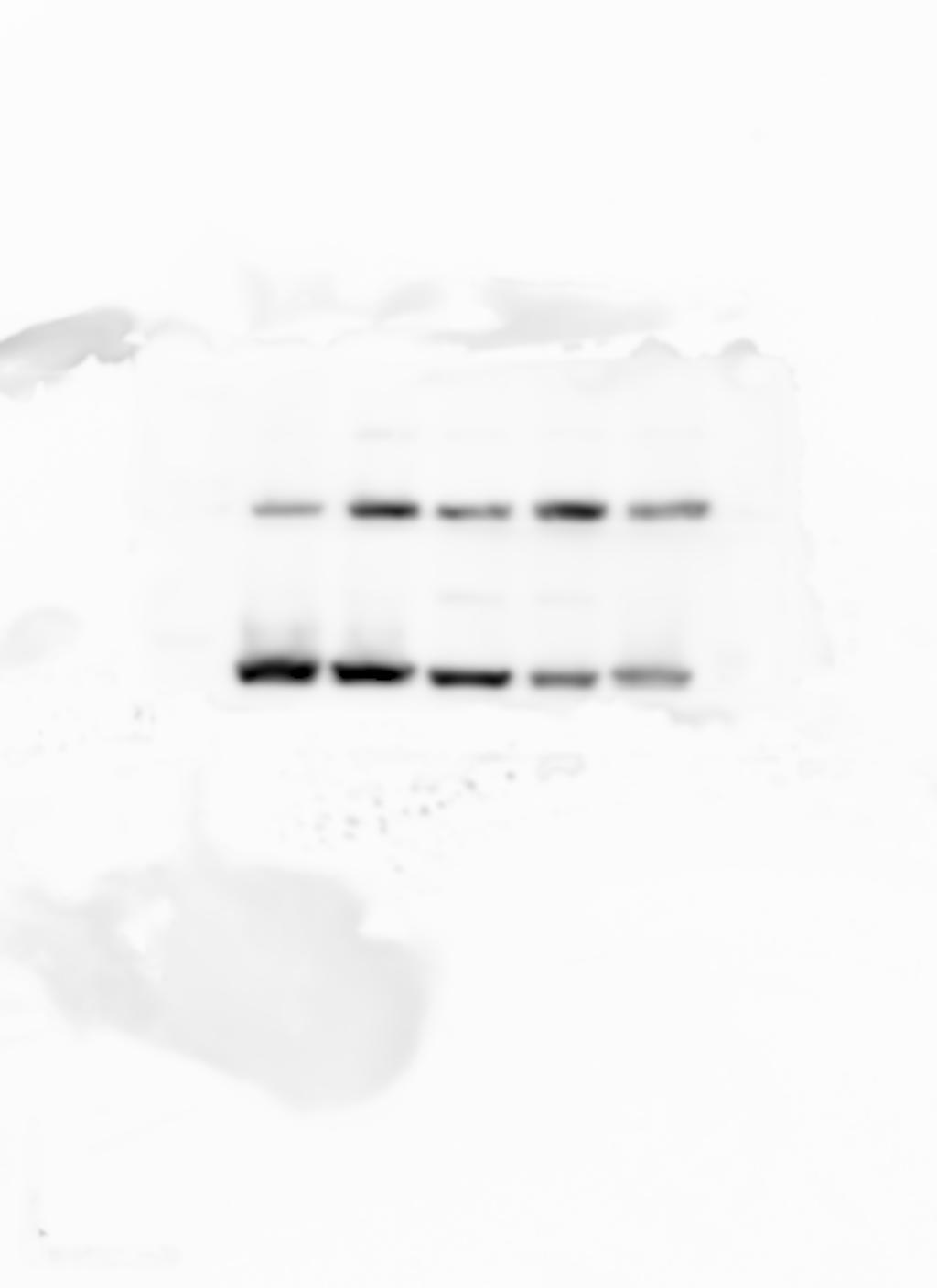

Supplement: Supplementary file 2 — Additional file 2. Raw data of western blot. [file 12974_2022_2632_MOESM2_ESM.zip › supplementary files/Figure5 WB/AR-pampk/1wtpampk 2ar pampk 2021.01.06_14.51.59-02_Ch.jpg]

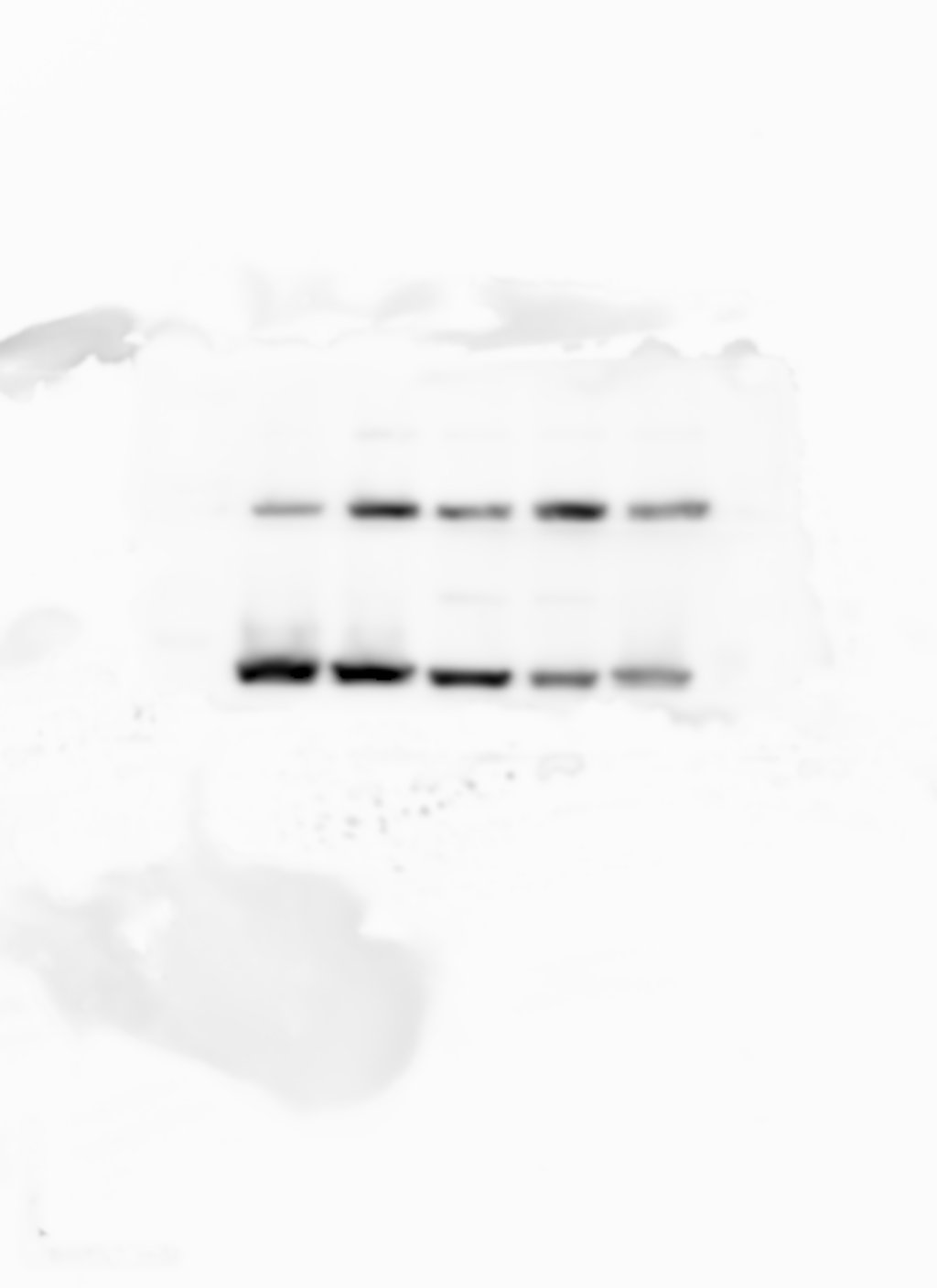

Supplement: Supplementary file 2 — Additional file 2. Raw data of western blot. [file 12974_2022_2632_MOESM2_ESM.zip › supplementary files/Figure5 WB/AR-pampk/1wtpampk 2ar pampk 2021.01.06_14.51.59-02_Ch.tif]

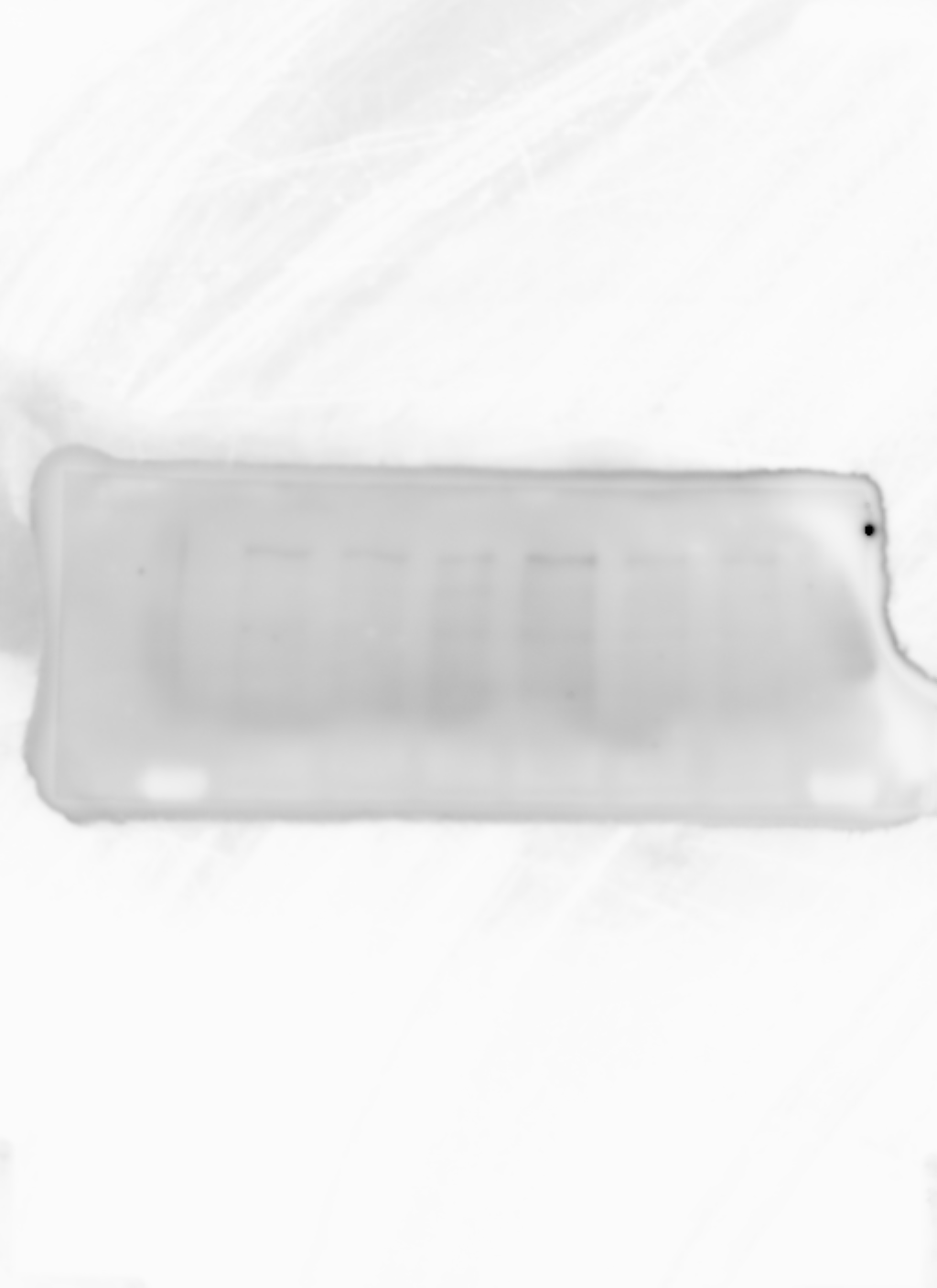

Supplement: Supplementary file 2 — Additional file 2. Raw data of western blot. [file 12974_2022_2632_MOESM2_ESM.zip › supplementary files/Figure5 WB/AR-pmtor/pmtor-2 2020.09.16_17.40.11_Ch.tif]

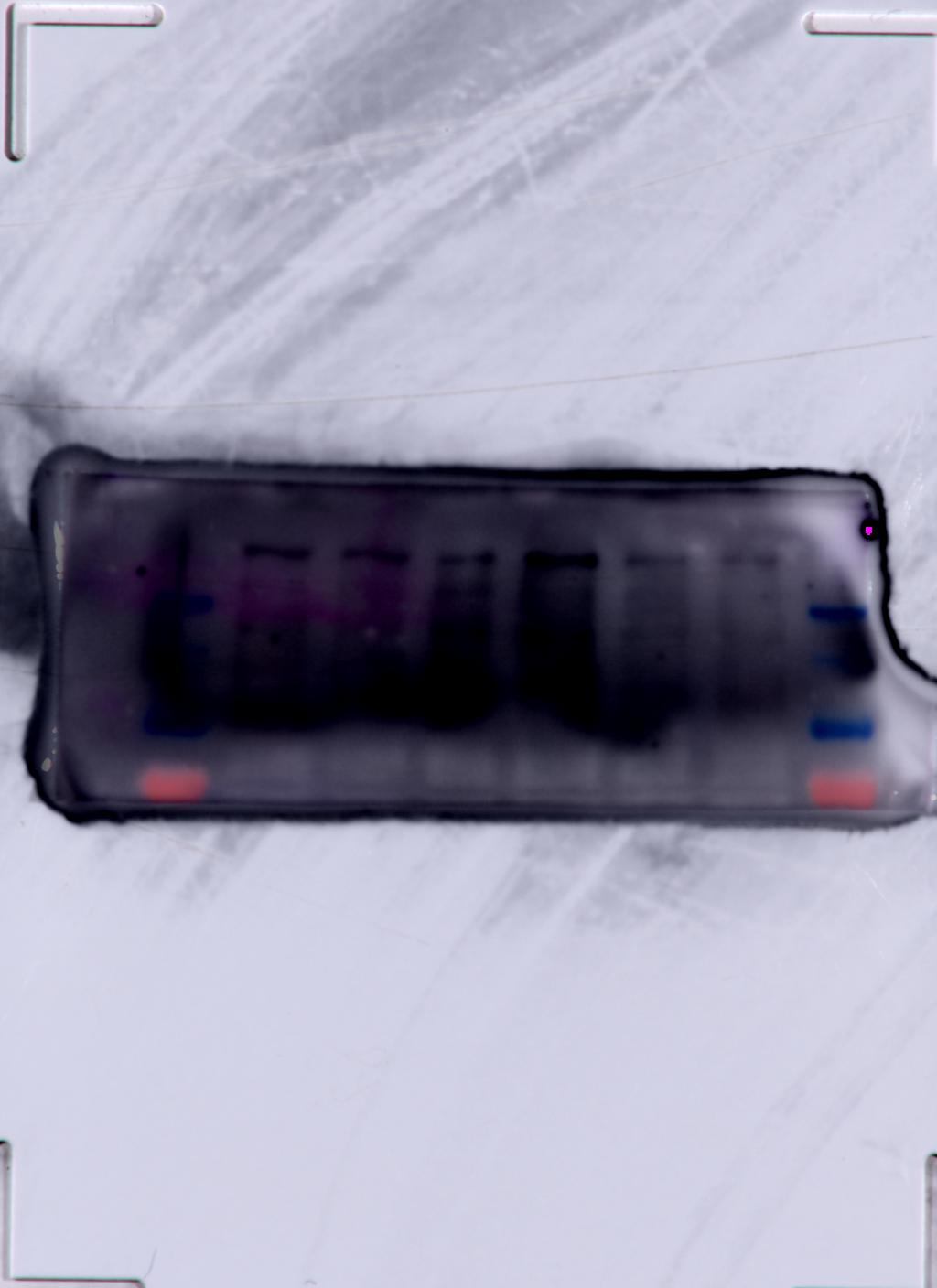

Supplement: Supplementary file 2 — Additional file 2. Raw data of western blot. [file 12974_2022_2632_MOESM2_ESM.zip › supplementary files/Figure5 WB/AR-pmtor/pmtor-2 2020.09.16_17.40.11_Ch+Marker.jpg]

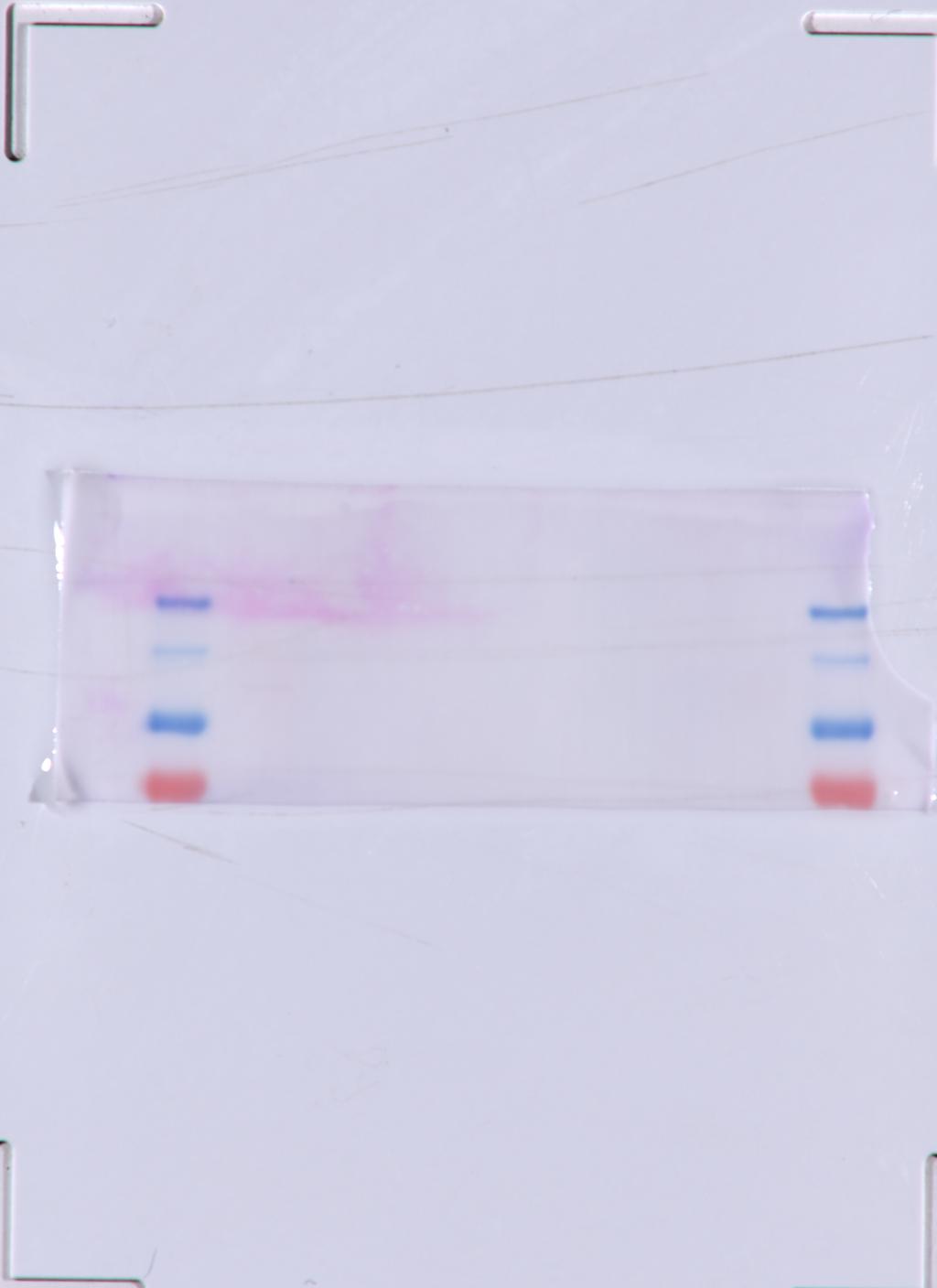

Supplement: Supplementary file 2 — Additional file 2. Raw data of western blot. [file 12974_2022_2632_MOESM2_ESM.zip › supplementary files/Figure5 WB/AR-pmtor/pmtor-2 2020.09.16_17.40.11_Ch-Marker.jpg]

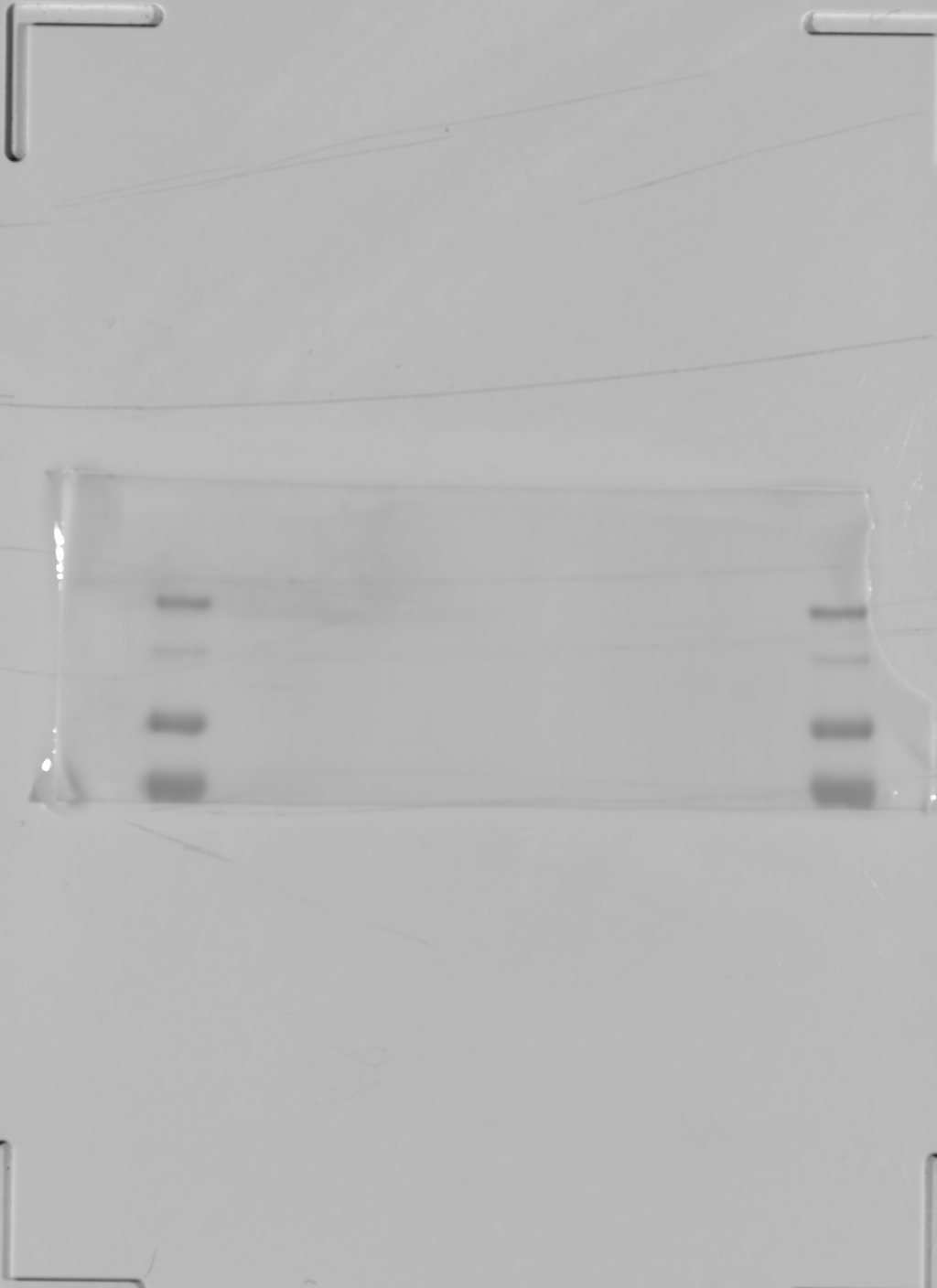

Supplement: Supplementary file 2 — Additional file 2. Raw data of western blot. [file 12974_2022_2632_MOESM2_ESM.zip › supplementary files/Figure5 WB/AR-pmtor/pmtor-2 2020.09.16_17.40.11_Ch-Marker.tif]

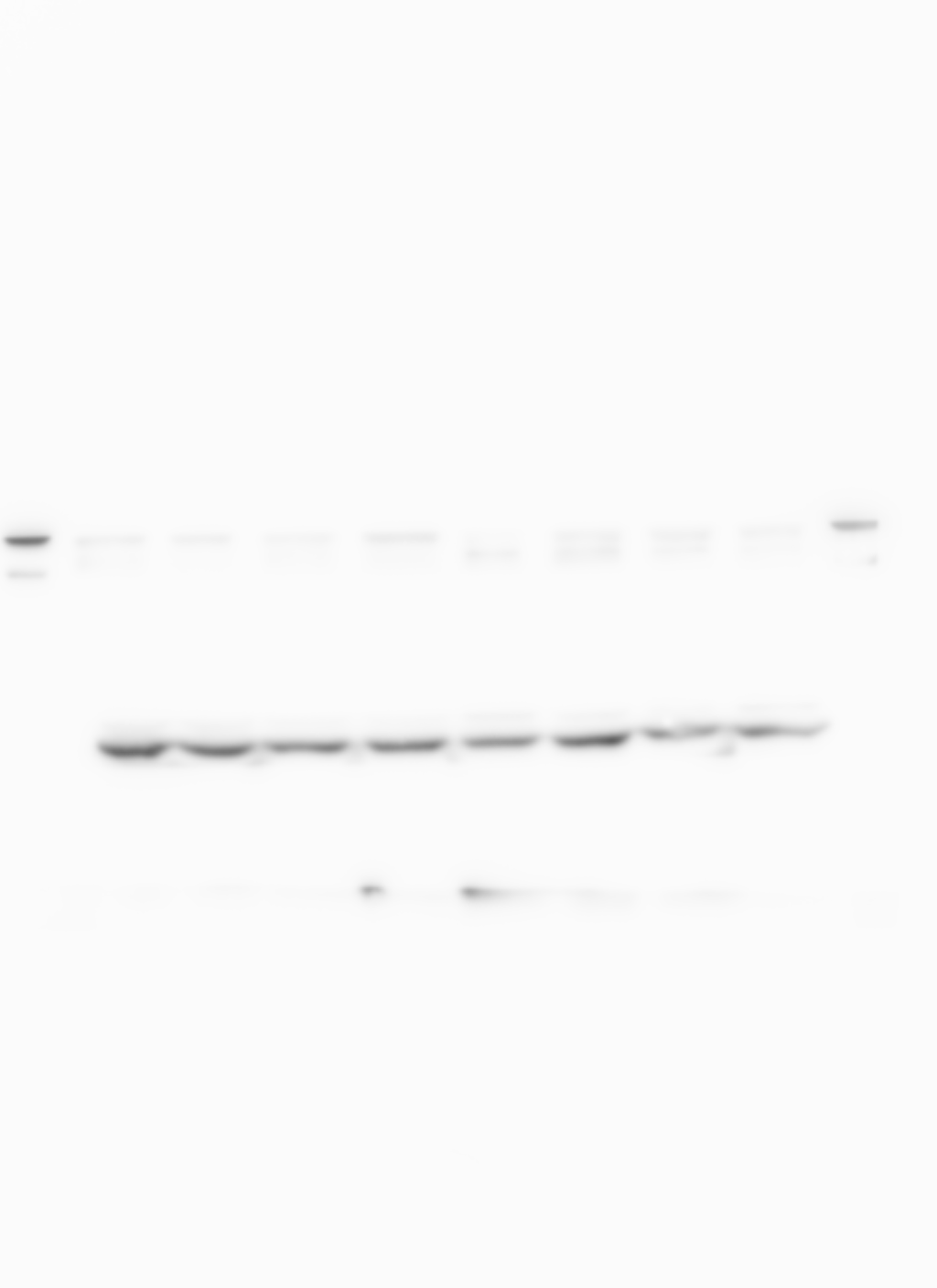

Supplement: Supplementary file 2 — Additional file 2. Raw data of western blot. [file 12974_2022_2632_MOESM2_ESM.zip › supplementary files/Figure5 WB/gapdh new/1 gapdh2020.08.07_19.45.10_Ch.tif]

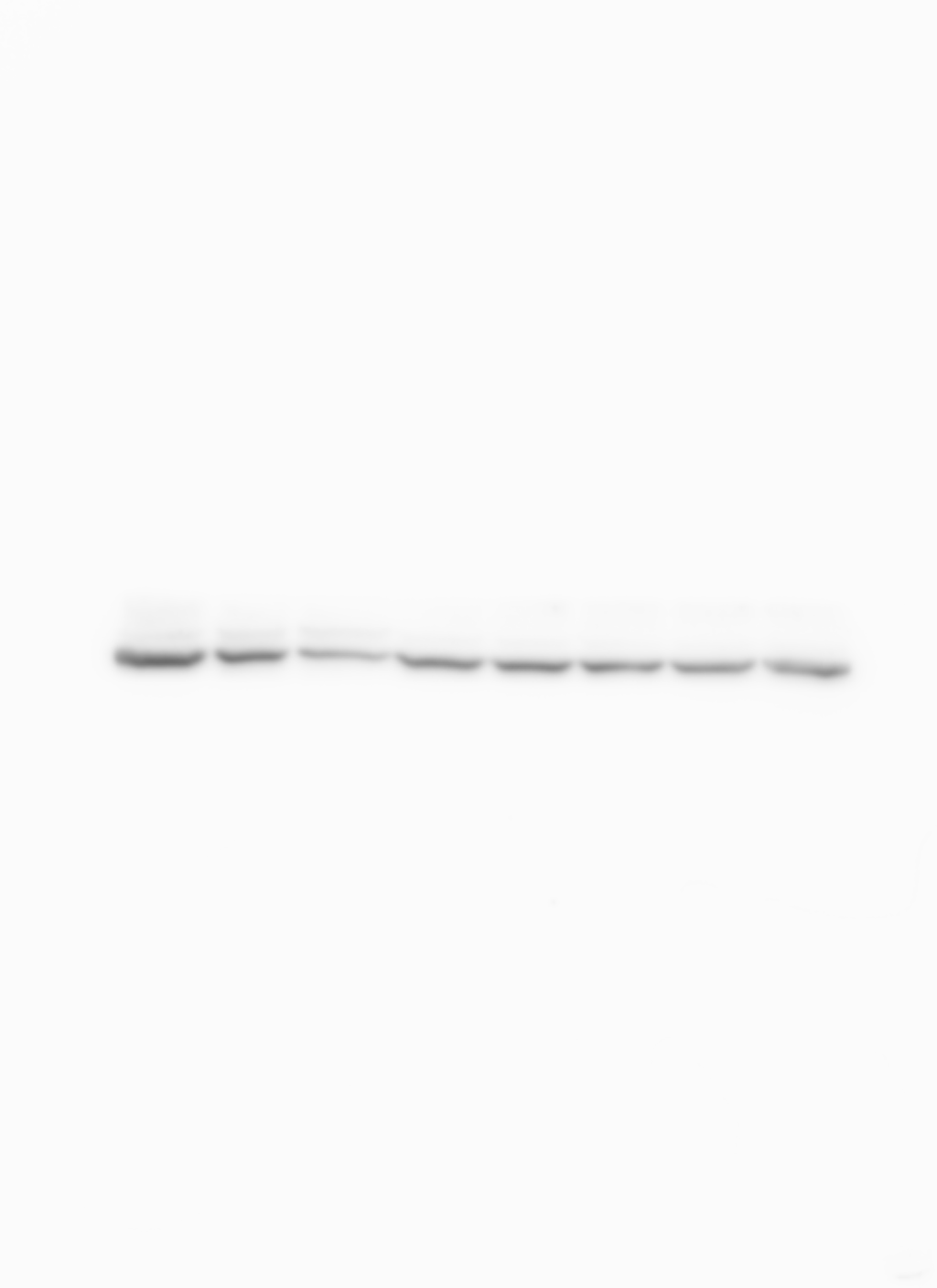

Supplement: Supplementary file 2 — Additional file 2. Raw data of western blot. [file 12974_2022_2632_MOESM2_ESM.zip › supplementary files/Figure5 WB/gapdh new/1ar gapdh 2020.08.05_16.56.40_Ch.tif]

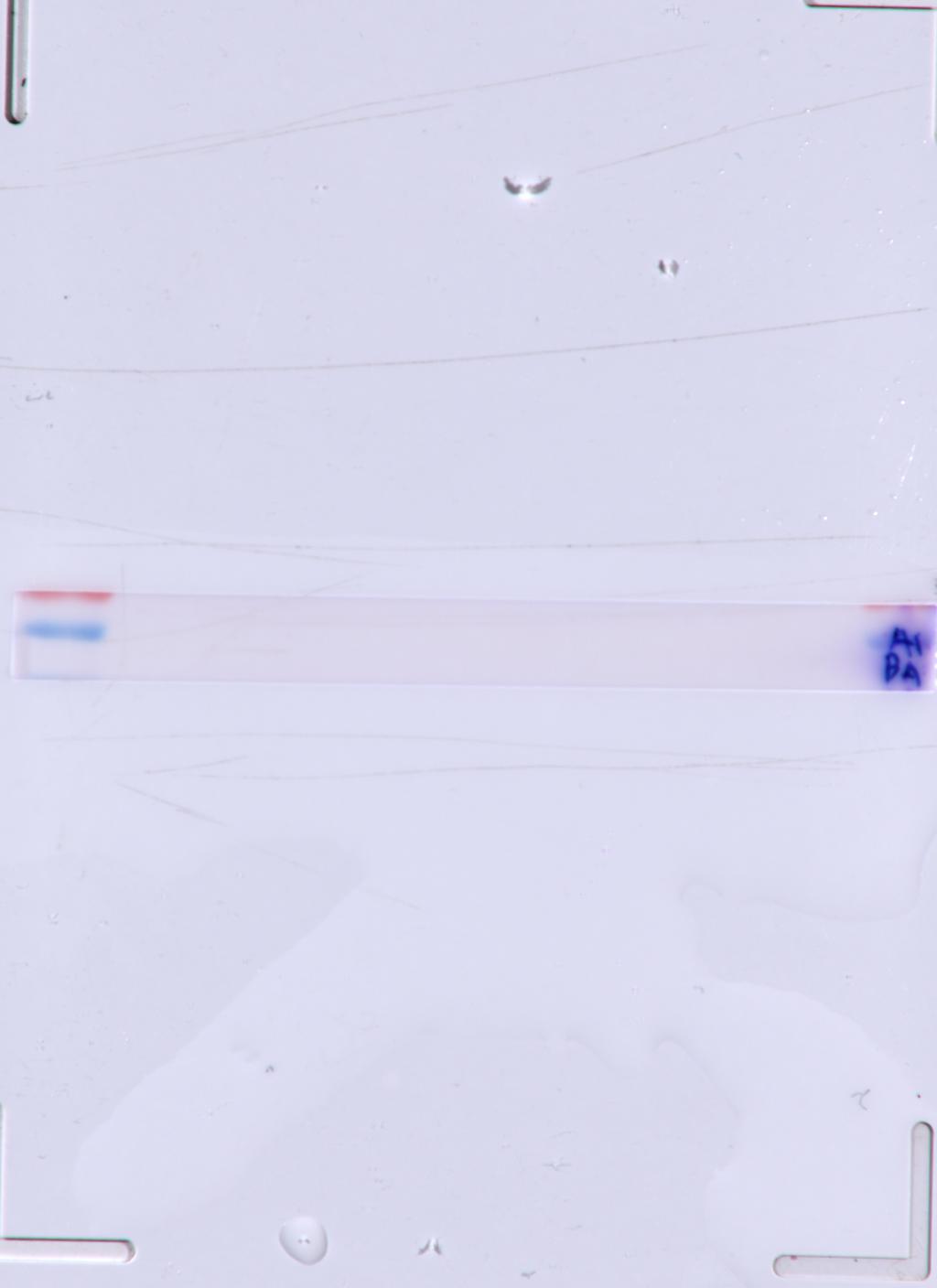

Supplement: Supplementary file 2 — Additional file 2. Raw data of western blot. [file 12974_2022_2632_MOESM2_ESM.zip › supplementary files/Figure5 WB/gapdh new/1ar gapdh 2020.08.05_16.56.40_Ch-Marker.jpg]

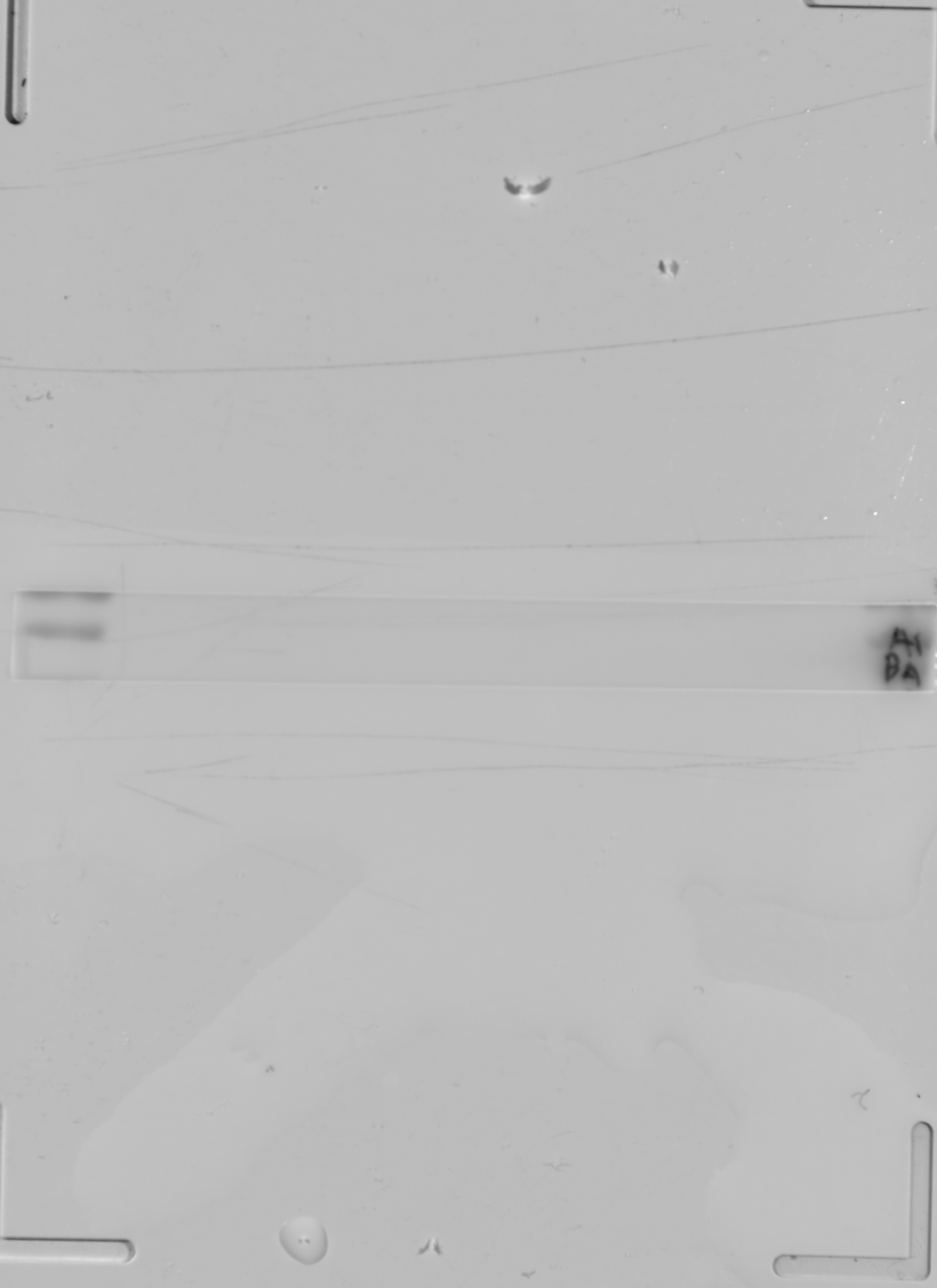

Supplement: Supplementary file 2 — Additional file 2. Raw data of western blot. [file 12974_2022_2632_MOESM2_ESM.zip › supplementary files/Figure5 WB/gapdh new/1ar gapdh 2020.08.05_16.56.40_Ch-Marker.tif]

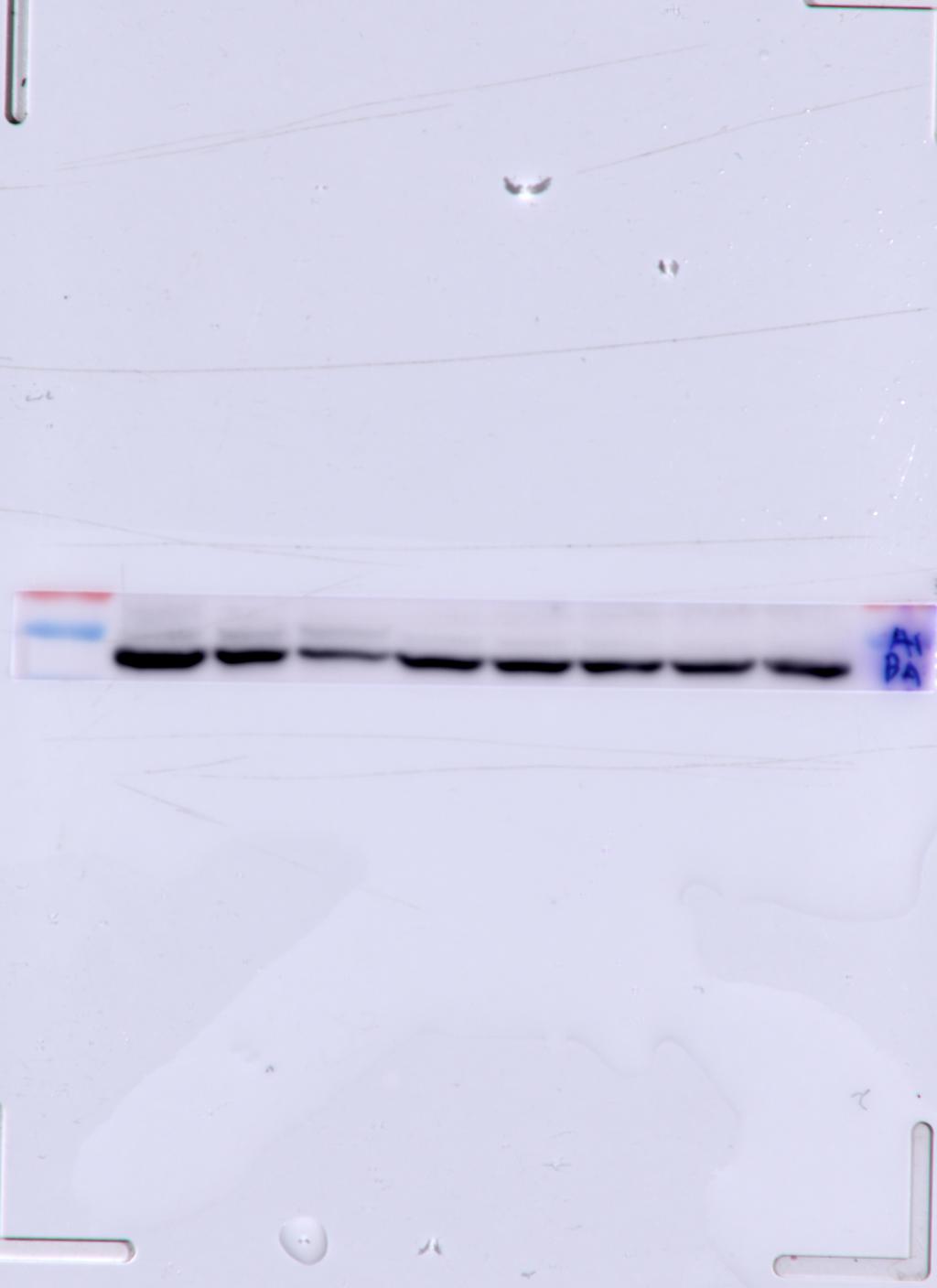

Supplement: Supplementary file 2 — Additional file 2. Raw data of western blot. [file 12974_2022_2632_MOESM2_ESM.zip › supplementary files/Figure5 WB/gapdh new/1ar gapdh2020.08.05_16.56.40_Ch+Marker.jpg]

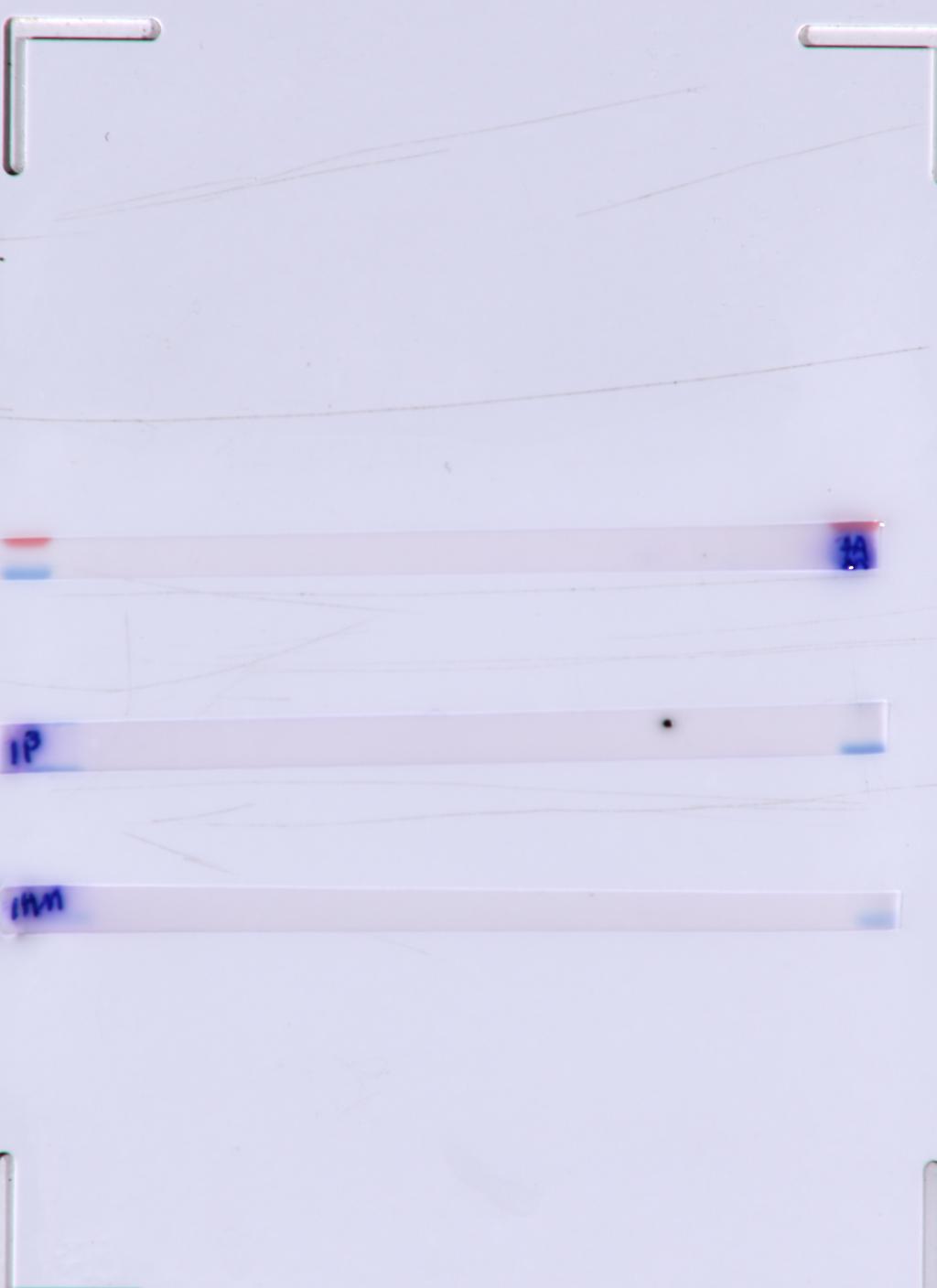

Supplement: Supplementary file 2 — Additional file 2. Raw data of western blot. [file 12974_2022_2632_MOESM2_ESM.zip › supplementary files/Figure5 WB/gapdh new/1gapdh 2020.08.07_19.45.10_Ch-Marker.jpg]

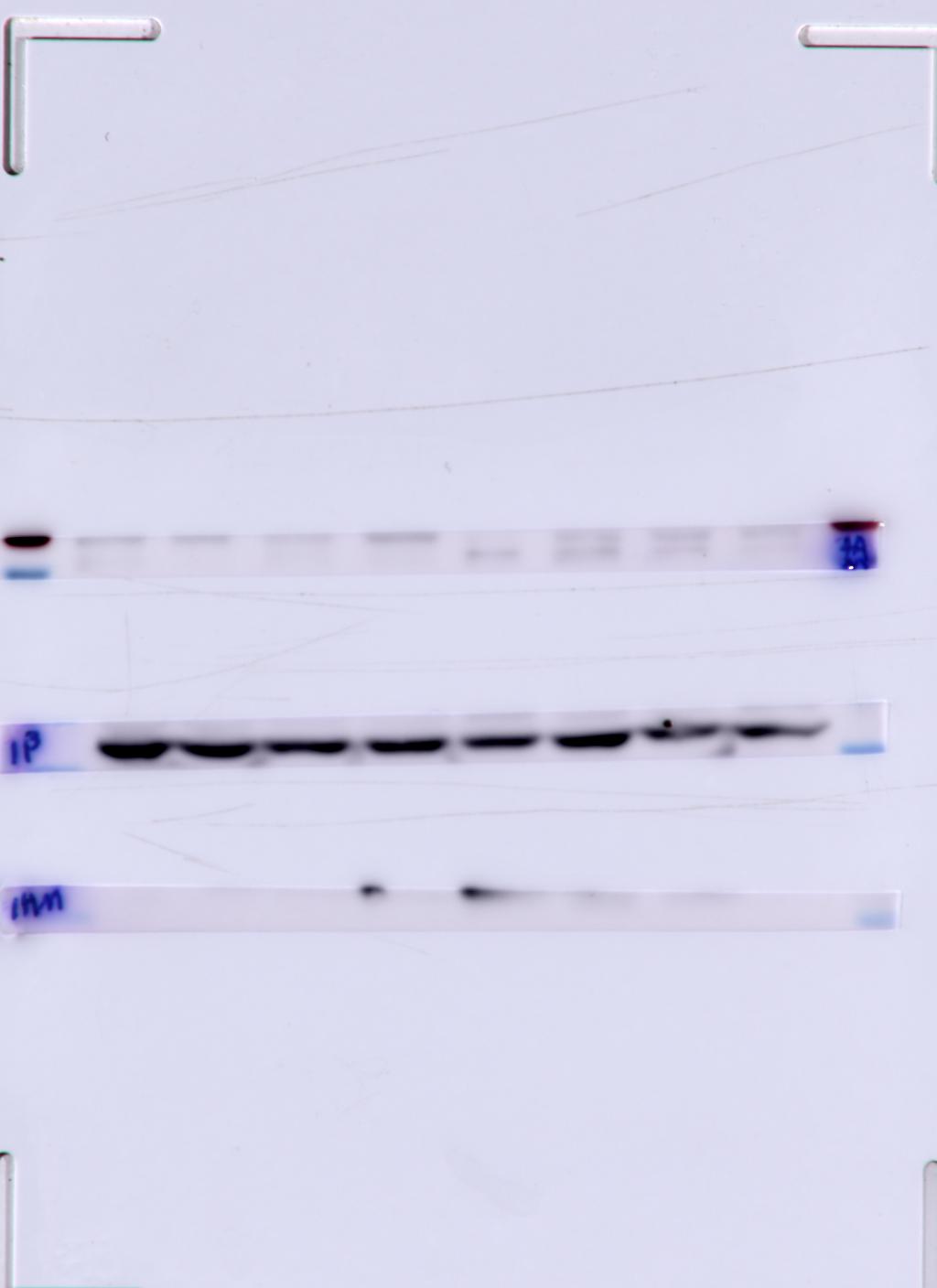

Supplement: Supplementary file 2 — Additional file 2. Raw data of western blot. [file 12974_2022_2632_MOESM2_ESM.zip › supplementary files/Figure5 WB/gapdh new/1gapdh2020.08.07_19.45.10_Ch+Marker.jpg]

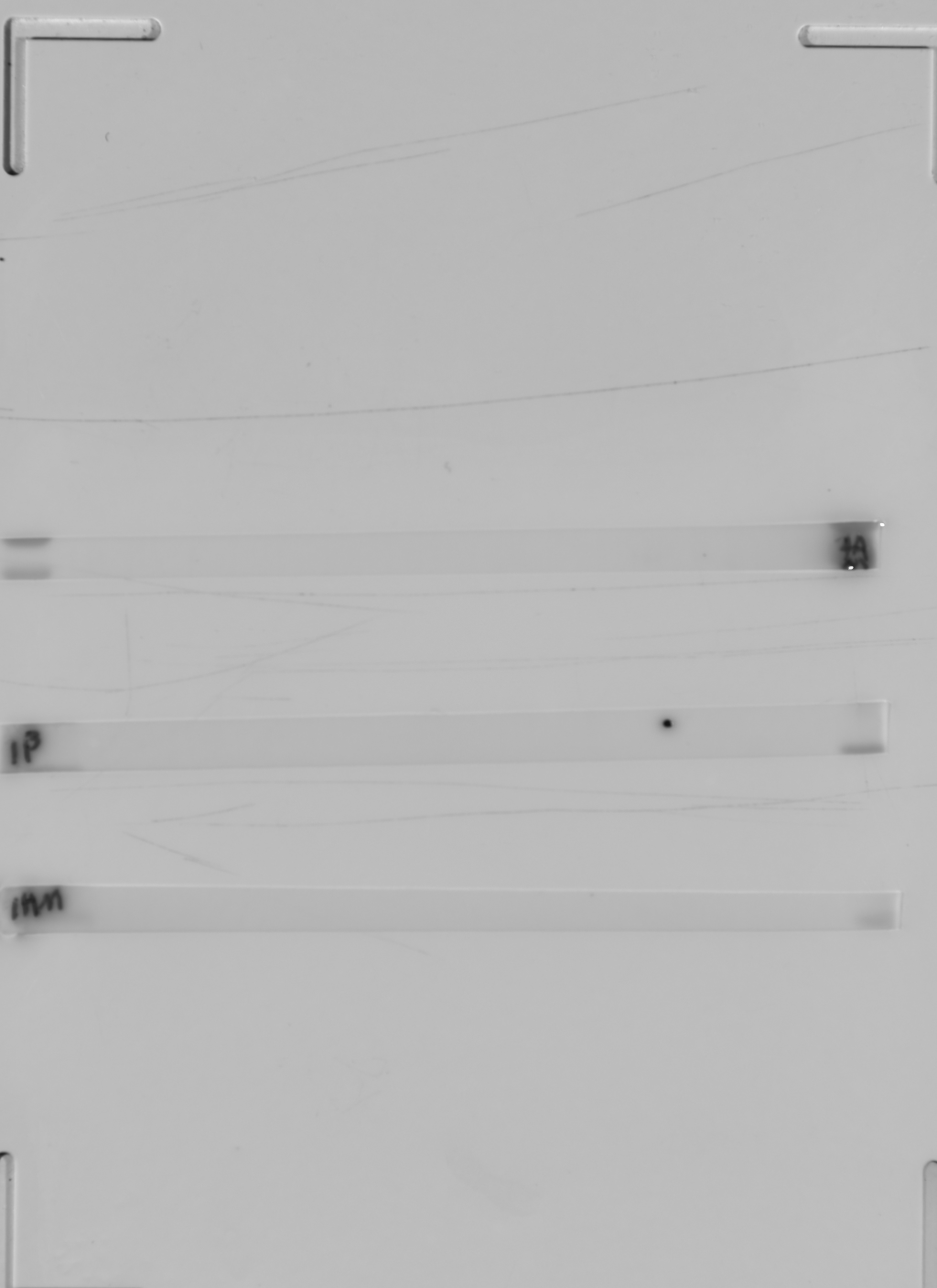

Supplement: Supplementary file 2 — Additional file 2. Raw data of western blot. [file 12974_2022_2632_MOESM2_ESM.zip › supplementary files/Figure5 WB/gapdh new/1gapdh2020.08.07_19.45.10_Ch-Marker.tif]

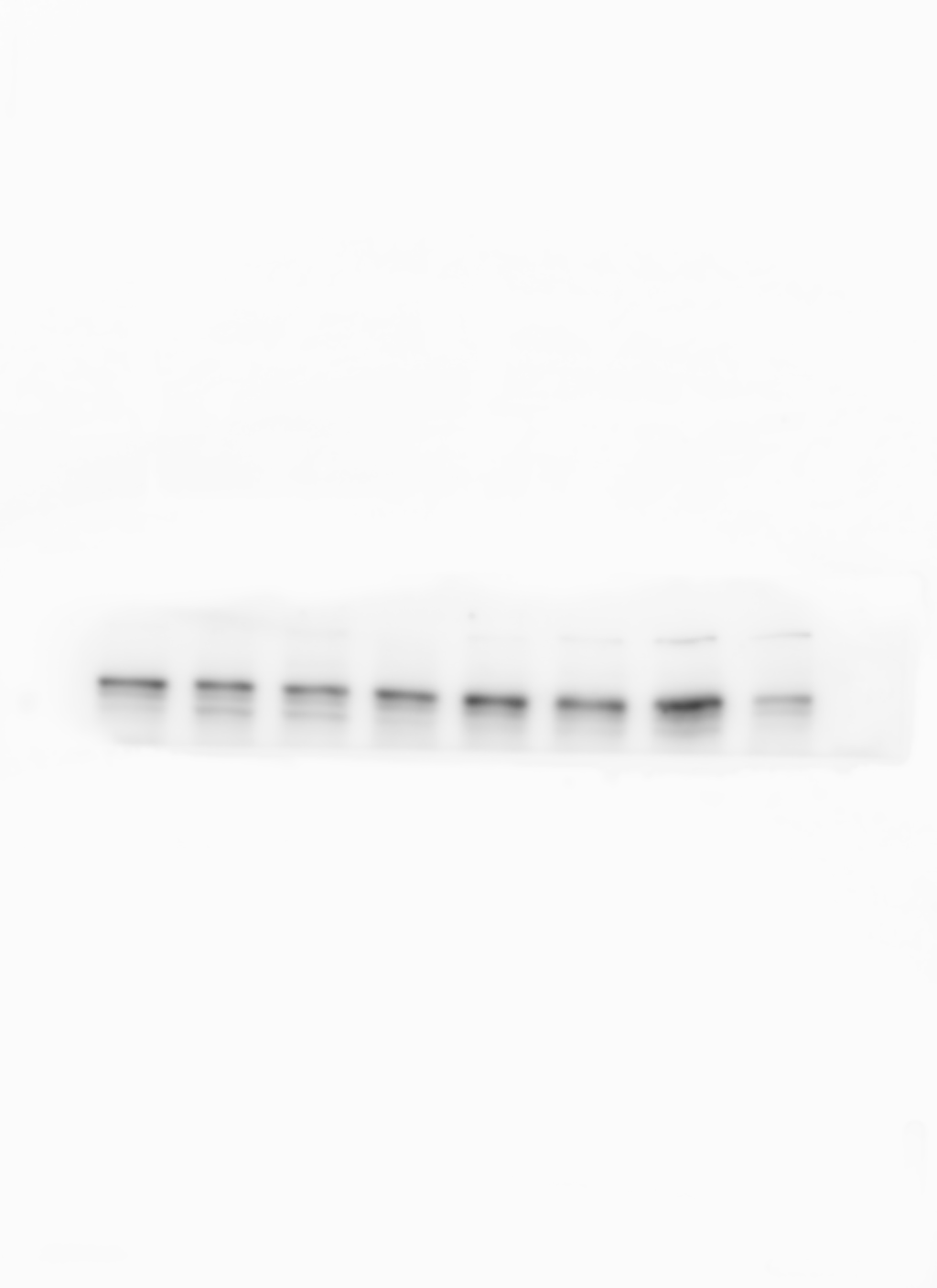

Supplement: Supplementary file 2 — Additional file 2. Raw data of western blot. [file 12974_2022_2632_MOESM2_ESM.zip › supplementary files/Figure5 WB/sirt1 new/4sirt1 2020.01.16_15.15.50_Ch.tif]

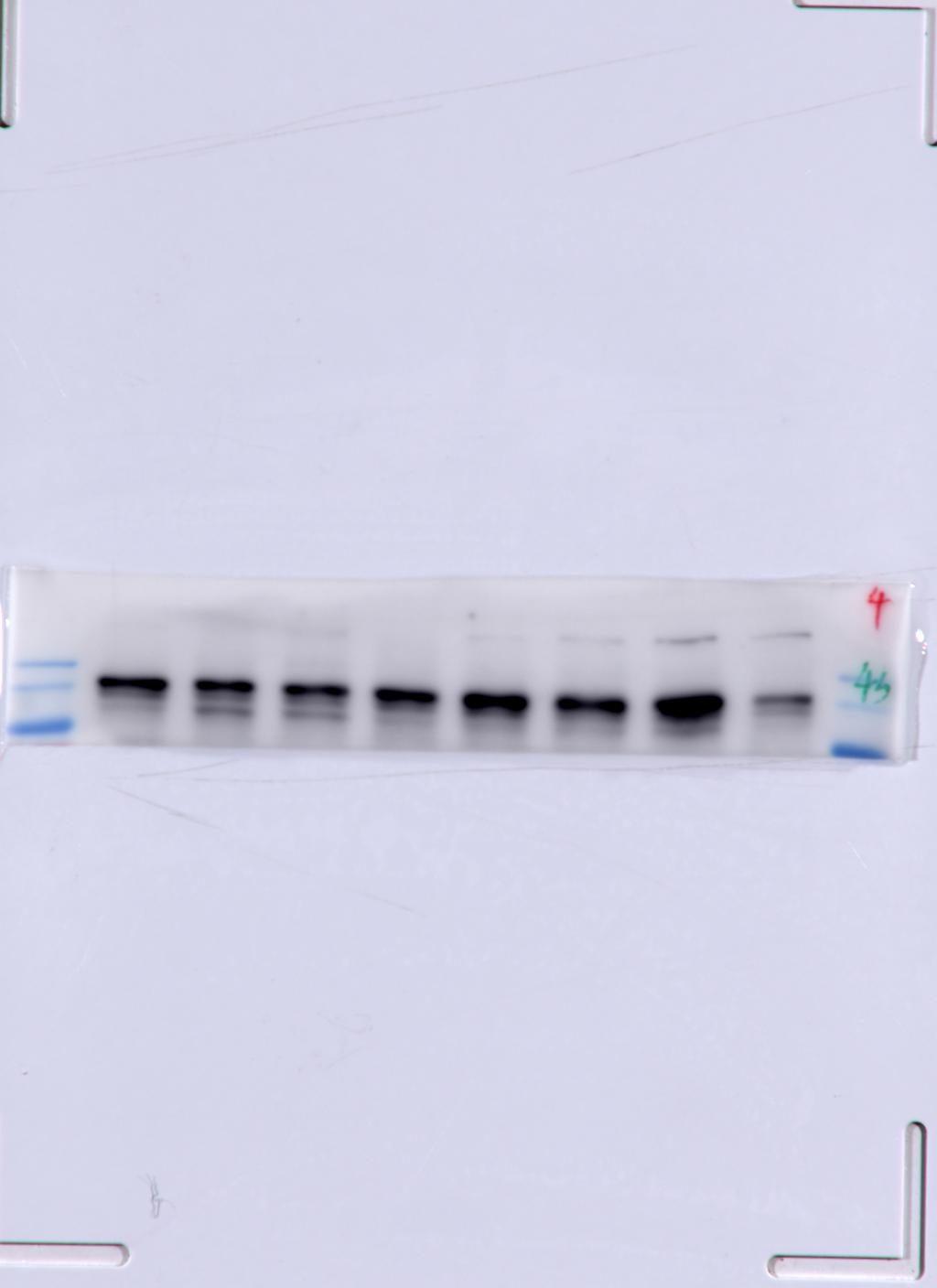

Supplement: Supplementary file 2 — Additional file 2. Raw data of western blot. [file 12974_2022_2632_MOESM2_ESM.zip › supplementary files/Figure5 WB/sirt1 new/4sirt1 2020.01.16_15.15.50_Ch+Marker.jpg]

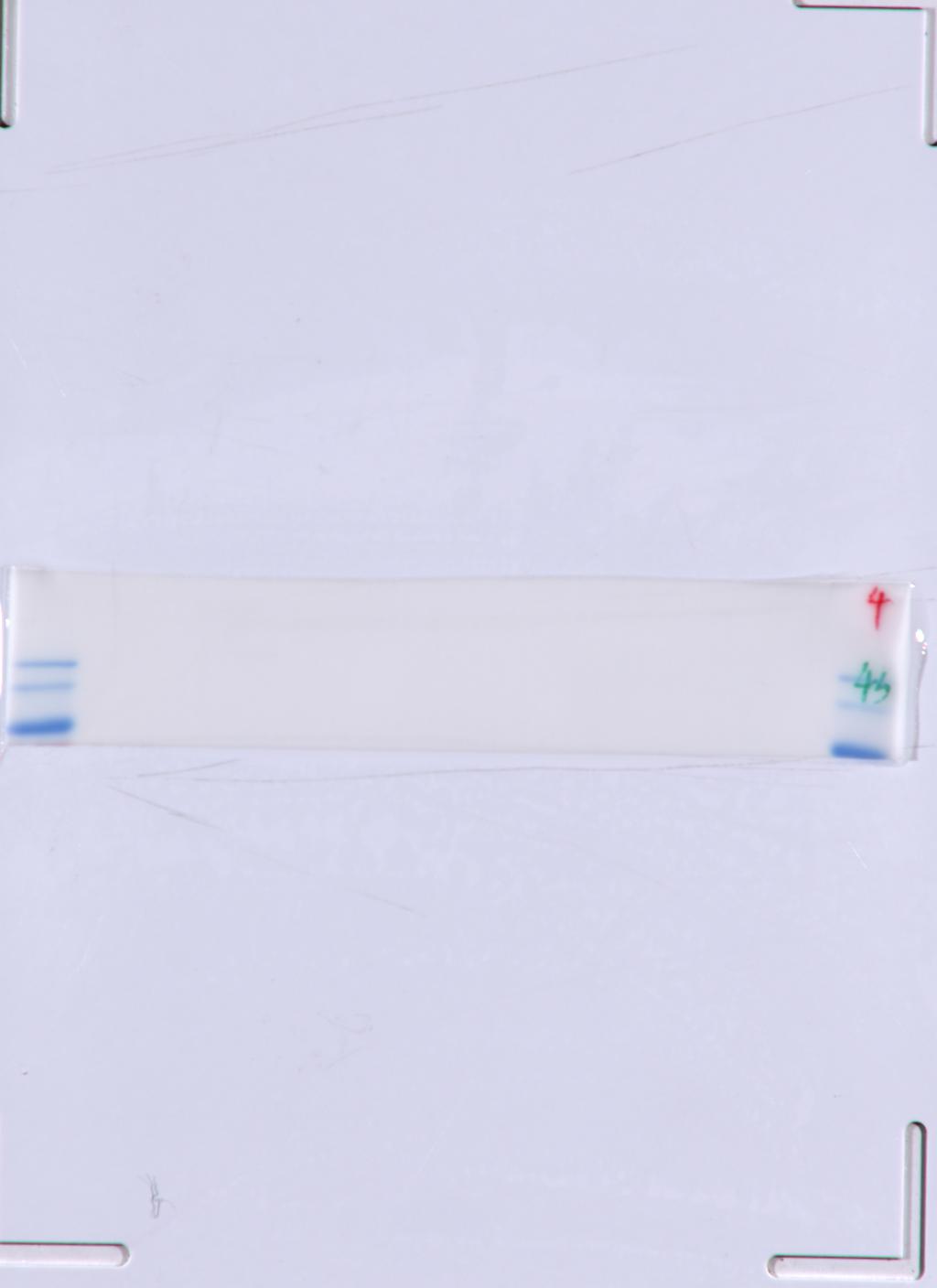

Supplement: Supplementary file 2 — Additional file 2. Raw data of western blot. [file 12974_2022_2632_MOESM2_ESM.zip › supplementary files/Figure5 WB/sirt1 new/4sirt1 2020.01.16_15.15.50_Ch-Marker.jpg]

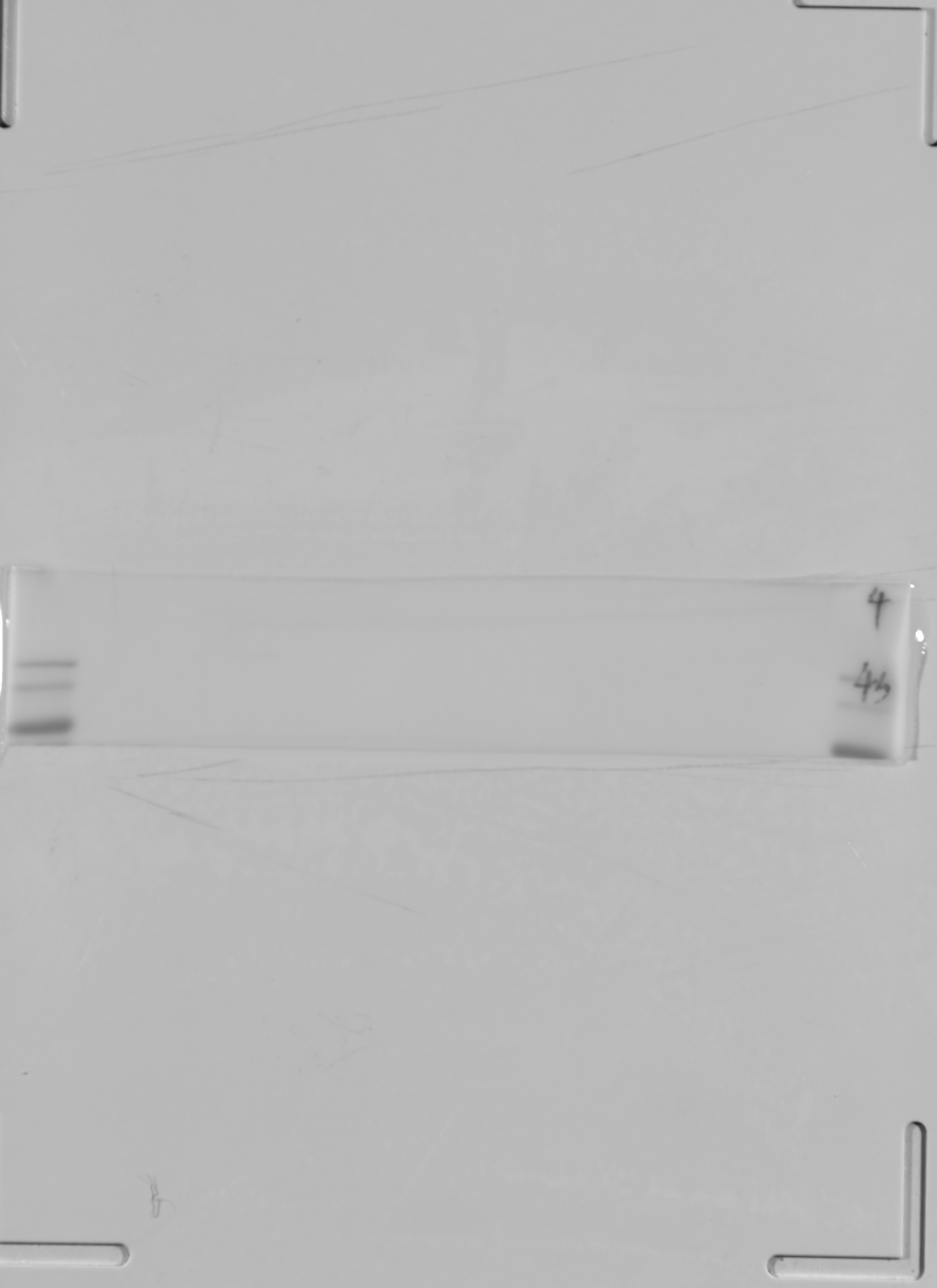

Supplement: Supplementary file 2 — Additional file 2. Raw data of western blot. [file 12974_2022_2632_MOESM2_ESM.zip › supplementary files/Figure5 WB/sirt1 new/4sirt1 2020.01.16_15.15.50_Ch-Marker.tif]

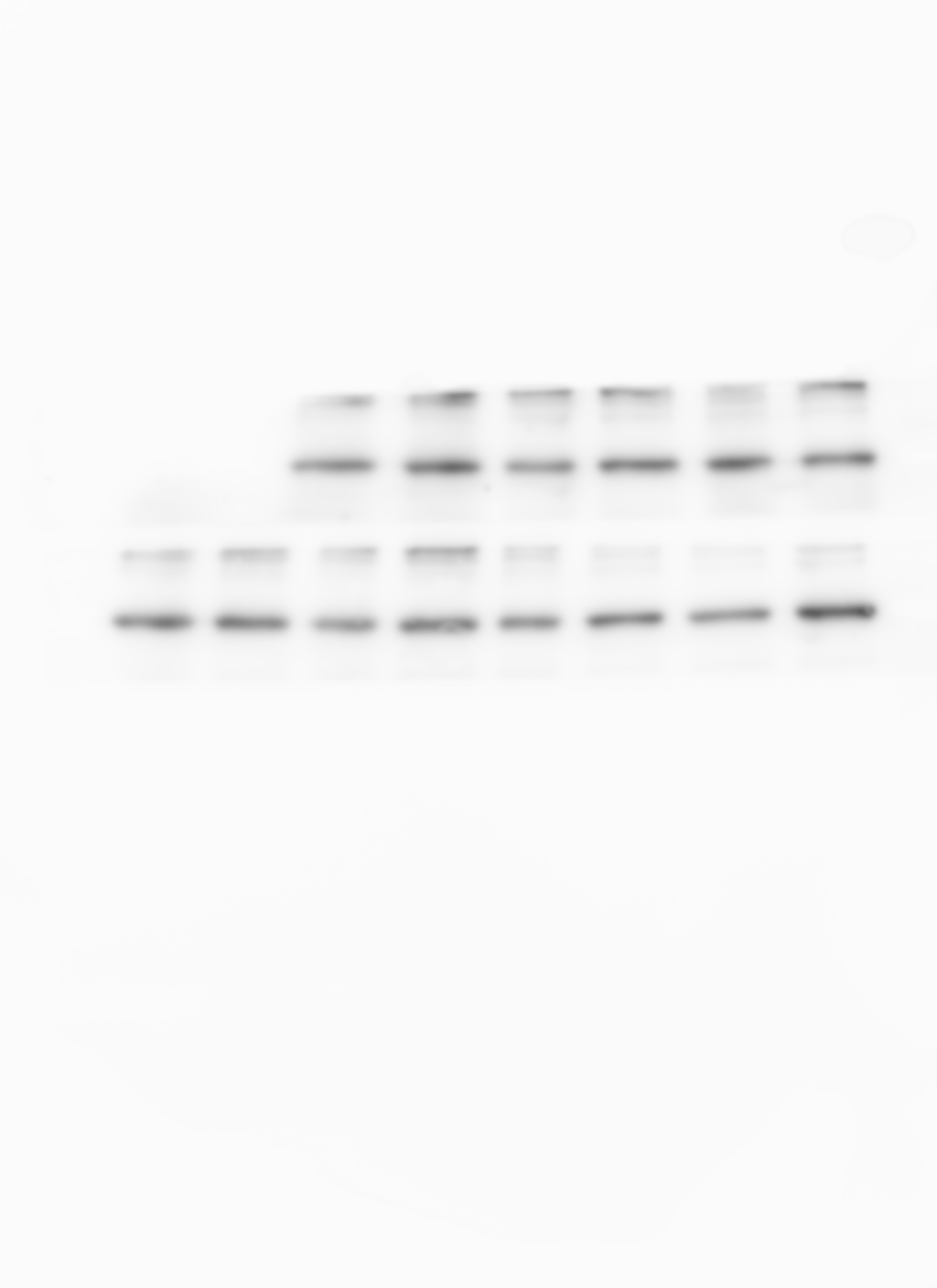

Supplement: Supplementary file 2 — Additional file 2. Raw data of western blot. [file 12974_2022_2632_MOESM2_ESM.zip › supplementary files/Figure5 WB/sirt1 new/sirt1 2020.08.20_17.15.52_Ch.tif]

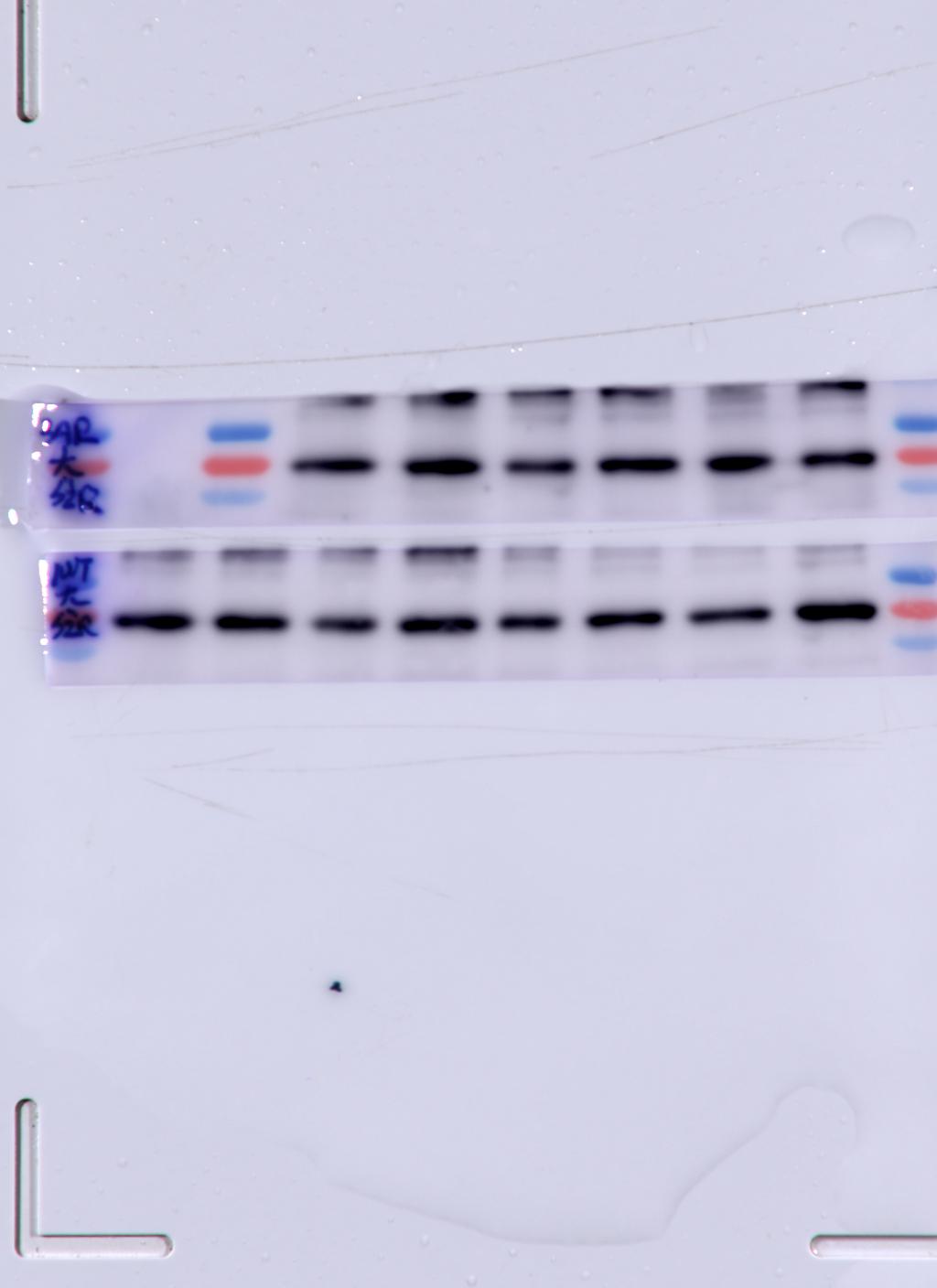

Supplement: Supplementary file 2 — Additional file 2. Raw data of western blot. [file 12974_2022_2632_MOESM2_ESM.zip › supplementary files/Figure5 WB/sirt1 new/sirt1 2020.08.20_17.15.52_Ch+Marker.jpg]

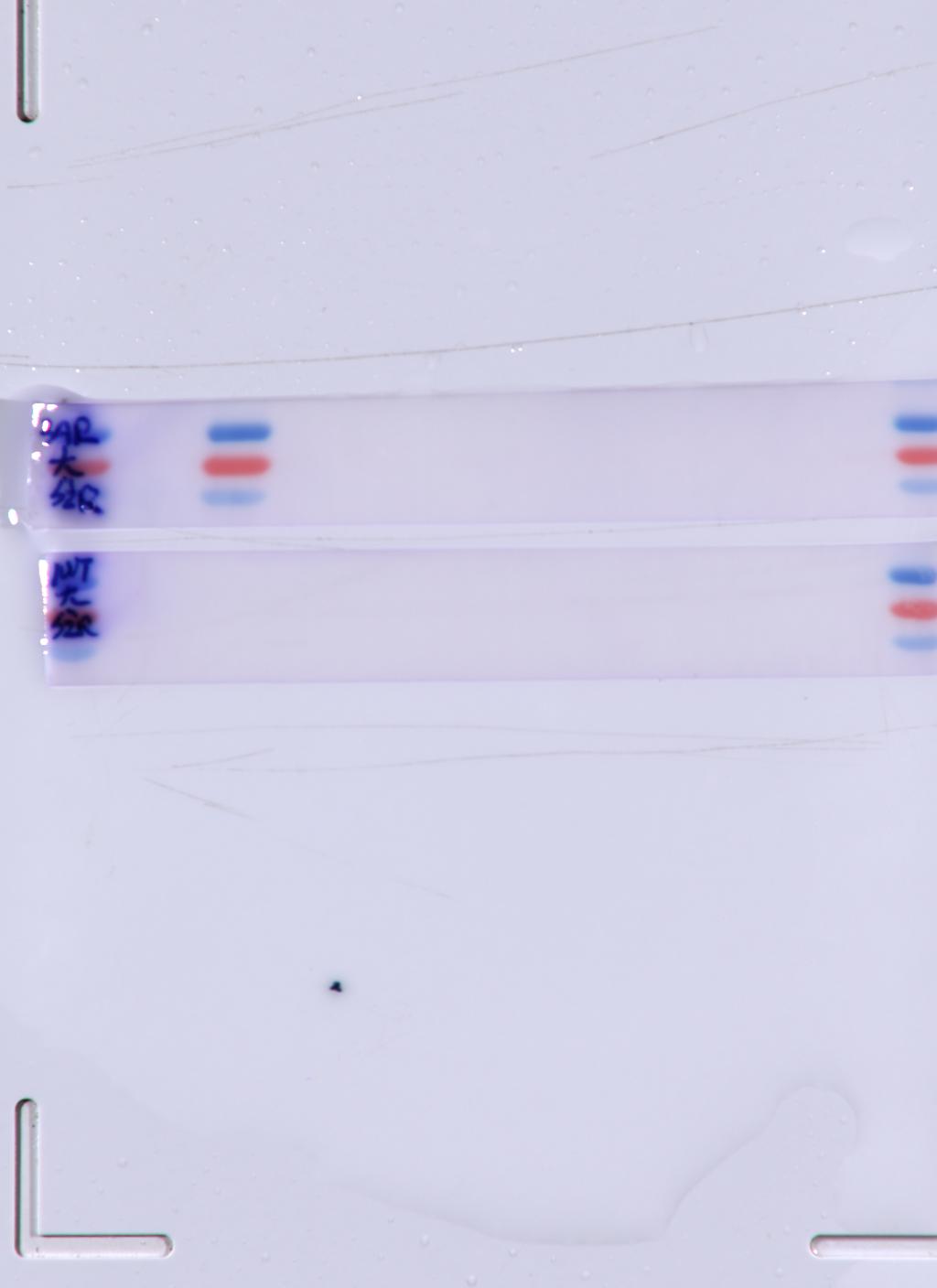

Supplement: Supplementary file 2 — Additional file 2. Raw data of western blot. [file 12974_2022_2632_MOESM2_ESM.zip › supplementary files/Figure5 WB/sirt1 new/sirt1 2020.08.20_17.15.52_Ch-Marker.jpg]

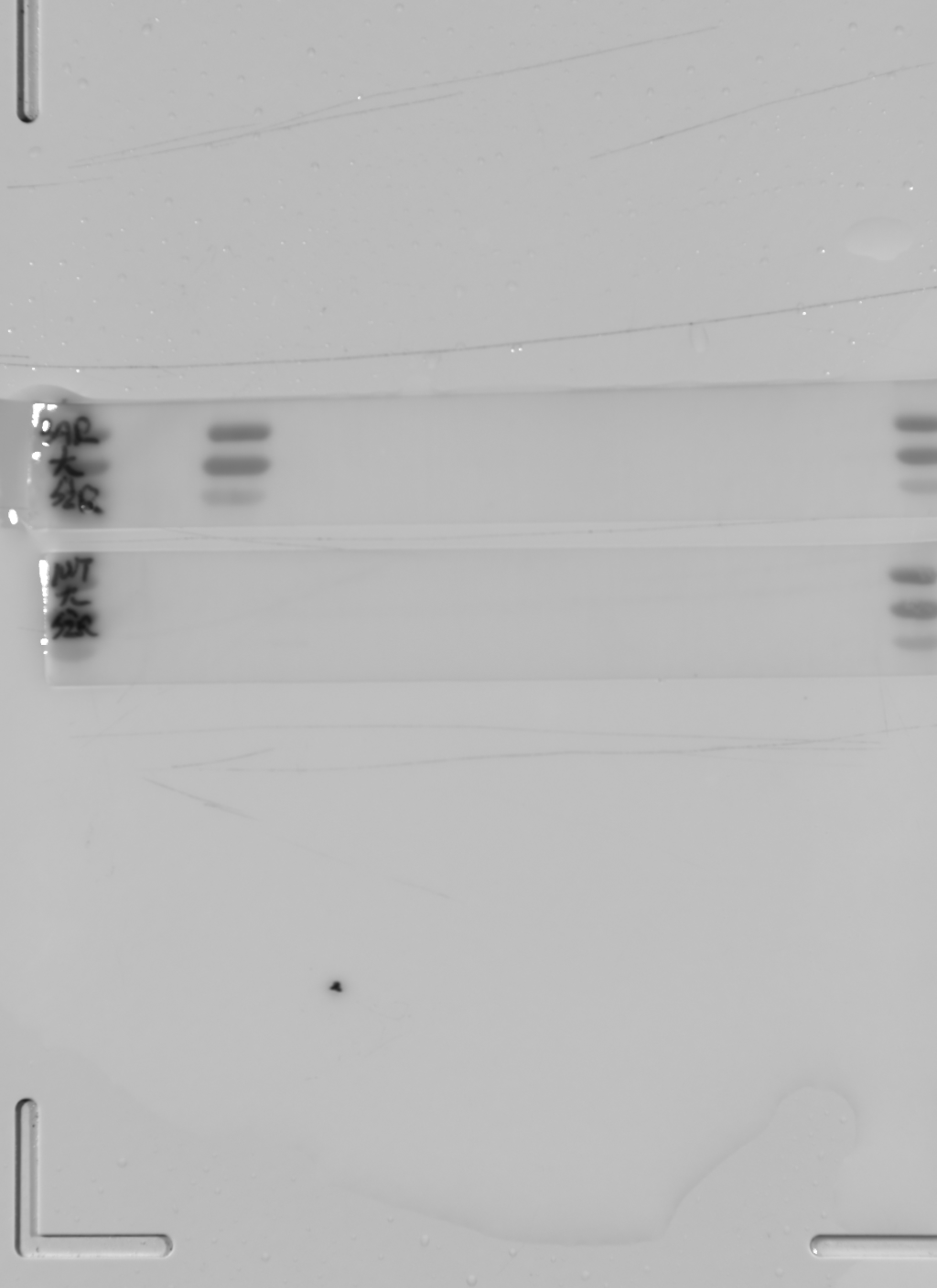

Supplement: Supplementary file 2 — Additional file 2. Raw data of western blot. [file 12974_2022_2632_MOESM2_ESM.zip › supplementary files/Figure5 WB/sirt1 new/sirt1 2020.08.20_17.15.52_Ch-Marker.tif]

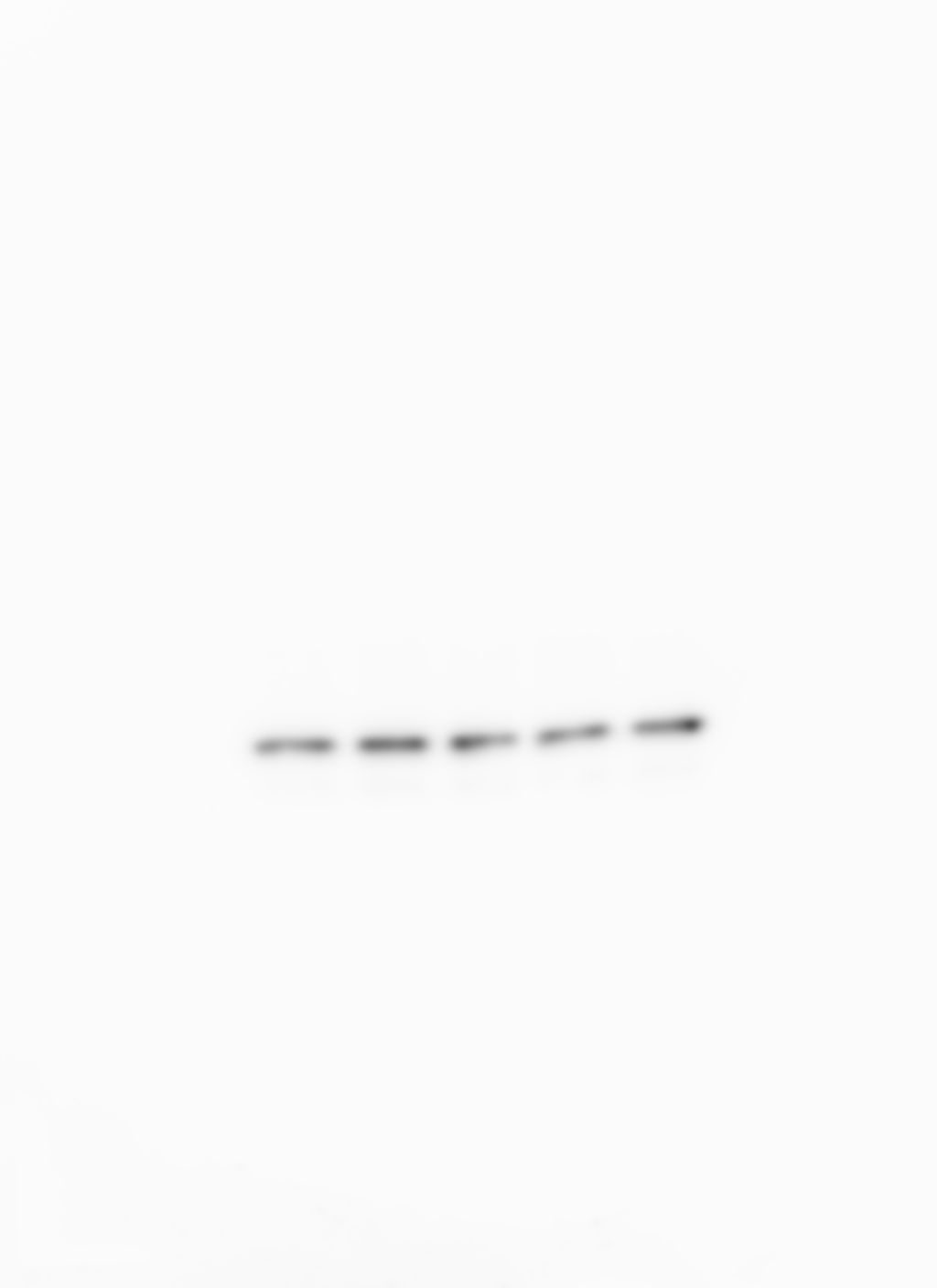

Supplement: Supplementary file 2 — Additional file 2. Raw data of western blot. [file 12974_2022_2632_MOESM2_ESM.zip › supplementary files/Figure5 WB/WT GAPDH/3gapdh 2021.04.24_16.57.16_Ch.tif]

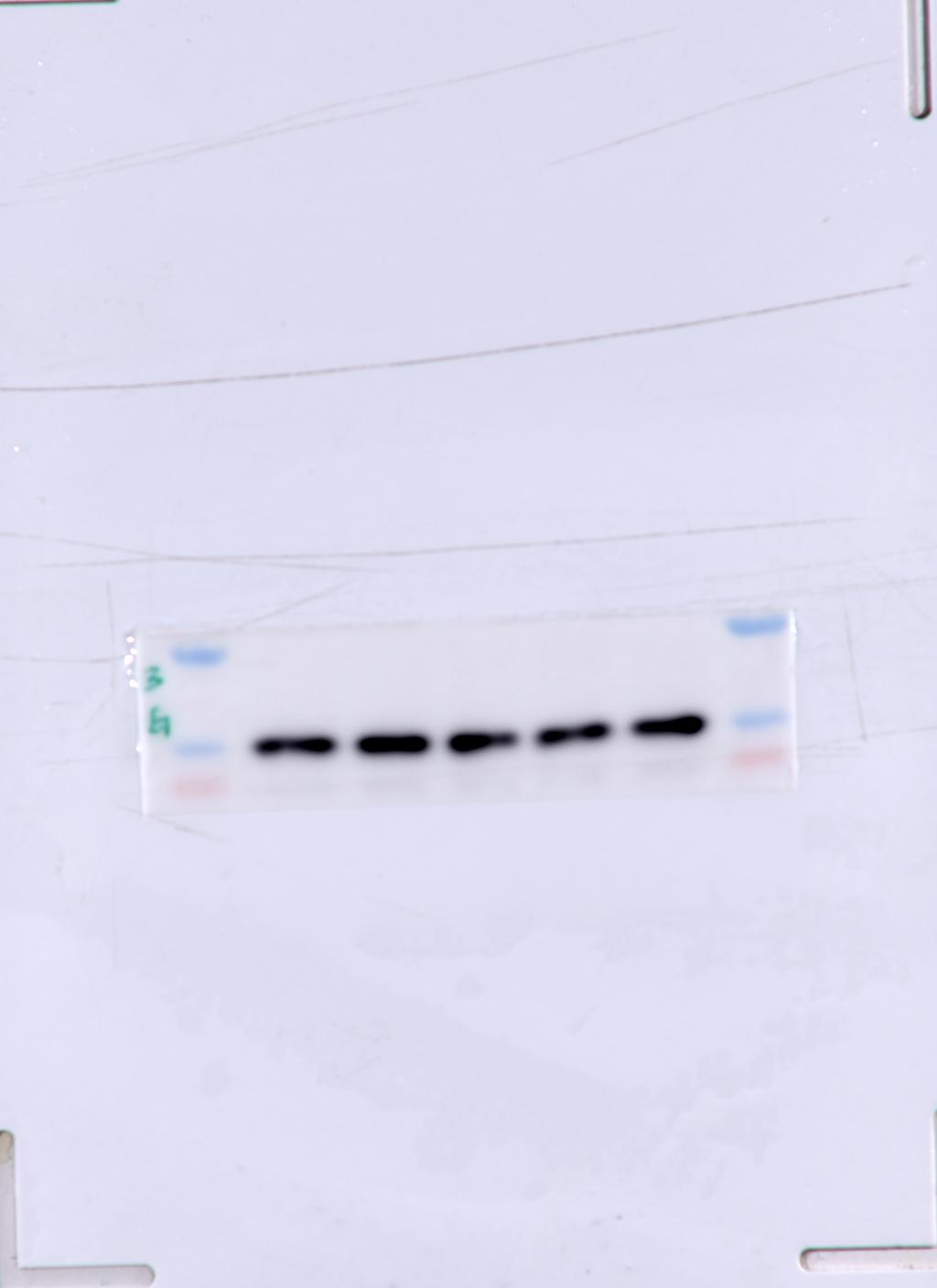

Supplement: Supplementary file 2 — Additional file 2. Raw data of western blot. [file 12974_2022_2632_MOESM2_ESM.zip › supplementary files/Figure5 WB/WT GAPDH/3gapdh 2021.04.24_16.57.16_Ch+Marker.jpg]

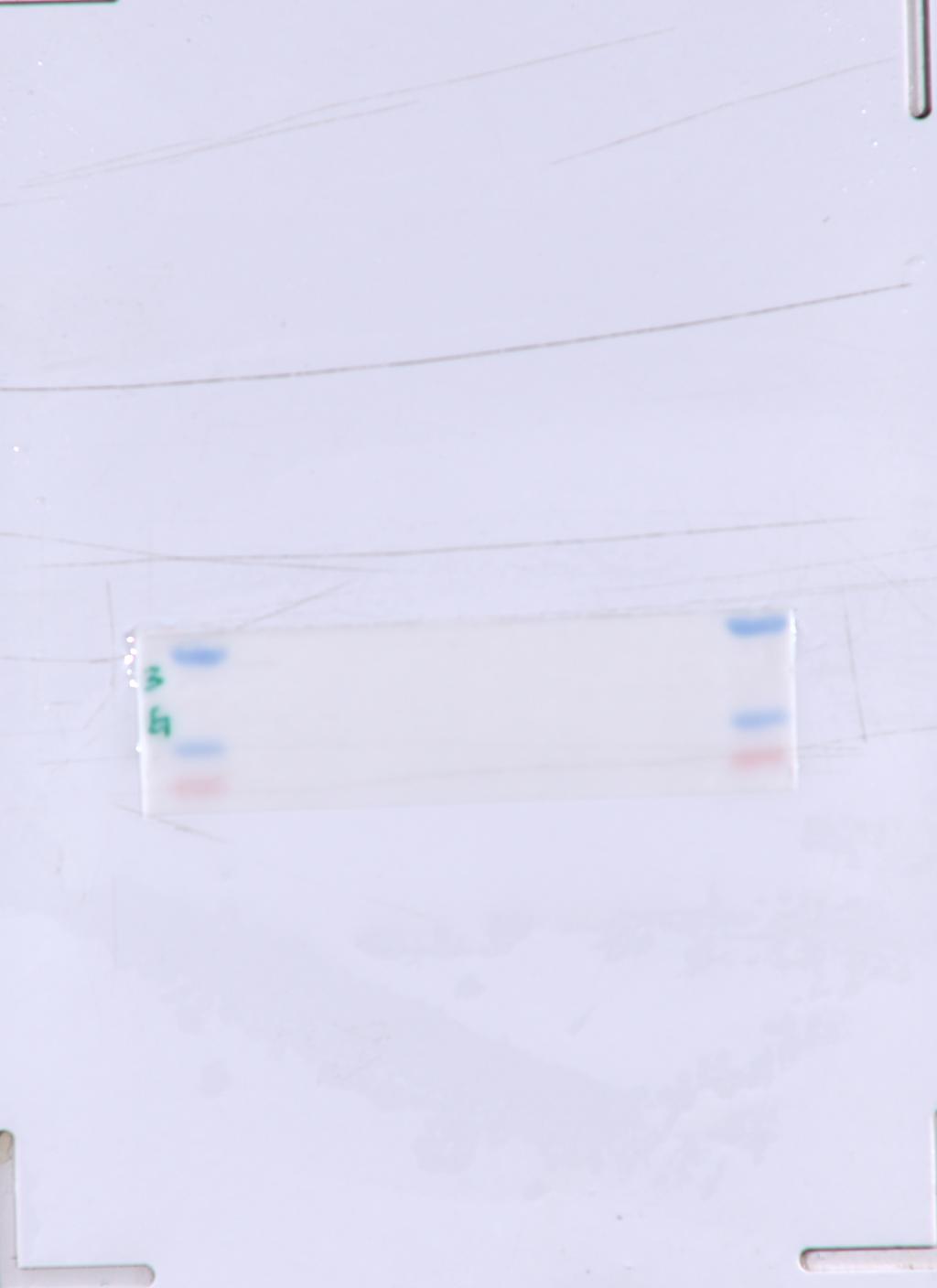

Supplement: Supplementary file 2 — Additional file 2. Raw data of western blot. [file 12974_2022_2632_MOESM2_ESM.zip › supplementary files/Figure5 WB/WT GAPDH/3gapdh 2021.04.24_16.57.16_Ch-Marker.jpg]

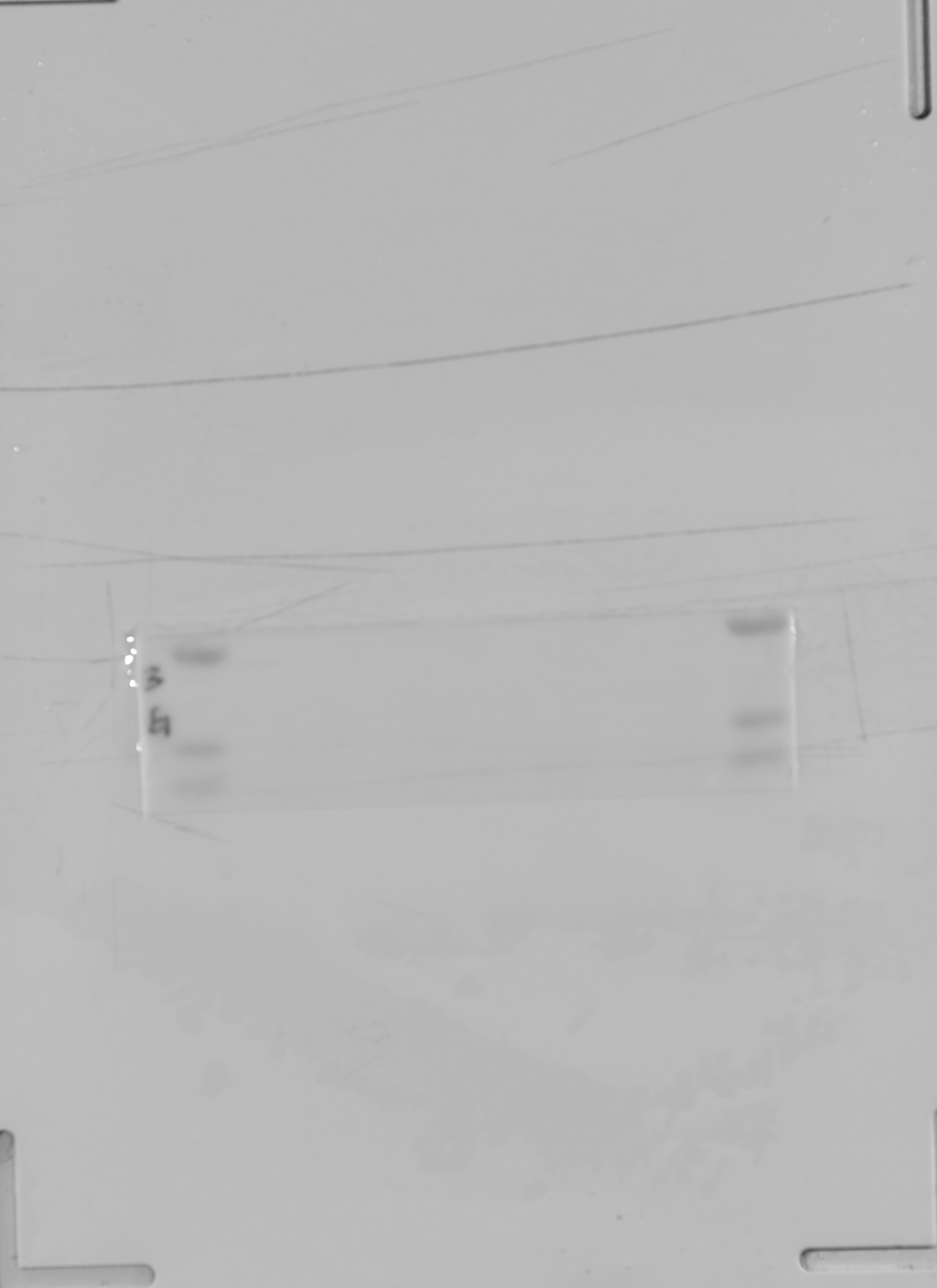

Supplement: Supplementary file 2 — Additional file 2. Raw data of western blot. [file 12974_2022_2632_MOESM2_ESM.zip › supplementary files/Figure5 WB/WT GAPDH/3gapdh 2021.04.24_16.57.16_Ch-Marker.tif]

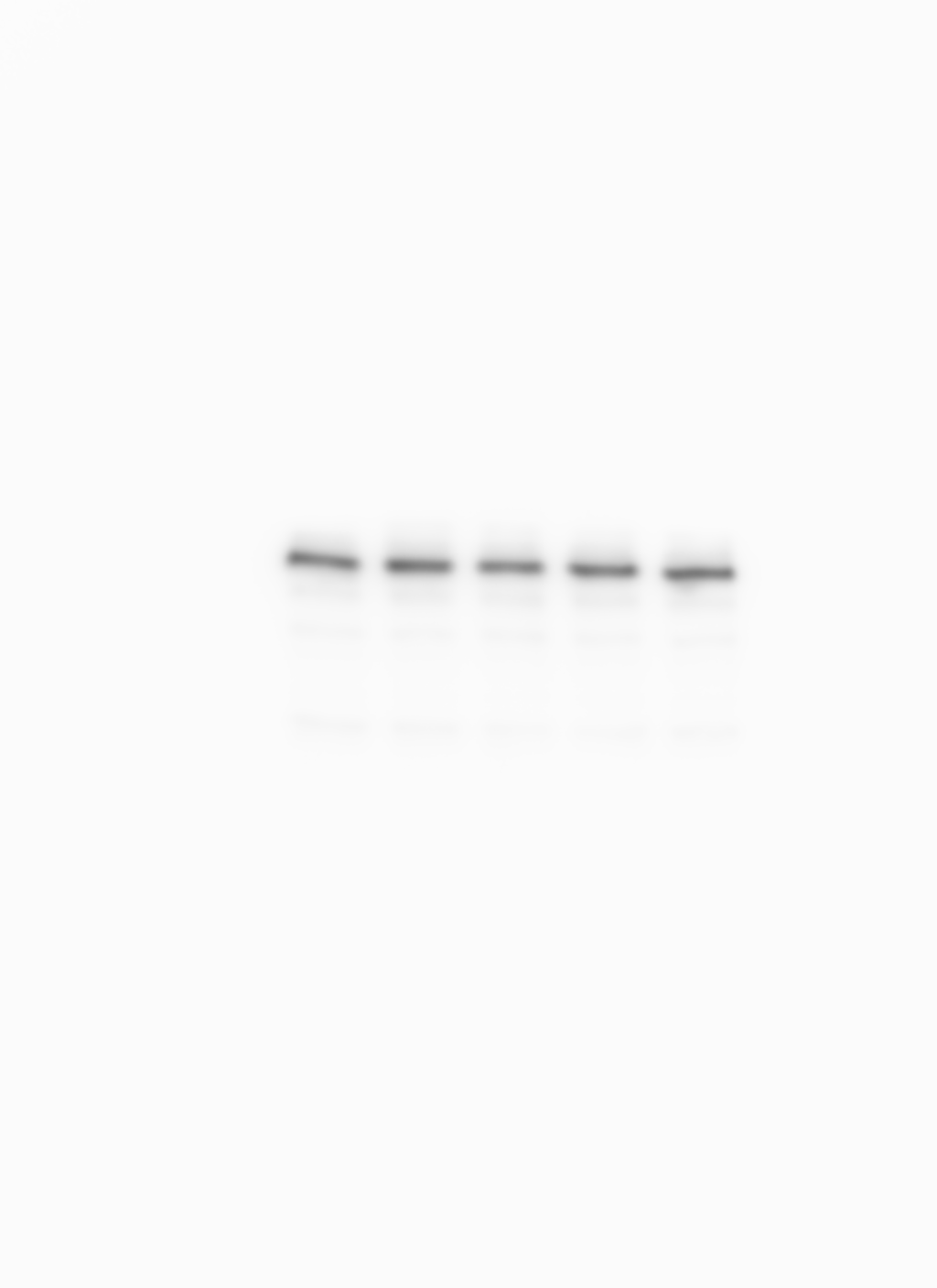

Supplement: Supplementary file 2 — Additional file 2. Raw data of western blot. [file 12974_2022_2632_MOESM2_ESM.zip › supplementary files/Figure5 WB/WT mtor/3 mtor 2021.04.24_17.05.21_Ch.tif]

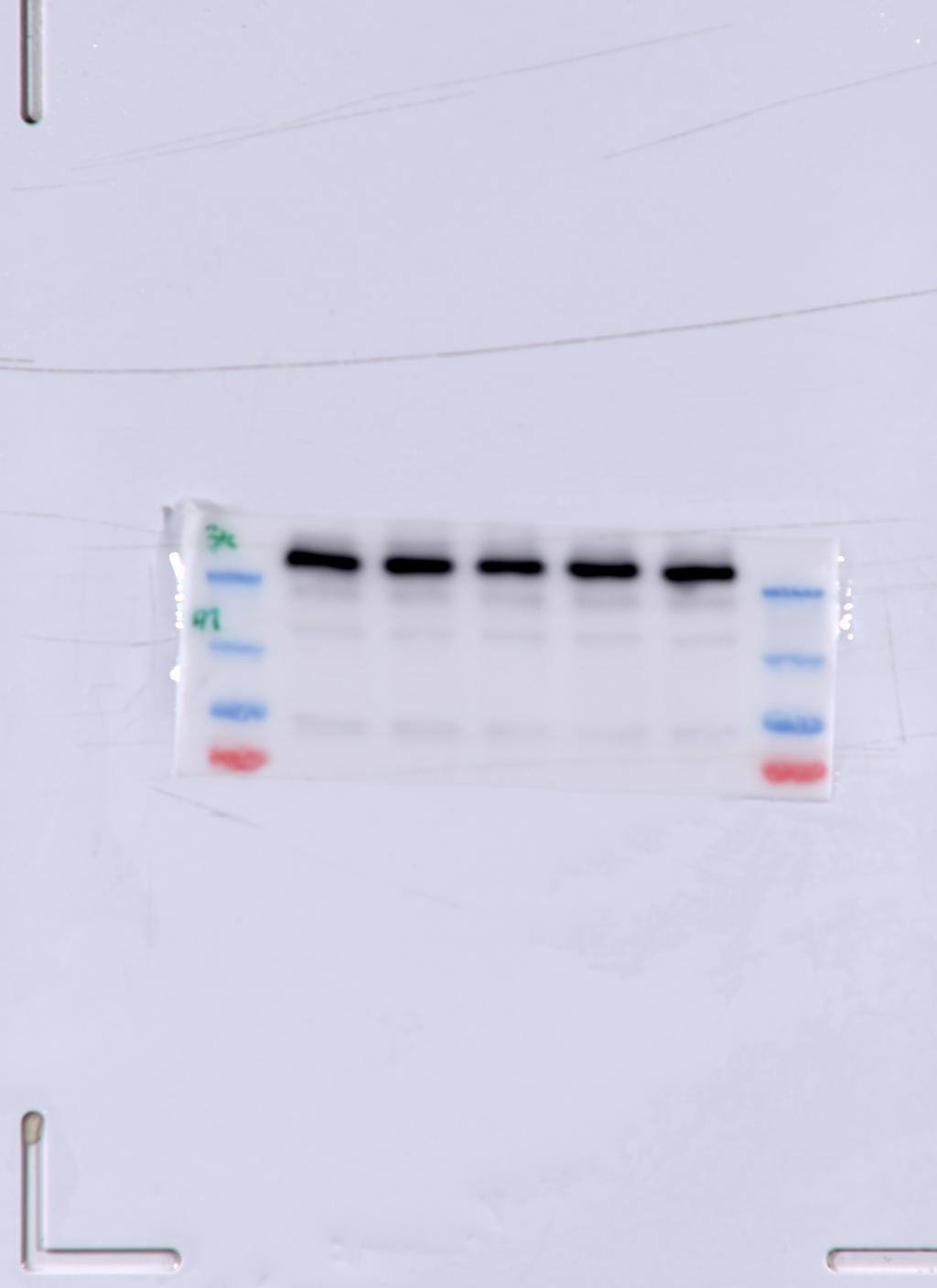

Supplement: Supplementary file 2 — Additional file 2. Raw data of western blot. [file 12974_2022_2632_MOESM2_ESM.zip › supplementary files/Figure5 WB/WT mtor/3 mtor 2021.04.24_17.05.21_Ch+Marker.jpg]

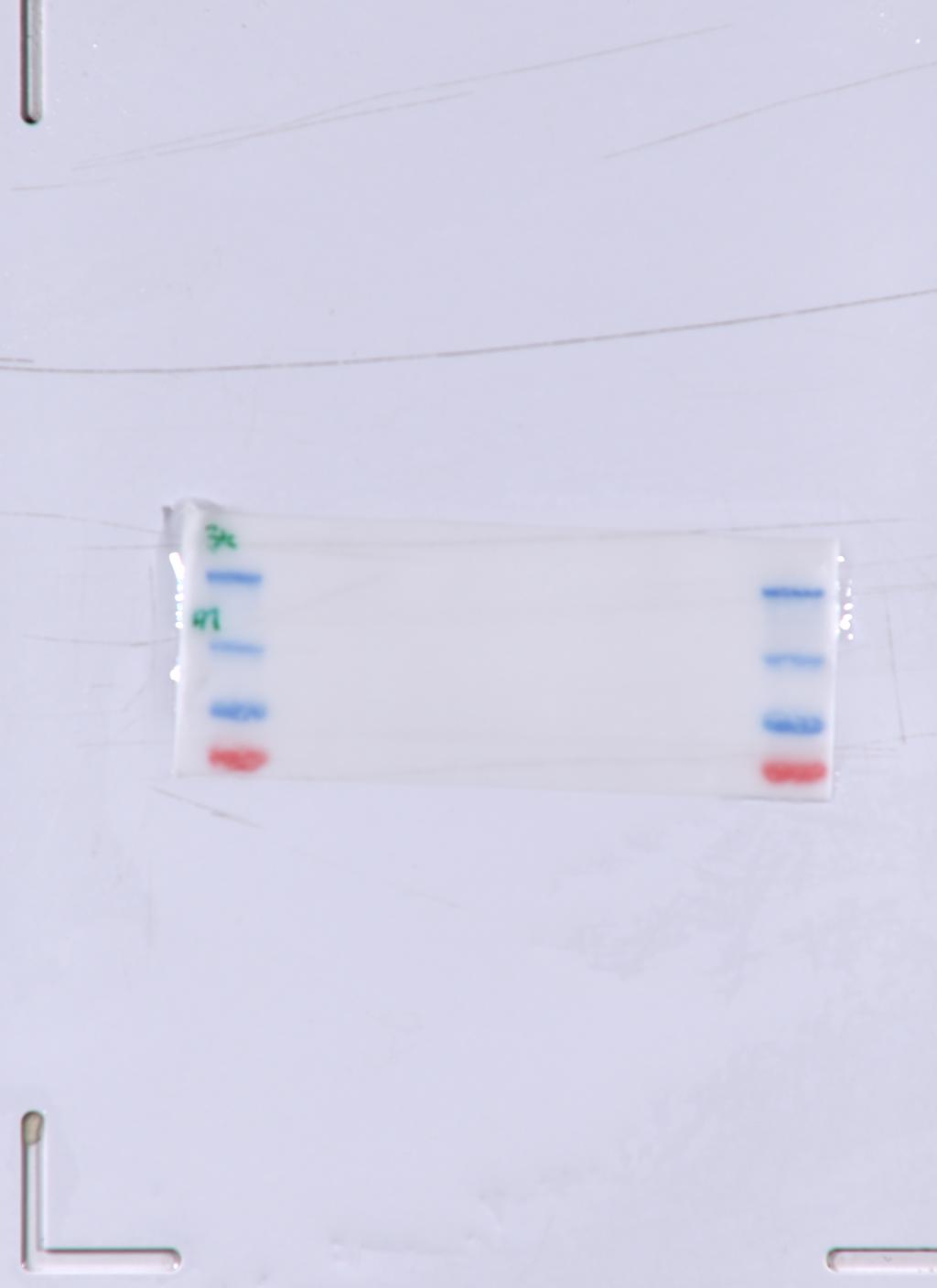

Supplement: Supplementary file 2 — Additional file 2. Raw data of western blot. [file 12974_2022_2632_MOESM2_ESM.zip › supplementary files/Figure5 WB/WT mtor/3 mtor 2021.04.24_17.05.21_Ch-Marker.jpg]

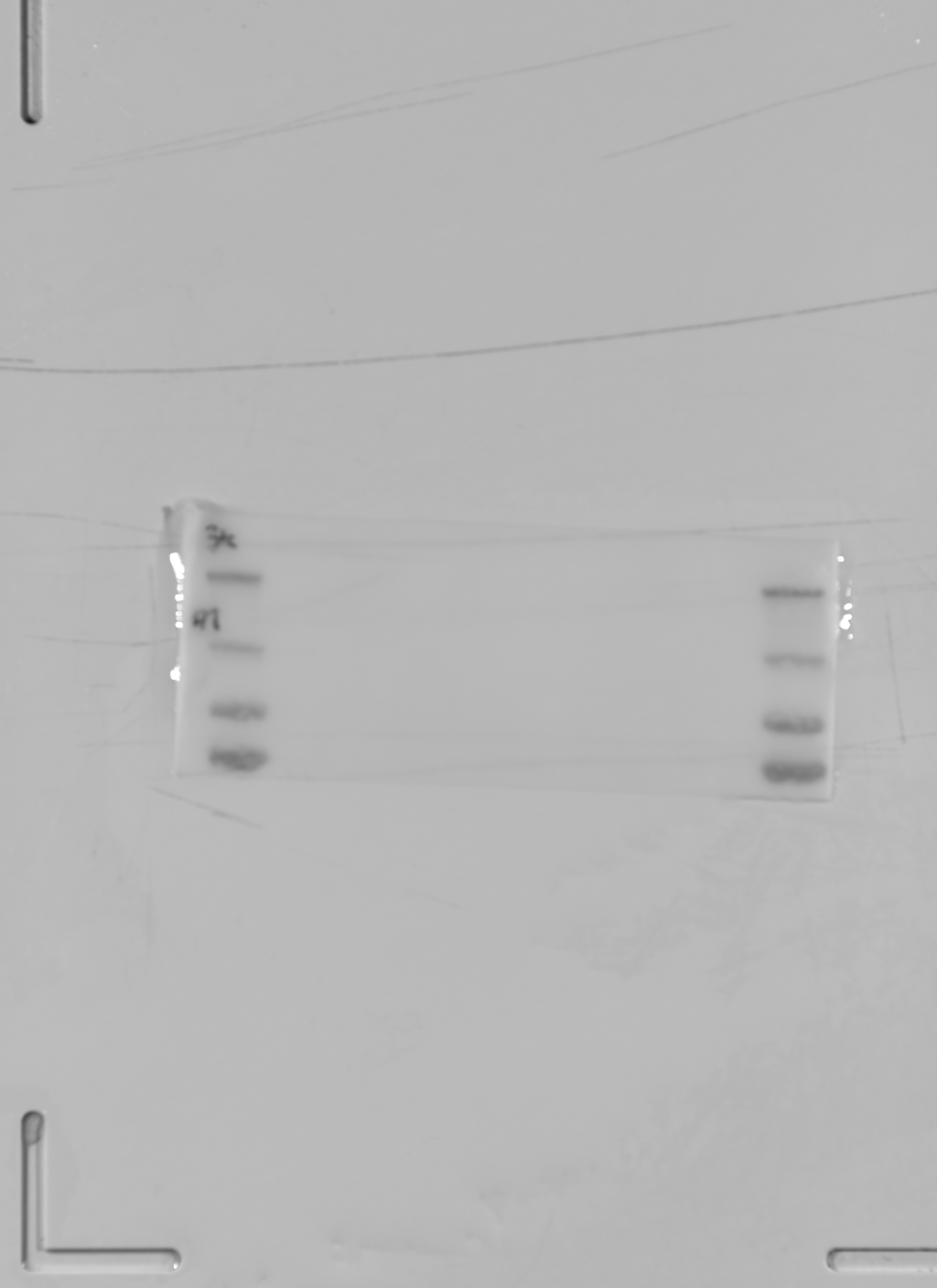

Supplement: Supplementary file 2 — Additional file 2. Raw data of western blot. [file 12974_2022_2632_MOESM2_ESM.zip › supplementary files/Figure5 WB/WT mtor/3 mtor 2021.04.24_17.05.21_Ch-Marker.tif]

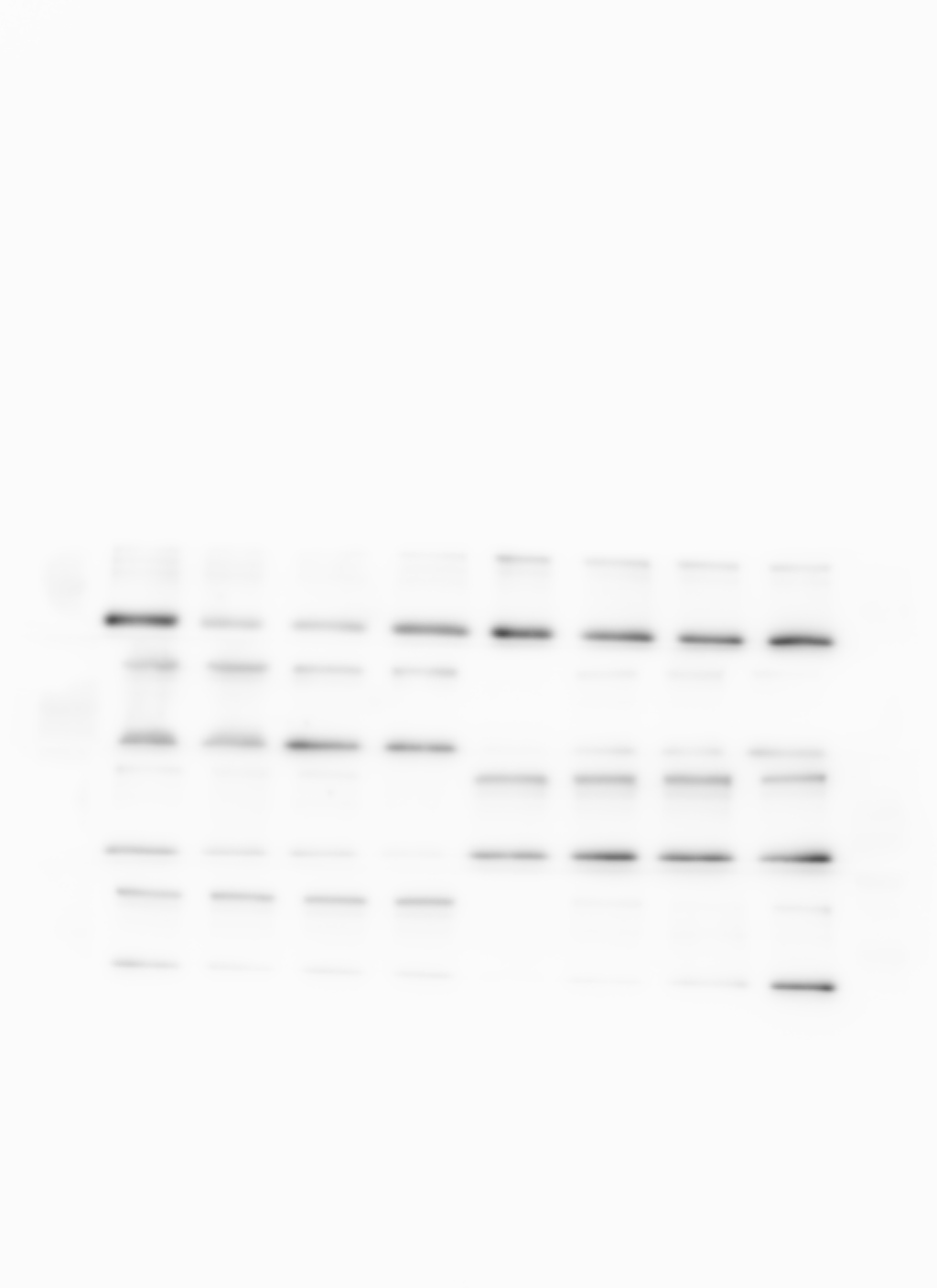

Supplement: Supplementary file 2 — Additional file 2. Raw data of western blot. [file 12974_2022_2632_MOESM2_ESM.zip › supplementary files/Figure5 WB/WT Sirt1/sirt1 2020.08.07_19.12.34_Ch.tif]

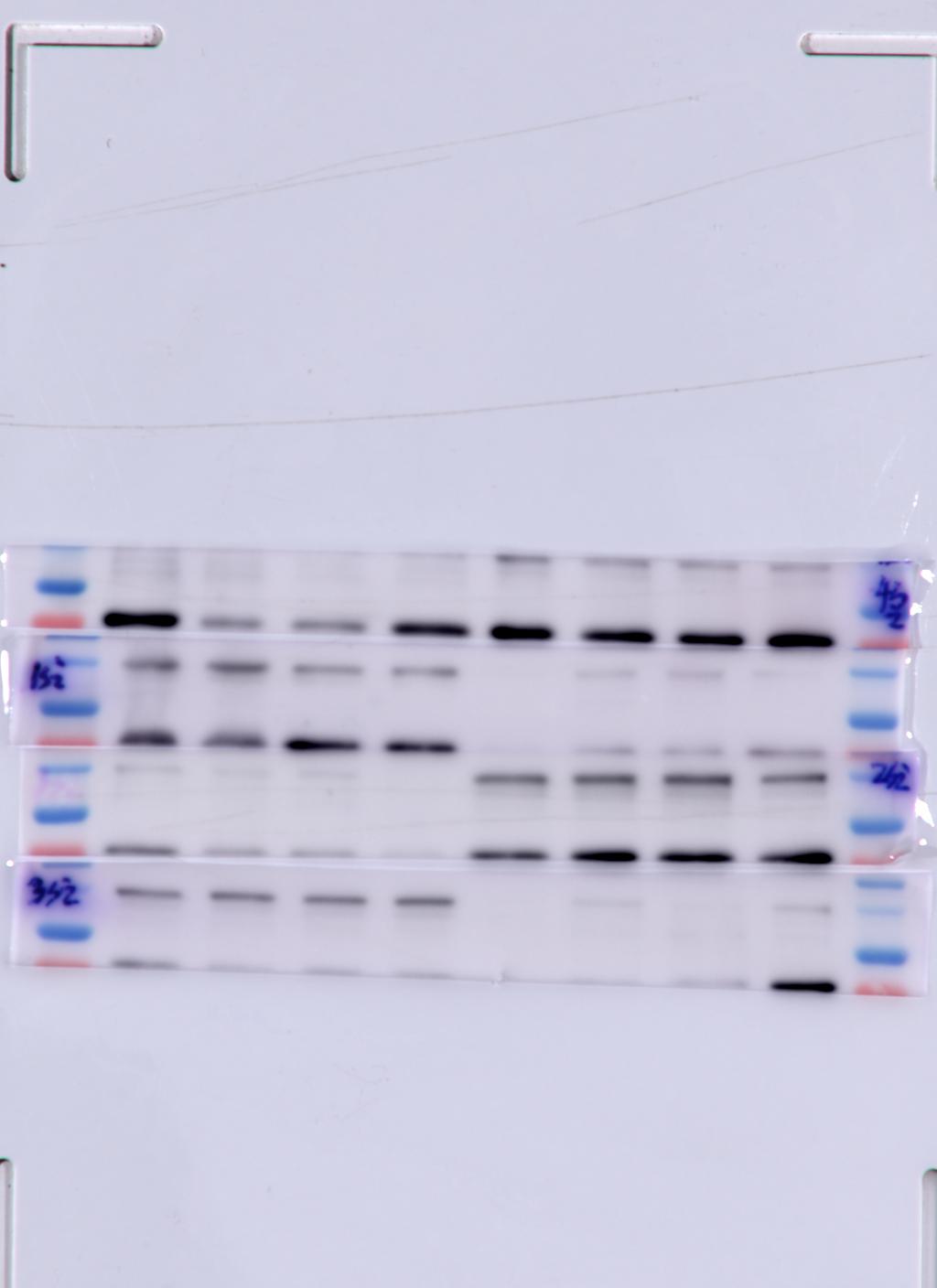

Supplement: Supplementary file 2 — Additional file 2. Raw data of western blot. [file 12974_2022_2632_MOESM2_ESM.zip › supplementary files/Figure5 WB/WT Sirt1/sirt1 2020.08.07_19.12.34_Ch+Marker.jpg]

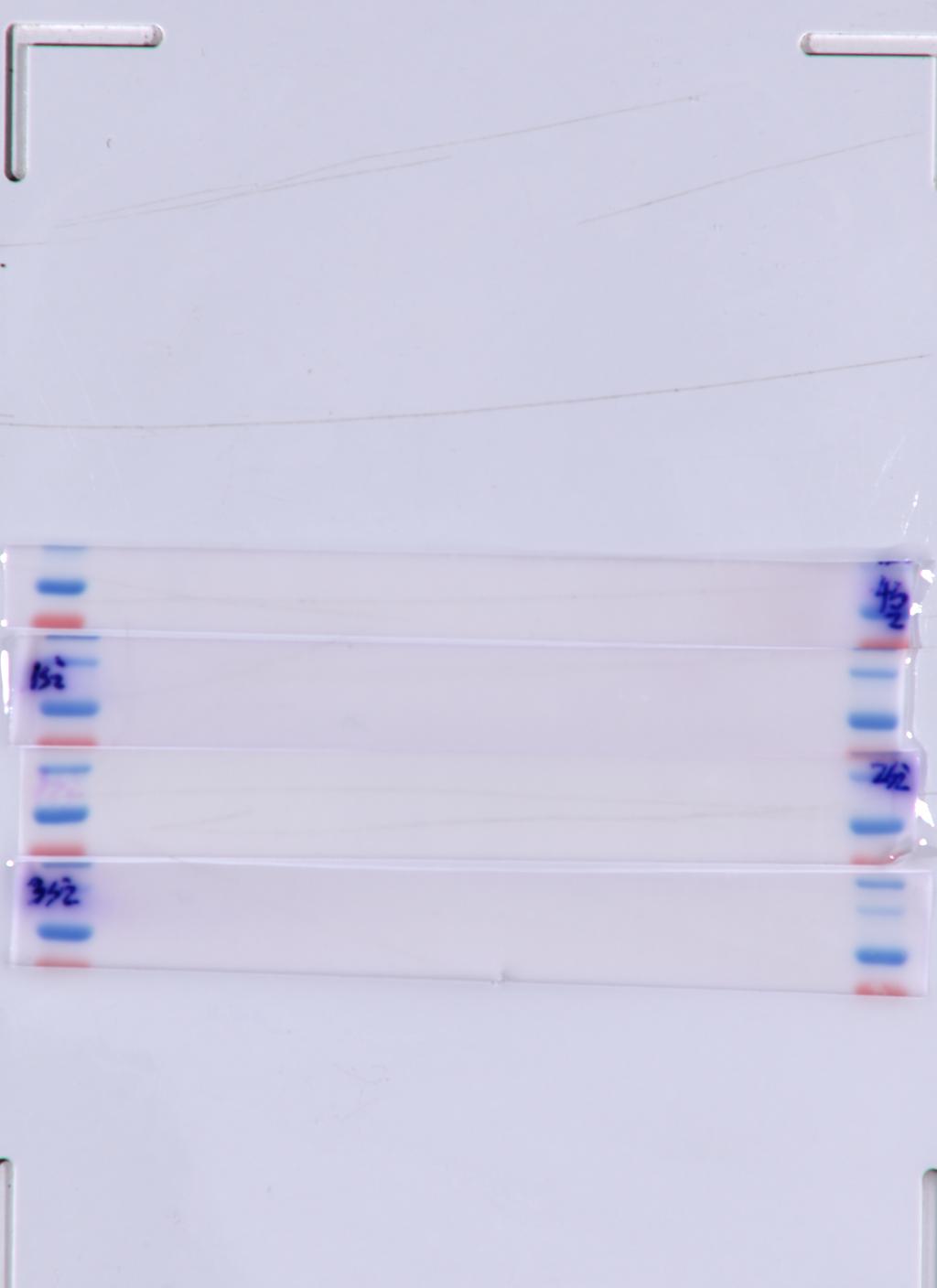

Supplement: Supplementary file 2 — Additional file 2. Raw data of western blot. [file 12974_2022_2632_MOESM2_ESM.zip › supplementary files/Figure5 WB/WT Sirt1/sirt1 2020.08.07_19.12.34_Ch-Marker.jpg]

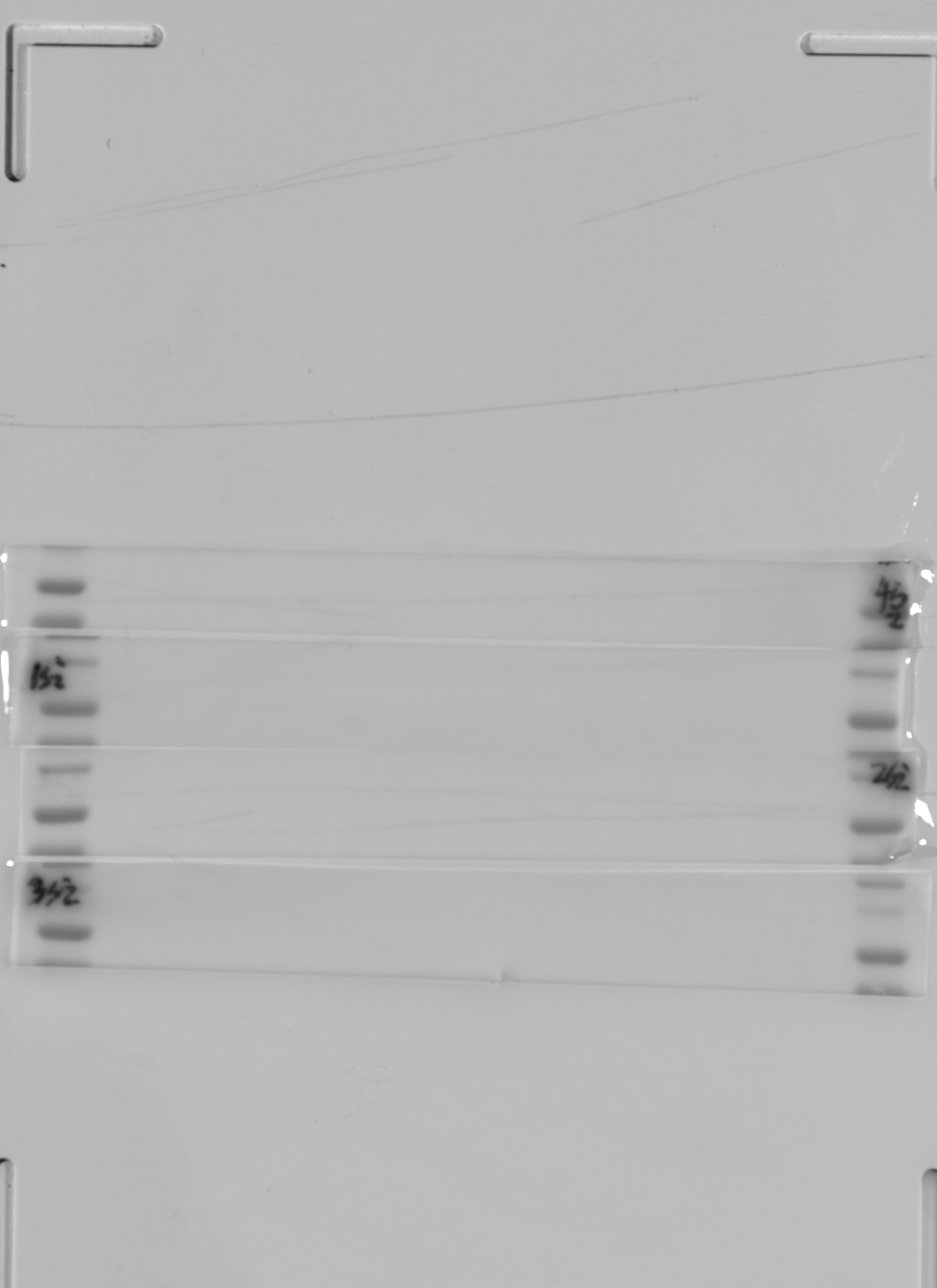

Supplement: Supplementary file 2 — Additional file 2. Raw data of western blot. [file 12974_2022_2632_MOESM2_ESM.zip › supplementary files/Figure5 WB/WT Sirt1/sirt1 2020.08.07_19.12.34_Ch-Marker.tif]

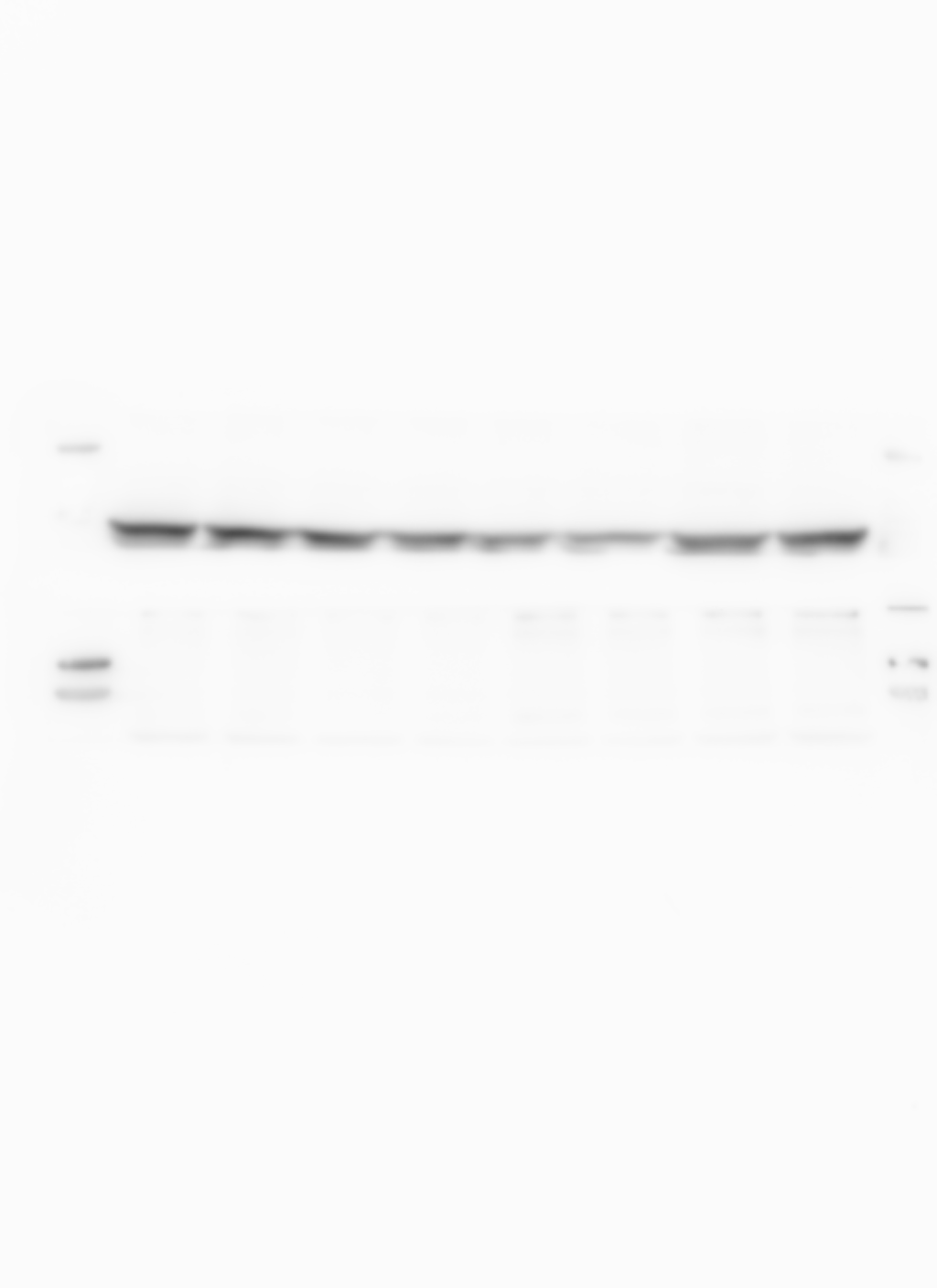

Supplement: Supplementary file 2 — Additional file 2. Raw data of western blot. [file 12974_2022_2632_MOESM2_ESM.zip › supplementary files/Figure5 WB/WT-pampk/pampk 2020.08.18_16.41.14_Ch.tif]

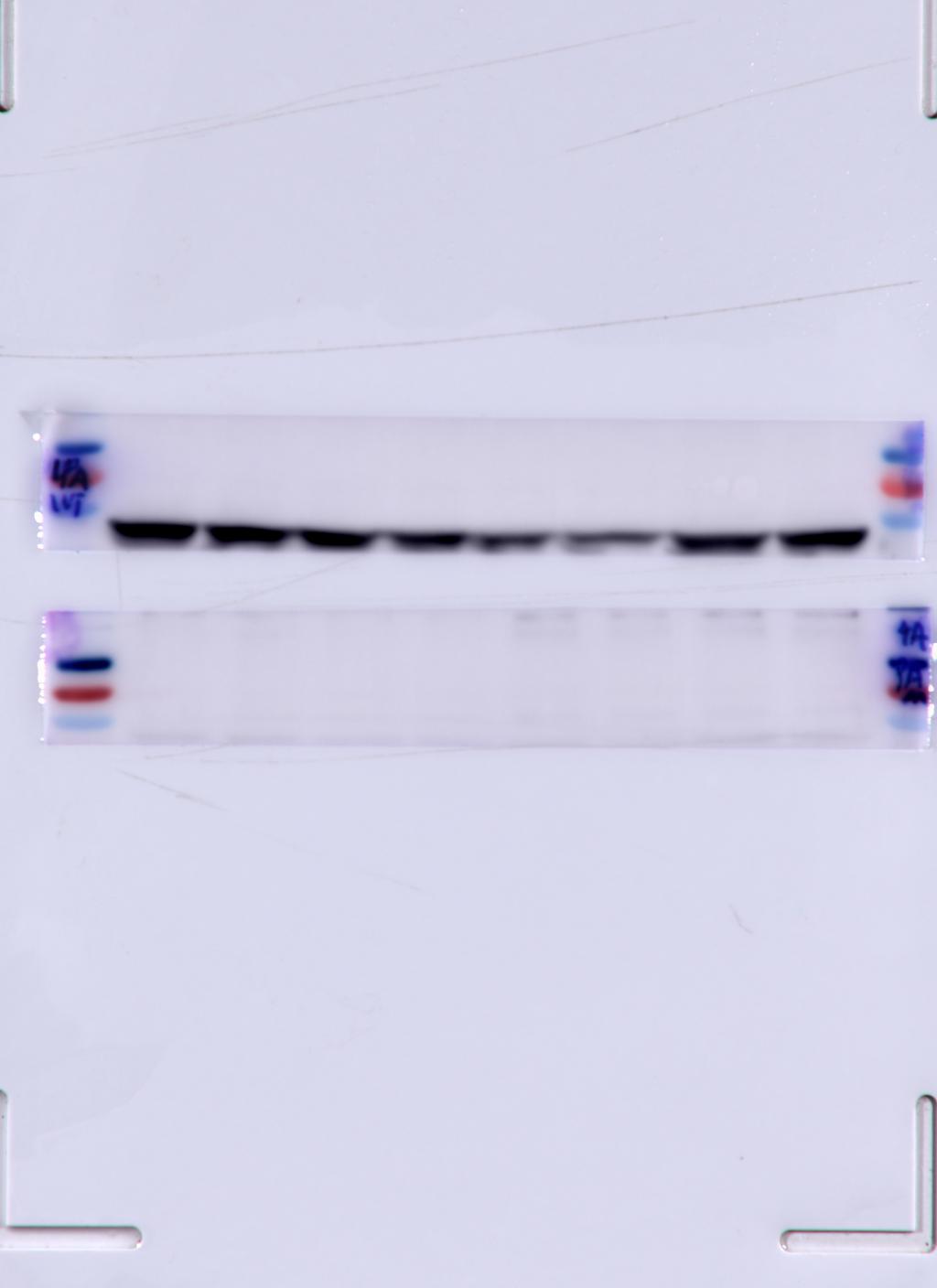

Supplement: Supplementary file 2 — Additional file 2. Raw data of western blot. [file 12974_2022_2632_MOESM2_ESM.zip › supplementary files/Figure5 WB/WT-pampk/pampk 2020.08.18_16.41.14_Ch+Marker.jpg]

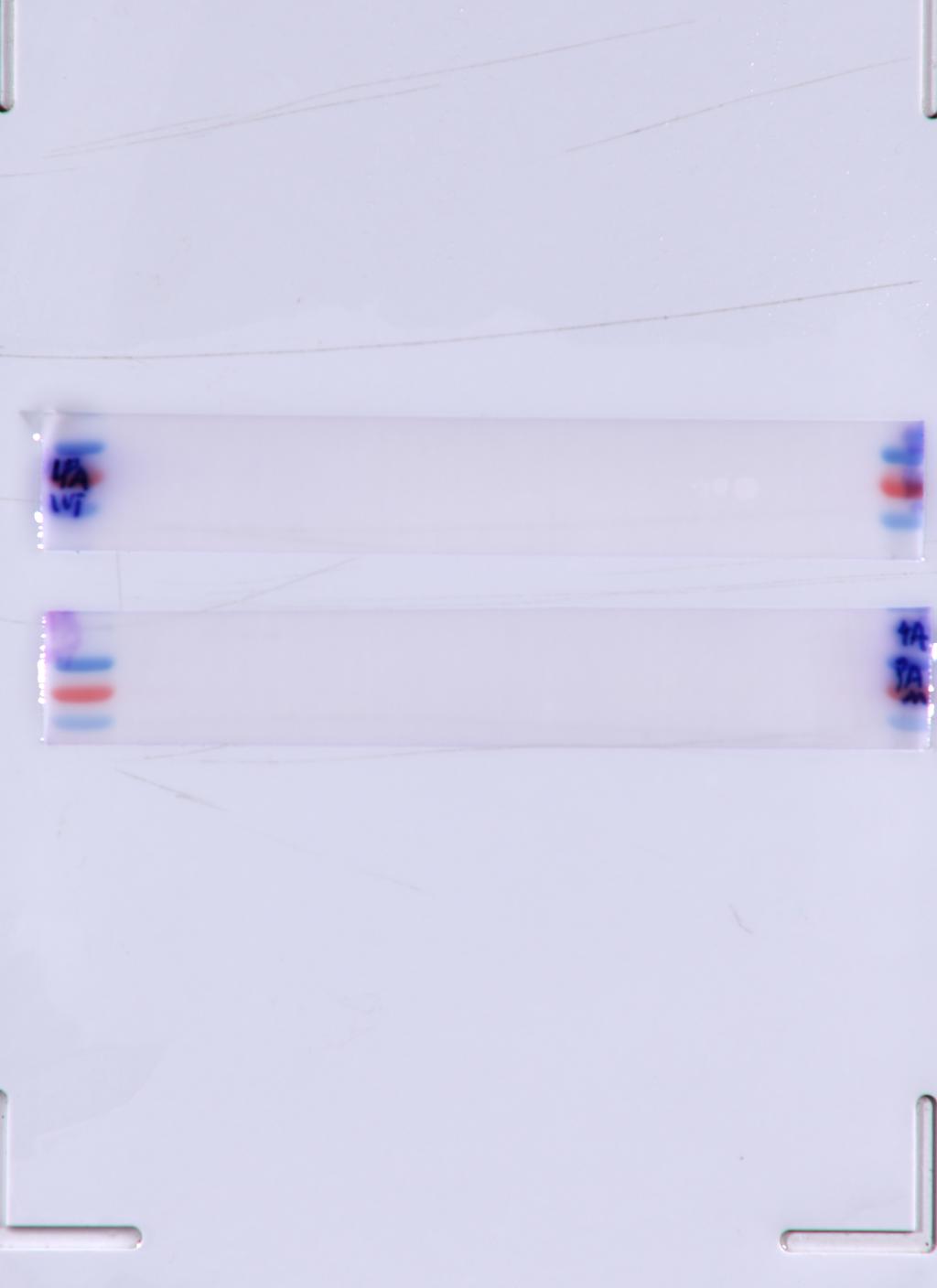

Supplement: Supplementary file 2 — Additional file 2. Raw data of western blot. [file 12974_2022_2632_MOESM2_ESM.zip › supplementary files/Figure5 WB/WT-pampk/pampk 2020.08.18_16.41.14_Ch-Marker.jpg]

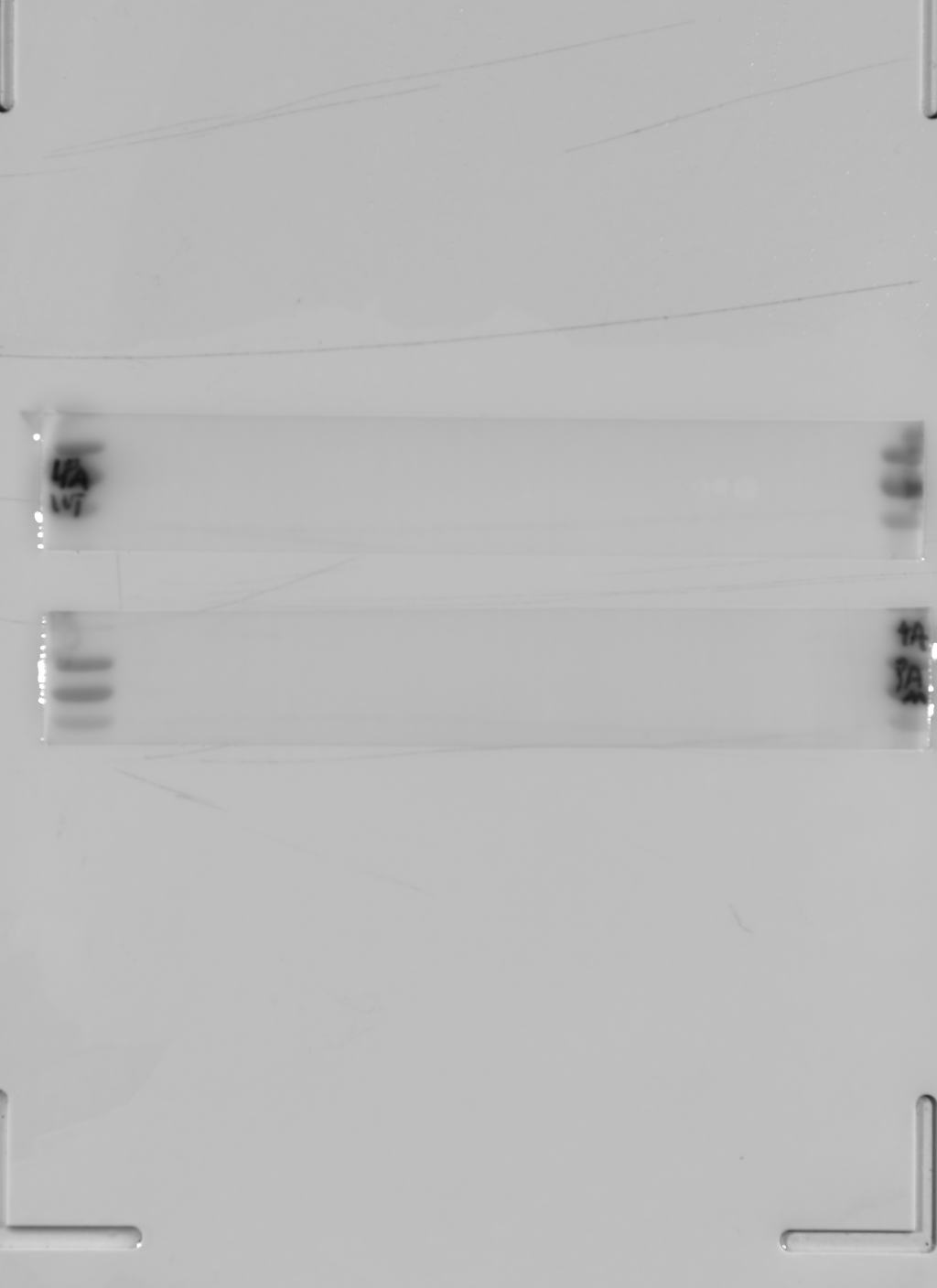

Supplement: Supplementary file 2 — Additional file 2. Raw data of western blot. [file 12974_2022_2632_MOESM2_ESM.zip › supplementary files/Figure5 WB/WT-pampk/pampk 2020.08.18_16.41.14_Ch-Marker.tif]

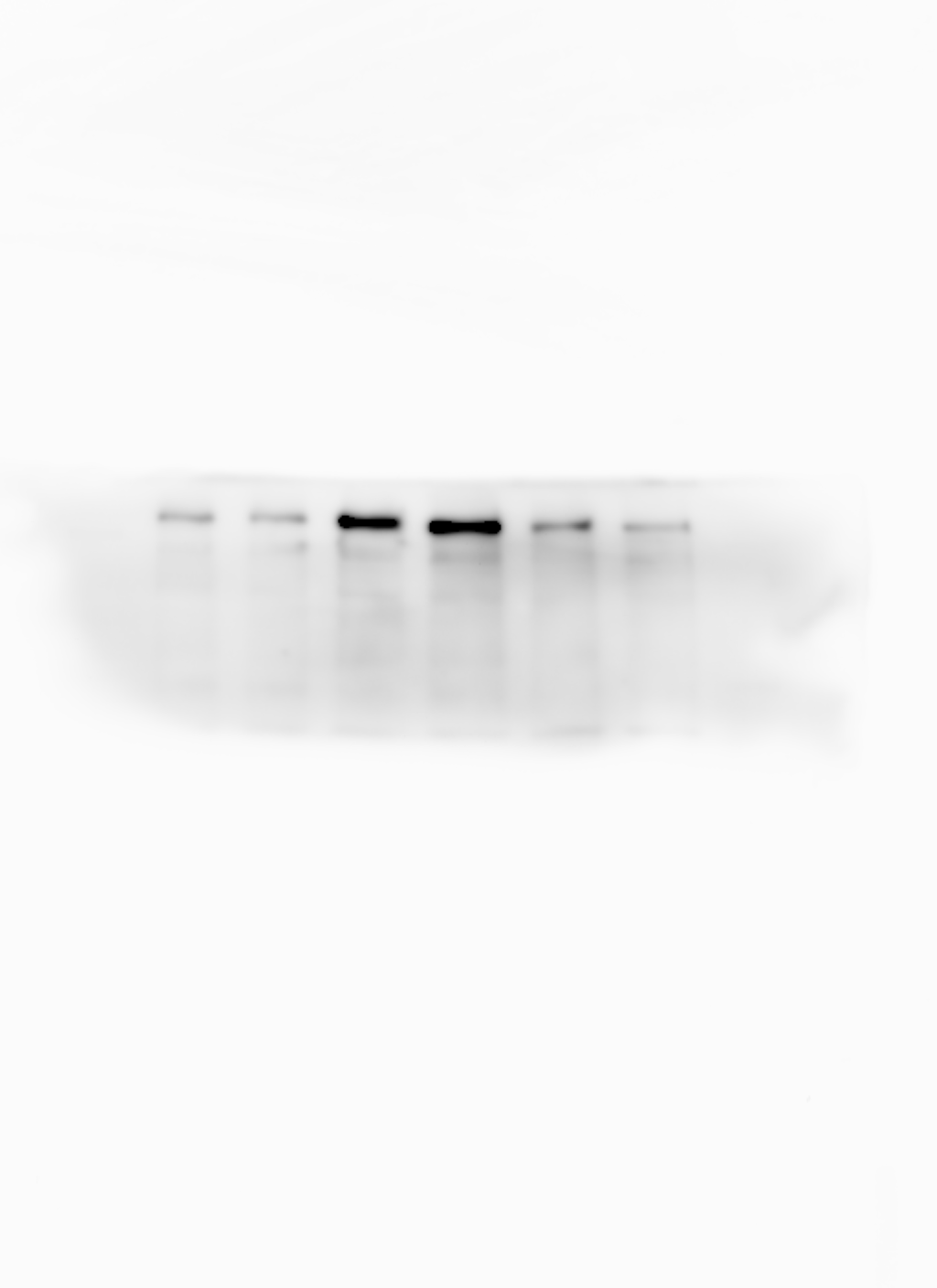

Supplement: Supplementary file 2 — Additional file 2. Raw data of western blot. [file 12974_2022_2632_MOESM2_ESM.zip › supplementary files/Figure5 WB/WT-pmtor/1-mtor 2020.09.18_11.59.16_Ch.tif]

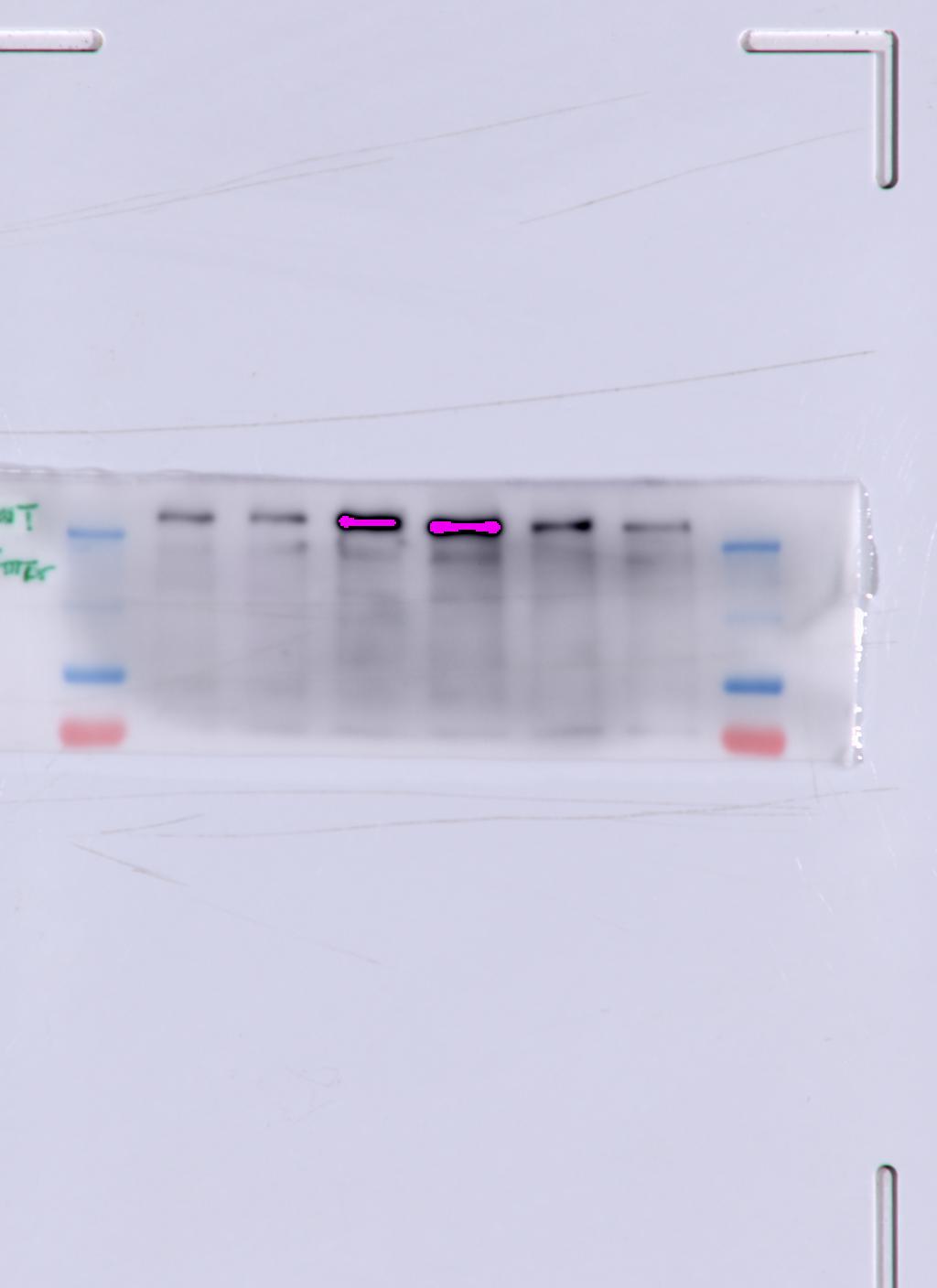

Supplement: Supplementary file 2 — Additional file 2. Raw data of western blot. [file 12974_2022_2632_MOESM2_ESM.zip › supplementary files/Figure5 WB/WT-pmtor/1-mtor 2020.09.18_11.59.16_Ch+Marker.jpg]

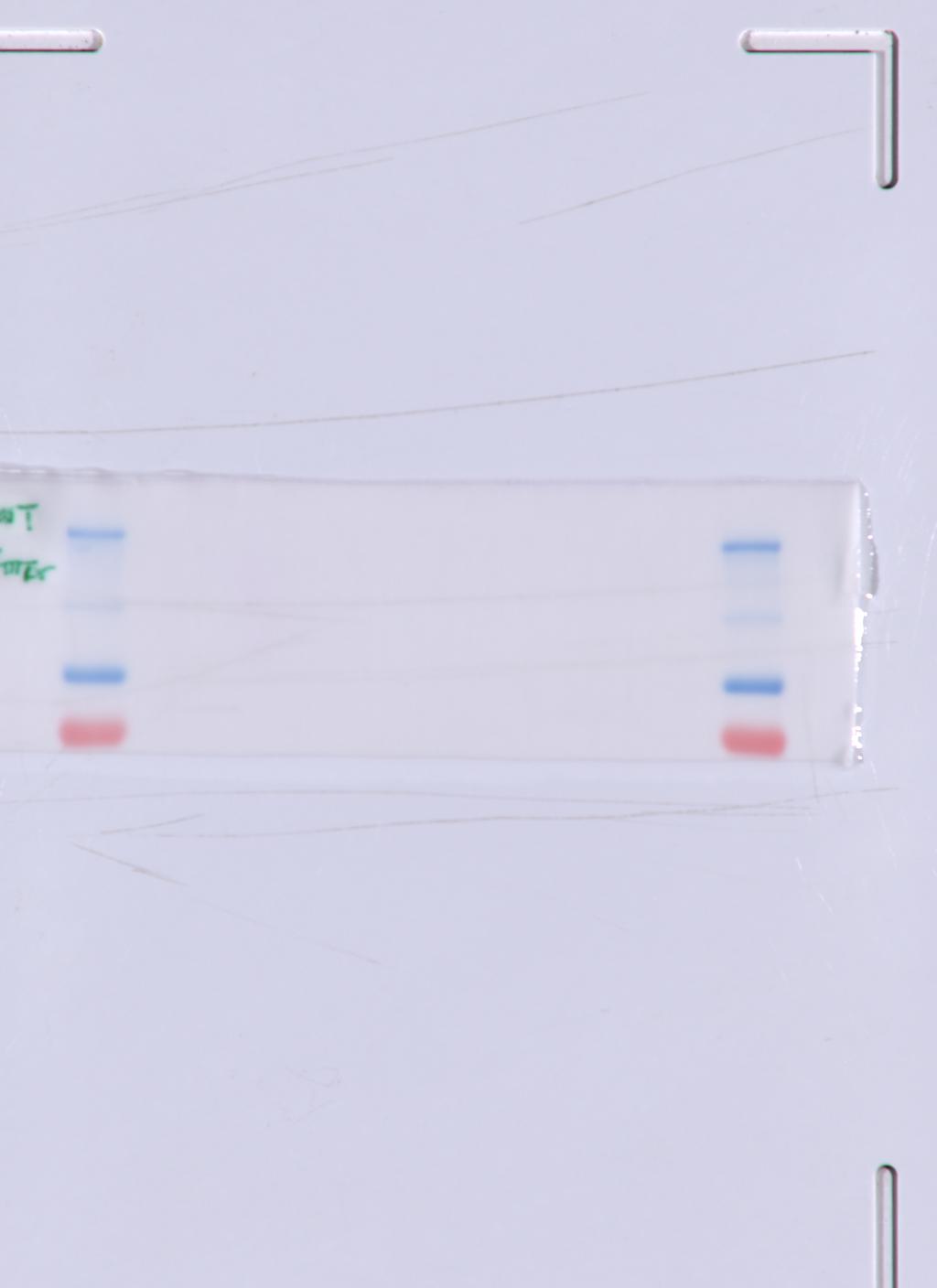

Supplement: Supplementary file 2 — Additional file 2. Raw data of western blot. [file 12974_2022_2632_MOESM2_ESM.zip › supplementary files/Figure5 WB/WT-pmtor/1-mtor 2020.09.18_11.59.16_Ch-Marker.jpg]

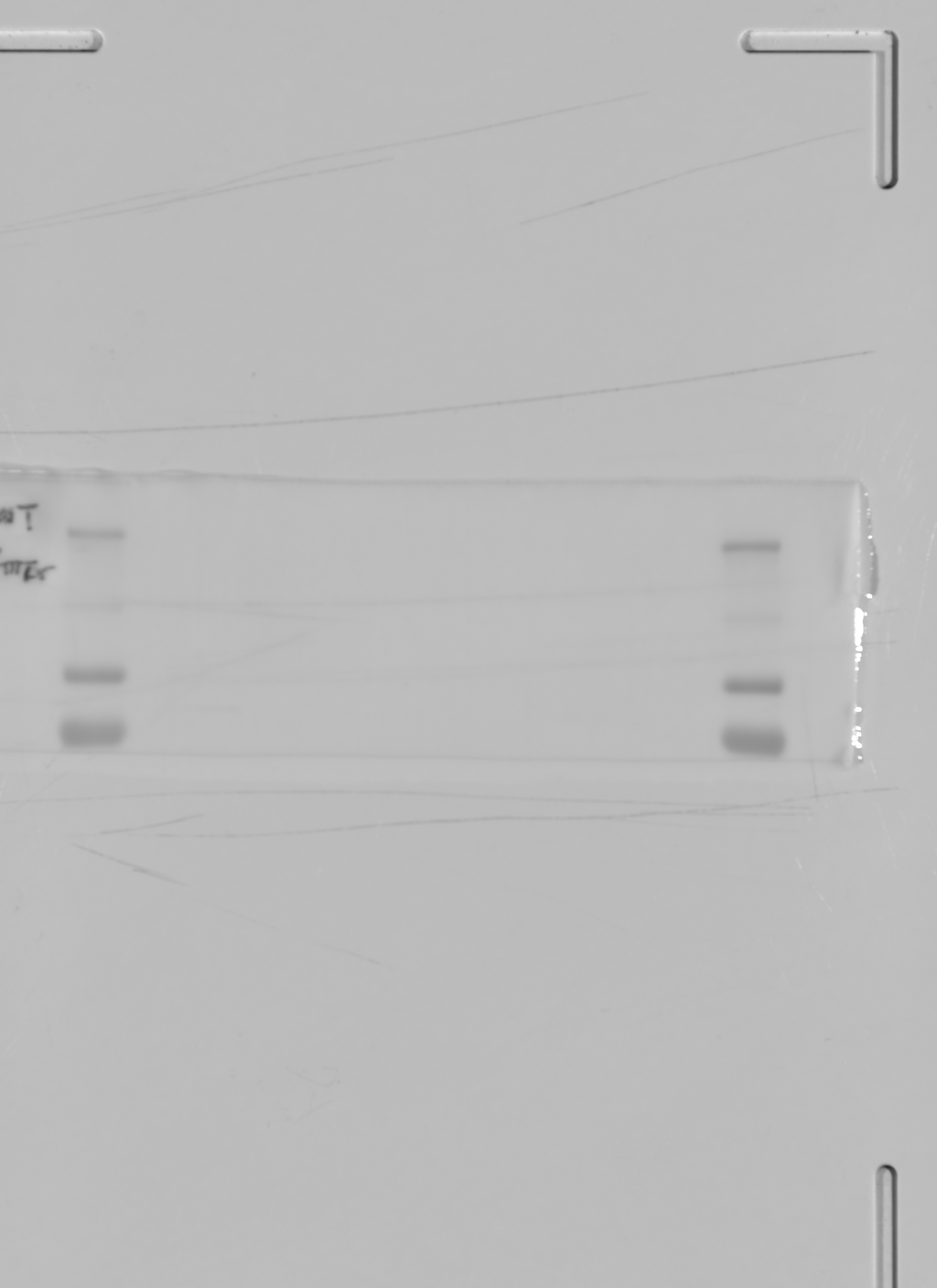

Supplement: Supplementary file 2 — Additional file 2. Raw data of western blot. [file 12974_2022_2632_MOESM2_ESM.zip › supplementary files/Figure5 WB/WT-pmtor/1-mtor 2020.09.18_11.59.16_Ch-Marker.tif]

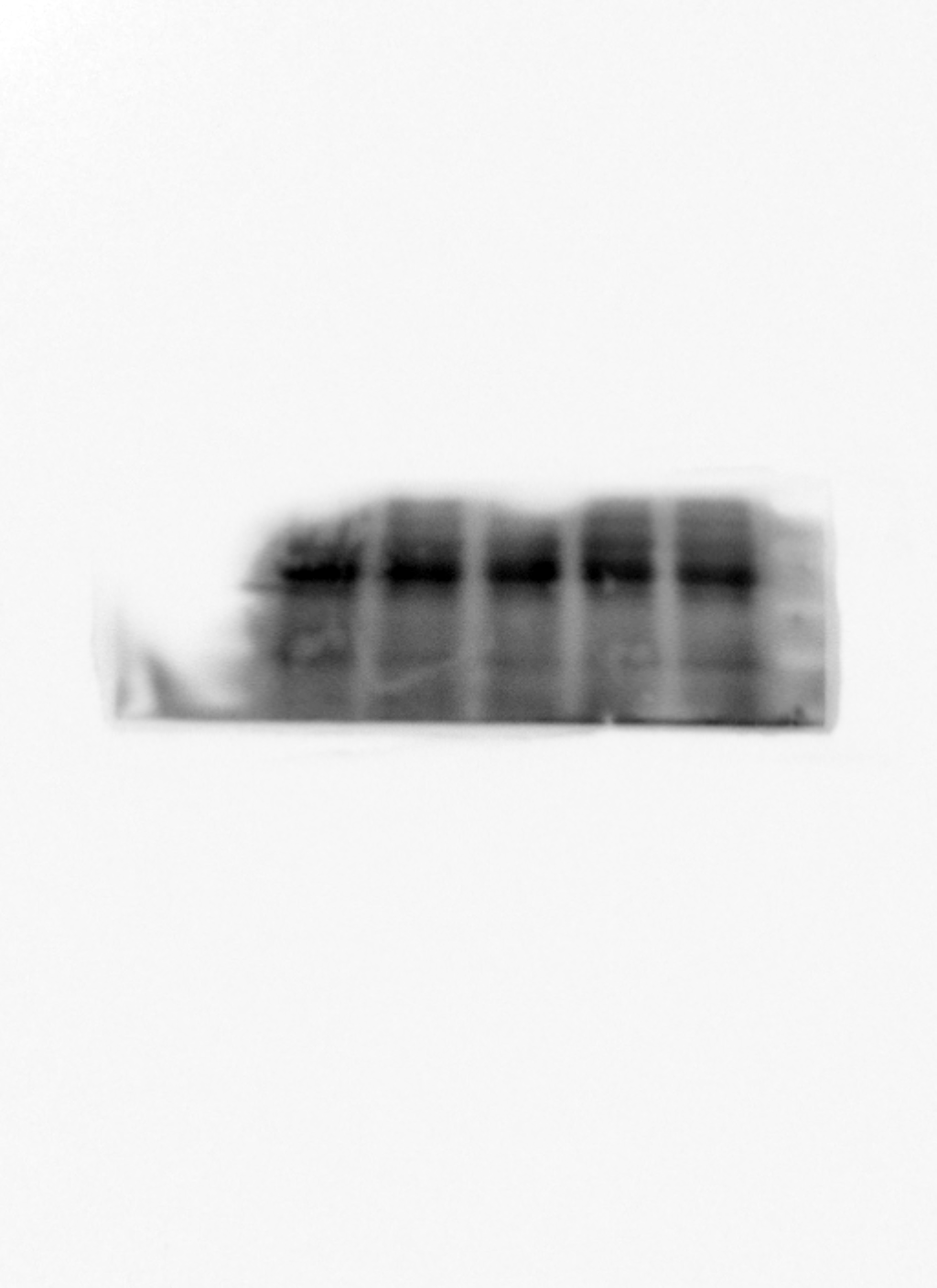

Supplement: Supplementary file 2 — Additional file 2. Raw data of western blot. [file 12974_2022_2632_MOESM2_ESM.zip › supplementary files/supplementary figure1/ar ar 2020.05.10_16.57.46_Ch/arar 2020.05.10_16.57.46_Ch.tif]

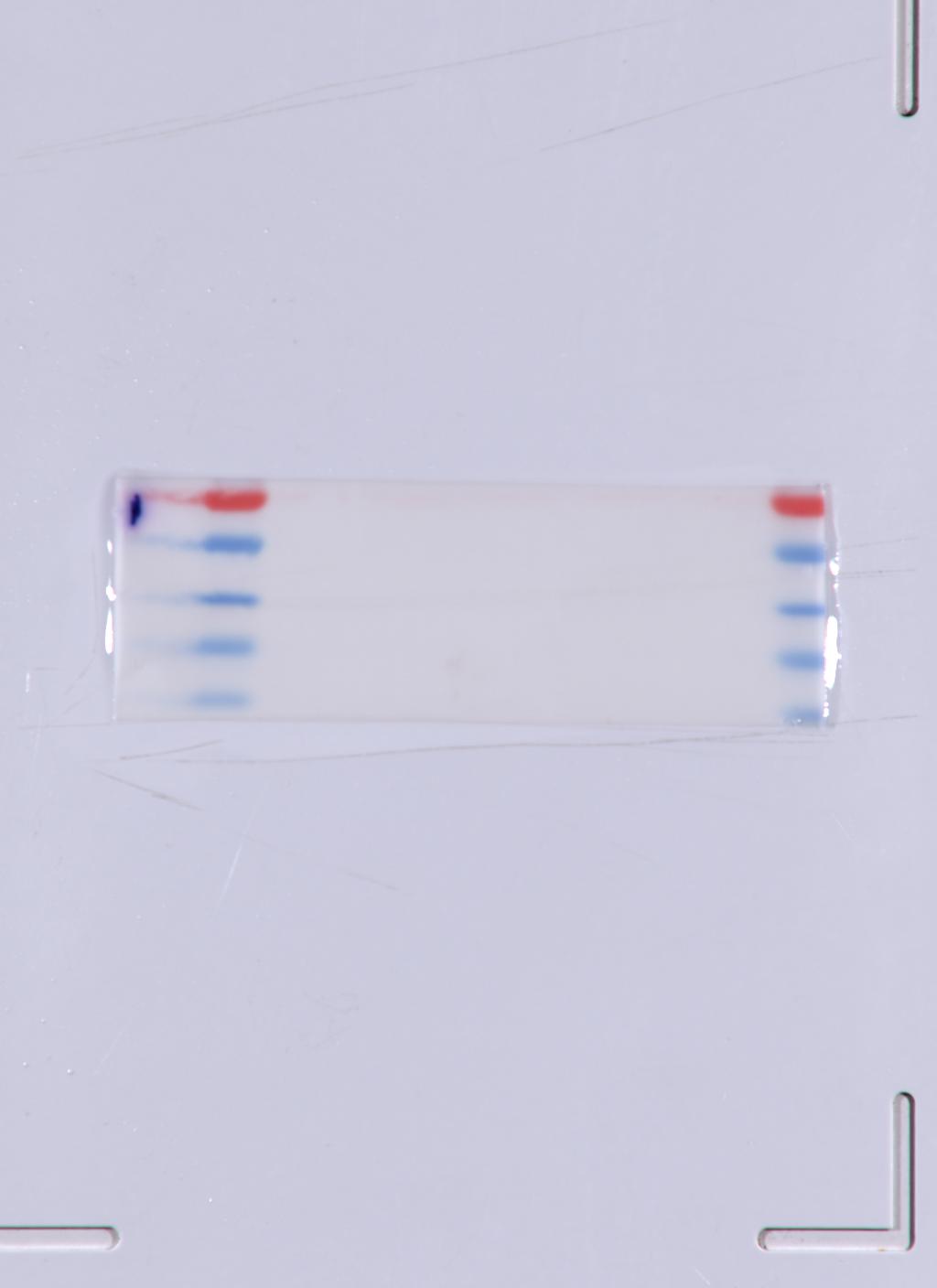

Supplement: Supplementary file 2 — Additional file 2. Raw data of western blot. [file 12974_2022_2632_MOESM2_ESM.zip › supplementary files/supplementary figure1/ar ar 2020.05.10_16.57.46_Ch/arar 2020.05.10_16.57.46_Ch-Marker.jpg]

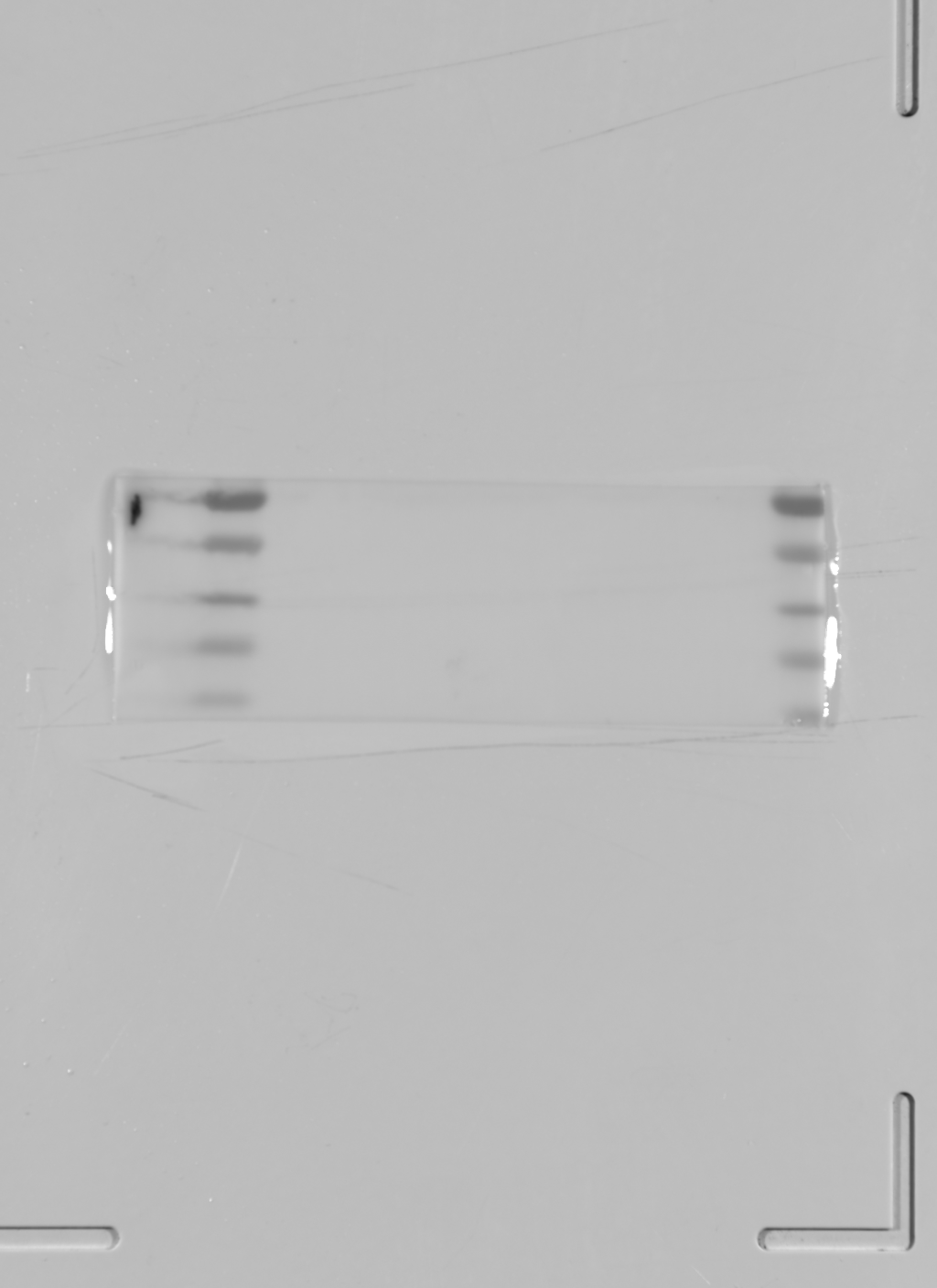

Supplement: Supplementary file 2 — Additional file 2. Raw data of western blot. [file 12974_2022_2632_MOESM2_ESM.zip › supplementary files/supplementary figure1/ar ar 2020.05.10_16.57.46_Ch/arar 2020.05.10_16.57.46_Ch-Marker.tif]

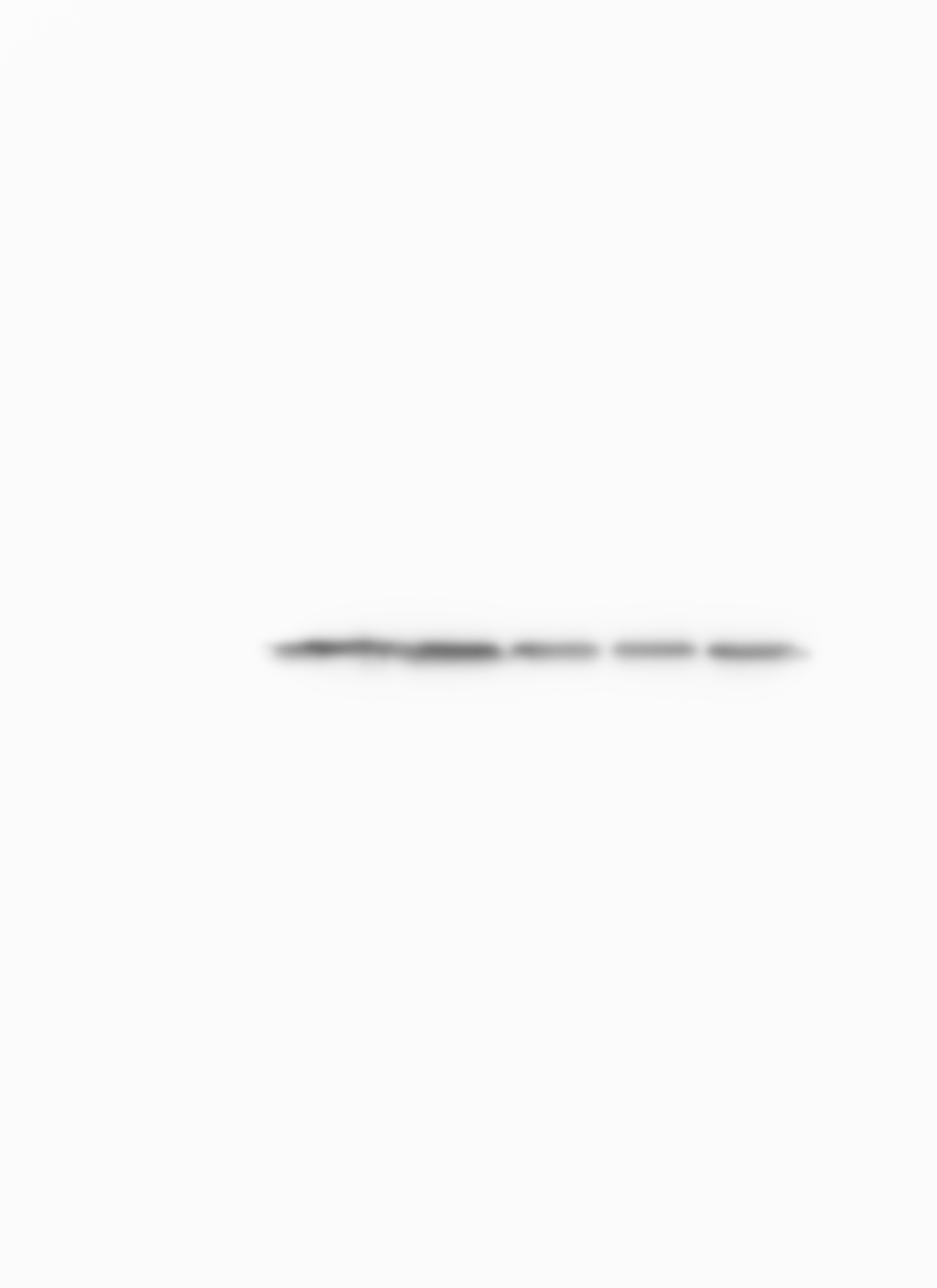

Supplement: Supplementary file 2 — Additional file 2. Raw data of western blot. [file 12974_2022_2632_MOESM2_ESM.zip › supplementary files/supplementary figure1/gapdh 2020.05.12_16.57.51_Ch/gapdh 2020.05.12_16.57.51_Ch.tif]

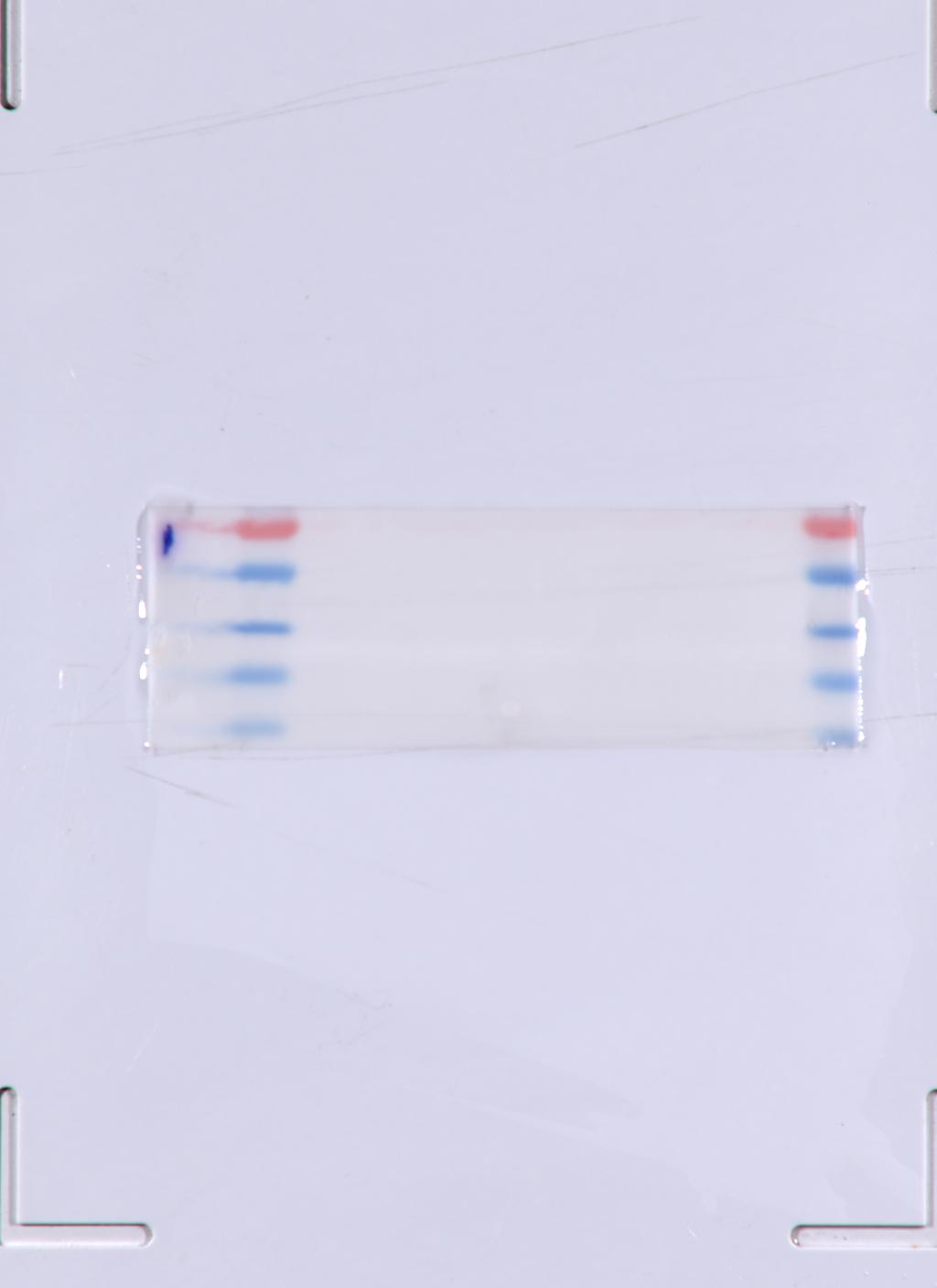

Supplement: Supplementary file 2 — Additional file 2. Raw data of western blot. [file 12974_2022_2632_MOESM2_ESM.zip › supplementary files/supplementary figure1/gapdh 2020.05.12_16.57.51_Ch/gapdh 2020.05.12_16.57.51_Ch-Marker.jpg]

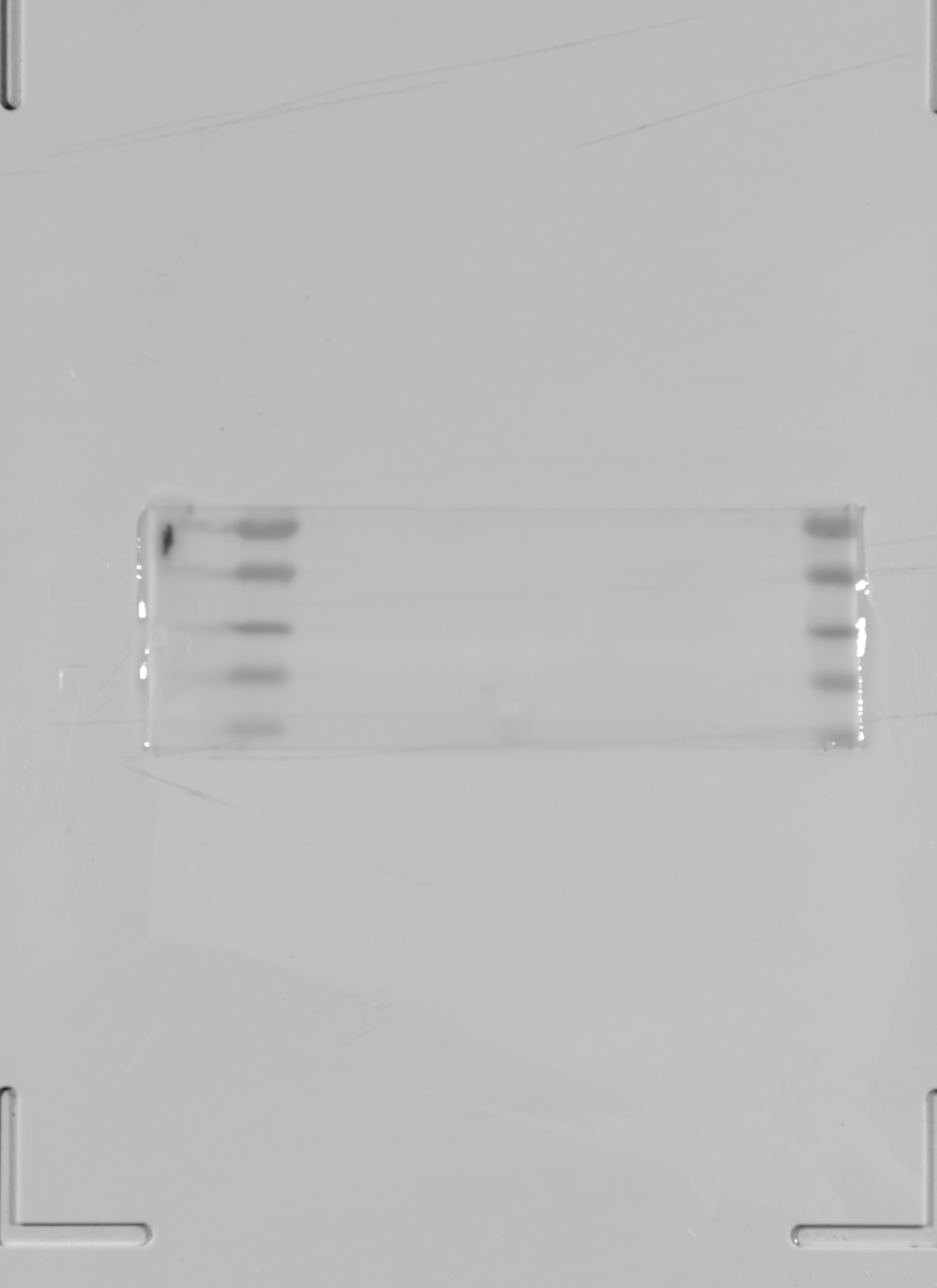

Supplement: Supplementary file 2 — Additional file 2. Raw data of western blot. [file 12974_2022_2632_MOESM2_ESM.zip › supplementary files/supplementary figure1/gapdh 2020.05.12_16.57.51_Ch/gapdh 2020.05.12_16.57.51_Ch-Marker.tif]
